# Supplementary material for: Food additive emulsifiers and cancer risk: Results from the French prospective NutriNet-Santé cohort
Source: PLoS Med. 2024 Feb 13;21(2):e1004338. doi: 10.1371/journal.pmed.1004338 (PMC10863884; doi:10.1371/journal.pmed.1004338)
Supplement: S1 Appendix — eFigure A: Correlations between intakes of food additive emulsifiers among participants from the NutriNet-Santé cohort, 2009–2021 (n = 92,000); eMethod A: Method for the identification of underreporters of energy intake; eMethod B: Detailed quantitative assessment of emulsifiers; eMethod C: Method for multiple imputation of missing values; eMethod D: Method for deriving emulsifier patterns by principal component analysis and corresponding factor loadings; eMethod E: Sensitivity analyses for the associations between food additive emulsifier intakes and cancer risks; eMethod F: Assessment of the proportional hazard assumption in multivariable Cox models using the Schoenfeld residual method; eFigure B: Correlations between Schoenfeld residuals and timescale (age, y) from multivariable Cox models between emulsifier intakes and overall, overall breast, premenopausal breast, postmenopausal breast, and prostate cancer risks in participants from the NutriNet-Santé cohort, 2009–2021 (n = 92,000); eMethod G: Dose-response analyses using restricted cubic splines; eFigure C: Restricted cubic spline plot for the linearity assumption of the association between emulsifier intakes and risks of overall, overall breast, premenopausal breast, postmenopausal breast, and prostate cancers in participants from the NutriNet-Santé cohort, 2009–2021 (n = 92,000); eFigure D: Cumulative incidence functions of the association between emulsifier intakes and risks of overall, breast, and prostate cancers, respectively, in the NutriNet-Santé cohort using Fine–Gray models, 2009–2021 (n = 92,000); eTable A: Detailed contribution of 24 food groups to emulsifier intakes among participants from the NutriNet-Santé cohort, 2009–2021 (n = 92,000); eTable B: Mean daily emulsifier intakes in mg/d (SD) among study participants from the NutriNet-Santé cohort, 2009–2021 (N = 92,000); eTable C: Absolute risks of cancer at 60 years old according to categories of emulsifier intakes at the same age, NutriNet-Santé [file pmed.1004338.s002.docx]

## **S1 Appendix**

**[eFigure A](#eFigure1).** Correlations between intakes of food additive emulsifiers among participants from the NutriNet-Santé cohort, 2009-2021 (n=92,000).

## [**eMethods**](#eMethods)

[eMethod A.](#eMethod1) Method for the identification of under-reporters of energy intake

[eMethod B.](#eMethod2) Detailed quantitative assessment of emulsifiers

[eMethod C](#eMethod3). Method for multiple imputation of missing values

[eMethod D.](#eMethod4) Method for deriving emulsifier patterns by principal component analysis and corresponding factor loadings

[eMethod E.](#eMethod5) Sensitivity analyses for the associations between food additive emulsifier intakes and cancer risks

[eMethod F.](#eMethod6) Assessment of the proportional hazard assumption in multivariable Cox models using the Schoenfeld residual method

[eFigure B.](#eFigure2) Correlations between Schoenfeld residuals and timescale (age, y) from multivariable Cox models between emulsifier intakes and overall, overall breast, premenopausal breast, postmenopausal breast, and prostate cancer risks in participants from the NutriNet-Santé cohort, 2009-2021 (n=92,000).

[eMethod G.](#eMethod7) Dose-response analyses using restricted cubic splines

[eFigure C.](#eFigure3) Restricted cubic spline plot for the linearity assumption of the association between emulsifier intakes and risks of overall, overall breast, premenopausal breast, postmenopausal breast, and prostate cancers in participants from the NutriNet-Santé cohort, 2009-2021 (n=92,000).

[eFigure D.](#eFigure4) Cumulative incidence functions of the association between emulsifier intakes and risks of overall, breast and prostate cancers, respectively, in the NutriNet-Santé cohort using Fine-Gray models, 2009-2021 (n=92,000).

# [**eResults**](#eResults)

[eTable](#eTable1) A. Detailed contribution of 24 food groups to emulsifier intakes among participants from the NutriNet-Santé cohort, 2009-2021 (n=92,000).

[eTable B.](#eTable2) Mean daily emulsifier intakes in mg/d (SD) among study participants from the NutriNet-Santé cohort, 2009-2021 (*N*=92,000)

[eTable C.](#eTable3) Absolute risks of cancer at 60 years old according to categories of emulsifier intakes at the same age, NutriNet-Santé cohort, 2009-2021 (n=92,000).

[eTable D.](#eTable4) Associations between emulsifier intakes and cancer risks among study participants from the NutriNet-Santé cohort, 2009-2021 (n=92,000)

[eTable E.](#eTable5) Sensitivity analyses for the associations between emulsifier intakes and cancer risks among study participants from the NutriNet-Santé cohort, 2009-2021 (n=92,000)

[eTable F.](#eTable6) ‘Any’ versus ‘none’ models for the associations between emulsifier intakes and cancer risks among study participants from the NutriNet-Santé cohort, 2009-2021 (n=92,000)

[eTable G.](#eTable7) Associations between patterns of emulsifier intakes (create with principal component analysis) and cancer risks among study participants from the NutriNet-Santé cohort, 2009-2021 (n=92,000)

**eFigure A.** Correlations between intakes of food additive emulsifiers among participants from the NutriNet-Santé cohort, 2009-2021 (n=92,000).


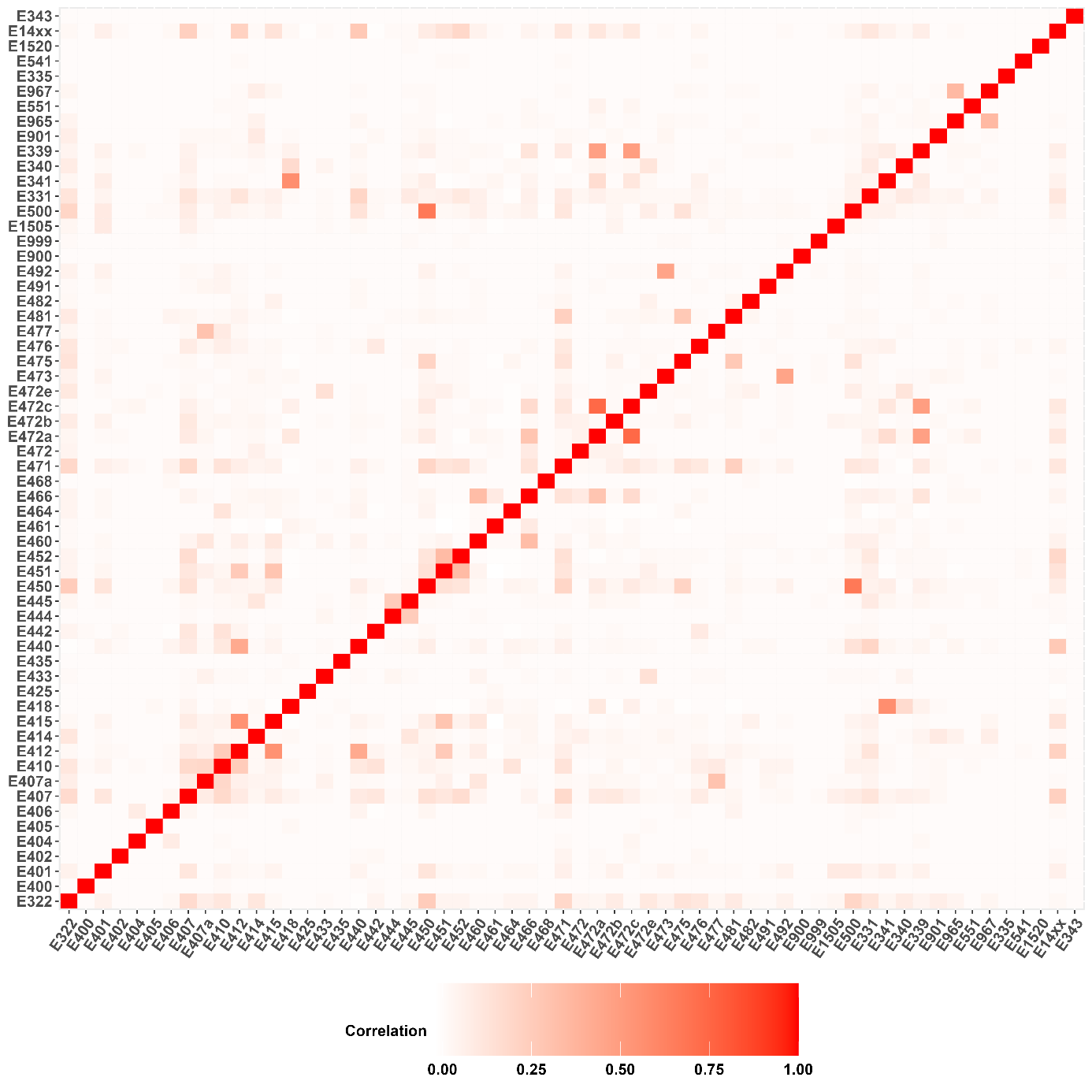


## **eMe****thods**

**e****Method A. Method for the identification of under-reporters of energy intake**

Participants who under-reported their energy intake were excluded from the analyses and were identified using the method from Black,^1,2^ based on the original method developed by Goldberg et al.^3^ This method relies on the hypothesis that the maintenance of a stable body weight requires a balance between energy intake and expenditure. The equations developed by Black account for the reported dietary energy intake, basal metabolic rate (calculated using Schofield’s equations)^4^, sex, age, height, weight, number of dietary records, physical activity level (PAL), and intra/inter-individual variability. As recommended by Black, the intra-individual coefficients of variations for BMR and PAL were fixed at 8.5 % and 15%, respectively. In addition, a PAL of 1.55 was used to reflect a “light” physical activity which is assumed to be attained by healthy, normally active individual living a sedentary lifestyle. Finally, some individuals identified as under-reporters of energy intakes using Black’s method were not excluded, if they also reported recent weight variations, adherence to weight-loss restrictive diets, or declared the consumptions entered in their dietary records as unusually low compared to their habitual diets.

In this study 21,423 participants (corresponding to 16.5% of the subjects) were considered as under-energy reporters and were excluded from the study. This proportion of under-reporters is common, for instance in the nationally representative INCA 3 study conducted in 2016 by the French Food Safety Agency ^5(p3)^ 18% of adult participants were identified as under-reporters using the Black method.

Several quality control operations were performed to account for over-reporting. Limitations in the online tool were set when participants reported the quantities of food consumed, aiming to alert them that the number they were about to enter was potentially an outlier, thereby encouraging double check and correction. Later on, during the data cleaning process, limitations were set per food category within one eating episode and per record for quantities; for instance, limitations for fruits were set for 3000 grams/day, 1500 grams/day for fish, 2000 grams/day for yoghurts, etc… if more than 10% of reported food items had outliers, then the full record was excluded. Otherwise, values were corrected to the maximum authorised values or standardised.

This study included a sample of 92,000 participants with an average of 6.0 completed 24-hour dietary records (SD 3.0) during their first two years of participation. The distribution of the number of dietary records per participant is detailed in the table below:

| **Number of dietary records** | **Percentage** | **Cumulative percentage** |
| --- | --- | --- |
| **3** | 39.13 | 39.13 |
| **4** | 4.56 | 43.70 |
| **5** | 4.57 | 48.27 |
| **6** | 15.43 | 63.70 |
| **7** | 2.8 | 66.50 |
| **8** | 4.02 | 70.52 |
| **9** | 22.61 | 93.13 |
| **10** | 0.67 | 93.80 |
| **11** | 0.74 | 94.54 |
| **12** | 3.79 | 98.33 |
| **13** | 0.18 | 98.51 |
| **14** | 0.27 | 98.78 |
| **15** | 1.16 | 99.94 |
| **16** | 0.01 | 99.95 |
| **17** | 0.02 | 99.97 |
| **18** | 0.03 | 99.99 |
| **21** | 0.001 | 100.00 |

**eMethod B. Detailed quantitative assessment of emulsifiers**

The strength of our methodology relies in the precise qualitative assessment of additive exposure, i.e., presence/absence of a specific emulsifier in the food consumed. This unique level of detail is permitted by the fact that commercial names/brands of industrial product consumed were collected and matched with Open Food Facts, Oqali and GNPD databases providing the ingredient list and thus, presence of the specific emulsifier), at the time when the product was consumed. Thus, we only attribute a non-null dose of a specific additive to a given product declare by a participant if this specific product contains this specific additive.

Then, the quantitative assessment of the doses of additives in the products which contain a specific additive is challenging since manufacturers are not compelled to declare this information on the packaging. Hence the 3-step method used to assess doses in our cohort. In all, in the framework of the ADDITIVES project, we performed 2677 quantified analyses, corresponding to a total of 61 food additives in 196 different (generic) food items. “Pairs” (i.e. a specific additive in a specific food vector) selected for laboratory assays corresponded to the most frequently consumed and most emblematic commercial food/beverage items for a given additive. Specifically, for emulsifiers, we had access to 501 laboratory quantified analyses corresponding to 9 emulsifiers (E322, E339, E340, E341, E343, E440, E450, E451, E452) in 37 (generic) food items (several commercial brands were tested per food item, e.g., in the case of milk chocolate, milk chocolate with nuts, creamy desserts, omega-3 enriched margarines, sausages, jams, chocolate mousse…). In addition to the assays carried out by certified laboratories, which were sent to us by the consumer association UFC Que Choisir, we contacted two companies (Mérieux & Eurofins) and the Direction Générale de la Consommation, de la Concurrence et de la Répression des Fraudes (DGCCRF) to carry out these assays. Only the additives listed in their catalogue could be measured. In case data was not available from this source, EFSA and GSFA doses were only applied if the specific food item did actually contain the specific emulsifier in the ingredients list. We used 1497 emulsifier data from EFSA (data available online in each EFSA Opinion + transmission of specific information by EFSA following an official Public Access to Document request PAD 2020/077), related to 58 food additive emulsifiers present in 237 food categories. EFSA collects many information from manufacturers related to their specific commercial products but for confidentiality reasons, only transfers information for generic food items or food groups (no brand-specific data). As regards GSFA, we used 5352 emulsifier data concerning 45 food additive emulsifiers coming from 226 food categories. As for EFSA, data from GSFA are not brand-specific but relate to generic food items or food categories.

**eMethod C. Method for multiple imputation of missing values**

Missing values for covariates were handled using multiple imputation by additive regression, bootstrapping, and predictive mean matching (n=20 imputed datasets) as implemented in the *Hmisc* R package.^6^ Missing values were imputed for the following variables: physical activity level (14.0% of missing values), number of cigarettes smoked in pack/year (0.02%), smoking status (0.1%), education level (0.9%), BMI (2.1%), height (2.1%), and family history of cancer (1.3%).

**eMethod D. Method for deriving emulsifier patterns by principal component analysis and corresponding factor loadings**

Emulsifier patterns were identified based on 13 emulsifiers (having shown at least one significant association with cancer risk) using a principal component analysis conducted with the R package *FactoMineR*.^7^ The principal component analysis creates linear combinations (called principal components) of the initial set of variables, with the aim to group those that are correlated while explaining as much variation from the dataset as possible. We used the scree plot generated by the principal component analysis to select the retained principal components (with eigen values ≥2). For easier interpretation, we used the R “varimax” option to rotate the principal components orthogonally and maximise the independence of the retained principal components.

The variable coefficients derived from the selected principal components are called factor loadings. A positive factor loading indicates a positive contribution of the variable to the principal component, whereas a negative factor loading indicates a negative contribution. For the interpretation of the three principal components selected, we considered the variables contributing the most to the component, i.e. with loading coefficients under -0.25 or over 0.25. Finally, we calculated an adherence score to each principal component and for each participant, using the emulsifier factor loadings to weigh the sum of all observed intakes. Thus, the adherence score measures a participant’s diet conformity to the identified emulsifier intake pattern.

We identified 3 components explaining respectively 17.5, 13.6 and 9.6% of the variance. The first component was characterized by higher exposure to carrageenans, E410, E412, E415, E450, E500 and E471. The second component was characterized by higher exposure to E450 and E500 and low exposure to carrageenans, while the third one was characterized by high exposure to E412 and E440 and low exposure to carrageenans.

| **Emulsifiers** |  | **Factor loadings** | |
| --- | --- | --- | --- |
|  | **Comp 1** | **Comp 2** | **Comp 3** |
| **Total carrageenans** | **0.78** | **-0.38** | **-0.44** |
| **E340** | 0.07 | -0.04 | -0.07 |
| **E407** | **0.78** | **-0.38** | **-0.45** |
| **E410** | **0.39** | -0.17 | 0.14 |
| **E412** | **0.45** | -0.21 | **0.77** |
| **E414** | 0.08 | 0.03 | -0.02 |
| **E415** | **0.34** | -0.14 | **0.6** |
| **E440** | **0.35** | -0.03 | **0.47** |
| **E450** | **0.5** | **0.73** | -0.04 |
| **E471** | **0.4** | 0.15 | -0.08 |
| **E475** | 0.2 | **0.39** | -0.03 |
| **E500** | **0.43** | **0.74** | -0.004 |
| **E901** | 0.06 | 0.04 | -0.03 |

**eMethod E. Sensitivity analyses for the associations between food additive emulsifier intakes and cancer risks**

A series of sensitivity analyses was conducted for all emulsifiers with at least one statistically significant association with cancer risk in the main model. I.) Based on the main model, model 1 included a mutual adjustment for the rest of emulsifiers (continuous, mg/d), model 2 included adjustment for the proportion of UPF in the diet (in % weight, continuous), model 3 included adjustment for total artificial sweeteners (continuous, mg/d), and model 4 used emulsifier intakes estimated from the average of all available 24h dietary records throughout the follow-up of each participant (up to 62 records per participant) instead of averaged values on their first two years of follow-up. II.) The False Discovery Rate (FDR) was used to adjust p-values from the main model for multiple testing. III.) We also tested 1) an “any versus none” approach by combining the medium and higher exposure categories, and compared cancer hazards in this category with the low/non-exposed category, 2) using principal component analysis for patterns of emulsifiers instead of individual exposures.

**eMethod F. Assessment of the proportional hazard assumption in multivariable Cox models using the Schoenfeld residual method**

The Schoenfeld residual method from the *survival*^9^ R package was used to test the proportional hazard assumption when performing Cox proportional hazard model.^10^ The assumption is verified if there is no statistically significant correlation between the Schoenfeld residuals and time.

**eFigure B**. Correlations between Schoenfeld residuals and timescale (age, y) from multivariable Cox models between emulsifier intakes and overall, overall breast, premenopausal breast, postmenopausal breast, and prostate cancer risks in participants from the NutriNet-Santé cohort, 2009-2021 (n=92,000).

**A**: Associations between total carrageenans and cancer risks. **B**: Associations between E407 and cancer risks. **C**: Associations between E340 and cancer risks. **D**: Associations between E450 and cancer risks. **E**: Associations between E471 and cancer risks. **F**: Associations between E475 and cancer risks. **G**: Associations between E410 and cancer risks. **H:** Associations between E412 and cancer risks. **I:** Associations between E414 and cancer risks. **J:** Associations between E415 and cancer risks. **K:** Associations between E440 and cancer risks. **L:** Associations between E500 and cancer risks. **M:** Associations between E901 and cancer risks.

Plots on the right correspond to overall cancer and prostate cancer respectively. Plots on the left correspond to breast cancer, premenopausal breast cancer, and postmenopausal breast cancer respectively. Horizontal axis corresponds to age, vertical axis to beta(t) of emulsifier residuals.

**A**: Associations between total carrageenans and cancer risks.


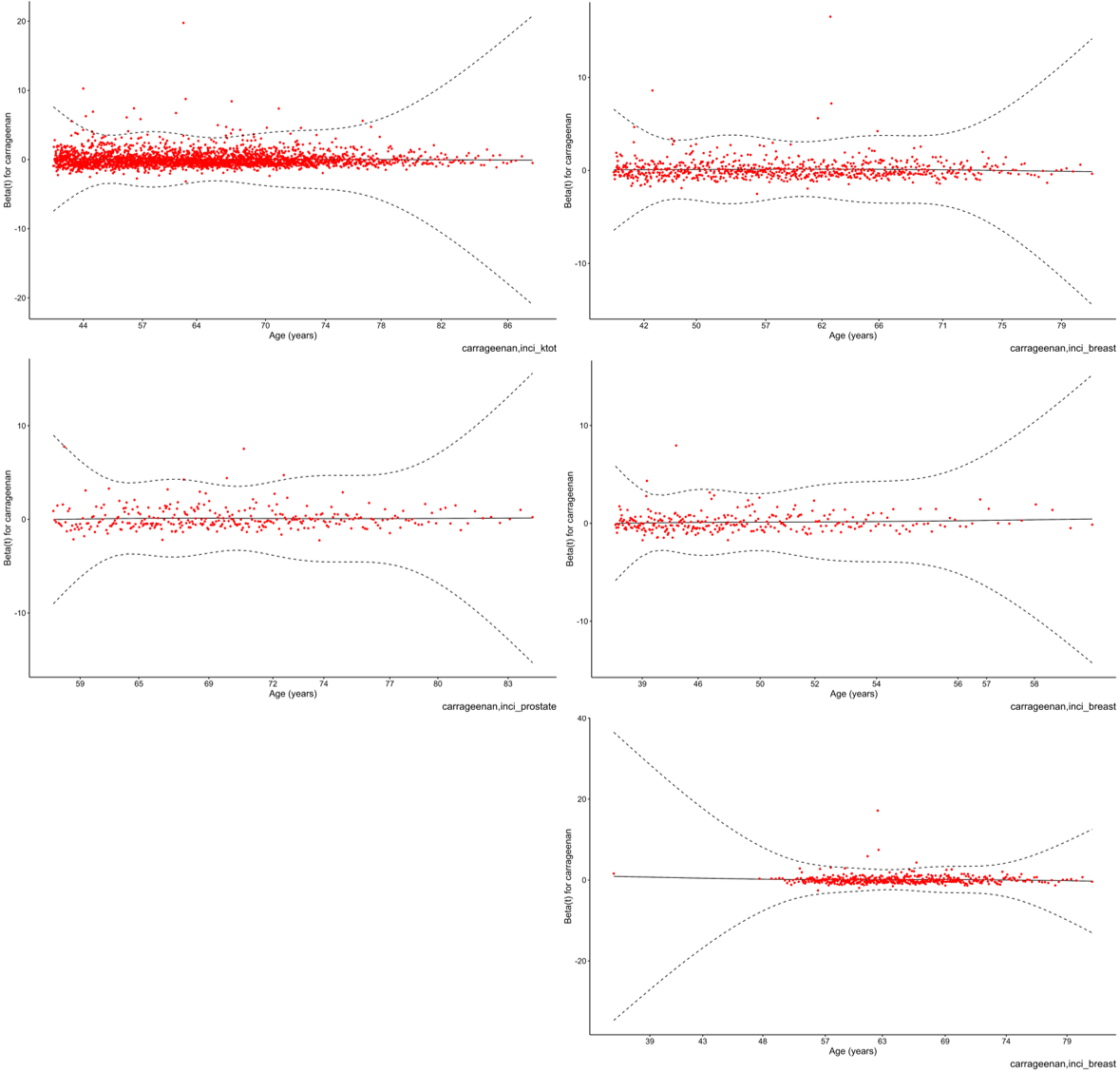


**B**: Associations between E407 and cancer risks.

**
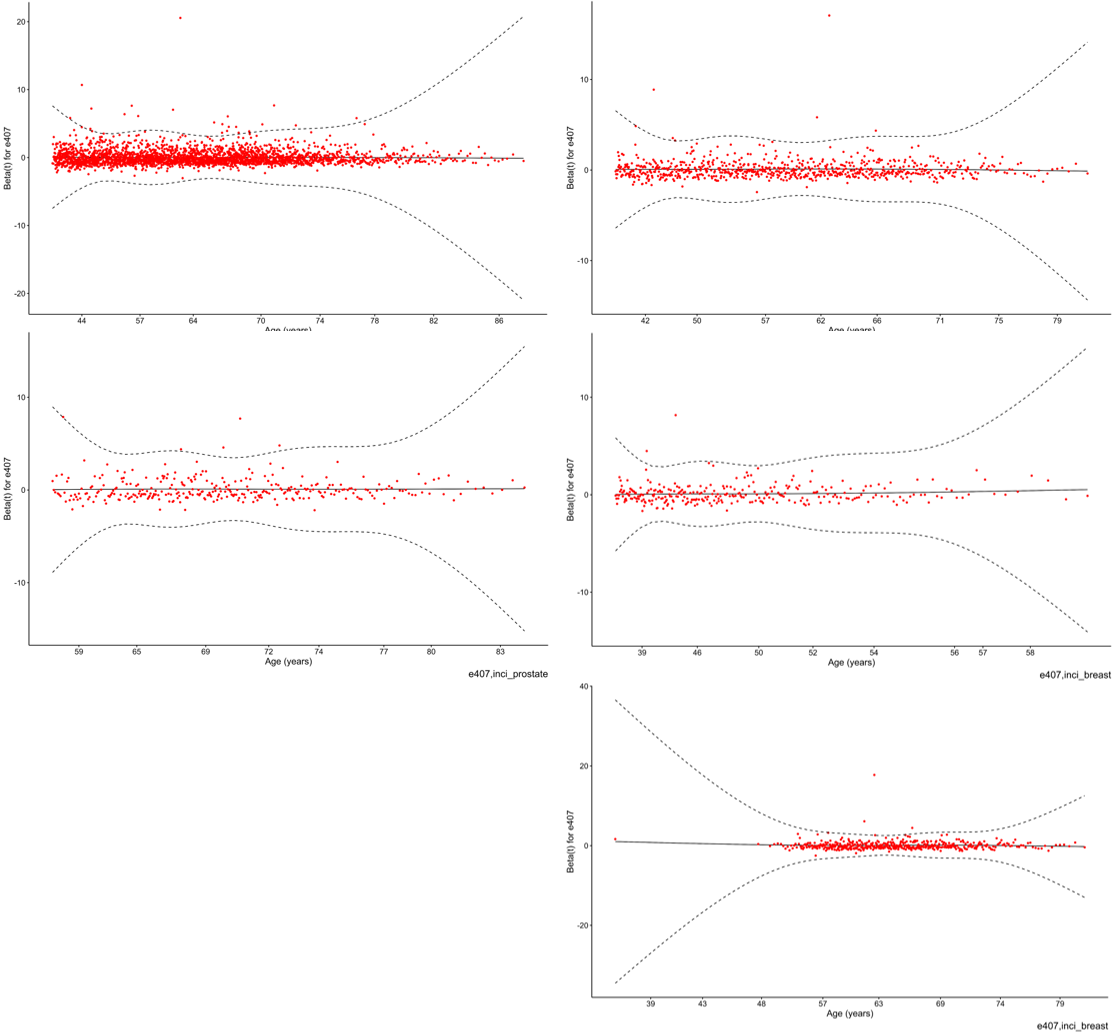
**

**C**: Associations between E340 and cancer risks.

**
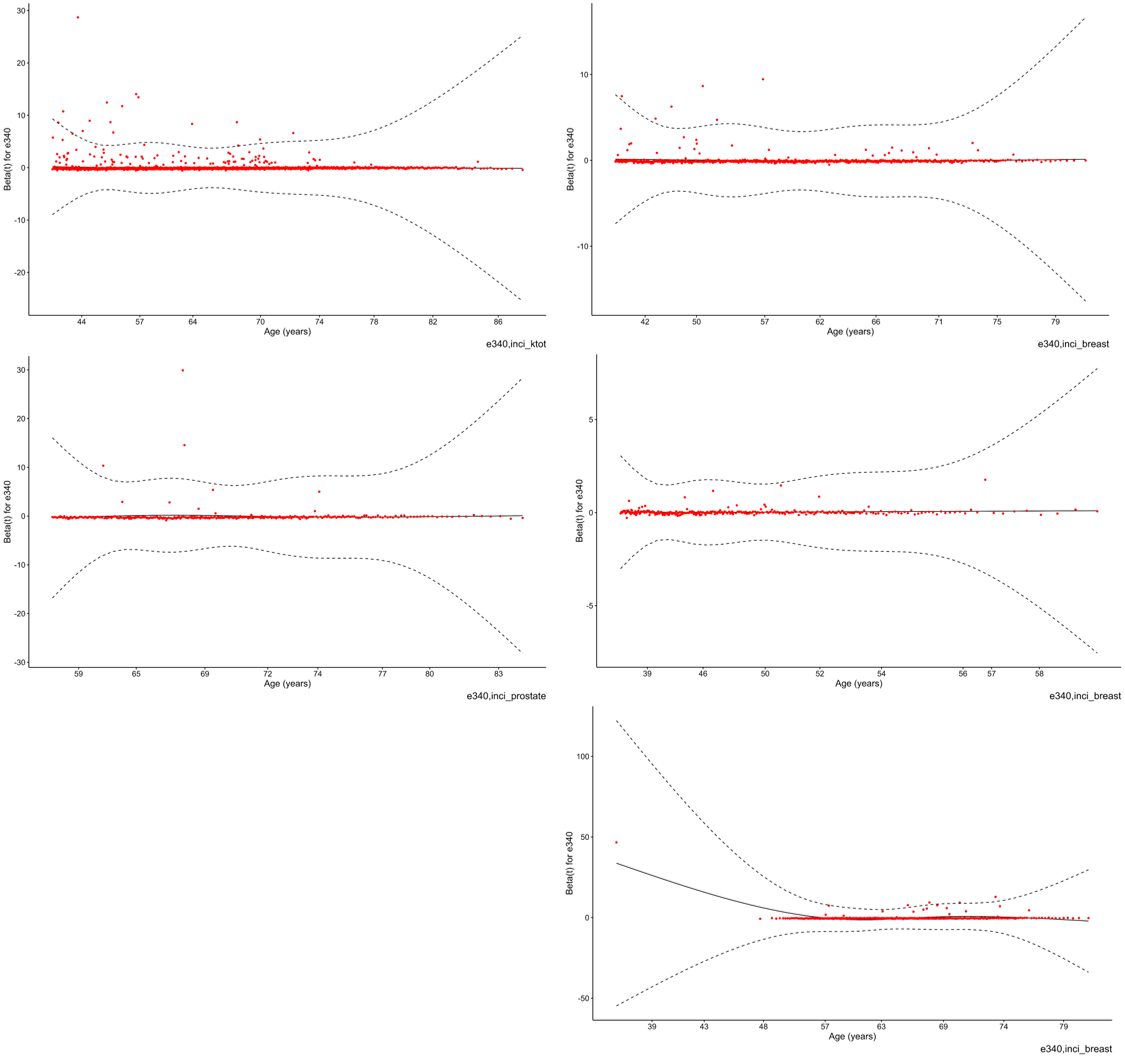
**

**D**: Associations between E450 and cancer risks.

**
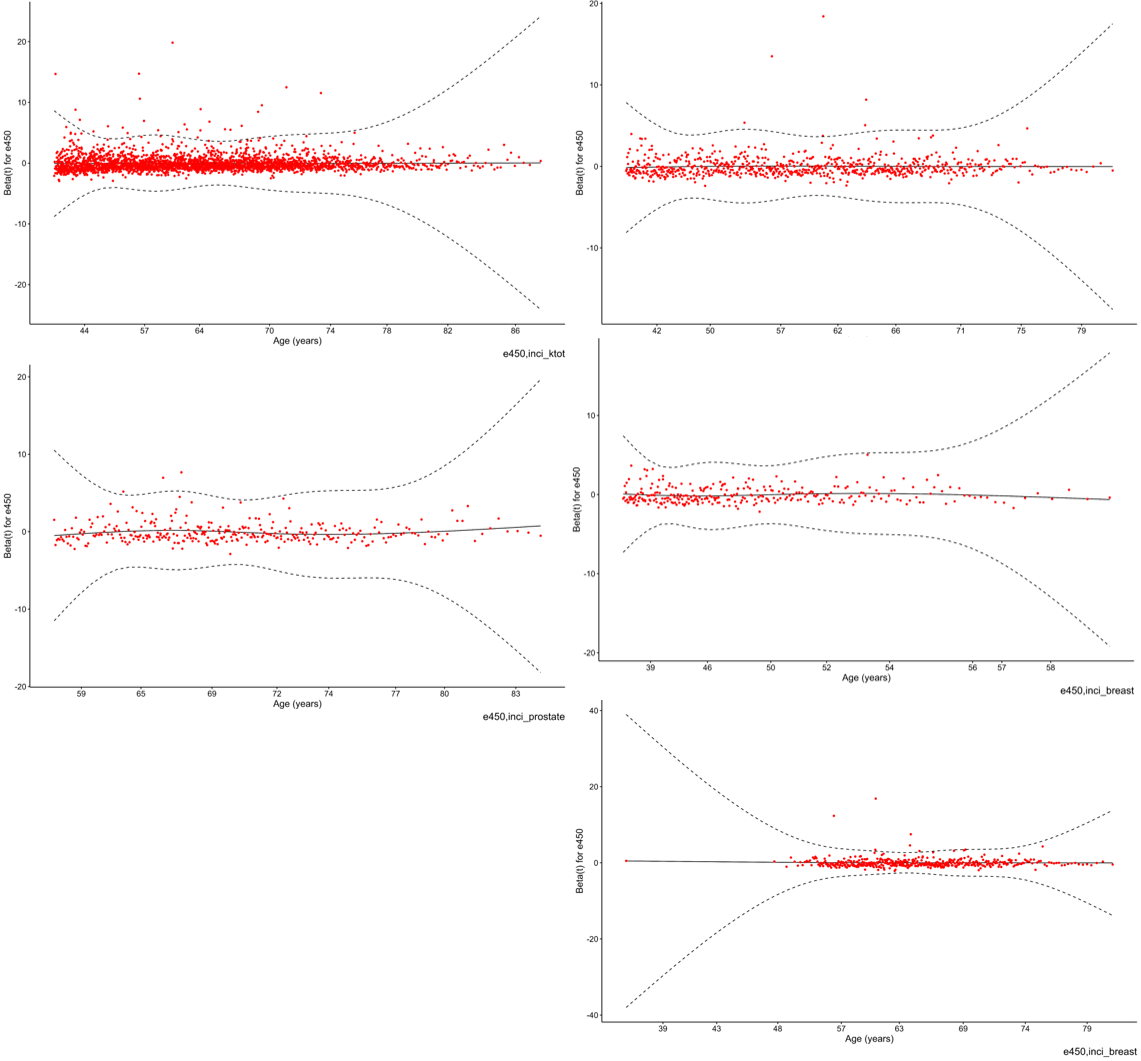
**

**E**: Associations between E471 and cancer risks.

**
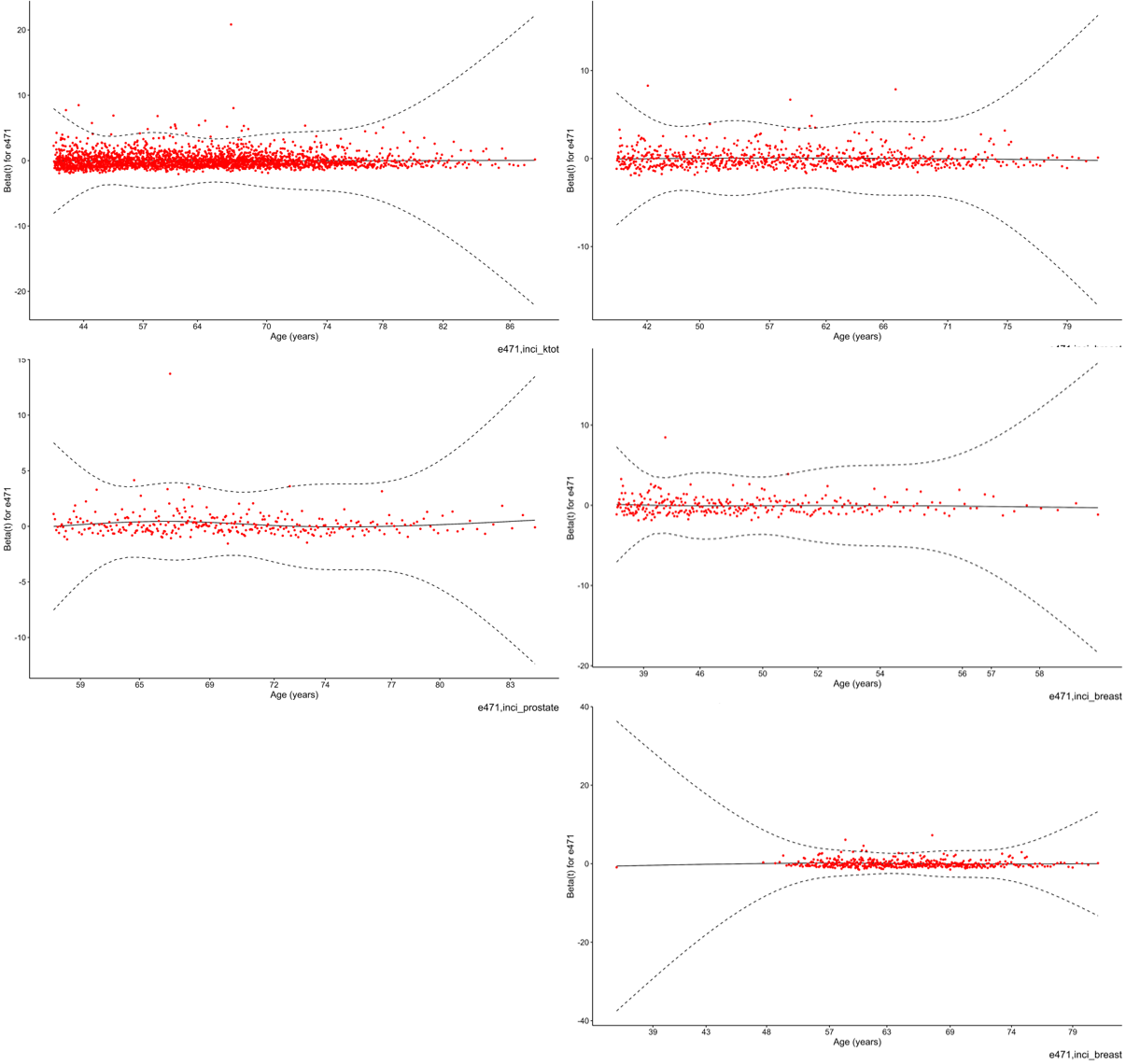
**

**F**: Associations between E475 and cancer risks.

**
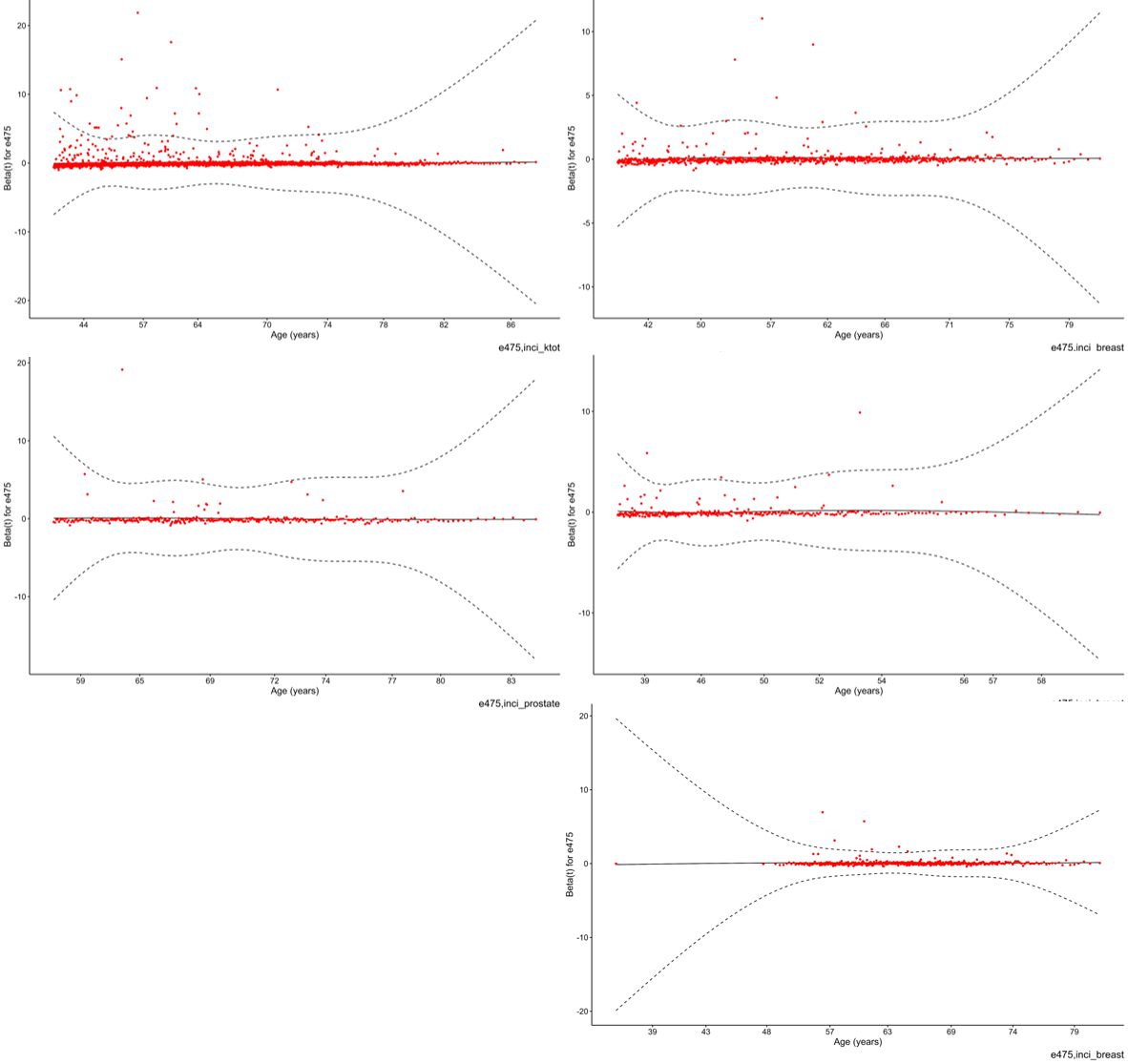
**

**G**: Associations between E410 and cancer risks.

**
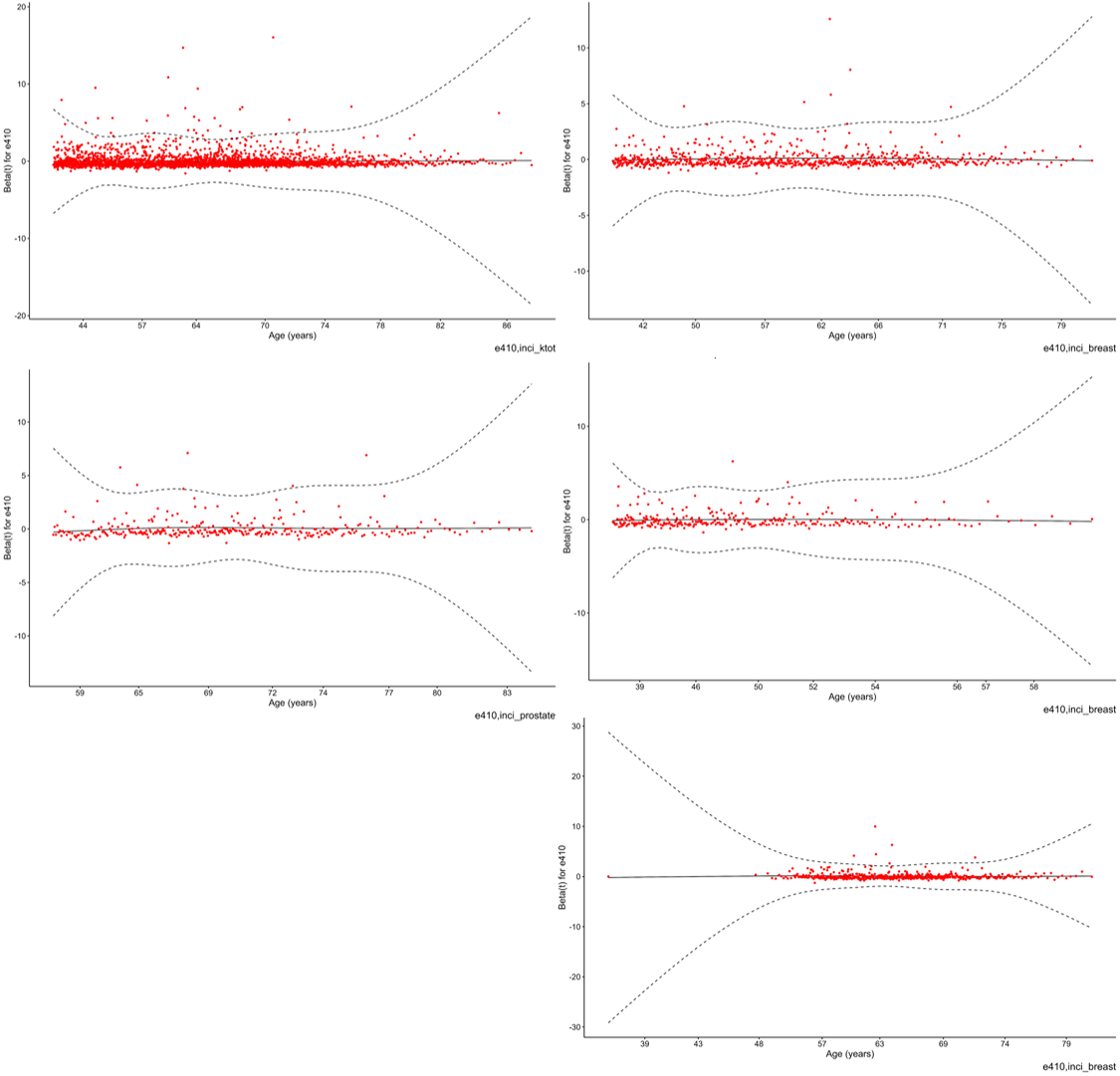
**

**H:** Associations between E412 and cancer risks.

**
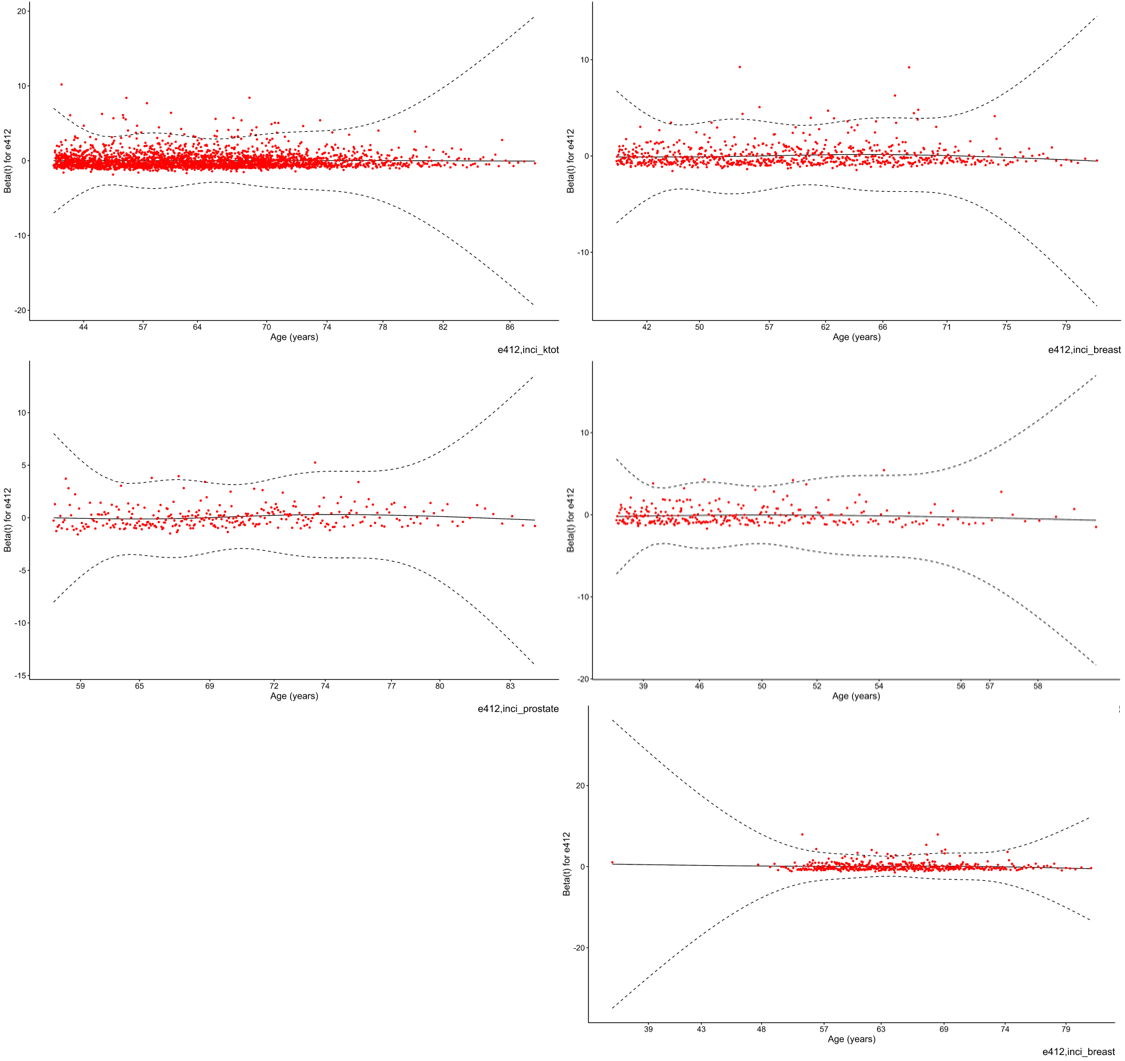
**

**I:** Associations between E414 and cancer risks.

**
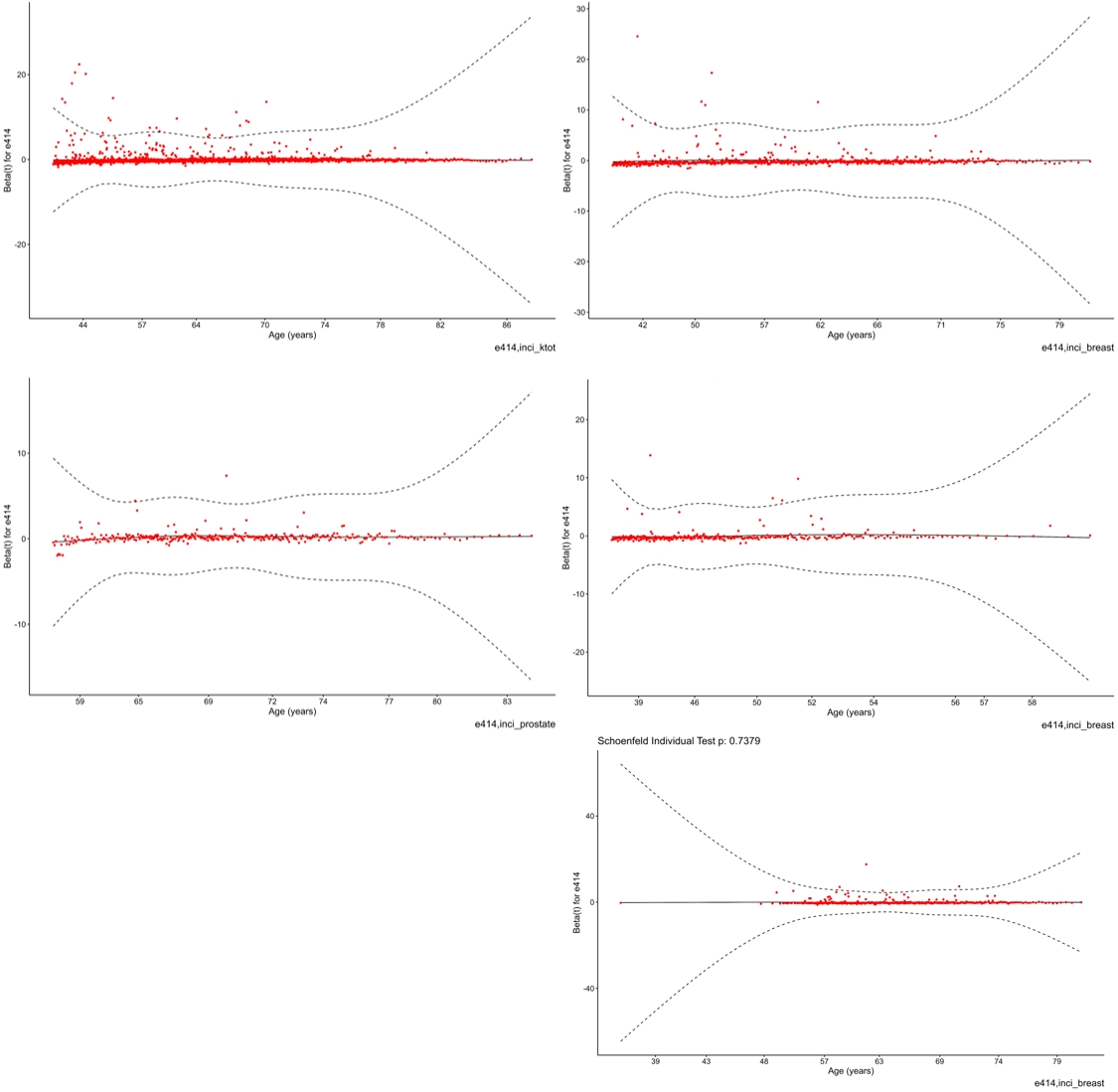
**

**J:** Associations between E415 and cancer risks.

**
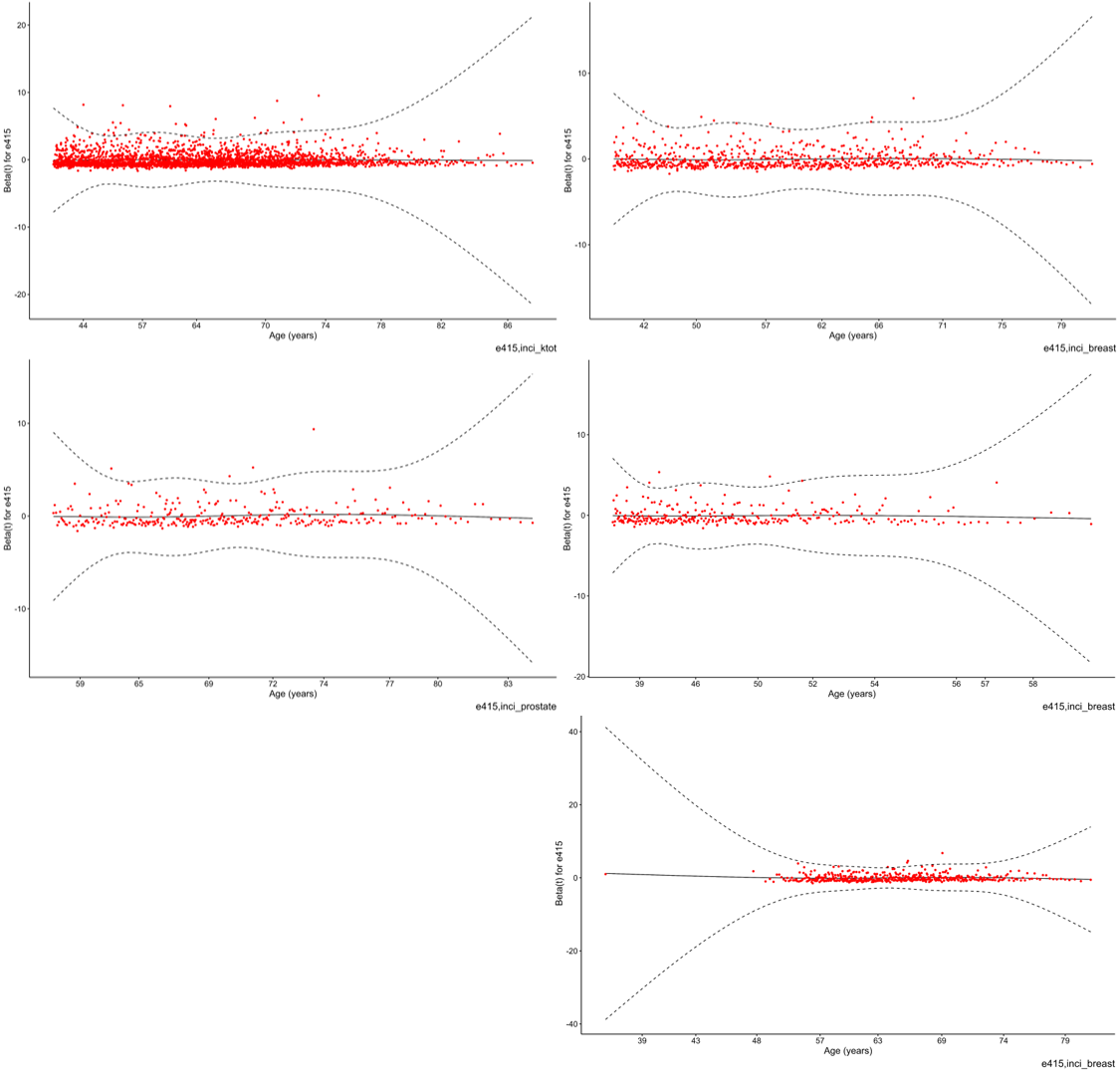
**

**K:** Associations between E440 and cancer risks.

**
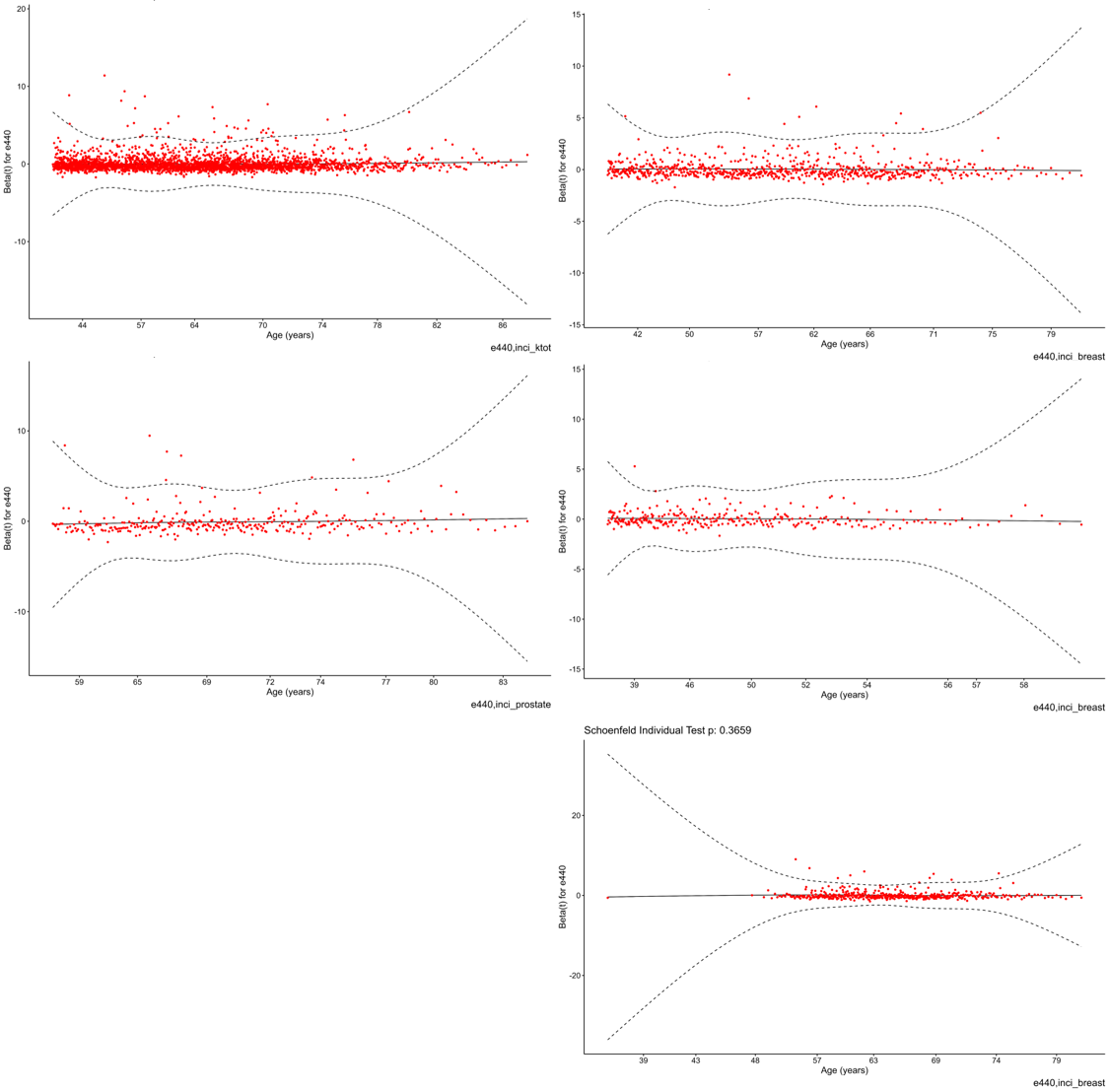
**

**L:** Associations between E500 and cancer risks.

**
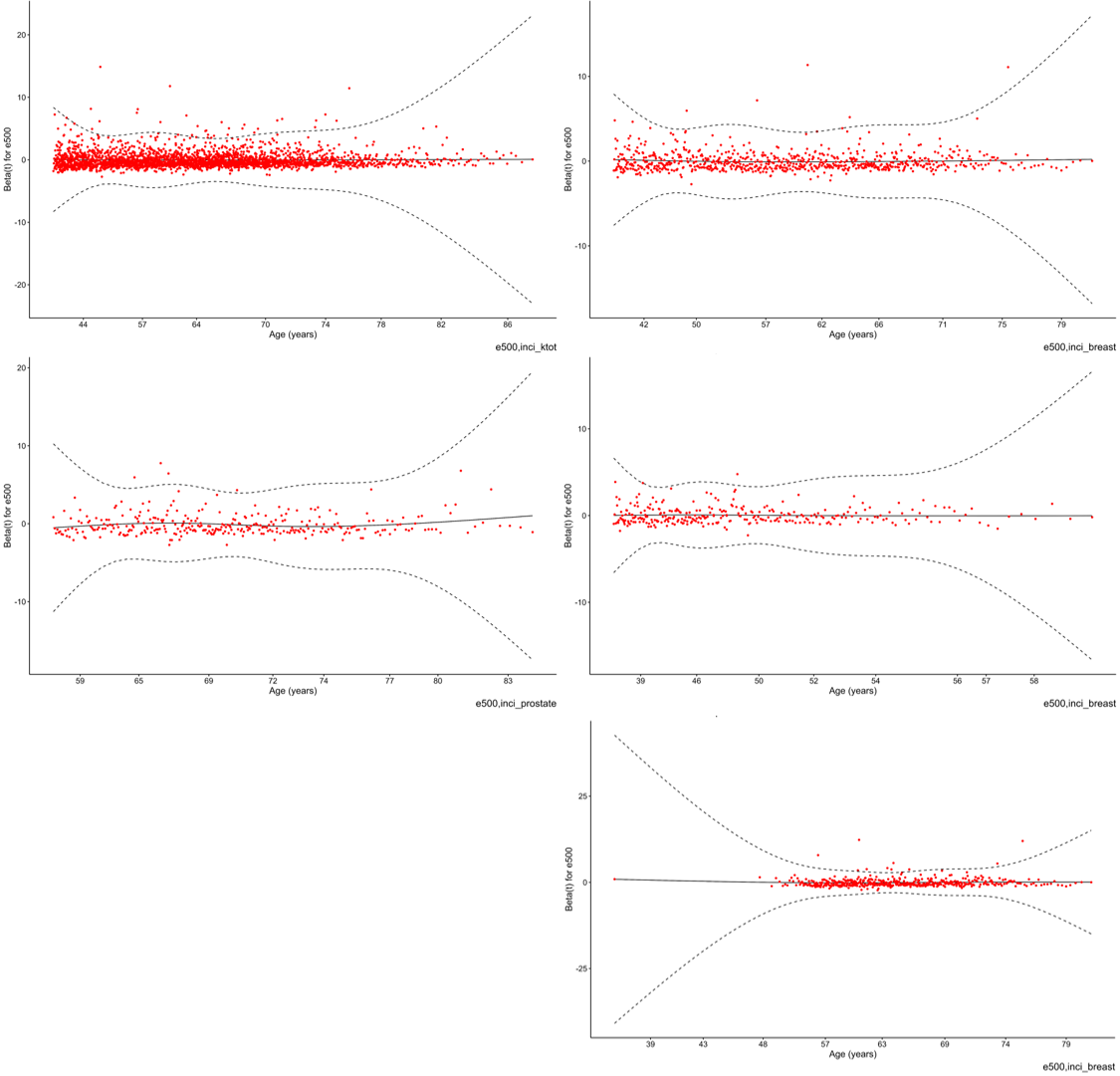
**

**M:** Associations between E901 and cancer risks.

**
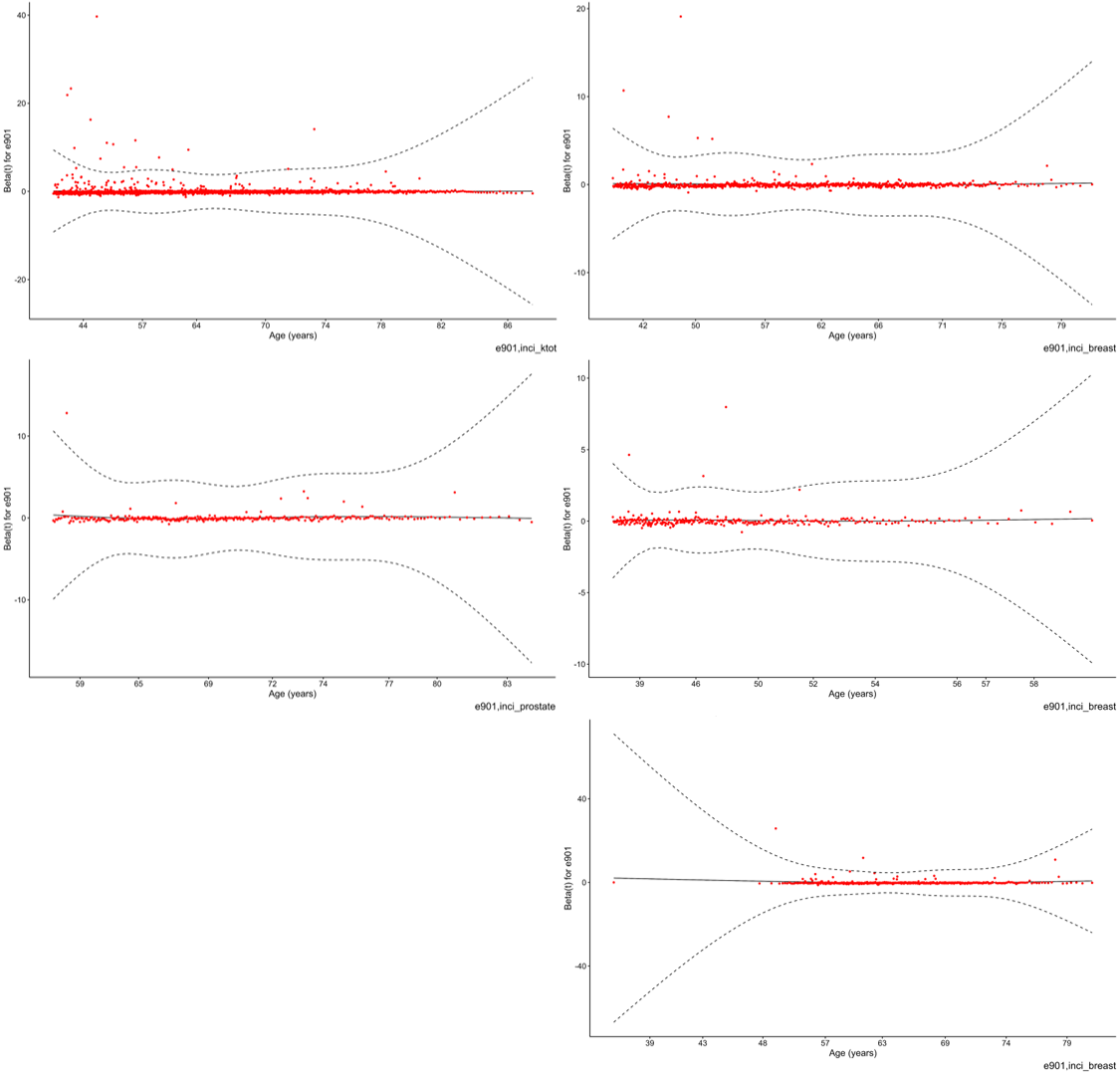
**

**eMethod G. Dose-response analyses using restricted cubic splines**

The dose-response relationships between emulsifier intakes and cancer risks were investigated using restricted cubic spline SAS macro developed by Desquilbet and Mariotti,^11^. The p-values resulting from non-linearity tests along with graphical representation of the associations are presented below.

**eFigure C**. Restricted cubic spline plot for the linearity assumption of the association between emulsifier intakes and risks of overall, overall breast, premenopausal breast, postmenopausal breast, and prostate cancers in participants from the NutriNet-Santé cohort, 2009-2021 (n=92,000).

**A**: Associations between total carrageenans and cancer risks. **B**: Associations between E407 and cancer risks. **C**: Associations between E340 and cancer risks. **D**: Associations between E450 and cancer risks. **E**: Associations between E471 and cancer risks. **F**: Associations between E475 and cancer risks. **G**: Associations between E410 and cancer risks. **H:** Associations between E412 and cancer risks. **I:** Associations between E414 and cancer risks. **J:** Associations between E415 and cancer risks. **K:** Associations between E440 and cancer risks. **L:** Associations between E500 and cancer risks. **M:** Associations between E901 and cancer risks.

Plots on the right correspond to overall cancer and prostate cancer respectively. Plots on the left correspond to breast cancer, premenopausal breast cancer, and postmenopausal breast cancer respectively. Horizontal axis corresponds to daily intakes of emulsifier (in mg/day), vertical axis to ln(HR). p-value = p for non-linearity.

**A**: Associations between total carrageenans and cancer risks.

**
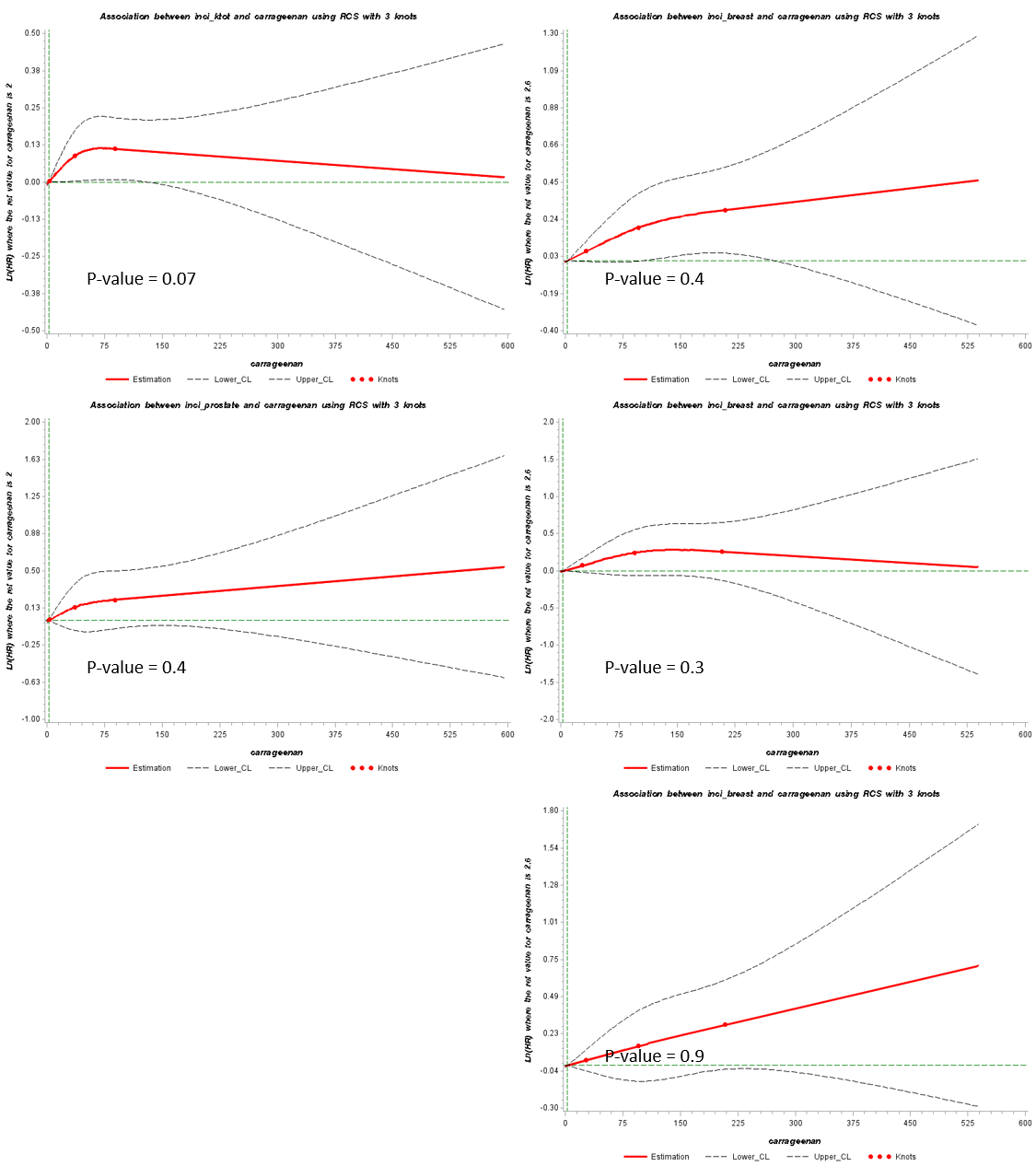
**

**B**: Associations between E407 and cancer risks.

**
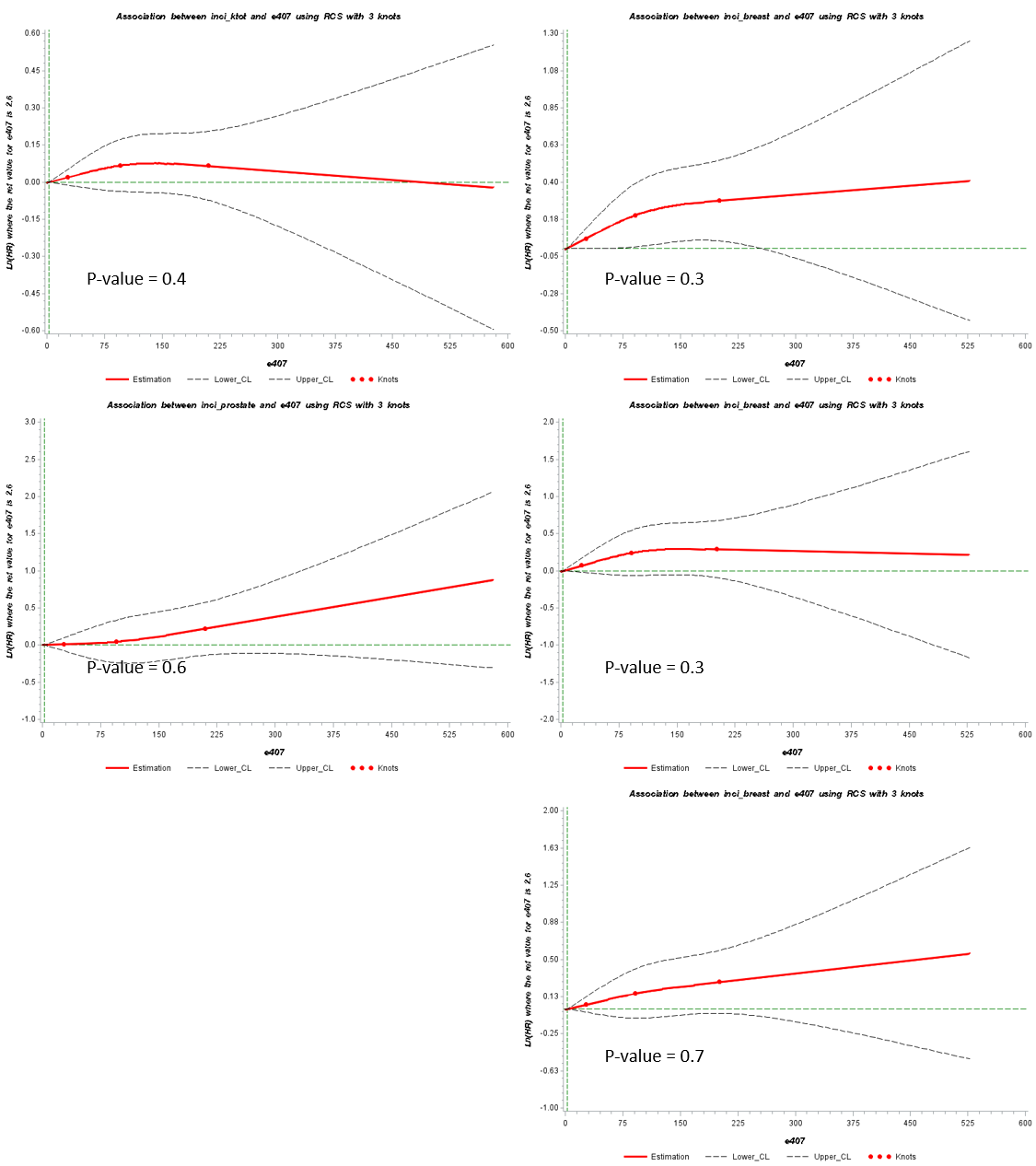
**

**C**: Associations between E340 and cancer risks.

**
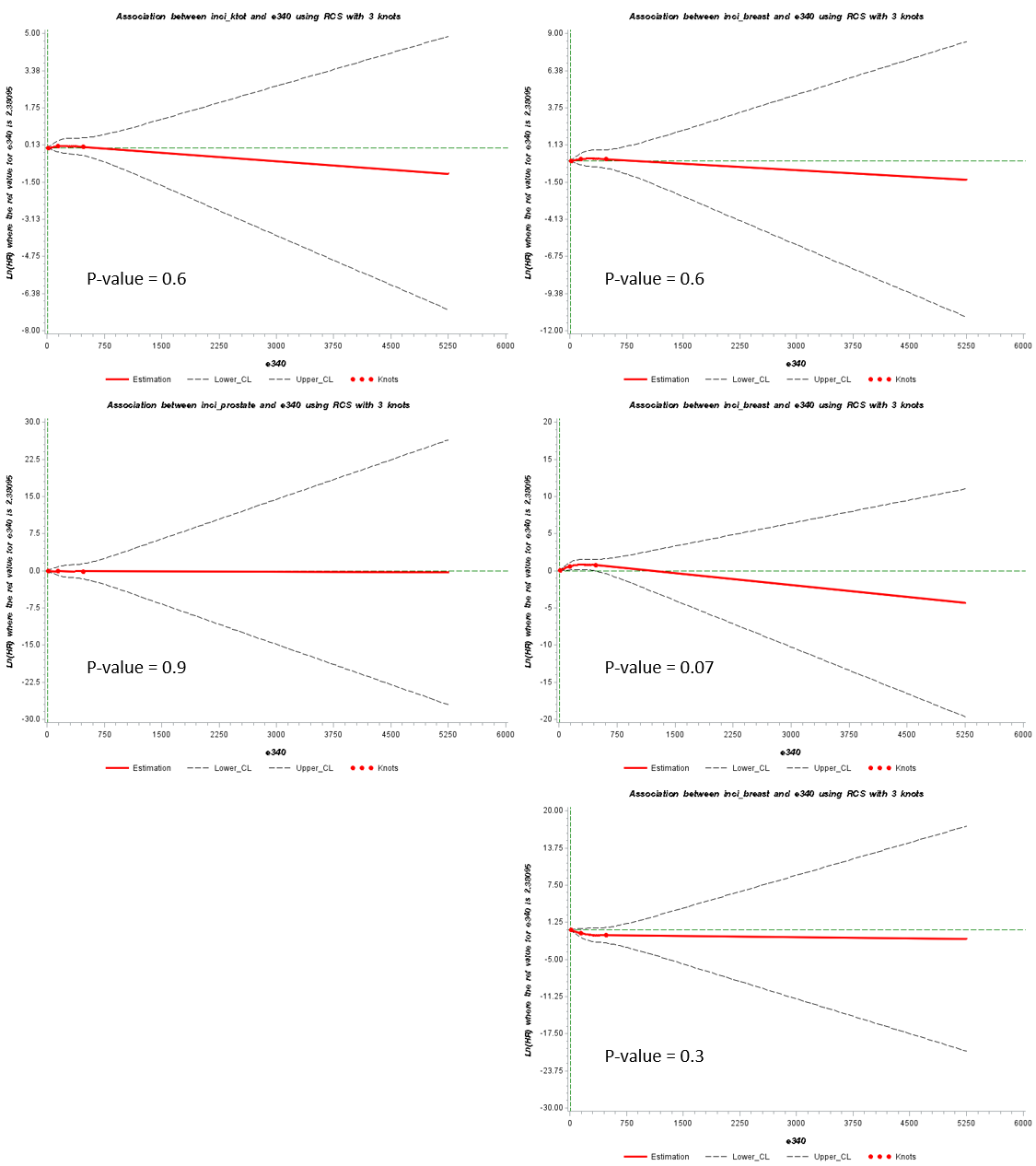
**

**D**: Associations between E450 and cancer risks.

**
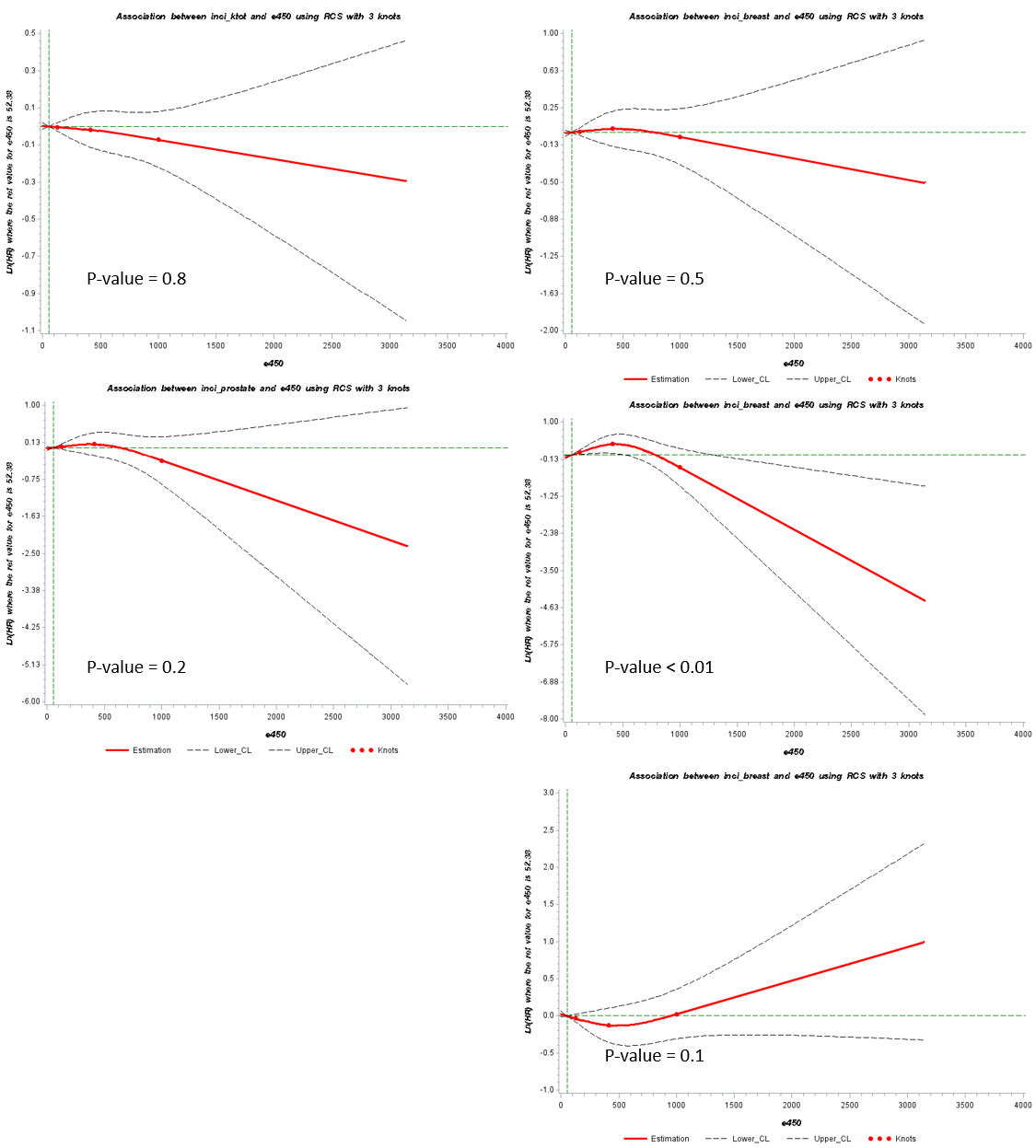
**

**E**: Associations between E471 and cancer risks.

**
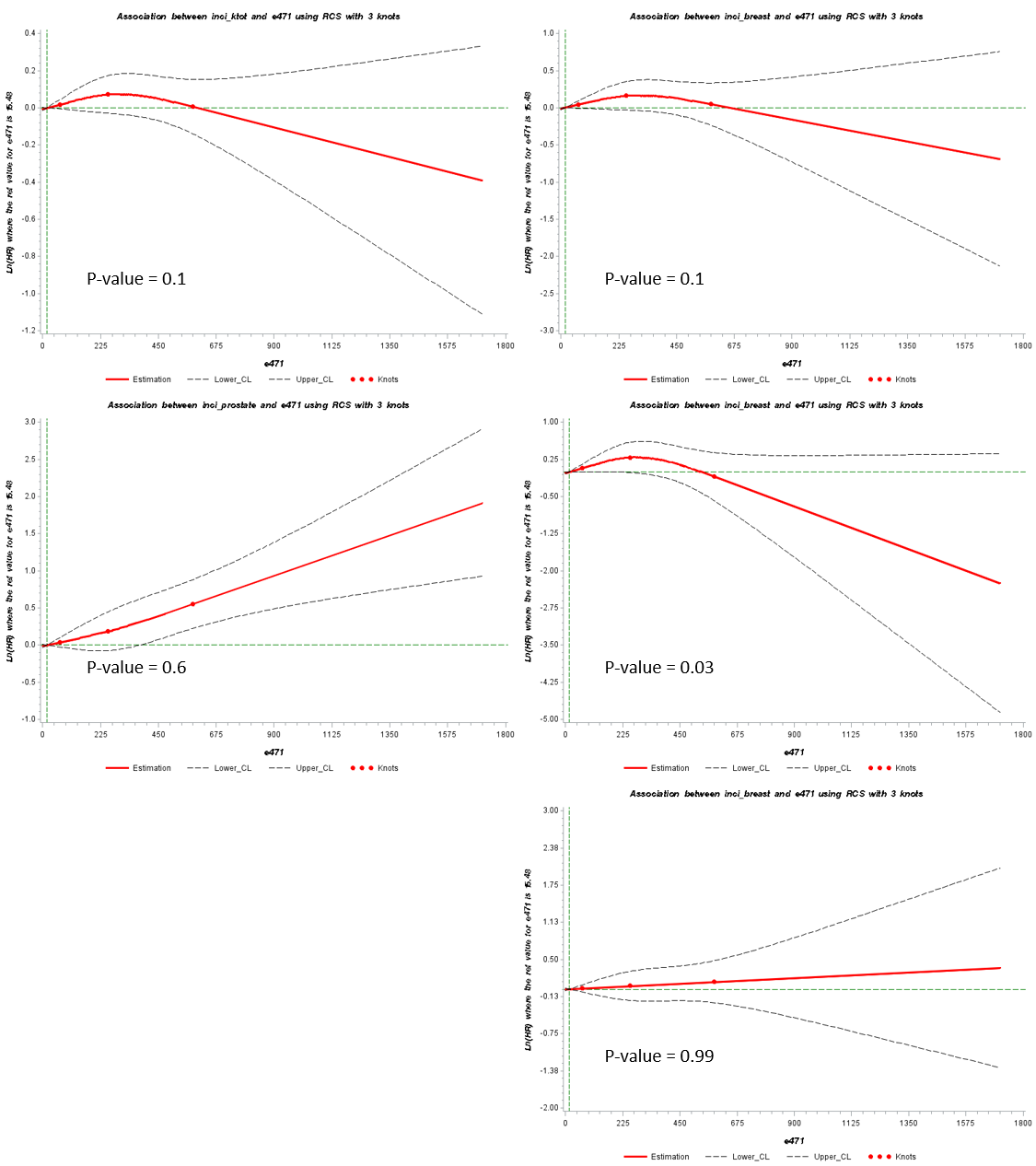
**

**F**: Associations between E475 and cancer risks.

**
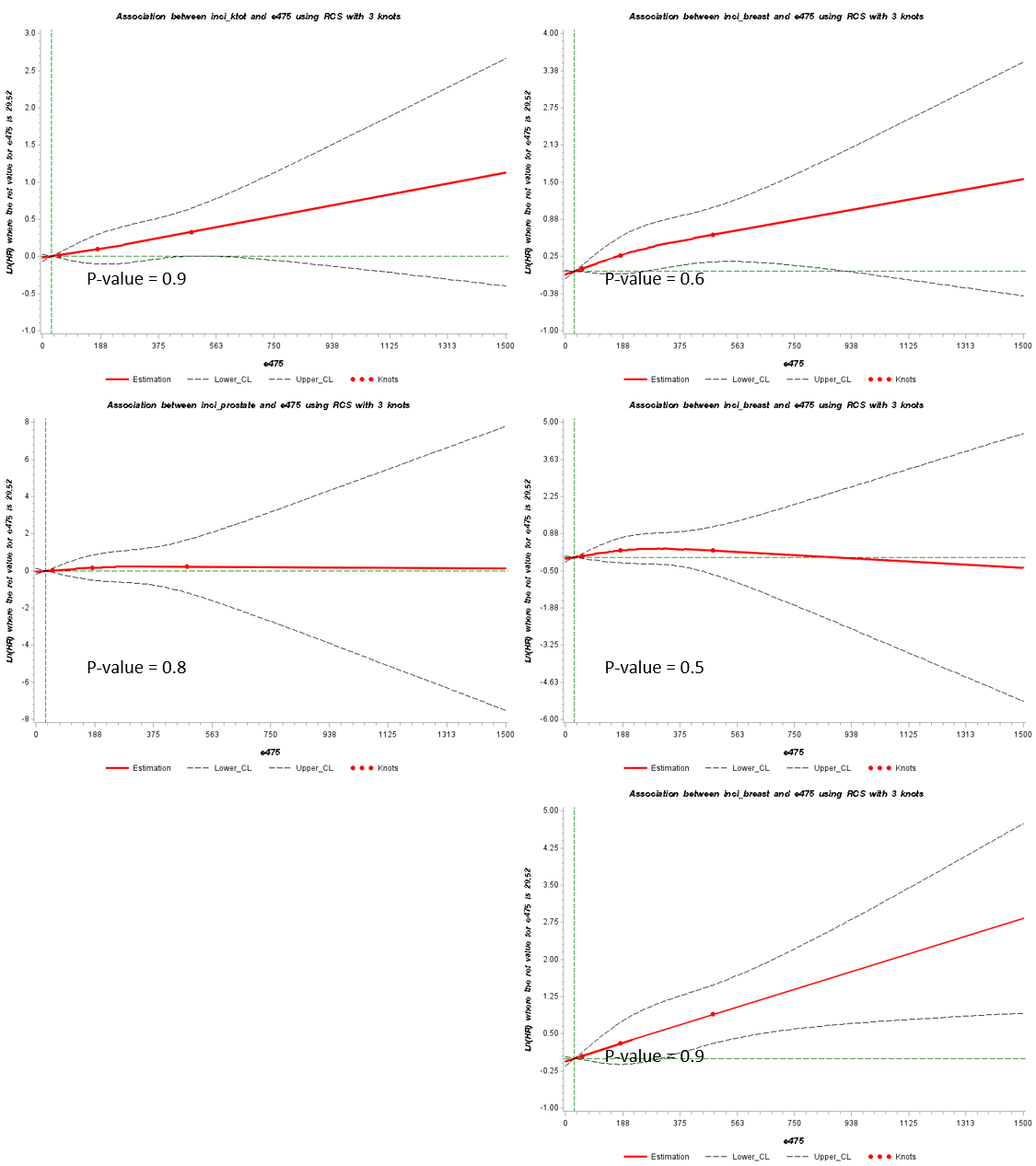
**

**G**: Associations between E410 and cancer risks.

**
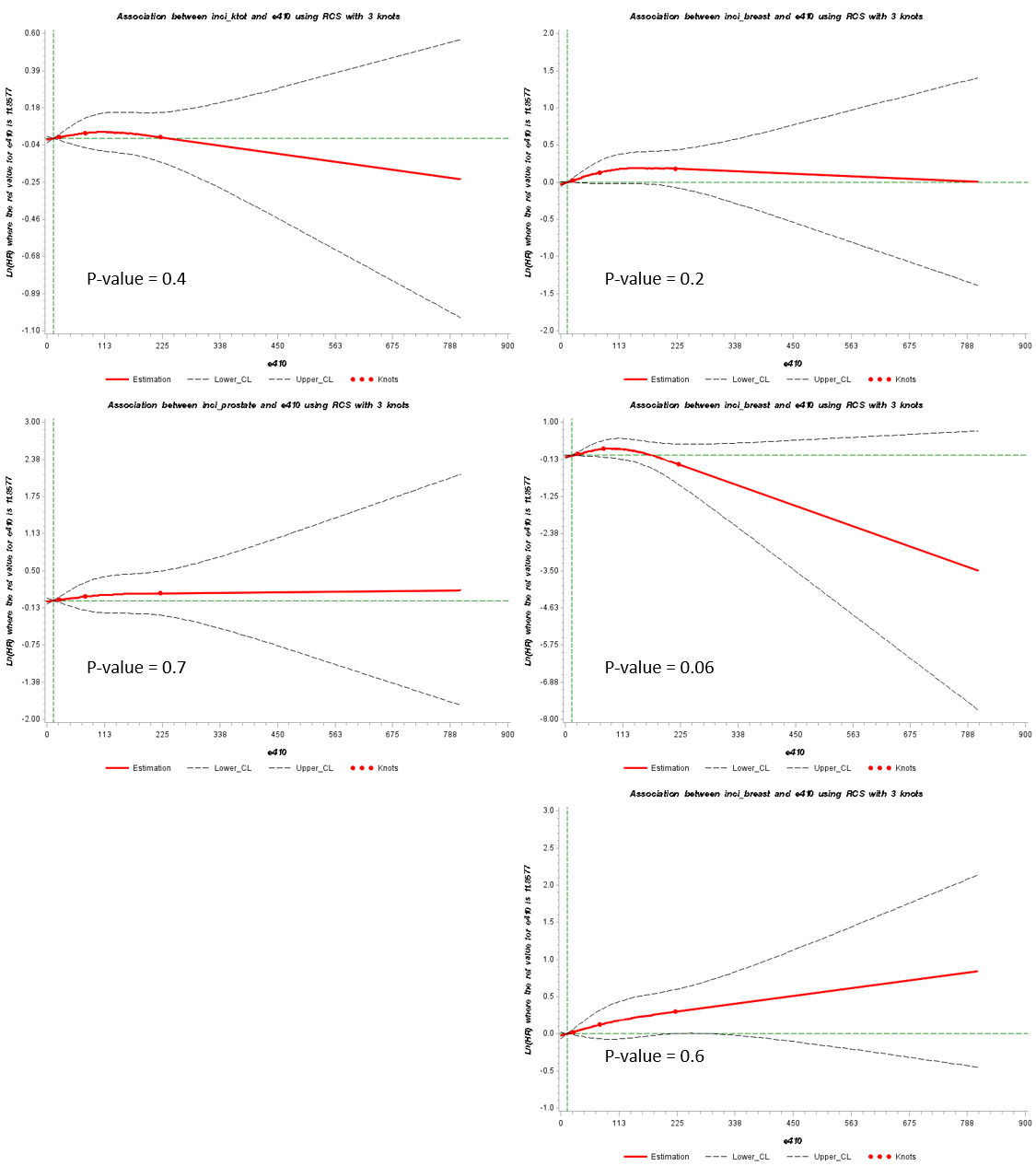
**

**H:** Associations between E412 and cancer risks.

**
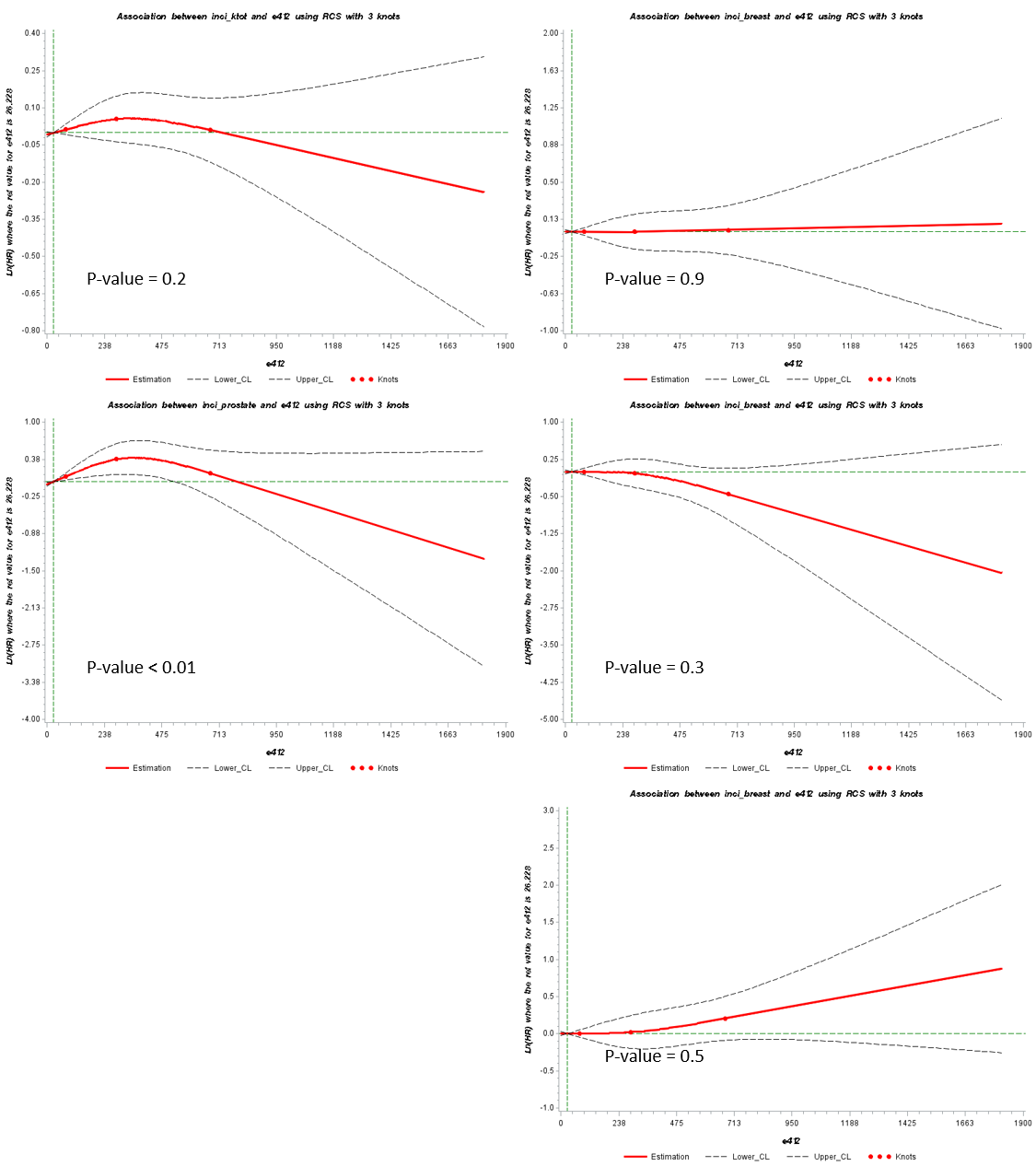
**

**I:** Associations between E414 and cancer risks.

**
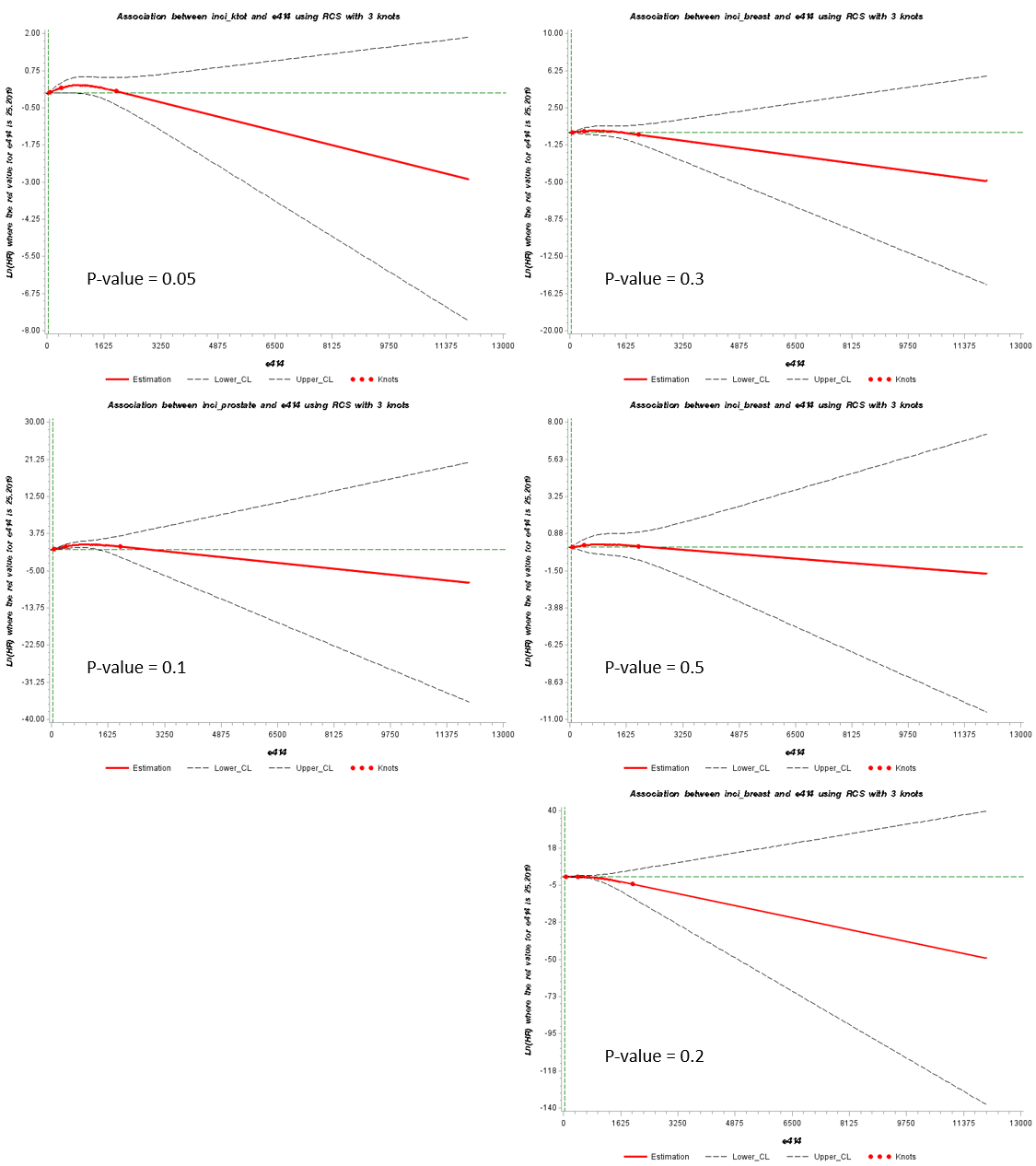
**

**J:** Associations between E415 and cancer risks.

**
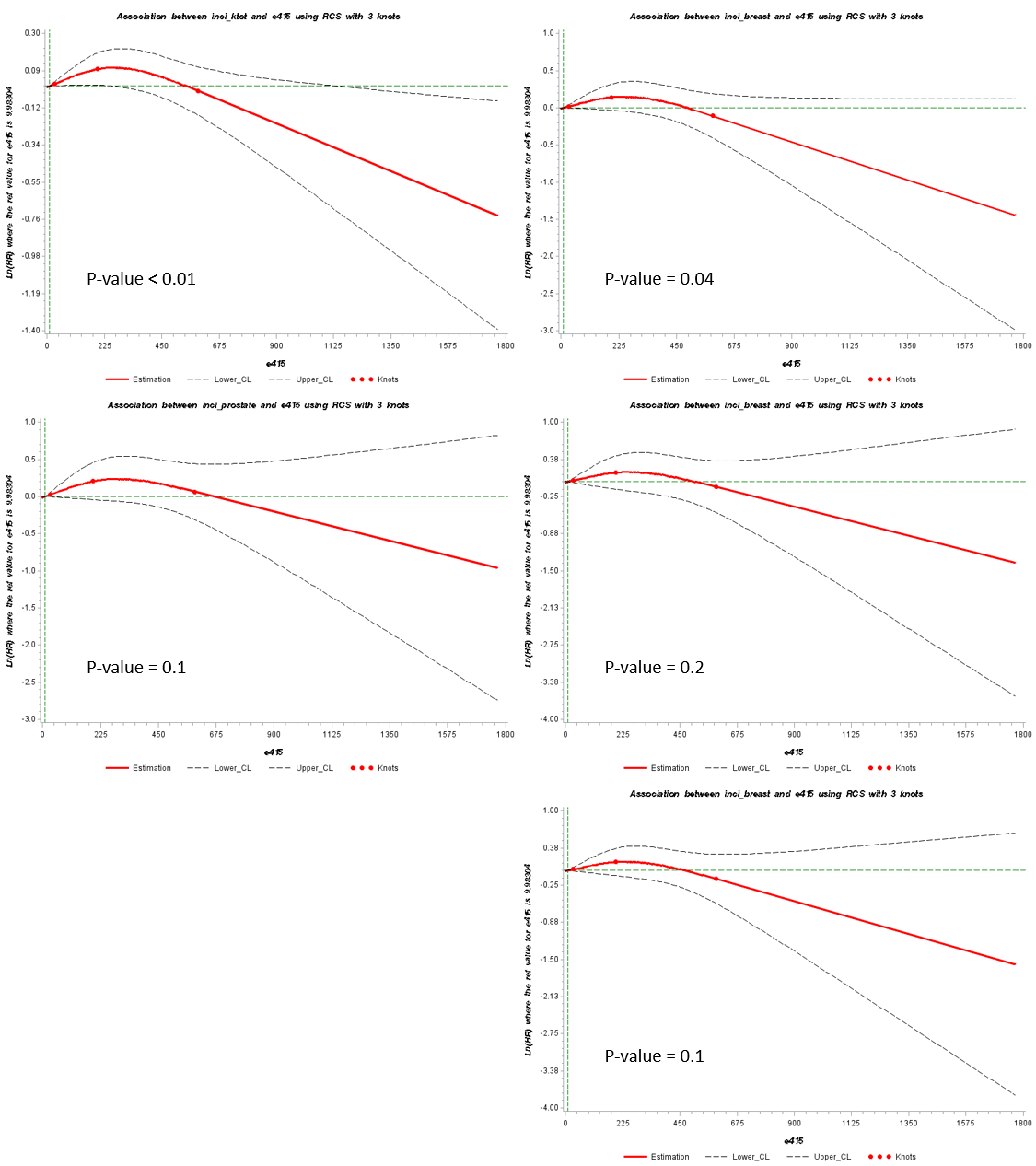
**

**K:** Associations between E440 and cancer risks.

**
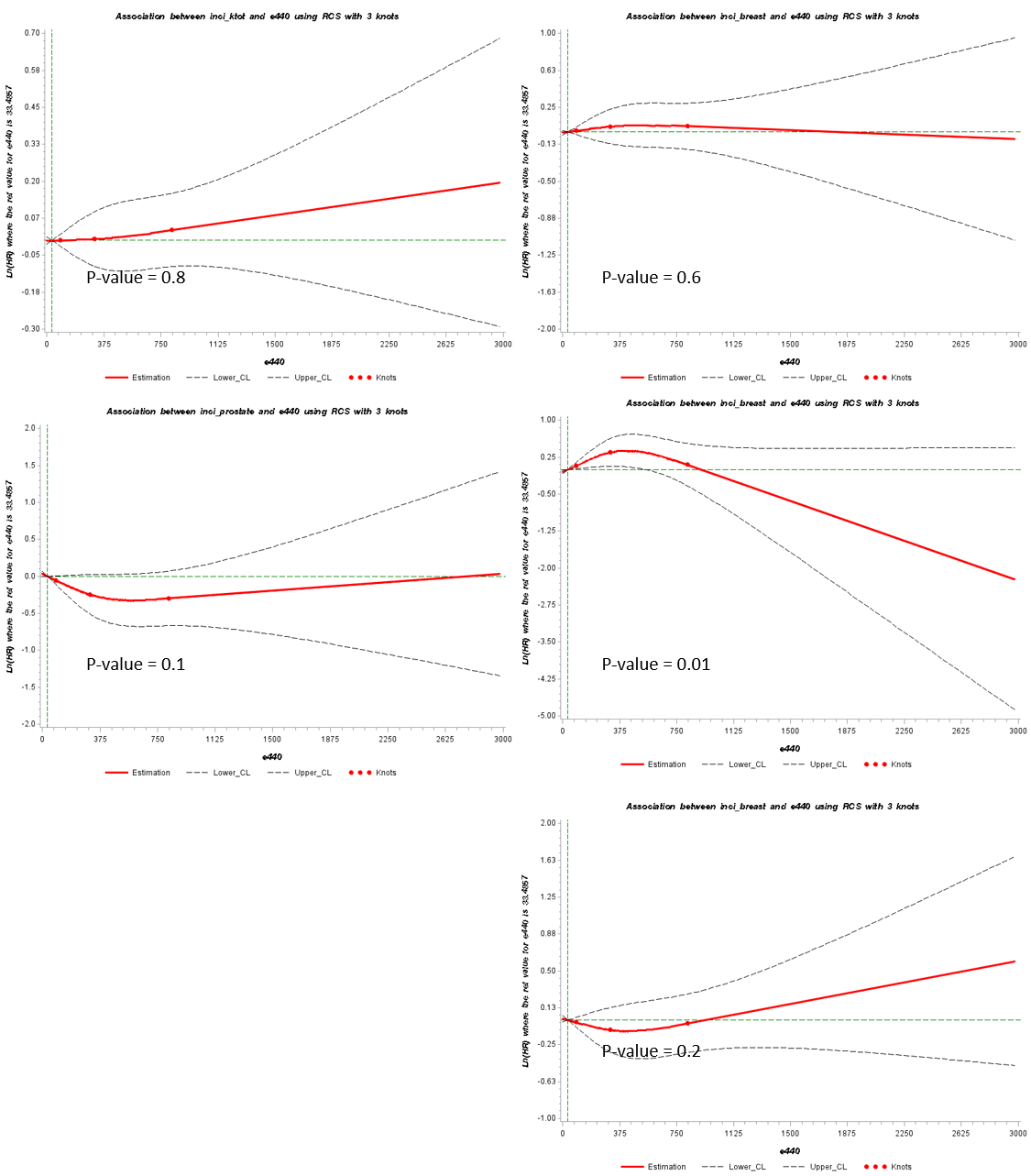
**

**L:** Associations between E500 and cancer risks.

**
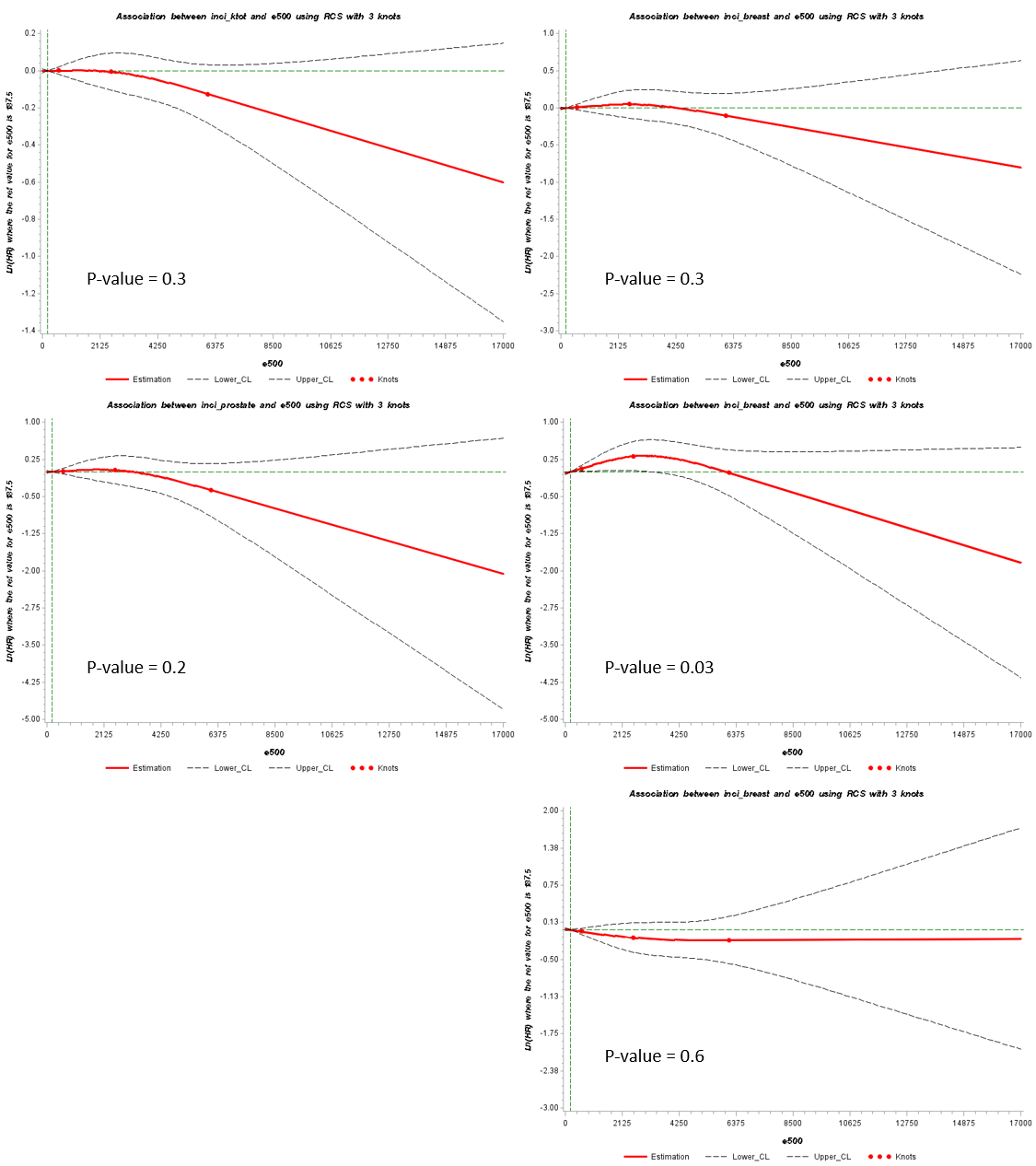
**

**M:** Associations between E901 and cancer risks.


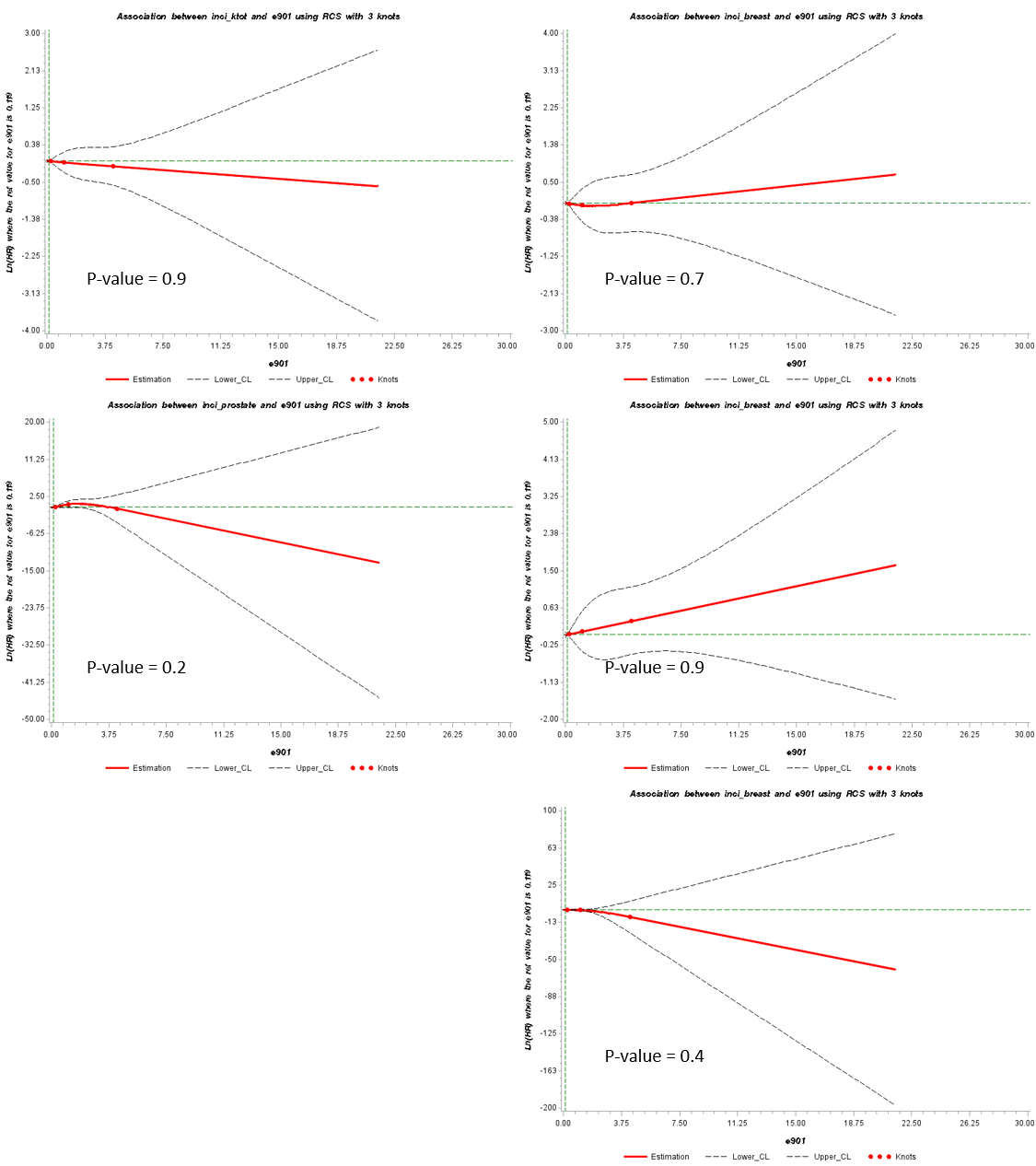


**eFigure D**. Cumulative incidence functions of the association between emulsifier intakes and risks of overall, breast and prostate cancers, respectively, in the NutriNet-Santé cohort using Fine-Gray models, 2009-2021 (n=92,000).

Total carrageenans and overall cancer risk


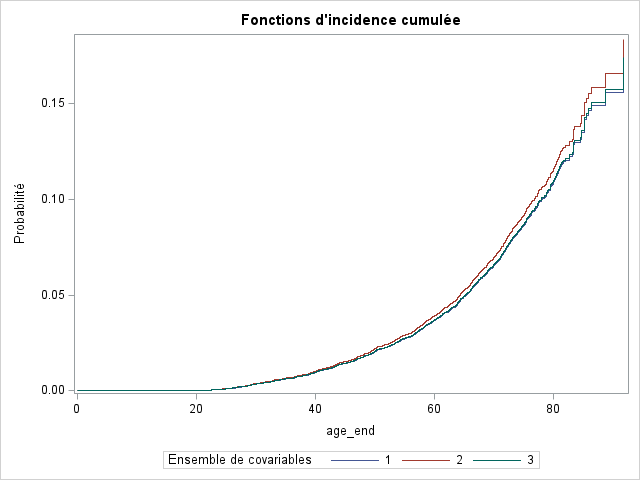


**Categories of Emulsifier intakes**

Total carrageenans and breast cancer risk


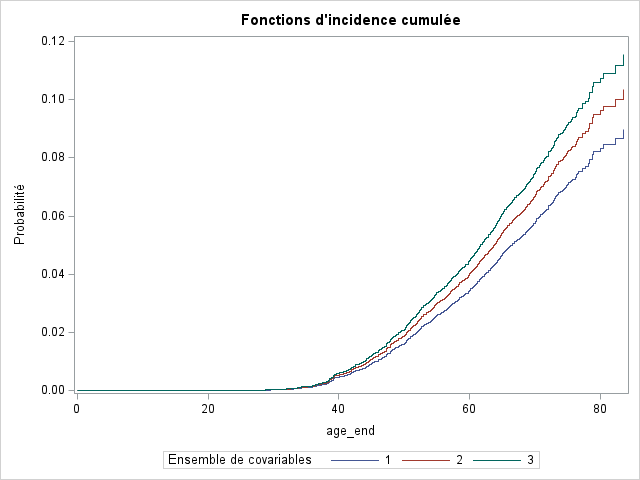


**Categories of Emulsifier intakes**

Total carrageenans and prostate cancer risk


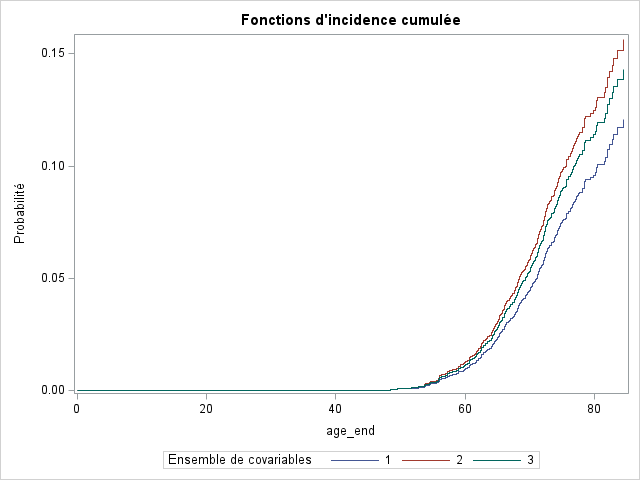


**Categories of Emulsifier intakes**

E407 and overall cancer risk


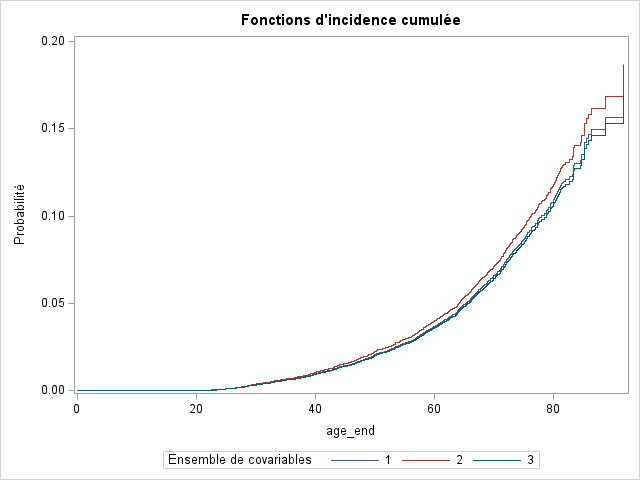


**Categories of Emulsifier intakes**

E407 and breast cancer risk


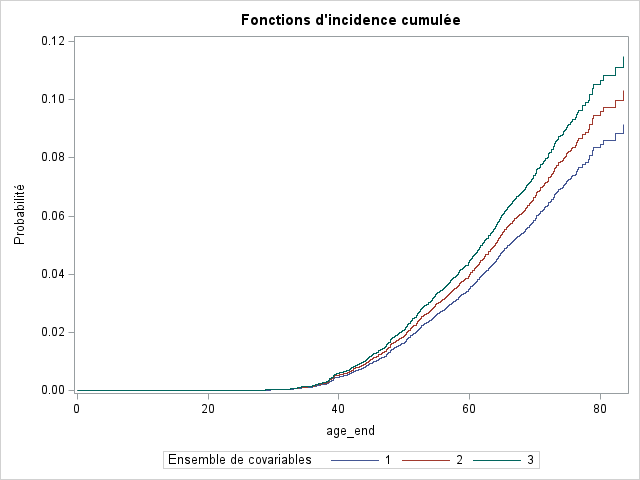


**Categories of Emulsifier intakes**

E407 and prostate cancer risk


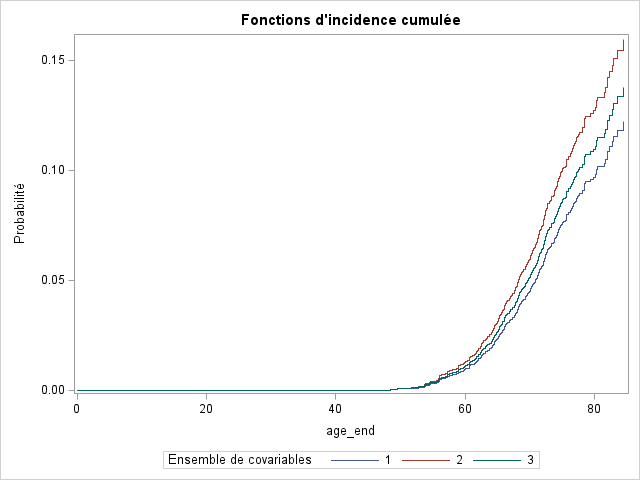


**Categories of Emulsifier intakes**

E340 and overall cancer risk


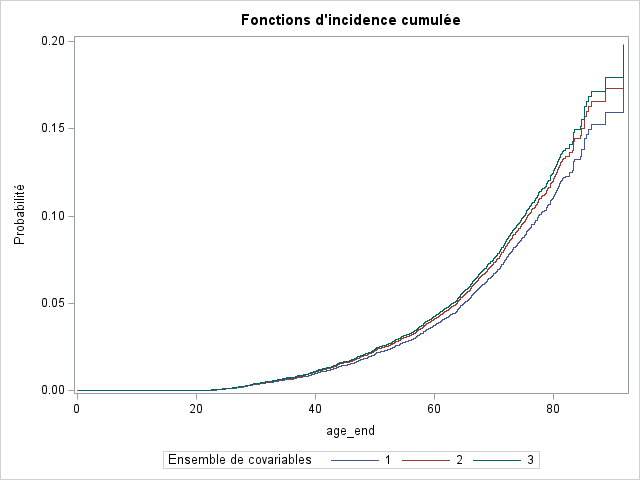


**Categories of Emulsifier intakes**

E340 and breast cancer risk


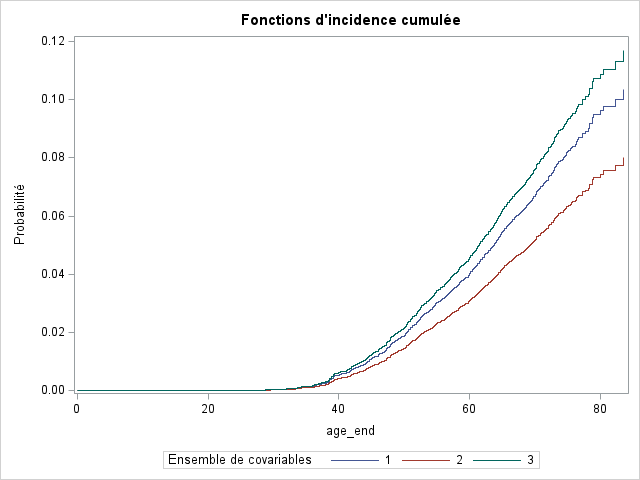


**Categories of Emulsifier intakes**

E340 and prostate cancer risk


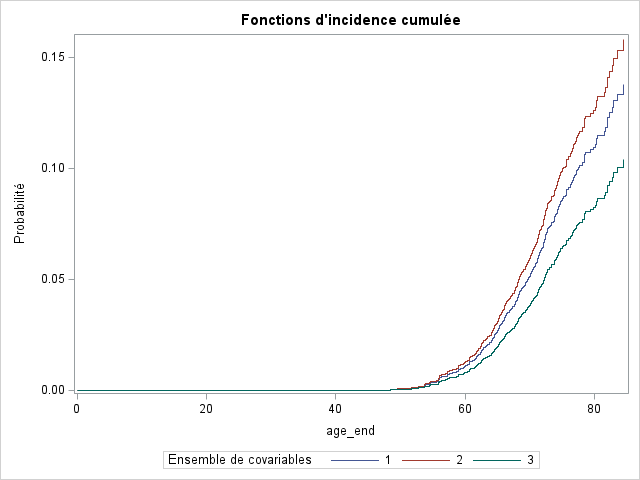


**Categories of Emulsifier intakes**

E450 and overall cancer risk


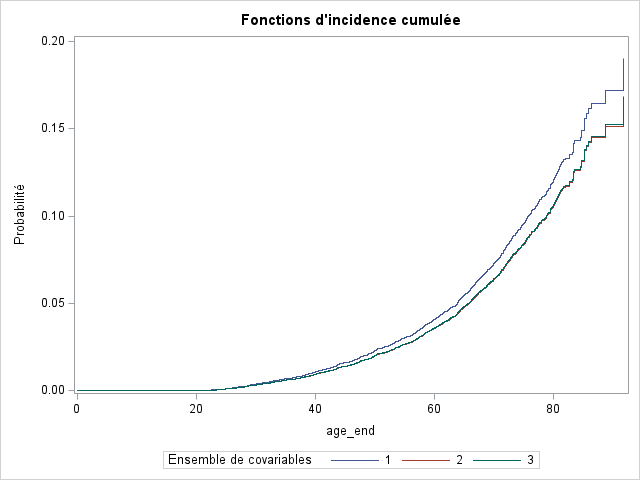


**Categories of Emulsifier intakes**

E450 and breast cancer risk


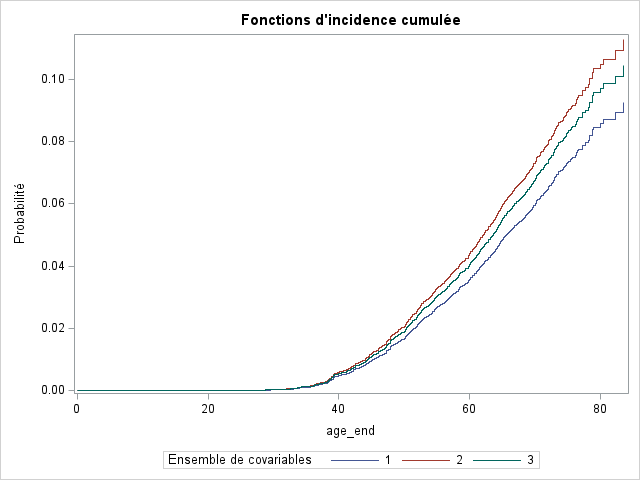


**Categories of Emulsifier intakes**

E450 and prostate cancer risk


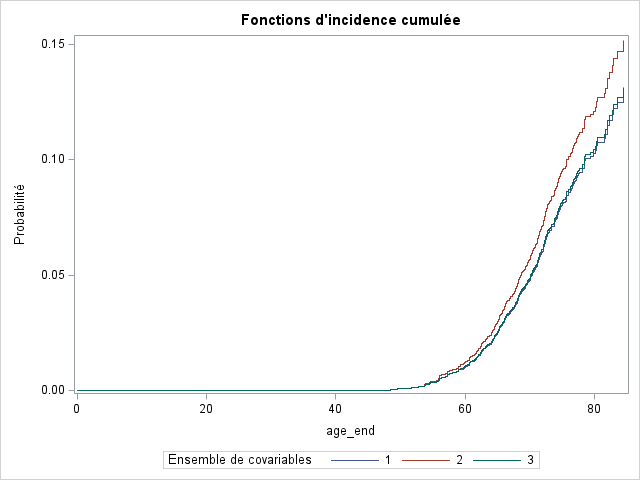


**Categories of Emulsifier intakes**

E471 and overall cancer risk


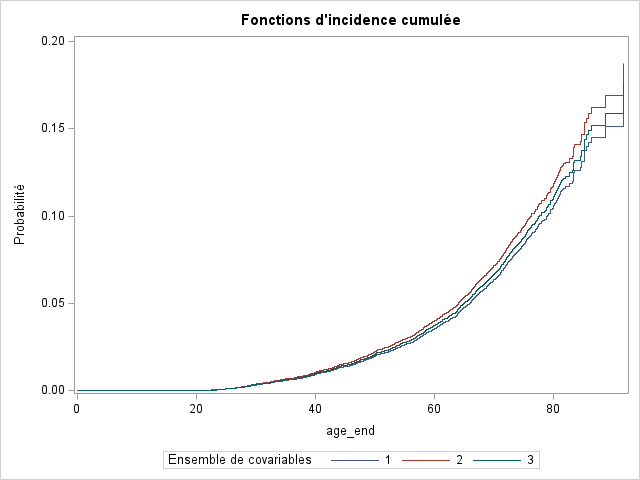


**Categories of Emulsifier intakes**

E471 and breast cancer risk


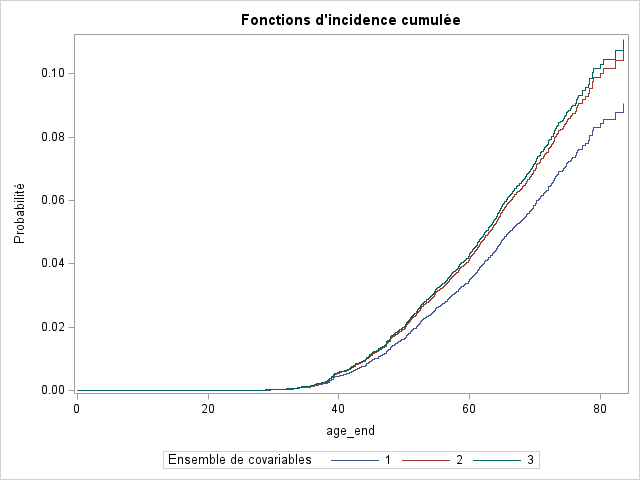


**Categories of Emulsifier intakes**

E471 and prostate cancer risk


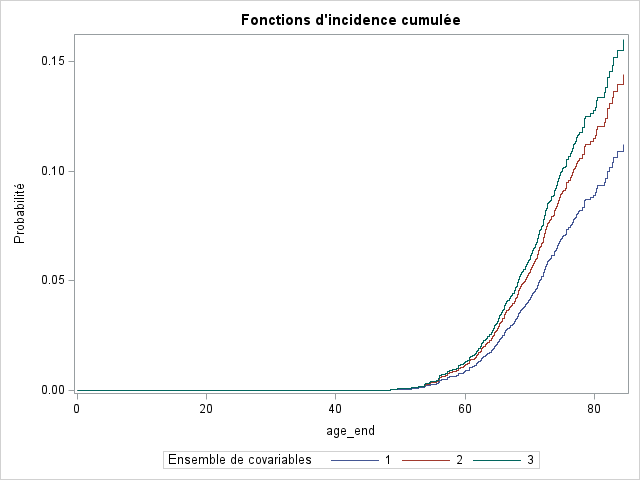


**Categories of Emulsifier intakes**

E475 and overall cancer risk


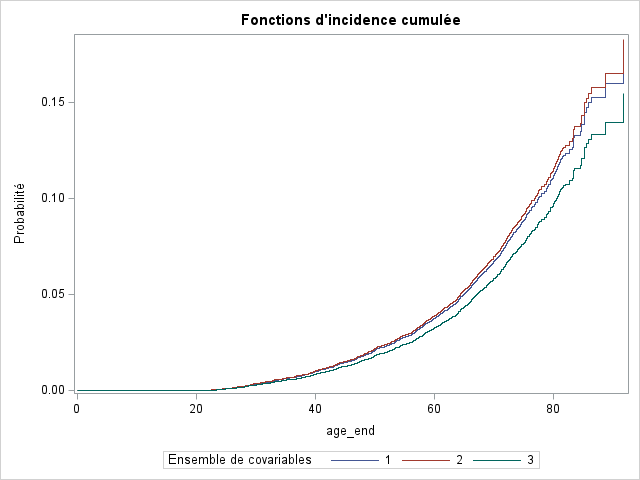


**Categories of Emulsifier intakes**

E475 and breast cancer risk


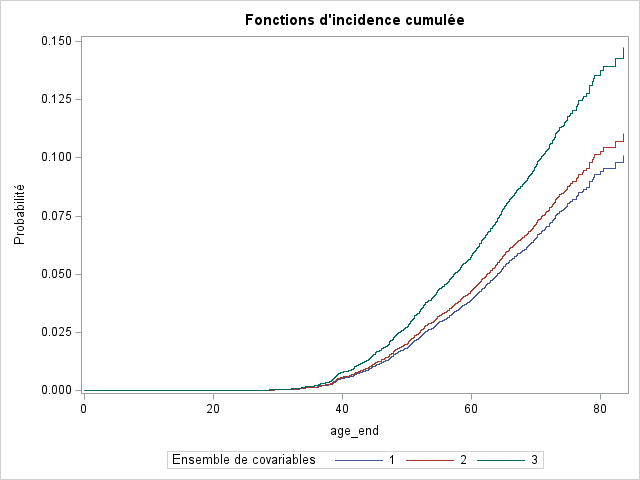


**Categories of Emulsifier intakes**

E475 and prostate cancer risk


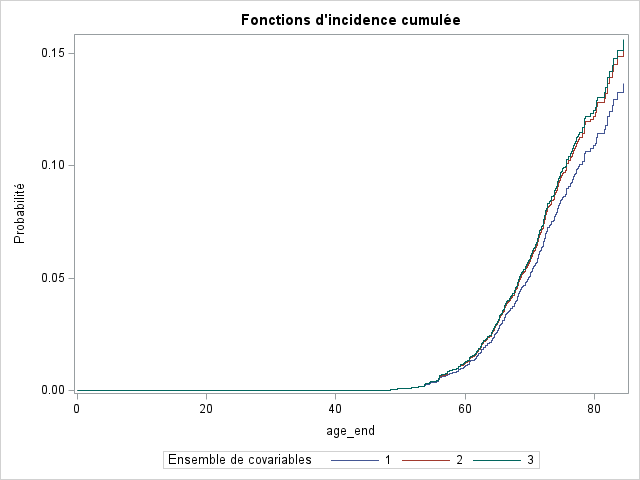


**Categories of Emulsifier intakes**

E410 and overall cancer risk


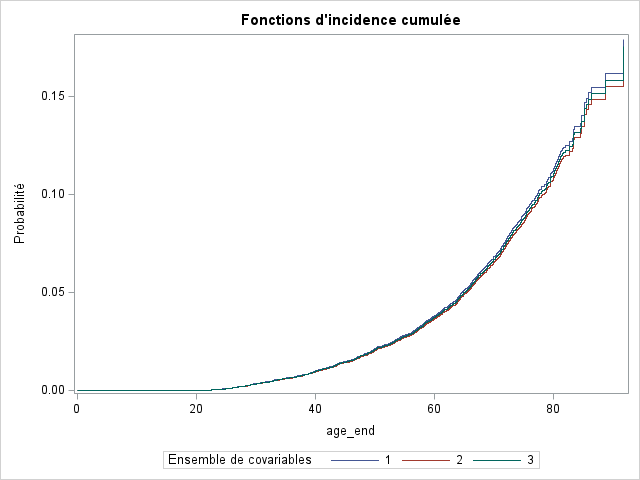


**Categories of Emulsifier intakes**

E410 and breast cancer risk


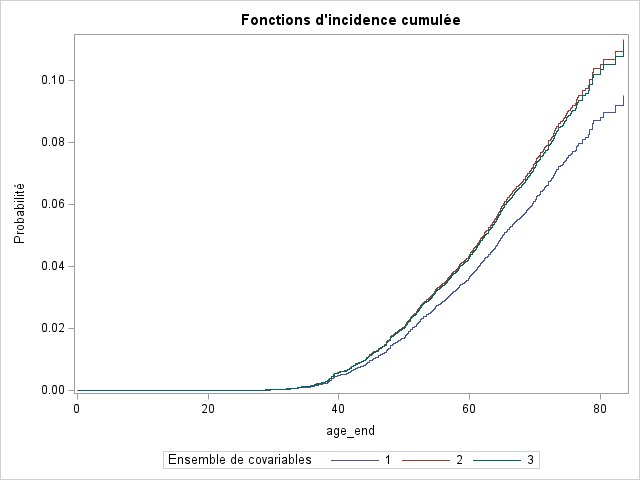


**Categories of Emulsifier intakes**

E410 and prostate cancer risk


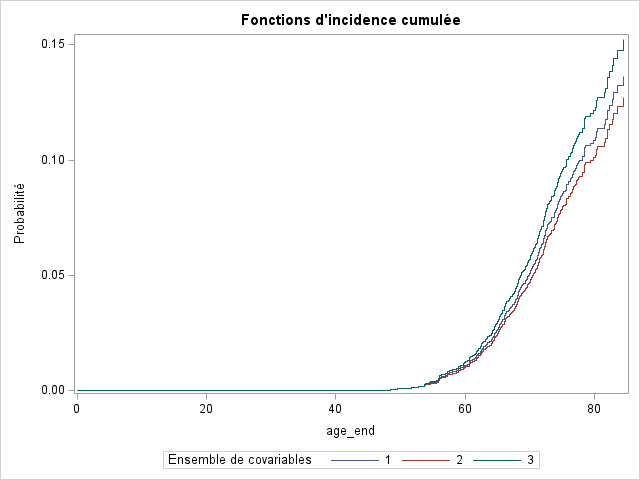


**Categories of Emulsifier intakes**

E412 and overall cancer risk


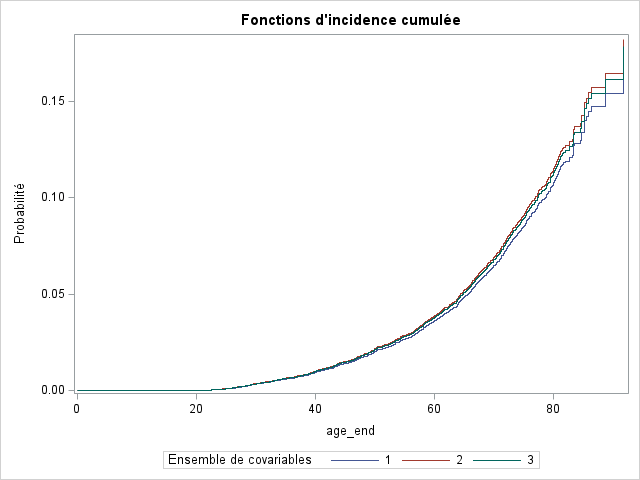


**Categories of Emulsifier intakes**

E412 and breast cancer risk


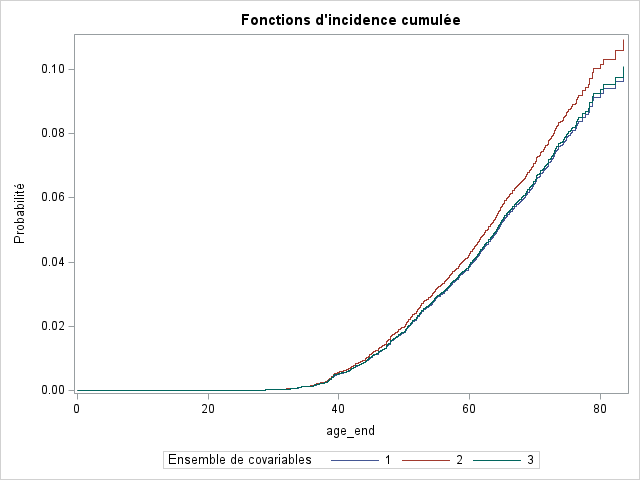


**Categories of Emulsifier intakes**

E412 and prostate cancer risk


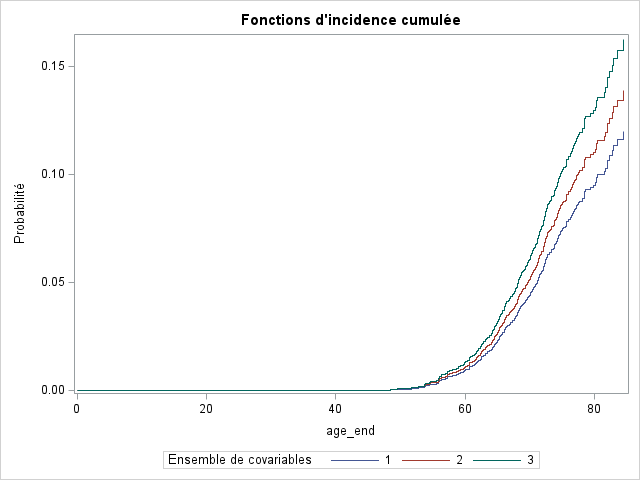


**Categories of Emulsifier intakes**

E414 and overall cancer risk


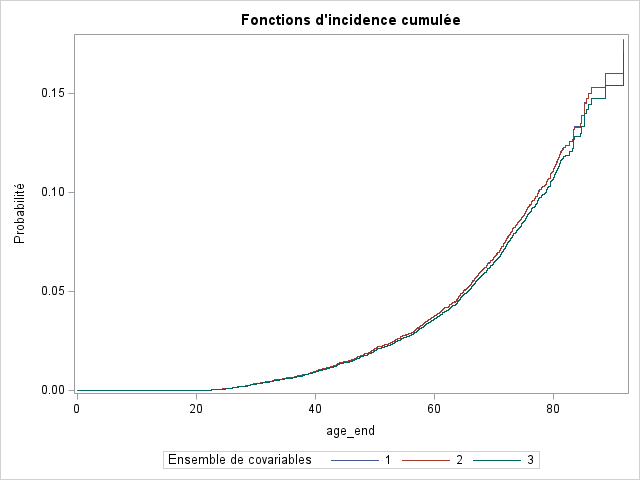


**Categories of Emulsifier intakes**

E414 and breast cancer risk


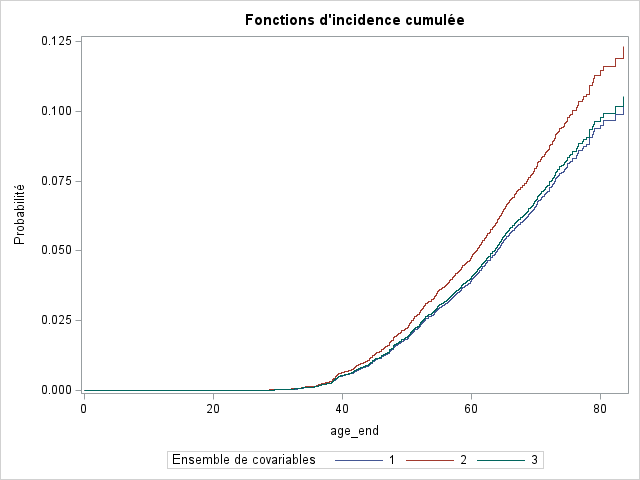


**Categories of Emulsifier intakes**

E414 and prostate cancer risk


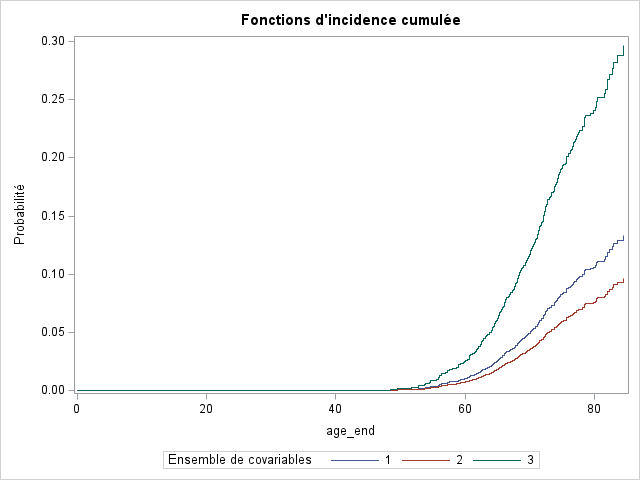


**Categories of Emulsifier intakes**

E415 and overall cancer risk


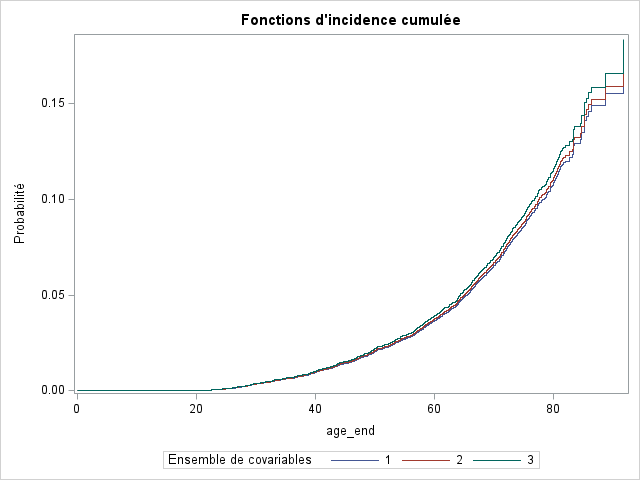


**Categories of Emulsifier intakes**

E415 and breast cancer risk


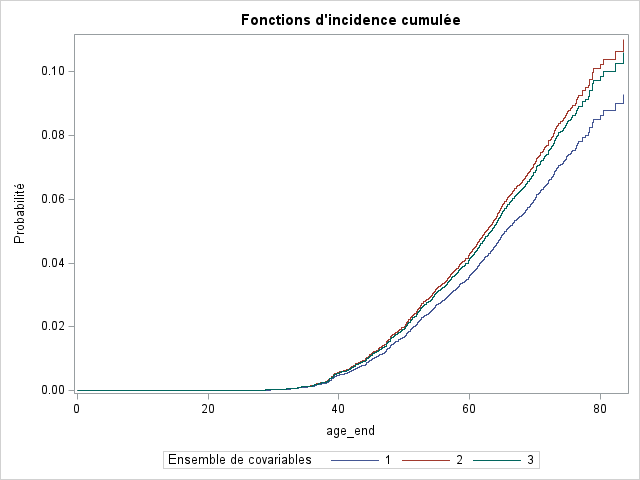


**Categories of Emulsifier intakes**

E415 and prostate cancer risk


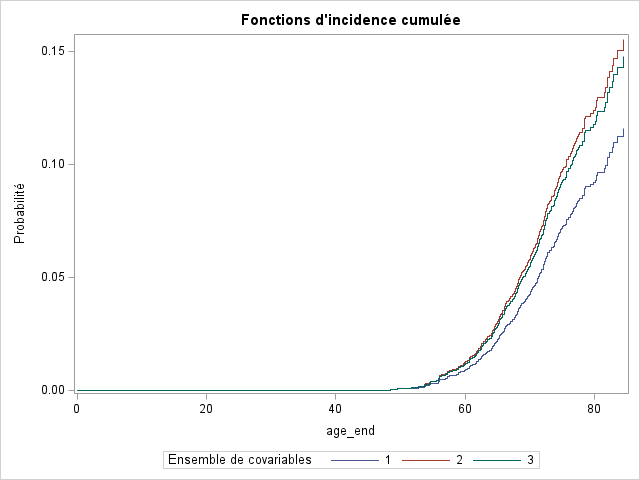


**Categories of Emulsifier intakes**

E440 and overall cancer risk


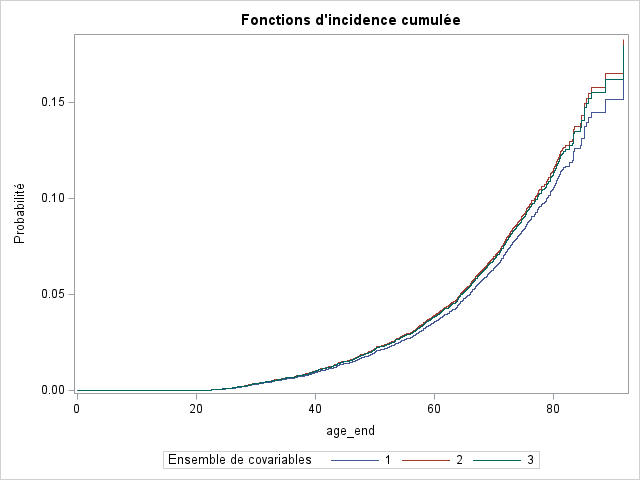


**Categories of Emulsifier intakes**

E440 and breast cancer risk


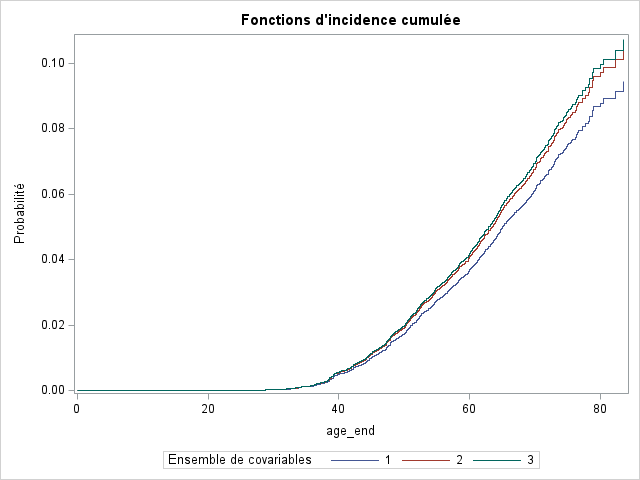


**Categories of Emulsifier intakes**

E440 and prostate cancer risk


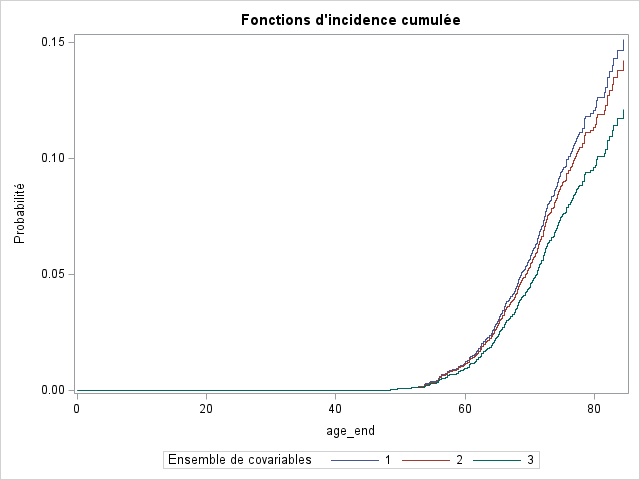


**Categories of Emulsifier intakes**

E500 and overall cancer risk


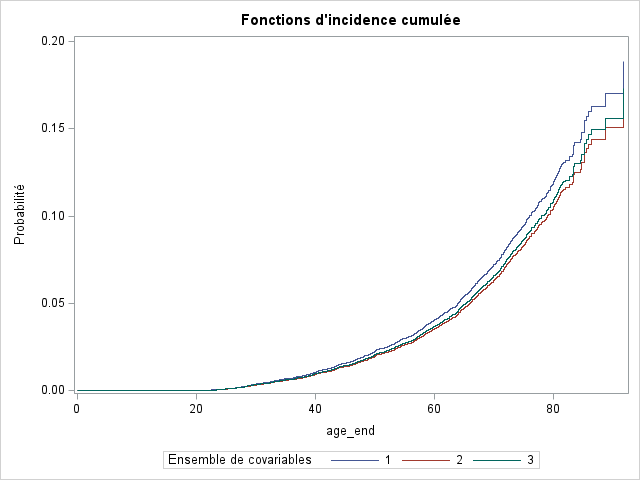


**Categories of Emulsifier intakes**

E500 and breast cancer risk


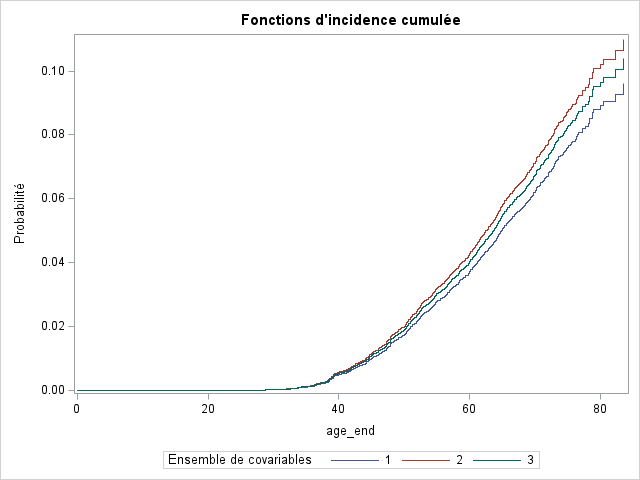


**Categories of Emulsifier intakes**

E500 and prostate cancer risk


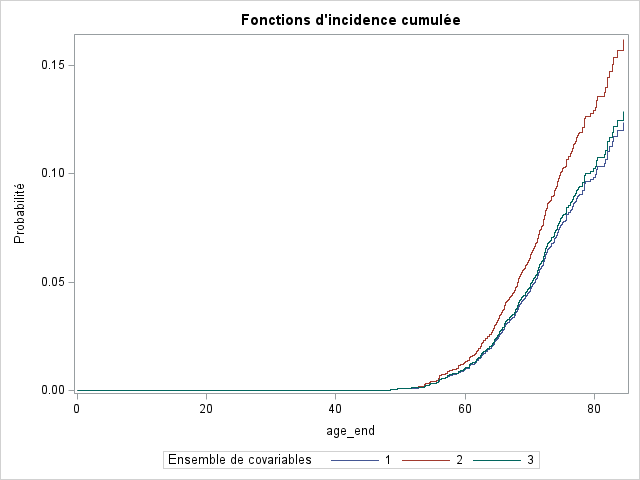


**Categories of Emulsifier intakes**

E901 and overall cancer risk


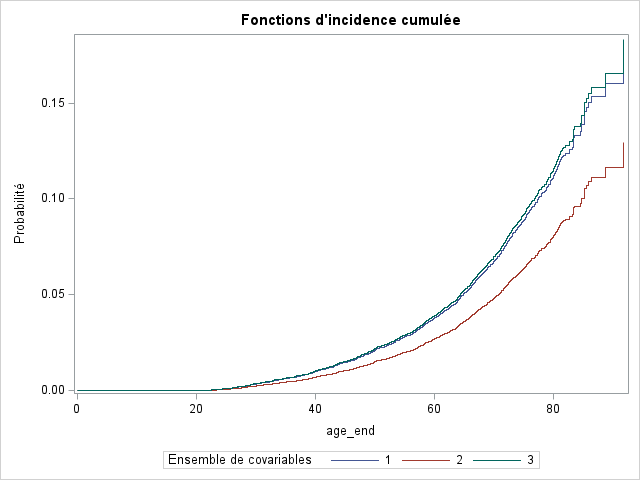


**Categories of Emulsifier intakes**

E901 and breast cancer risk


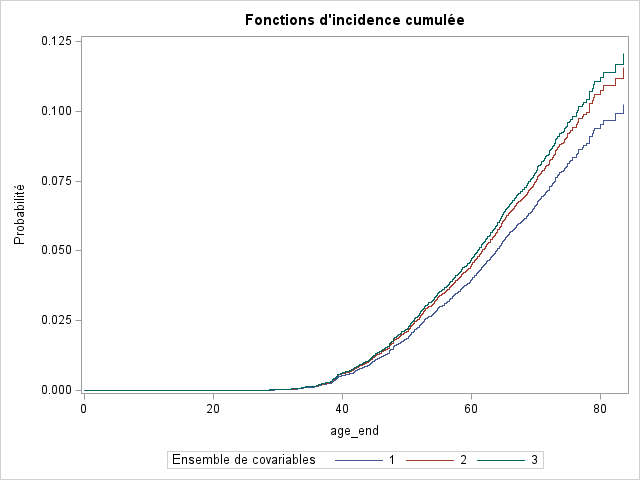


**Categories of Emulsifier intakes**

E901 and prostate cancer risk


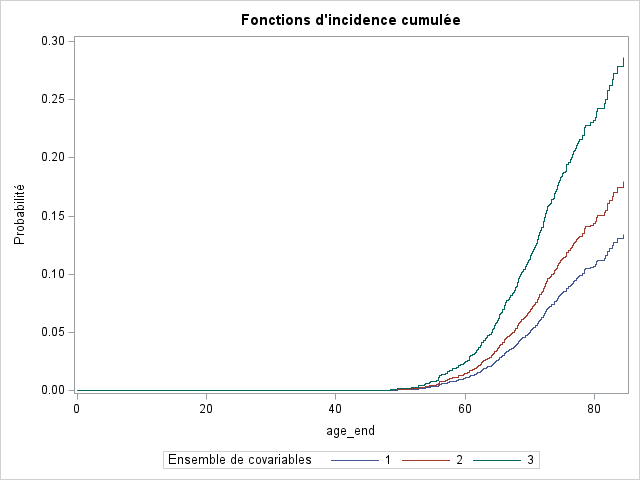


**Categories of Emulsifier intakes**

# **eResults**

**eTable A.** Detailed contribution of 24 food groups to emulsifier intakes among participants from the NutriNet-Santé cohort, 2009-2021 (n=92,000). ^a^

| **Food groups** | **Total emulsifiers** | **Total modified starches** | **Total phosphates** | **Total lactylates** | **Total polyglycerol esters of FAs** | **Total mono- and diglycerides of FAs** | **Total celluloses** | **Total carrageenans** | | **Total alginates** |
| --- | --- | --- | --- | --- | --- | --- | --- | --- | --- | --- |
| Food group contribution (%) | | | | | | | | | | |
| **Processed fruits and vegetables** | 15.0 | 27.9 | 3.7 | 1.3 | 0 | 2.1 | 11.3 | 1.7 | 1.4 | |
| **Processed potatoes and tubers** | 3.4 | 5.4 | 0.9 | 0 | 0 | 3.1 | 20.2 | 0.1 | <0.1 | |
| **Refined grains and cereals** | 5.2 | 5.5 | 7.0 | 14.0 | 0 | 7.5 | 5.2 | 1.6 | 0.8 | |
| **Whole grains and cereals** | 0.3 | 0.1 | <0.1 | 5.7 | 0 | 4.7 | 0.1 | <0.1 | 0 | |
| **Processed pulses and legumes** | 0.4 | 0.4 | 0.6 | 0 | 0 | <0.1 | 1.3 | <0.1 | 0.7 | |
| **Nuts. seeds and dried fruits** | 0.1 | <0.1 | <0.1 | 0 | 0 | <0.1 | 0 | <0.1 | 0 | |
| **Dairy products** | 7.6 | 9.0 | 7.3 | 0.2 | 0 | 3.1 | 0.5 | 17.1 | 2.3 | |
| **Dairy desserts** | 5.4 | 8.0 | 0.8 | 0 | 0 | 2.6 | 0.5 | 30.6 | 17.5 | |
| **Processed red meat and offals** | 1.5 | 2.4 | 1.6 | 0.4 | 0 | 0.1 | 0.2 | <0.1 | 0.7 | |
| **Processed poultry** | 0.9 | 1.4 | 1.5 | 0.1 | 0 | 0.2 | 1.0 | 0.4 | 0.7 | |
| **Other processed meat** | 1.5 | 1.6 | 4.3 | 0.3 | 0 | 2.1 | 0.2 | 1.2 | 1.7 | |
| **Processed eggs** | 2.0 | 0.8 | 3.2 | <0.1 | 0 | 0.7 | 0.1 | 0.1 | 0.8 | |
| **Processed fish and seafood** | 1.6 | 1.5 | 0.8 | <0.1 | 0 | 0.2 | <0.1 | 1.5 | 0.1 | |
| **Fats and sauces** | 7.3 | 11.0 | 2.5 | 0.4 | 10.8 | 22.2 | 10.9 | 11.3 | 5.7 | |
| **Broths** | 5.8 | 13.6 | 0.8 | 0 | 0 | 0.1 | 0 | 0.1 | 0 | |
| **Breakfast cereals** | 0.3 | <0.1 | 1.0 | 0 | 0.6 | 1.3 | 0.4 | <0.1 | 0.2 | |
| **Confectionery** | 5.4 | 1.0 | <0.1 | <0.1 | 9.2 | 10.6 | 0.4 | 13.7 | 4.1 | |
| **Cakes and biscuits** | 28.7 | 6.6 | 57.0 | 2.4 | 18.9 | 22.0 | 43.3 | 14.0 | 50.9 | |
| **Pastries** | 1.2 | 0.2 | 0.4 | 74.9 | 60.4 | 14.5 | 3.0 | 2.0 | 0.1 | |
| **Unsweetened soft drinks** | 1.5 | 0.4 | 2.6 | 0 | 0 | <0.1 | 0.9 | 2.6 | <0.1 | |
| **Sweetened soft drinks** | 0.6 | <0.1 | 0.5 | 0 | 0 | 0.1 | <0.1 | 0.5 | 0 | |
| **Alcoholic drinks** | <0.1 | 0 | 0 | 0 | 0 | 0 | 0 | 0 | <0.1 | |
| **Meal replacements** | 0.4 | 0.9 | <0.1 | 0 | 0 | <0.1 | 0 | 0.6 | 0 | |
| **Others** | 3.8 | 2.5 | 3.4 | 0.3 | 0 | 2.8 | 0.5 | 0.9 | 12.1 | |

**Abbreviations**: FAs: fatty acids.

a Groups of emulsifiers were defined as follows (European codes): total phosphates (E339, E340, E341, E343, E450, E451, E452), total lactylates (E481, E482), total polyglycerol esters of FAs (E475, E476), total mono and diglycerides of FAs (E471, E472, E472a, E472b, E472c, E472e), total celluloses (E460, E461, E464, E466, E468), total carrageenans (E407, E407a), total alginates (E400, E401, E402, E404, E405), and total modified starches (E14xx).

**eTable B.** Mean daily emulsifier intakes in mg/d (SD) among study participants from the NutriNet-Santé cohort, 2009-2021 (*N*=92,000).

|  | **Categories of emulsifier intakes ^b^** | | |
| --- | --- | --- | --- |
|  | **1 (low /none intake)** | **2 (medium / low)** | **3 (high intake)** |

| **Total emulsifiers** | **All** | 1524.63 (720.5) | 3687.1 (644.98) | 7613.98 (2909.7) |
| --- | --- | --- | --- | --- |
|  | **Women** | 1526.01 (712.65) | 3640.45 (621.36) | 7452.37 (2792.75) |
|  | **Men** | 1519.59 (748.58) | 3857.98 (698.89) | 8205.91 (3234.86) |
| **Total alginates** | **All** | 0 (0) | 17.14 (10.37) | 95.01 (79.19) |
|  | **Women** | 0 (0) | 16.77 (10.22) | 91.55 (72.96) |
|  | **Men** | 0 (0) | 18.68 (10.83) | 109.38 (99.78) |
| **Sodium alginate E401** | **All** | 0 (0) | 17.68 (10.83) | 97.37 (79.88) |
|  | **Women** | 0 (0) | 17.4 (10.73) | 94.03 (73.75) |
|  | **Men** | 0 (0) | 18.91 (11.15) | 111 (99.99) |
| **Total carrageenans** | **All** | 1.92 (3.48) | 39.47 (15.68) | 138.99 (77.2) |
|  | **Women** | 2.04 (3.59) | 38.92 (15.11) | 134.18 (72.16) |
|  | **Men** | 1.48 (3) | 41.47 (17.47) | 156.61 (91.22) |
| **Carrageenan E407** | **All** | 1.6 (3.01) | 37.45 (15.32) | 133.82 (75.26) |
|  | **Women** | 1.73 (3.13) | 36.98 (14.76) | 129.09 (70.4) |
|  | **Men** | 1.15 (2.46) | 39.2 (17.12) | 151.11 (88.72) |
| **Processed Euchema Seaweed E407a** | **All** | 0 (0) | 5.61 (6.83) | 52.38 (40.6) |
|  | **Women** | 0 (0) | 6.27 (7.28) | 52.59 (36.84) |
|  | **Men** | 0 (0) | 3.27 (4.11) | 51.74 (50.45) |
| **Total phosphates** | **All** | 22.02 (33.12) | 234.1 (79.94) | 824.83 (611.22) |
|  | **Women** | 23.48 (34.09) | 232.82 (77.57) | 805.33 (549.14) |
|  | **Men** | 16.67 (28.7) | 238.78 (87.93) | 896.25 (794.38) |
| **Trisodium phosphate E339** | **All** | 0 (0) | 45.4 (27.79) | 257.9 (201.5) |
|  | **Women** | 0 (0) | 45.88 (27.71) | 259.06 (195.81) |
|  | **Men** | 0 (0) | 43.26 (28.05) | 252.45 (226.42) |
| **Tripotassium phosphate E340** | **All** | 0 (0) | 16.28 (15.59) | 272.21 (481.76) |
|  | **Women** | 0 (0) | 16.31 (15.9) | 279.34 (490.04) |
|  | **Men** | 0 (0) | 16.11 (13.85) | 235.66 (435.58) |
| **Tricalcium phosphate E341** | **All** | 0 (0) | 25.39 (15.71) | 285.32 (729.05) |
|  | **Women** | 0 (0) | 24.91 (15.12) | 271.17 (571.94) |
|  | **Men** | 0 (0) | 27.51 (17.96) | 349.04 (1203.49) |
| **Diphosphates E450** | **All** | 4.1 (10.6) | 144.42 (61.64) | 591.7 (395.85) |
|  | **Women** | 4.85 (11.59) | 145.42 (60.14) | 580.06 (387.73) |
|  | **Men** | 1.37 (4.81) | 140.75 (66.71) | 634.31 (421.59) |
| **Triphosphates E451** | **All** | 0 (0) | 62.8 (33.87) | 285.14 (189.05) |
|  | **Women** | 0 (0) | 59.6 (31.27) | 268.46 (173.4) |
|  | **Men** | 0 (0) | 72.69 (39.26) | 340.78 (225.09) |
| **Polyphosphates E452** | **All** | 0 (0) | 20.31 (14.99) | 194.97 (157.18) |
|  | **Women** | 0 (0) | 18.99 (13.59) | 188.45 (150.87) |
|  | **Men** | 0 (0) | 25.4 (18.6) | 220 (177.17) |
| **Total celluloses** | **All** | 0 (0) | 6.84 (7.81) | 174.8 (234.63) |
|  | **Women** | 0 (0) | 6.52 (7.31) | 168.01 (227.56) |
|  | **Men** | 0 (0) | 8.49 (9.78) | 209.1 (264.92) |
| **Cellulose E460** | **All** | 0 (0) | 4.24 (4.34) | 187.63 (245.57) |
|  | **Women** | 0 (0) | 4.06 (4.05) | 174.86 (232.02) |
|  | **Men** | 0 (0) | 5.16 (5.56) | 255.82 (299.12) |
| **Hydroxypropylmethyl cellulose E464** | **All** | 0 (0) | 2.1 (5.84) | 148.33 (159.9) |
|  | **Women** | 0 (0) | 1.92 (5.41) | 147.13 (151.62) |
|  | **Men** | 0 (0) | 2.92 (7.47) | 153.79 (193.28) |
| **Carboxymethyl cellulose E466** | **All** | 0 (0) | 6.06 (4.18) | 66.33 (113.62) |
|  | **Women** | 0 (0) | 5.72 (3.85) | 64.99 (116.92) |
|  | **Men** | 0 (0) | 8.02 (5.32) | 73.79 (92.9) |
| **Total mono- and diglycerides of FAs** | **All** | 12.18 (16.58) | 127.59 (46.47) | 477.03 (323.79) |
|  | **Women** | 13.02 (17.14) | 127.54 (45.45) | 468.42 (310.22) |
|  | **Men** | 9.1 (13.91) | 127.75 (50.01) | 508.57 (367.57) |
| **Mono- and diglycerides of FAs E471** | **All** | 7.16 (11.15) | 104.17 (40.48) | 376.1 (219.03) |
|  | **Women** | 7.69 (11.63) | 104.16 (39.57) | 368.93 (212.44) |
|  | **Men** | 5.21 (8.91) | 104.23 (43.66) | 402.26 (239.8) |
| **Lactic acid esters of mono- and diglycerides of FAs E472b** | **All** | 0 (0) | 27.19 (15.81) | 287.07 (274.13) |
|  | **Women** | 0 (0) | 26.34 (15.06) | 275.14 (257.54) |
|  | **Men** | 0 (0) | 30.63 (18.15) | 337.16 (330.42) |
| **Citric acid esters of mono- and diglycerides of FAs E472c** | **All** | 0 (0) | 23.84 (13.28) | 201.36 (195.19) |
|  | **Women** | 0 (0) | 22.14 (11.88) | 192.82 (181.53) |
|  | **Men** | 0 (0) | 30.2 (16.03) | 233.72 (237.35) |
| **Acetyl tartaric acid esters of mono- and diglycerides of FAs E472e** | **All** | 0 (0) | 10.65 (5.03) | 60.29 (83.1) |
|  | **Women** | 0 (0) | 10.69 (5) | 59.62 (84.18) |
|  | **Men** | 0 (0) | 10.48 (5.16) | 63.46 (77.72) |
| **Total polyglycerol esters of FAs** | **All** | 0 (0) | 10.23 (7.45) | 122.79 (151.71) |
|  | **Women** | 0 (0) | 9.81 (7.16) | 122.59 (151.8) |
|  | **Men** | 0 (0) | 11.98 (8.28) | 123.63 (151.36) |
| **Polyglycerol esters of FAs E475** | **All** | 0 (0) | 52.63 (22.52) | 253.03 (204.05) |
|  | **Women** | 0 (0) | 52.75 (22.62) | 251.85 (203.08) |
|  | **Men** | 0 (0) | 52.12 (22.09) | 258.38 (208.51) |
| **Polyglycerol esters of interesterified ricinoleic acid E476** | **All** | 0 (0) | 5.97 (4.12) | 40.62 (38.14) |
|  | **Women** | 0 (0) | 5.64 (3.87) | 38.81 (34.54) |
|  | **Men** | 0 (0) | 7.3 (4.78) | 47.79 (49.31) |
| **Total lactylates** | **All** | 0 (0) | 14.31 (8.11) | 83.45 (67.14) |
|  | **Women** | 0 (0) | 13.72 (7.68) | 80.8 (65.7) |
|  | **Men** | 0 (0) | 16.87 (9.34) | 95.61 (72.16) |
| **Sodium stearoyl-2-lactylate E481** | **All** | 0 (0) | 14.24 (8.09) | 82.71 (66.81) |
|  | **Women** | 0 (0) | 13.63 (7.64) | 79.94 (65.22) |
|  | **Men** | 0 (0) | 16.89 (9.36) | 95.36 (72.35) |
| **Total modified starches** | **All** | 295.84 (226.98) | 1070.26 (236.72) | 2535.21 (1024.54) |
|  | **Women** | 295.15 (224.17) | 1054.06 (229.01) | 2491.57 (1001.67) |
|  | **Men** | 298.35 (236.96) | 1129.71 (254.41) | 2694.76 (1089.44) |
| **Lecithins E322** | **All** | 5.86 (6.44) | 39.18 (12.78) | 138.33 (88.81) |
|  | **Women** | 6.35 (6.67) | 39.9 (12.64) | 137.18 (86.31) |
|  | **Men** | 4.08 (5.16) | 36.54 (12.98) | 142.54 (97.33) |
| **Sodium citrate E331** | **All** | 0 (0) | 61.67 (35.72) | 413.39 (418.89) |
|  | **Women** | 0 (0) | 61.26 (35.76) | 411.69 (411.26) |
|  | **Men** | 0 (0) | 63.32 (35.54) | 420.13 (447.98) |
| **Carob bean gum E410** | **All** | 0 (0) | 22.54 (11.03) | 117.12 (101.72) |
|  | **Women** | 0 (0) | 22.12 (10.78) | 112.95 (97.13) |
|  | **Men** | 0 (0) | 24.23 (11.83) | 132.79 (116.08) |
| **Guar gum E412** | **All** | 2.66 (6.15) | 94.67 (47.36) | 404.5 (245.16) |
|  | **Women** | 2.43 (5.64) | 89.29 (44.02) | 387.28 (232.41) |
|  | **Men** | 3.52 (7.67) | 114.34 (53.53) | 467.58 (278.11) |
| **Gum arabic E414** | **All** | 0 (0) | 68.48 (46.34) | 931.41 (1518.29) |
|  | **Women** | 0 (0) | 66.75 (44.7) | 906.92 (1500.46) |
|  | **Men** | 0 (0) | 78.15 (53.58) | 1063.93 (1605.97) |
| **Gum Xanthan Gum E415** | **All** | 4.96 (6.59) | 56.9 (28.28) | 343.15 (263.3) |
|  | **Women** | 4.53 (5.98) | 52.84 (24.98) | 324.16 (248.4) |
|  | **Men** | 6.53 (8.28) | 71.75 (34.11) | 412.7 (301.85) |
| **Pectins E440** | **All** | 15.15 (19.5) | 133.59 (46.04) | 509.36 (379.01) |
|  | **Women** | 14.86 (18.81) | 130.59 (45.17) | 504.77 (377.7) |
|  | **Men** | 16.21 (21.81) | 144.75 (47.49) | 525.94 (383.26) |
| **Ammonium salts of phosphatidic acid E442** | **All** | 0 (0) | 3.17 (1.97) | 114.28 (147.58) |
|  | **Women** | 0 (0) | 3.03 (1.81) | 109.84 (146.68) |
|  | **Men** | 0 (0) | 3.71 (2.41) | 130.75 (149.81) |
| **Sodium bicarbonate E500** | **All** | 23.87 (51.72) | 789.49 (400.54) | 3657.01 (2242.75) |
|  | **Women** | 28.78 (56.5) | 801.53 (387.33) | 3567.39 (2175.06) |
|  | **Men** | 5.93 (19.24) | 745.4 (442.85) | 3985.33 (2447.26) |
| **Beeswax E901** | **All** | 0 (0) | 0.25 (0.12) | 2.08 (2.56) |
|  | **Women** | 0 (0) | 0.25 (0.12) | 2.03 (2.5) |
|  | **Men** | 0 (0) | 0.24 (0.12) | 2.37 (2.88) |

Groups of emulsifiers were calculated as the sum of individual emulsifiers and defined as follows: total phosphates (E339, E 340, E341, E343, E450, E452), total lactylates (E481, E482), total polyglycerol esters of FAs (E475, E476), total mono and diglycerides of FAs (E471, E472, E472a, E472b, E472c, E472e), total celluloses (E460, E461, E464, E466, E468), total carrageenans (E407, E407a), total alginates (E400, E401, E402, E404, E405), total modified starches (E14xx).

The following emulsifiers were coded as sex-specific tertiles: total emulsifiers, total phosphates, total mono- and diglycerides of FAs, total carrageenans, total modified starches, E322, E407, E412, E415, E440, E450, E471, and E500. Due to a higher proportion of non- consumers among the included participants, the following emulsifiers were coded as non-consumers (1^st^ category), low consumers (2^nd^ category), and high consumers (3^rd^ category), with low- and high-consumptions defined according to sex-specific median intakes among consumers: total lactylates, total polyglycerol esters of FAs, total celluloses, total alginates, E331, E339, E340, E341, E401, E407a, E410, E414, E442, E451, E452, E460, E464, E466, E472c, E472b, E472e, E475, E476, E481, and E901. Cut-offs for intakes of: total emulsifier were 2701.3 and 5162.5 mg/d in men and 2618.5 and 4790.6 mg/d in women; total phosphates were 94 and 407.1 mg/d in men and 107.1 and 379.6 mg/d in women; total lactylates were 0.2 and 34.3 mg/d in men and 0.2 and 28.6 mg/d in women; polyglycerol esters of FAs were 0.0 and 29 mg/d in men and 0.0 and 25.7 mg/d in women; total mono- and diglycerides of FAs were 42.4 and 221.4 mg/d in men and 55 and 214.2 mg/d in women; total celluloses were 0.0 and 34.8 mg/d in men and 0.0 and 25.2 mg/d in women; total carrageenans were 12.5 and 75.3 mg/d in men and 13.5 and 66.6 mg/d in women; total alginates were 0.0 and 37.2 mg/d in men and 0.0 and 35.8 mg/d in women; total starches were 709 and 1607.7 mg/d in men and 671.7 and 1484 mg/d in women; E322 were 16.4 and 62.4 mg/d in men and 20.2 and 64.6 mg/d in women; E331 were 0.3 and 128.6 mg/d in men and 0.3 and 128.6 mg/d in women; E339 were 0.8 and 92.3 mg/d in men and 0.4 and 95.2 mg/d in women; E340 were 0.6 and 52.9 mg/d in men and 0.2 and 57.1 mg/d in women; E341 were 0.6 and 65.5 mg/d in men and 0.2 and 57.1 mg/d in women; E401 were 0.1 and 38.3 mg/d in men and 0.1 and 35.9 mg/d in women; E407 were 10.3 and 72 mg/d in men and 11.9 and 63.4 mg/d in women; E407a were 0.0 and 15.2 mg/d in men and 0.0 and 20.3 mg/d in women; E410 were 0.4 and 47.5 mg/d in men and 0.1 and 42.2 mg/d in women; E412 were 29.1 and 213.7 mg/d in men and 21.3 and 174.8 mg/d in women; E414 were 0.2 and 200.2 mg/d in men and 0.1 and 160.1 mg/d in women; E415 were 26.2 and 147.4 mg/d in men and 19 and 109.1 mg/d in women; E440 were 69.1 and 235.6 mg/d in men and 57.6 and 216.1 mg/d in women; E442 were 0.0 and 10.1 in men and 0.0 and 7.3 mg/d in women; E450 were 28 and 264.3 mg/d in men and 47.3 and 259.5 mg/d in women; E451 were 0.7 and 142.9 mg/d in men and 0.4 and 116.6 mg/d in women; E452 were 0.4 and 70.5 mg/d in men and 0.2 and 53.1 mg/d in women; E460 were 0.0 and 21.4 mg/d in men and 0.1 and 14.3 mg/d in women; E464 were 0.0 and 28.9 mg/d in men and 0.0 and 24.1 mg/d in women; E466 were 0.0 and 19.3 mg/d in men and 0.1 and 12.6 mg/d in women; E471 were 34 and 187.3 mg/d in men and 40.4 and 177.8 mg/d in women; E472b were 2.4 and 71.4 mg/d in men and 0.7 and 57.1 mg/d in women; E472c were 0.0 and 64.7 mg/d in men and 0.0 and 48.5 mg/d in women; E472e were 0.9 and 21 mg/d in men and 0.3 and 19.7 mg/d in women; E475 were 5.9 and 89.3 mg/d in men and 1.8 and 90.5 mg/d in women; E476 were 0.1 and 16.1 mg/d in men and 0.1 and 12.9 mg/d in women; E481 were 0.2 and 34.3 mg/d in men and 0.2 and 28.6 mg/d in women.; E500 were 100 and 1625 mg/d in men and 204 and 1550 mg/d in women; E901 were 0.0 and 0.5 mg/d in men and 0.0 and 0.5 mg/d in women.

**eTable C. Absolute risks of cancer at 60 years old according to categories of emulsifier intakes at the same age, NutriNet-Santé cohort, 2009-2021 (n=92,000)^a^**

|  |  | **Categories of emulsifier intakes** |  |
| --- | --- | --- | --- |
|  | **1 (low /none intake)** | **2 (medium / low)** | **3 (high intake)** |
| **Overall cancer** |  |  |  |
| **Total carrageenans**^b^ | 0.113 | 0.118 | 0.123 |
| **Carrageenan E407** | 0.115 | 0.118 | 0.121 |
| **Tripotassium phosphate E340** | 0.118 | 0.121 | 0.125 |
| **Diphosphates E450** | 0.117 | 0.118 | 0.119 |
| **Mono- and diglycerides of FAs E471** | 0.112 | 0.118 | 0.124 |
| **Polyglycerol esters of FAs E475** | 0.117 | 0.123 | 0.128 |
| **Carob bean gum E410** | 0.116 | 0.119 | 0.121 |
| **Guar gum E412** | 0.114 | 0.118 | 0.122 |
| **Gum arabic E414** | 0.117 | 0.121 | 0.123 |
| **Gum Xanthan Gum E415** | 0.113 | 0.118 | 0.123 |
| **Pectins E440** | 0.115 | 0.118 | 0.120 |
| **Sodium bicarbonate E500** | 0.116 | 0.118 | 0.120 |
| **Beeswax E901** | 0.118 | 0.123 | 0.128 |
| **Overall breast cancer** |  |  |  |
| **Total carrageenans**^b^ | 0.041 | 0.046 | 0.052 |
| **Carrageenan E407** | 0.041 | 0.046 | 0.052 |
| **Tripotassium phosphate E340** | 0.041 | 0.048 | 0.049 |
| **Diphosphates E450** | 0.043 | 0.046 | 0.051 |
| **Mono- and diglycerides of FAs E471** | 0.042 | 0.047 | 0.051 |
| **Polyglycerol esters of FAs E475** | 0.046 | 0.055 | 0.065 |
| **Carob bean gum E410** | 0.044 | 0.048 | 0.052 |
| **Guar gum E412** | 0.046 | 0.047 | 0.048 |
| **Gum arabic E414** | 0.046 | 0.048 | 0.051 |
| **Gum Xanthan Gum E415** | 0.044 | 0.046 | 0.049 |
| **Pectins E440** | 0.043 | 0.046 | 0.049 |
| **Sodium bicarbonate E500** | 0.044 | 0.047 | 0.049 |
| **Beeswax E901** | 0.046 | 0.051 | 0.057 |
| **Premenopausal breast cancer** |  |  |  |
| **Total carrageenans**^b^ | 0.019 | 0.021 | 0.024 |
| **Carrageenan E407** | 0.019 | 0.021 | 0.024 |
| **Tripotassium phosphate E340** | 0.021 | 0.027 | 0.034 |
| **Diphosphates E450** | 0.017 | 0.021 | 0.025 |
| **Mono- and diglycerides of FAs E471** | 0.019 | 0.021 | 0.024 |
| **Polyglycerol esters of FAs E475** | 0.021 | 0.025 | 0.031 |
| **Carob bean gum E410** | 0.021 | 0.021 | 0.021 |
| **Guar gum E412** | 0.022 | 0.021 | 0.021 |
| **Gum arabic E414** | 0.021 | 0.022 | 0.023 |
| **Gum Xanthan Gum E415** | 0.020 | 0.021 | 0.022 |
| **Pectins E440** | 0.017 | 0.021 | 0.025 |
| **Sodium bicarbonate E500** | 0.017 | 0.021 | 0.025 |
| **Beeswax E901** | 0.021 | 0.024 | 0.028 |
| **Postmenopausal breast cancer** |  |  |  |
| **Total carrageenans**^b^ | 0.063 | 0.071 | 0.080 |
| **Carrageenan E407** | 0.063 | 0.070 | 0.079 |
| **Tripotassium phosphate E340** | 0.073 | 0.060 | 0.050 |
| **Diphosphates E450** | 0.070 | 0.072 | 0.074 |
| **Mono- and diglycerides of FAs E471** | 0.066 | 0.071 | 0.077 |
| **Polyglycerol esters of FAs E475** | 0.069 | 0.082 | 0.096 |
| **Carob bean gum E410** | 0.066 | 0.073 | 0.081 |
| **Guar gum E412** | 0.069 | 0.072 | 0.074 |
| **Gum arabic E414** | 0.071 | 0.076 | 0.081 |
| **Gum Xanthan Gum E415** | 0.067 | 0.072 | 0.076 |
| **Pectins E440** | 0.074 | 0.072 | 0.070 |
| **Sodium bicarbonate E500** | 0.075 | 0.072 | 0.069 |
| **Beeswax E901** | 0.072 | 0.069 | 0.064 |
| **Prostate cancer** |  |  |  |
| **Total carrageenans**^b^ | 0.039 | 0.042 | 0.045 |
| **Carrageenan E407** | 0.041 | 0.042 | 0.044 |
| **Tripotassium phosphate E340** | 0.042 | 0.040 | 0.037 |
| **Diphosphates E450** | 0.041 | 0.042 | 0.044 |
| **Mono- and diglycerides of FAs E471** | 0.036 | 0.042 | 0.049 |
| **Polyglycerol esters of FAs E475** | 0.042 | 0.045 | 0.048 |
| **Carob bean gum E410** | 0.041 | 0.043 | 0.044 |
| **Guar gum E412** | 0.036 | 0.042 | 0.049 |
| **Gum arabic E414** | 0.041 | 0.054 | 0.068 |
| **Gum Xanthan Gum E415** | 0.038 | 0.042 | 0.042 |
| **Pectins E440** | 0.049 | 0.043 | 0.038 |
| **Sodium bicarbonate E500** | 0.041 | 0.042 | 0.044 |
| **Beeswax E901** | 0.041 | 0.058 | 0.081 |

^a^Absolute risks were obtained using the main Cox models followed by counterfactual scenarios applied to the overall cumulative incidence function (accounting for competing risks). All absolute risks are at 60 years old, except for premenopausal breast cancer (40 years old).

^b^ Groups of emulsifiers were calculated as the sum of individual emulsifiers and defined as follows: total carrageenans (E407, E407a).

**eTable D. Associations between emulsifier intakes and cancer risks among study participants from the NutriNet-Santé cohort, 2009-2021 (n=92,000).^a^**

|  |  | **Categories of emulsifier intakes ^b,c^** | | |  |
| --- | --- | --- | --- | --- | --- |
|  |  | **1 (low /none intake)** | **2 (medium / low)** | **3 (high intake)** | **P-trend** |
| **Overall cancer** |  |  |  |  |  |
| **Total emulsifiers** | **Cases/Participants** | 901 / 30667 | 923 / 30666 | 780 / 30667 |  |
|  | **HR (95%CI)** | 1.00 | 1.00 (0.91,1.10) | 1.00 (0.90,1.11) | p=0.9 |
| **Total alginates** | **Cases/Participants** | 2172 / 77519 | 242 / 7246 | 190 / 7235 |  |
|  | **HR (95%CI)** | 1.00 | 0.96 (0.84,1.10) | 0.99 (0.86,1.15) | p=0.8 |
| **Sodium alginate E401** | **Cases/Participants** | 2203 / 78211 | 225 / 7073 | 176 / 6716 |  |
|  | **HR (95%CI)** | 1.00 | 0.94 (0.82,1.08) | 1.00 (0.86,1.17) | p=0.7 |
| **Total carrageenans** | **Cases/Participants** | 753 / 30684 | 1029 / 30651 | 822 / 30665 |  |
|  | **HR (95%CI)** | 1.00 | 1.14 (1.03,1.25) | 1.12 (1.01,1.25) | p=0.06 |
| **Carrageenan E407** | **Cases/Participants** | 755 / 30667 | 1043 / 30692 | 806 / 30641 |  |
|  | **HR (95%CI)** | 1.00 | 1.14 (1.03,1.25) | 1.09 (0.98,1.21) | p=0.2 |
| **Processed Euchema Seaweed E407a** | **Cases/Participants** | 2309 / 83607 | 170 / 4442 | 125 / 3951 |  |
|  | **HR (95%CI)** | 1.00 | 1.14 (0.98,1.34) | 1.10 (0.92,1.32) | p=0.09 |
| **Total phosphates** | **Cases/Participants** | 862 / 30667 | 1019 / 30666 | 723 / 30667 |  |
|  | **HR (95%CI)** | 1.00 | 1.08 (0.98,1.18) | 1.02 (0.92,1.13) | p=0.7 |
| **Trisodium phosphate E339** | **Cases/Participants** | 2435 / 86423 | 98 / 2847 | 71 / 2730 |  |
|  | **HR (95%CI)** | 1.00 | 1.09 (0.89,1.33) | 1.09 (0.86,1.38) | p=0.3 |
| **Tripotassium phosphate E340** | **Cases/Participants** | 2445 / 86824 | 77 / 2605 | 82 / 2571 |  |
|  | **HR (95%CI)** | 1.00 | 0.98 (0.78,1.24) | 1.10 (0.88,1.38) | p=0.4 |
| **Tricalcium phosphate E341** | **Cases/Participants** | 2099 / 75438 | 326 / 8314 | 179 / 8248 |  |
|  | **HR (95%CI)** | 1.00 | 1.05 (0.93,1.18) | 0.86 (0.74,1.00) | p=0.2 |
| **Diphosphates E450** | **Cases/Participants** | 838 / 30683 | 1036 / 30658 | 730 / 30659 |  |
|  | **HR (95%CI)** | 1.00 | 1.03 (0.94,1.13) | 0.98 (0.88,1.09) | p=0.97 |
| **Triphosphates E451** | **Cases/Participants** | 1839 / 68381 | 445 / 11915 | 320 / 11704 |  |
|  | **HR (95%CI)** | 1.00 | 1.10 (0.99,1.23) | 1.11 (0.98,1.25) | p=0.03 |
| **Polyphosphates E452** | **Cases/Participants** | 1905 / 71283 | 385 / 10361 | 314 / 10356 |  |
|  | **HR (95%CI)** | 1.00 | 1.03 (0.92,1.15) | 1.08 (0.95,1.22) | p=0.2 |
| **Total celluloses** | **Cases/Participants** | 1996 / 72861 | 338 / 9591 | 270 / 9548 |  |
|  | **HR (95%CI)** | 1.00 | 1.16 (1.03,1.30) | 0.99 (0.87,1.12) | p=0.5 |
| **Cellulose E460** | **Cases/Participants** | 2240 / 82526 | 202 / 4770 | 162 / 4704 |  |
|  | **HR (95%CI)** | 1.00 | 1.23 (1.06,1.42) | 1.10 (0.94,1.29) | p=0.03 |
| **Hydroxypropylmethyl cellulose E464** | **Cases/Participants** | 2502 / 87945 | 45 / 2042 | 57 / 2013 |  |
|  | **HR (95%CI)** | 1.00 | 0.95 (0.70,1.27) | 0.96 (0.74,1.25) | p=0.7 |
| **Carboxymethyl cellulose E466** | **Cases/Participants** | 2310 / 82120 | 176 / 5001 | 118 / 4879 |  |
|  | **HR (95%CI)** | 1.00 | 1.11 (0.95,1.30) | 0.82 (0.68,0.99) | p=0.2 |
| **Total mono- and diglycerides of FAs** | **Cases/Participants** | 841 / 30675 | 1009 / 30658 | 754 / 30667 |  |
|  | **HR (95%CI)** | 1.00 | 1.12 (1.02,1.23) | 1.03 (0.93,1.14) | p=0.4 |
| **Mono- and diglycerides of FAs E471** | **Cases/Participants** | 790 / 30667 | 1018 / 30729 | 796 / 30604 |  |
|  | **HR (95%CI)** | 1.00 | 1.16 (1.05,1.27) | 1.15 (1.04,1.27) | p=0.01 |
| **Lactic acid esters of mono- and diglycerides of FAs E472b** | **Cases/Participants** | 2247 / 79718 | 204 / 6193 | 153 / 6089 |  |
|  | **HR (95%CI)** | 1.00 | 1.06 (0.92,1.23) | 0.91 (0.78,1.08) | p=0.5 |
| **Citric acid esters of mono- and diglycerides of FAs E472c** | **Cases/Participants** | 2329 / 85264 | 164 / 3390 | 111 / 3346 |  |
|  | **HR (95%CI)** | 1.00 | 1.10 (0.94,1.29) | 1.01 (0.84,1.23) | p=0.5 |
| **Acetyl tartaric acid esters of mono- and diglycerides of FAs E472e** | **Cases/Participants** | 2263 / 78827 | 230 / 6597 | 111 / 6576 |  |
|  | **HR (95%CI)** | 1.00 | 1.05 (0.92,1.21) | 0.83 (0.69,1.01) | p=0.2 |
| **Total polyglycerol esters of FAs** | **Cases/Participants** | 1956 / 72053 | 370 / 10006 | 278 / 9941 |  |
|  | **HR (95%CI)** | 1.00 | 1.03 (0.92,1.16) | 1.07 (0.94,1.21) | p=0.3 |
| **Polyglycerol esters of FAs E475** | **Cases/Participants** | 2431 / 85557 | 103 / 3237 | 70 / 3206 |  |
|  | **HR (95%CI)** | 1.00 | 1.09 (0.89,1.33) | 1.09 (0.86,1.39) | p=0.3 |
| **Polyglycerol esters of interesterified ricinoleic acid E476** | **Cases/Participants** | 2090 / 77272 | 285 / 7409 | 229 / 7319 |  |
|  | **HR (95%CI)** | 1.00 | 1.05 (0.93,1.20) | 1.01 (0.88,1.16) | p=0.7 |
| **Total lactylates** | **Cases/Participants** | 2393 / 84054 | 125 / 3992 | 86 / 3954 |  |
|  | **HR (95%CI)** | 1.00 | 0.89 (0.74,1.06) | 1.01 (0.81,1.25) | p=0.6 |
| **Sodium stearoyl-2-lactylate E481** | **Cases/Participants** | 2395 / 84175 | 124 / 3934 | 85 / 3891 |  |
|  | **HR (95%CI)** | 1.00 | 0.89 (0.74,1.07) | 1.01 (0.81,1.26) | p=0.6 |
| **Total modified starches** | **Cases/Participants** | 778 / 30668 | 910 / 30711 | 916 / 30621 |  |
|  | **HR (95%CI)** | 1.00 | 1.09 (0.99,1.20) | 1.03 (0.94,1.14) | p=0.7 |
| **Lecithins E322** | **Cases/Participants** | 1073 / 30667 | 956 / 30667 | 575 / 30666 |  |
|  | **HR (95%CI)** | 1.00 | 1.01 (0.92,1.10) | 1.05 (0.94,1.18) | p=0.5 |
| **Sodium citrate E331** | **Cases/Participants** | 1261 / 46381 | 834 / 22867 | 509 / 22752 |  |
|  | **HR (95%CI)** | 1.00 | 1.09 (0.99,1.19) | 1.05 (0.94,1.17) | p=0.2 |
| **Carob bean gum E410** | **Cases/Participants** | 1277 / 49966 | 747 / 21402 | 580 / 20632 |  |
|  | **HR (95%CI)** | 1.00 | 1.06 (0.96,1.16) | 1.05 (0.95,1.16) | p=0.3 |
| **Guar gum E412** | **Cases/Participants** | 733 / 30693 | 996 / 30640 | 875 / 30667 |  |
|  | **HR (95%CI)** | 1.00 | 1.11 (1.01,1.23) | 1.08 (0.97,1.19) | p=0.1 |
| **Gum arabic E414** | **Cases/Participants** | 2342 / 82111 | 151 / 5015 | 111 / 4874 |  |
|  | **HR (95%CI)** | 1.00 | 1.10 (0.93,1.30) | 1.07 (0.88,1.30) | p=0.3 |
| **Gum Xanthan Gum E415** | **Cases/Participants** | 735 / 30670 | 928 / 30663 | 941 / 30667 |  |
|  | **HR (95%CI)** | 1.00 | 1.12 (1.02,1.24) | 1.13 (1.02,1.25) | p=0.04 |
| **Pectins E440** | **Cases/Participants** | 657 / 30676 | 985 / 30963 | 962 / 30361 |  |
|  | **HR (95%CI)** | 1.00 | 1.09 (0.99,1.21) | 1.07 (0.97,1.19) | p=0.4 |
| **Ammonium salts of phosphatidic acid E442** | **Cases/Participants** | 2288 / 82241 | 174 / 4880 | 142 / 4879 |  |
|  | **HR (95%CI)** | 1.00 | 1.07 (0.91,1.25) | 1.05 (0.89,1.25) | p=0.4 |
| **Sodium bicarbonate E500** | **Cases/Participants** | 841 / 30705 | 978 / 30629 | 785 / 30666 |  |
|  | **HR (95%CI)** | 1.00 | 1.02 (0.93,1.12) | 1.00 (0.90,1.11) | p=0.7 |
| **Beeswax E901** | **Cases/Participants** | 2465 / 86421 | 76 / 2802 | 63 / 2777 |  |
|  | **HR (95%CI)** | 1.00 | 0.93 (0.74,1.18) | 1.18 (0.91,1.52) | p=0.5 |
| **Overall breast cancer** |  |  |  |  |  |
| **Total emulsifiers** | **Cases/Participants** | 244 / 24090 | 266 / 24090 | 238 / 24090 |  |
|  | **HR (95%CI)** | 1.00 | 1.03 (0.86,1.23) | 1.01 (0.84,1.22) | p=0.8 |
| **Total alginates** | **Cases/Participants** | 603 / 60610 | 80 / 5830 | 65 / 5830 |  |
|  | **HR (95%CI)** | 1.00 | 1.06 (0.83,1.34) | 1.10 (0.85,1.43) | p=0.5 |
| **Sodium alginate E401** | **Cases/Participants** | 611 / 61134 | 79 / 5742 | 58 / 5394 |  |
|  | **HR (95%CI)** | 1.00 | 1.08 (0.85,1.36) | 1.07 (0.82,1.41) | p=0.5 |
| **Total carrageenans** | **Cases/Participants** | 192 / 24107 | 283 / 24075 | 273 / 24088 |  |
|  | **HR (95%CI)** | 1.00 | 1.17 (0.97,1.42) | 1.32 (1.09,1.60) | p=0.009 |
| **Carrageenan E407** | **Cases/Participants** | 194 / 24090 | 283 / 24116 | 271 / 24064 |  |
|  | **HR (95%CI)** | 1.00 | 1.15 (0.95,1.39) | 1.28 (1.06,1.56) | p=0.01 |
| **Processed Euchema Seaweed E407a** | **Cases/Participants** | 654 / 65816 | 58 / 3472 | 36 / 2982 |  |
|  | **HR (95%CI)** | 1.00 | 1.32 (1.01,1.73) | 1.15 (0.82,1.62) | p=0.1 |
| **Total phosphates** | **Cases/Participants** | 233 / 24090 | 299 / 24090 | 216 / 24090 |  |
|  | **HR (95%CI)** | 1.00 | 1.12 (0.94,1.34) | 1.01 (0.83,1.22) | p=0.96 |
| **Trisodium phosphate E339** | **Cases/Participants** | 699 / 67693 | 33 / 2327 | 16 / 2250 |  |
|  | **HR (95%CI)** | 1.00 | 1.19 (0.83,1.69) | 0.78 (0.47,1.27) | p=0.7 |
| **Tripotassium phosphate E340** | **Cases/Participants** | 700 / 67935 | 20 / 2184 | 28 / 2151 |  |
|  | **HR (95%CI)** | 1.00 | 0.76 (0.49,1.19) | 1.15 (0.79,1.68) | p=0.7 |
| **Tricalcium phosphate E341** | **Cases/Participants** | 597 / 58724 | 96 / 6796 | 55 / 6750 |  |
|  | **HR (95%CI)** | 1.00 | 0.99 (0.79,1.24) | 0.80 (0.61,1.06) | p=0.1 |
| **Diphosphates E450** | **Cases/Participants** | 207 / 24101 | 312 / 24084 | 229 / 24085 |  |
|  | **HR (95%CI)** | 1.00 | 1.24 (1.03,1.49) | 1.14 (0.94,1.40) | p=0.08 |
| **Triphosphates E451** | **Cases/Participants** | 531 / 54260 | 126 / 9005 | 91 / 9005 |  |
|  | **HR (95%CI)** | 1.00 | 1.14 (0.93,1.40) | 1.11 (0.88,1.39) | p=0.2 |
| **Polyphosphates E452** | **Cases/Participants** | 538 / 55831 | 112 / 8222 | 98 / 8217 |  |
|  | **HR (95%CI)** | 1.00 | 1.06 (0.86,1.31) | 1.16 (0.93,1.44) | p=0.2 |
| **Total celluloses** | **Cases/Participants** | 547 / 56290 | 114 / 8010 | 87 / 7970 |  |
|  | **HR (95%CI)** | 1.00 | 1.26 (1.02,1.54) | 0.99 (0.79,1.24) | p=0.5 |
| **Cellulose E460** | **Cases/Participants** | 636 / 64288 | 58 / 4020 | 54 / 3962 |  |
|  | **HR (95%CI)** | 1.00 | 1.14 (0.87,1.50) | 1.13 (0.85,1.49) | p=0.3 |
| **Hydroxypropylmethyl cellulose E464** | **Cases/Participants** | 719 / 68943 | 14 / 1677 | 15 / 1650 |  |
|  | **HR (95%CI)** | 1.00 | 0.89 (0.52,1.51) | 0.77 (0.46,1.29) | p=0.3 |
| **Carboxymethyl cellulose E466** | **Cases/Participants** | 648 / 63880 | 60 / 4255 | 40 / 4135 |  |
|  | **HR (95%CI)** | 1.00 | 1.14 (0.87,1.49) | 0.83 (0.61,1.15) | p=0.6 |
| **Total mono- and diglycerides of FAs** | **Cases/Participants** | 219 / 24090 | 298 / 24090 | 231 / 24090 |  |
|  | **HR (95%CI)** | 1.00 | 1.24 (1.04,1.48) | 1.13 (0.93,1.37) | p=0.2 |
| **Mono- and diglycerides of FAs E471** | **Cases/Participants** | 211 / 24090 | 292 / 24153 | 245 / 24027 |  |
|  | **HR (95%CI)** | 1.00 | 1.20 (1.00,1.44) | 1.24 (1.03,1.51) | p=0.04 |
| **Lactic acid esters of mono- and diglycerides of FAs E472b** | **Cases/Participants** | 628 / 62386 | 71 / 4966 | 49 / 4918 |  |
|  | **HR (95%CI)** | 1.00 | 1.21 (0.94,1.55) | 0.93 (0.69,1.25) | p=0.8 |
| **Citric acid esters of mono- and diglycerides of FAs E472c** | **Cases/Participants** | 672 / 66949 | 46 / 2674 | 30 / 2647 |  |
|  | **HR (95%CI)** | 1.00 | 1.07 (0.79,1.45) | 0.94 (0.65,1.36) | p=0.99 |
| **Acetyl tartaric acid esters of mono- and diglycerides of FAs E472e** | **Cases/Participants** | 638 / 61402 | 73 / 5440 | 37 / 5428 |  |
|  | **HR (95%CI)** | 1.00 | 1.07 (0.84,1.37) | 0.86 (0.62,1.20) | p=0.7 |
| **Total polyglycerol esters of FAs** | **Cases/Participants** | 546 / 56225 | 115 / 8039 | 87 / 8006 |  |
|  | **HR (95%CI)** | 1.00 | 1.10 (0.90,1.35) | 1.08 (0.86,1.36) | p=0.3 |
| **Polyglycerol esters of FAs E475** | **Cases/Participants** | 683 / 67018 | 33 / 2626 | 32 / 2626 |  |
|  | **HR (95%CI)** | 1.00 | 1.10 (0.77,1.56) | 1.50 (1.05,2.15) | p=0.02 |
| **Polyglycerol esters of interesterified ricinoleic acid E476** | **Cases/Participants** | 597 / 60489 | 90 / 5933 | 61 / 5848 |  |
|  | **HR (95%CI)** | 1.00 | 1.11 (0.88,1.39) | 0.92 (0.70,1.19) | p=0.7 |
| **Total lactylates** | **Cases/Participants** | 670 / 65779 | 45 / 3246 | 33 / 3245 |  |
|  | **HR (95%CI)** | 1.00 | 1.05 (0.78,1.43) | 1.22 (0.86,1.73) | p=0.3 |
| **Sodium stearoyl-2-lactylate E481** | **Cases/Participants** | 671 / 65880 | 44 / 3198 | 33 / 3192 |  |
|  | **HR (95%CI)** | 1.00 | 1.05 (0.77,1.43) | 1.23 (0.87,1.76) | p=0.2 |
| **Total modified starches** | **Cases/Participants** | 213 / 24090 | 278 / 24136 | 257 / 24044 |  |
|  | **HR (95%CI)** | 1.00 | 1.18 (0.99,1.42) | 1.05 (0.87,1.26) | p=0.7 |
| **Lecithins E322** | **Cases/Participants** | 278 / 24090 | 292 / 24091 | 178 / 24089 |  |
|  | **HR (95%CI)** | 1.00 | 1.12 (0.95,1.33) | 1.09 (0.88,1.36) | p=0.5 |
| **Sodium citrate E331** | **Cases/Participants** | 345 / 35792 | 242 / 18293 | 161 / 18185 |  |
|  | **HR (95%CI)** | 1.00 | 1.05 (0.89,1.25) | 1.07 (0.88,1.30) | p=0.5 |
| **Carob bean gum E410** | **Cases/Participants** | 330 / 38896 | 233 / 17072 | 185 / 16302 |  |
|  | **HR (95%CI)** | 1.00 | 1.21 (1.01,1.44) | 1.19 (0.99,1.43) | p=0.045 |
| **Guar gum E412** | **Cases/Participants** | 208 / 24116 | 290 / 24064 | 250 / 24090 |  |
|  | **HR (95%CI)** | 1.00 | 1.11 (0.92,1.33) | 1.02 (0.84,1.23) | p=0.7 |
| **Gum arabic E414** | **Cases/Participants** | 653 / 63902 | 57 / 4254 | 38 / 4114 |  |
|  | **HR (95%CI)** | 1.00 | 1.22 (0.92,1.60) | 1.03 (0.74,1.43) | p=0.5 |
| **Gum Xanthan Gum E415** | **Cases/Participants** | 196 / 24093 | 276 / 24087 | 276 / 24090 |  |
|  | **HR (95%CI)** | 1.00 | 1.21 (1.00,1.45) | 1.16 (0.96,1.40) | p=0.2 |
| **Pectins E440** | **Cases/Participants** | 172 / 24095 | 281 / 24389 | 295 / 23786 |  |
|  | **HR (95%CI)** | 1.00 | 1.12 (0.92,1.36) | 1.15 (0.95,1.41) | p=0.2 |
| **Ammonium salts of phosphatidic acid E442** | **Cases/Participants** | 655 / 64583 | 53 / 3844 | 40 / 3843 |  |
|  | **HR (95%CI)** | 1.00 | 1.12 (0.84,1.48) | 0.99 (0.72,1.36) | p=0.8 |
| **Sodium bicarbonate E500** | **Cases/Participants** | 214 / 24118 | 293 / 24062 | 241 / 24090 |  |
|  | **HR (95%CI)** | 1.00 | 1.16 (0.97,1.39) | 1.09 (0.90,1.33) | p=0.3 |
| **Beeswax E901** | **Cases/Participants** | 692 / 67515 | 33 / 2383 | 23 / 2372 |  |
|  | **HR (95%CI)** | 1.00 | 1.13 (0.79,1.61) | 1.18 (0.77,1.80) | p=0.3 |
| **Premenopausal breast cancer** |  |  |  |  |  |
| **Total emulsifiers** | **Cases/Participants** | 80 / 16704 | 98 / 17432 | 116 / 18827 |  |
|  | **HR (95%CI)** | 1.00 | 1.07 (0.79,1.45) | 1.25 (0.92,1.69) | p=0.09 |
| **Total alginates** | **Cases/Participants** | 228 / 44104 | 32 / 4273 | 34 / 4586 |  |
|  | **HR (95%CI)** | 1.00 | 1.03 (0.71,1.51) | 1.32 (0.92,1.90) | p=0.2 |
| **Sodium alginate E401** | **Cases/Participants** | 231 / 44438 | 33 / 4265 | 30 / 4260 |  |
|  | **HR (95%CI)** | 1.00 | 1.08 (0.74,1.57) | 1.27 (0.86,1.86) | p=0.2 |
| **Total carrageenans** | **Cases/Participants** | 64 / 17077 | 117 / 17189 | 113 / 18697 |  |
|  | **HR (95%CI)** | 1.00 | 1.42 (1.04,1.95) | 1.37 (0.99,1.89) | p=0.2 |
| **Carrageenan E407** | **Cases/Participants** | 65 / 17093 | 117 / 17230 | 112 / 18640 |  |
|  | **HR (95%CI)** | 1.00 | 1.39 (1.02,1.91) | 1.34 (0.98,1.85) | p=0.1 |
| **Processed Euchema Seaweed E407a** | **Cases/Participants** | 249 / 48049 | 25 / 2562 | 20 / 2352 |  |
|  | **HR (95%CI)** | 1.00 | 1.42 (0.94,2.16) | 1.43 (0.90,2.26) | p=0.05 |
| **Total phosphates** | **Cases/Participants** | 68 / 16330 | 115 / 17175 | 111 / 19458 |  |
|  | **HR (95%CI)** | 1.00 | 1.36 (1.00,1.86) | 1.29 (0.94,1.78) | p=0.1 |
| **Trisodium phosphate E339** | **Cases/Participants** | 277 / 49348 | 10 / 1772 | 7 / 1843 |  |
|  | **HR (95%CI)** | 1.00 | 0.83 (0.44,1.56) | 0.69 (0.32,1.46) | p=0.3 |
| **Tripotassium phosphate E340** | **Cases/Participants** | 267 / 49714 | 10 / 1657 | 17 / 1592 |  |
|  | **HR (95%CI)** | 1.00 | 0.98 (0.52,1.85) | 1.77 (1.08,2.91) | p=0.03 |
| **Tricalcium phosphate E341** | **Cases/Participants** | 230 / 43051 | 35 / 4557 | 29 / 5355 |  |
|  | **HR (95%CI)** | 1.00 | 1.06 (0.74,1.53) | 0.93 (0.63,1.37) | p=0.8 |
| **Diphosphates E450** | **Cases/Participants** | 61 / 16560 | 120 / 17021 | 113 / 19382 |  |
|  | **HR (95%CI)** | 1.00 | 1.56 (1.13,2.15) | 1.45 (1.04,2.02) | p=0.03 |
| **Triphosphates E451** | **Cases/Participants** | 200 / 39327 | 53 / 6555 | 41 / 7081 |  |
|  | **HR (95%CI)** | 1.00 | 1.24 (0.90,1.70) | 1.13 (0.80,1.59) | p=0.3 |
| **Polyphosphates E452** | **Cases/Participants** | 204 / 41177 | 45 / 5616 | 45 / 6170 |  |
|  | **HR (95%CI)** | 1.00 | 1.27 (0.91,1.76) | 1.34 (0.96,1.86) | p=0.05 |
| **Total celluloses** | **Cases/Participants** | 204 / 41105 | 55 / 5956 | 35 / 5902 |  |
|  | **HR (95%CI)** | 1.00 | 1.58 (1.16,2.14) | 1.03 (0.72,1.48) | p=0.3 |
| **Cellulose E460** | **Cases/Participants** | 247 / 47354 | 25 / 2742 | 22 / 2867 |  |
|  | **HR (95%CI)** | 1.00 | 1.46 (0.96,2.22) | 1.18 (0.76,1.83) | p=0.2 |
| **Hydroxypropylmethyl cellulose E464** | **Cases/Participants** | 282 / 50300 | 7 / 1428 | 5 / 1235 |  |
|  | **HR (95%CI)** | 1.00 | 0.84 (0.39,1.78) | 0.60 (0.25,1.46) | p=0.2 |
| **Carboxymethyl cellulose E466** | **Cases/Participants** | 256 / 46744 | 21 / 3123 | 17 / 3096 |  |
|  | **HR (95%CI)** | 1.00 | 0.97 (0.62,1.52) | 0.85 (0.52,1.39) | p=0.5 |
| **Total mono- and diglycerides of FAs** | **Cases/Participants** | 72 / 16499 | 114 / 17400 | 108 / 19064 |  |
|  | **HR (95%CI)** | 1.00 | 1.33 (0.99,1.80) | 1.26 (0.92,1.72) | p=0.3 |
| **Mono- and diglycerides of FAs E471** | **Cases/Participants** | 73 / 16654 | 110 / 17284 | 111 / 19025 |  |
|  | **HR (95%CI)** | 1.00 | 1.23 (0.91,1.67) | 1.29 (0.95,1.76) | p=0.1 |
| **Lactic acid esters of mono- and diglycerides of FAs E472b** | **Cases/Participants** | 240 / 45360 | 29 / 3764 | 25 / 3839 |  |
|  | **HR (95%CI)** | 1.00 | 1.19 (0.80,1.76) | 1.07 (0.70,1.62) | p=0.5 |
| **Citric acid esters of mono- and diglycerides of FAs E472c** | **Cases/Participants** | 274 / 49491 | 10 / 1605 | 10 / 1867 |  |
|  | **HR (95%CI)** | 1.00 | 0.76 (0.40,1.44) | 0.81 (0.43,1.52) | p=0.4 |
| **Acetyl tartaric acid esters of mono- and diglycerides of FAs E472e** | **Cases/Participants** | 244 / 44421 | 33 / 3975 | 17 / 4567 |  |
|  | **HR (95%CI)** | 1.00 | 1.29 (0.89,1.87) | 0.80 (0.49,1.31) | p=0.9 |
| **Total polyglycerol esters of FAs** | **Cases/Participants** | 209 / 41136 | 48 / 5552 | 37 / 6275 |  |
|  | **HR (95%CI)** | 1.00 | 1.32 (0.96,1.82) | 1.05 (0.74,1.50) | p=0.4 |
| **Polyglycerol esters of FAs E475** | **Cases/Participants** | 262 / 48656 | 15 / 2065 | 17 / 2242 |  |
|  | **HR (95%CI)** | 1.00 | 1.11 (0.65,1.88) | 1.50 (0.92,2.46) | p=0.1 |
| **Polyglycerol esters of interesterified ricinoleic acid E476** | **Cases/Participants** | 233 / 44643 | 44 / 4136 | 17 / 4184 |  |
|  | **HR (95%CI)** | 1.00 | 1.56 (1.12,2.17) | 0.68 (0.42,1.12) | p=0.8 |
| **Total lactylates** | **Cases/Participants** | 260 / 47960 | 17 / 2283 | 17 / 2720 |  |
|  | **HR (95%CI)** | 1.00 | 1.03 (0.63,1.69) | 1.25 (0.76,2.05) | p=0.4 |
| **Sodium stearoyl-2-lactylate E481** | **Cases/Participants** | 261 / 48041 | 16 / 2245 | 17 / 2677 |  |
|  | **HR (95%CI)** | 1.00 | 0.98 (0.59,1.64) | 1.25 (0.76,2.06) | p=0.5 |
| **Total modified starches** | **Cases/Participants** | 82 / 18019 | 116 / 18333 | 96 / 16611 |  |
|  | **HR (95%CI)** | 1.00 | 1.23 (0.93,1.64) | 1.17 (0.86,1.58) | p=0.4 |
| **Lecithins E322** | **Cases/Participants** | 83 / 14378 | 118 / 17380 | 93 / 21205 |  |
|  | **HR (95%CI)** | 1.00 | 1.09 (0.82,1.46) | 0.89 (0.64,1.25) | p=0.3 |
| **Sodium citrate E331** | **Cases/Participants** | 115 / 25142 | 94 / 12802 | 85 / 15019 |  |
|  | **HR (95%CI)** | 1.00 | 1.24 (0.93,1.65) | 1.30 (0.97,1.73) | p=0.09 |
| **Carob bean gum E410** | **Cases/Participants** | 125 / 28265 | 100 / 12254 | 69 / 12444 |  |
|  | **HR (95%CI)** | 1.00 | 1.41 (1.06,1.86) | 1.07 (0.79,1.45) | p=0.5 |
| **Guar gum E412** | **Cases/Participants** | 79 / 17423 | 122 / 17568 | 93 / 17972 |  |
|  | **HR (95%CI)** | 1.00 | 1.21 (0.90,1.63) | 0.94 (0.69,1.28) | p=0.8 |
| **Gum arabic E414** | **Cases/Participants** | 250 / 46093 | 24 / 3416 | 20 / 3454 |  |
|  | **HR (95%CI)** | 1.00 | 1.04 (0.68,1.59) | 1.06 (0.67,1.69) | p=0.8 |
| **Gum Xanthan Gum E415** | **Cases/Participants** | 71 / 17423 | 119 / 17631 | 104 / 17909 |  |
|  | **HR (95%CI)** | 1.00 | 1.45 (1.08,1.97) | 1.15 (0.84,1.57) | p=0.5 |
| **Pectins E440** | **Cases/Participants** | 60 / 18635 | 114 / 17402 | 120 / 16926 |  |
|  | **HR (95%CI)** | 1.00 | 1.44 (1.05,1.99) | 1.55 (1.12,2.14) | p=0.008 |
| **Ammonium salts of phosphatidic acid E442** | **Cases/Participants** | 250 / 47097 | 23 / 2857 | 21 / 3009 |  |
|  | **HR (95%CI)** | 1.00 | 1.23 (0.80,1.88) | 1.18 (0.75,1.84) | p=0.3 |
| **Sodium bicarbonate E500** | **Cases/Participants** | 63 / 16630 | 113 / 17272 | 118 / 19061 |  |
|  | **HR (95%CI)** | 1.00 | 1.46 (1.06,2.01) | 1.48 (1.07,2.05) | p=0.01 |
| **Beeswax E901** | **Cases/Participants** | 266 / 49020 | 11 / 1890 | 17 / 2053 |  |
|  | **HR (95%CI)** | 1.00 | 0.78 (0.42,1.44) | 1.56 (0.95,2.57) | p=0.2 |
| **Postmenopausal breast cancer** |  |  |  |  |  |
| **Total emulsifiers** | **Cases/Participants** | 164 / 10107 | 168 / 9487 | 122 / 7836 |  |
|  | **HR (95%CI)** | 1.00 | 1.01 (0.81,1.25) | 0.87 (0.68,1.12) | p=0.3 |
| **Total alginates** | **Cases/Participants** | 375 / 23202 | 48 / 2339 | 31 / 1889 |  |
|  | **HR (95%CI)** | 1.00 | 1.06 (0.78,1.44) | 0.93 (0.64,1.35) | p=0.8 |
| **Sodium alginate E401** | **Cases/Participants** | 380 / 23468 | 46 / 2243 | 28 / 1719 |  |
|  | **HR (95%CI)** | 1.00 | 1.07 (0.78,1.45) | 0.92 (0.63,1.36) | p=0.9 |
| **Total carrageenans** | **Cases/Participants** | 128 / 9458 | 166 / 9852 | 160 / 8120 |  |
|  | **HR (95%CI)** | 1.00 | 1.04 (0.82,1.32) | 1.28 (1.00,1.64) | p=0.04 |
| **Carrageenan E407** | **Cases/Participants** | 129 / 9405 | 166 / 9866 | 159 / 8159 |  |
|  | **HR (95%CI)** | 1.00 | 1.02 (0.80,1.29) | 1.25 (0.97,1.59) | p=0.06 |
| **Processed Euchema Seaweed E407a** | **Cases/Participants** | 405 / 25088 | 33 / 1389 | 16 / 953 |  |
|  | **HR (95%CI)** | 1.00 | 1.22 (0.86,1.75) | 0.91 (0.55,1.51) | p=0.8 |
| **Total phosphates** | **Cases/Participants** | 165 / 10350 | 184 / 9953 | 105 / 7127 |  |
|  | **HR (95%CI)** | 1.00 | 1.00 (0.81,1.24) | 0.84 (0.65,1.09) | p=0.2 |
| **Trisodium phosphate E339** | **Cases/Participants** | 422 / 25912 | 23 / 840 | 9 / 678 |  |
|  | **HR (95%CI)** | 1.00 | 1.44 (0.94,2.21) | 0.85 (0.44,1.65) | p=0.6 |
| **Tripotassium phosphate E340** | **Cases/Participants** | 433 / 25795 | 10 / 801 | 11 / 834 |  |
|  | **HR (95%CI)** | 1.00 | 0.62 (0.33,1.17) | 0.75 (0.41,1.36) | p=0.2 |
| **Tricalcium phosphate E341** | **Cases/Participants** | 367 / 22093 | 61 / 3202 | 26 / 2135 |  |
|  | **HR (95%CI)** | 1.00 | 0.95 (0.72,1.25) | 0.70 (0.47,1.05) | p=0.08 |
| **Diphosphates E450** | **Cases/Participants** | 146 / 10072 | 192 / 10149 | 116 / 7209 |  |
|  | **HR (95%CI)** | 1.00 | 1.11 (0.88,1.38) | 0.99 (0.77,1.29) | p=0.7 |
| **Triphosphates E451** | **Cases/Participants** | 331 / 20932 | 73 / 3616 | 50 / 2882 |  |
|  | **HR (95%CI)** | 1.00 | 1.07 (0.82,1.39) | 1.08 (0.80,1.47) | p=0.5 |
| **Polyphosphates E452** | **Cases/Participants** | 334 / 20788 | 67 / 3672 | 53 / 2970 |  |
|  | **HR (95%CI)** | 1.00 | 0.94 (0.72,1.23) | 1.01 (0.75,1.36) | p=0.96 |
| **Total celluloses** | **Cases/Participants** | 343 / 21289 | 59 / 3098 | 52 / 3043 |  |
|  | **HR (95%CI)** | 1.00 | 1.06 (0.80,1.40) | 0.97 (0.72,1.30) | p=0.9 |
| **Cellulose E460** | **Cases/Participants** | 389 / 23983 | 33 / 1857 | 32 / 1590 |  |
|  | **HR (95%CI)** | 1.00 | 0.98 (0.69,1.41) | 1.08 (0.75,1.56) | p=0.7 |
| **Hydroxypropylmethyl cellulose E464** | **Cases/Participants** | 437 / 26388 | 7 / 409 | 10 / 633 |  |
|  | **HR (95%CI)** | 1.00 | 0.94 (0.44,1.99) | 0.91 (0.49,1.71) | p=0.7 |
| **Carboxymethyl cellulose E466** | **Cases/Participants** | 392 / 24179 | 39 / 1693 | 23 / 1558 |  |
|  | **HR (95%CI)** | 1.00 | 1.26 (0.90,1.75) | 0.81 (0.53,1.24) | p=0.8 |
| **Total mono- and diglycerides of FAs** | **Cases/Participants** | 147 / 10280 | 184 / 9646 | 123 / 7504 |  |
|  | **HR (95%CI)** | 1.00 | 1.17 (0.94,1.47) | 1.04 (0.81,1.33) | p=0.5 |
| **Mono- and diglycerides of FAs E471** | **Cases/Participants** | 138 / 10053 | 182 / 9929 | 134 / 7448 |  |
|  | **HR (95%CI)** | 1.00 | 1.17 (0.93,1.46) | 1.20 (0.94,1.54) | p=0.2 |
| **Lactic acid esters of mono- and diglycerides of FAs E472b** | **Cases/Participants** | 388 / 23951 | 42 / 1792 | 24 / 1687 |  |
|  | **HR (95%CI)** | 1.00 | 1.21 (0.87,1.67) | 0.81 (0.54,1.23) | p=0.7 |
| **Citric acid esters of mono- and diglycerides of FAs E472c** | **Cases/Participants** | 398 / 24786 | 36 / 1503 | 20 / 1141 |  |
|  | **HR (95%CI)** | 1.00 | 1.21 (0.86,1.71) | 1.03 (0.66,1.61) | p=0.5 |
| **Acetyl tartaric acid esters of mono- and diglycerides of FAs E472e** | **Cases/Participants** | 394 / 23933 | 40 / 2165 | 20 / 1332 |  |
|  | **HR (95%CI)** | 1.00 | 0.94 (0.67,1.30) | 0.93 (0.59,1.46) | p=0.7 |
| **Total polyglycerol esters of FAs** | **Cases/Participants** | 337 / 21229 | 67 / 3606 | 50 / 2595 |  |
|  | **HR (95%CI)** | 1.00 | 0.98 (0.75,1.28) | 1.10 (0.82,1.49) | p=0.6 |
| **Polyglycerol esters of FAs E475** | **Cases/Participants** | 421 / 25917 | 18 / 891 | 15 / 622 |  |
|  | **HR (95%CI)** | 1.00 | 1.08 (0.67,1.73) | 1.49 (0.89,2.50) | p=0.1 |
| **Polyglycerol esters of interesterified ricinoleic acid E476** | **Cases/Participants** | 364 / 22426 | 46 / 2662 | 44 / 2342 |  |
|  | **HR (95%CI)** | 1.00 | 0.87 (0.64,1.19) | 1.06 (0.77,1.45) | p=0.8 |
| **Total lactylates** | **Cases/Participants** | 410 / 25229 | 28 / 1376 | 16 / 825 |  |
|  | **HR (95%CI)** | 1.00 | 1.05 (0.71,1.54) | 1.20 (0.73,1.98) | p=0.4 |
| **Sodium stearoyl-2-lactylate E481** | **Cases/Participants** | 410 / 25261 | 28 / 1357 | 16 / 812 |  |
|  | **HR (95%CI)** | 1.00 | 1.07 (0.73,1.57) | 1.22 (0.74,2.02) | p=0.4 |
| **Total modified starches** | **Cases/Participants** | 131 / 8626 | 162 / 8632 | 161 / 10172 |  |
|  | **HR (95%CI)** | 1.00 | 1.14 (0.90,1.44) | 0.98 (0.77,1.25) | p=0.8 |
| **Lecithins E322** | **Cases/Participants** | 195 / 12755 | 174 / 9810 | 85 / 4865 |  |
|  | **HR (95%CI)** | 1.00 | 1.12 (0.91,1.39) | 1.29 (0.97,1.72) | p=0.1 |
| **Sodium citrate E331** | **Cases/Participants** | 230 / 14561 | 148 / 8000 | 76 / 4869 |  |
|  | **HR (95%CI)** | 1.00 | 0.96 (0.77,1.19) | 0.91 (0.70,1.20) | p=0.6 |
| **Carob bean gum E410** | **Cases/Participants** | 205 / 14641 | 133 / 7062 | 116 / 5727 |  |
|  | **HR (95%CI)** | 1.00 | 1.09 (0.87,1.37) | 1.27 (1.00,1.61) | p=0.05 |
| **Guar gum E412** | **Cases/Participants** | 129 / 9105 | 168 / 9363 | 157 / 8962 |  |
|  | **HR (95%CI)** | 1.00 | 1.04 (0.82,1.31) | 1.06 (0.83,1.34) | p=0.5 |
| **Gum arabic E414** | **Cases/Participants** | 403 / 24979 | 33 / 1375 | 18 / 1076 |  |
|  | **HR (95%CI)** | 1.00 | 1.36 (0.95,1.95) | 0.99 (0.61,1.59) | p=0.5 |
| **Gum Xanthan Gum E415** | **Cases/Participants** | 125 / 9111 | 157 / 9125 | 172 / 9194 |  |
|  | **HR (95%CI)** | 1.00 | 1.05 (0.83,1.34) | 1.16 (0.91,1.47) | p=0.3 |
| **Pectins E440** | **Cases/Participants** | 112 / 7566 | 167 / 9897 | 175 / 9967 |  |
|  | **HR (95%CI)** | 1.00 | 0.95 (0.74,1.22) | 0.96 (0.74,1.24) | p=0.6 |
| **Ammonium salts of phosphatidic acid E442** | **Cases/Participants** | 405 / 24661 | 30 / 1511 | 19 / 1258 |  |
|  | **HR (95%CI)** | 1.00 | 1.05 (0.72,1.52) | 0.83 (0.52,1.32) | p=0.6 |
| **Sodium bicarbonate E500** | **Cases/Participants** | 151 / 9994 | 180 / 9777 | 123 / 7659 |  |
|  | **HR (95%CI)** | 1.00 | 1.03 (0.82,1.30) | 0.90 (0.70,1.16) | p=0.5 |
| **Beeswax E901** | **Cases/Participants** | 426 / 26090 | 22 / 805 | 6 / 535 |  |
|  | **HR (95%CI)** | 1.00 | 1.42 (0.92,2.19) | 0.71 (0.31,1.59) | p=0.8 |
| **Prostate cancer** |  |  |  |  |  |
| **Total emulsifiers** | **Cases/Participants** | 115 / 6577 | 124 / 6576 | 83 / 6577 |  |
|  | **HR (95%CI)** | 1.00 | 1.05 (0.81,1.37) | 0.96 (0.71,1.30) | p=0.8 |
| **Total alginates** | **Cases/Participants** | 265 / 16909 | 38 / 1416 | 19 / 1405 |  |
|  | **HR (95%CI)** | 1.00 | 1.23 (0.87,1.74) | 0.95 (0.59,1.51) | p=0.7 |
| **Sodium alginate E401** | **Cases/Participants** | 271 / 17077 | 33 / 1331 | 18 / 1322 |  |
|  | **HR (95%CI)** | 1.00 | 1.16 (0.80,1.67) | 0.98 (0.61,1.59) | p=0.7 |
| **Total carrageenans** | **Cases/Participants** | 88 / 6577 | 144 / 6576 | 90 / 6577 |  |
|  | **HR (95%CI)** | 1.00 | 1.32 (1.01,1.74) | 1.20 (0.88,1.65) | p=0.3 |
| **Carrageenan E407** | **Cases/Participants** | 88 / 6577 | 148 / 6576 | 86 / 6577 |  |
|  | **HR (95%CI)** | 1.00 | 1.34 (1.02,1.76) | 1.14 (0.83,1.56) | p=0.6 |
| **Processed Euchema Seaweed E407a** | **Cases/Participants** | 281 / 17791 | 21 / 970 | 20 / 969 |  |
|  | **HR (95%CI)** | 1.00 | 1.14 (0.73,1.78) | 1.28 (0.81,2.02) | p=0.2 |
| **Total phosphates** | **Cases/Participants** | 99 / 6577 | 145 / 6576 | 78 / 6577 |  |
|  | **HR (95%CI)** | 1.00 | 1.32 (1.01,1.71) | 1.10 (0.81,1.50) | p=0.5 |
| **Trisodium phosphate E339** | **Cases/Participants** | 308 / 18730 | 4 / 520 | 10 / 480 |  |
|  | **HR (95%CI)** | 1.00 | 0.37 (0.14,1.00) | 1.51 (0.80,2.84) | p=0.96 |
| **Tripotassium phosphate E340** | **Cases/Participants** | 308 / 18889 | 9 / 421 | 5 / 420 |  |
|  | **HR (95%CI)** | 1.00 | 1.19 (0.61,2.32) | 0.76 (0.31,1.84) | p=0.8 |
| **Tricalcium phosphate E341** | **Cases/Participants** | 271 / 16714 | 36 / 1518 | 15 / 1498 |  |
|  | **HR (95%CI)** | 1.00 | 0.98 (0.69,1.39) | 0.75 (0.44,1.26) | p=0.3 |
| **Diphosphates E450** | **Cases/Participants** | 94 / 6582 | 149 / 6574 | 79 / 6574 |  |
|  | **HR (95%CI)** | 1.00 | 1.19 (0.91,1.55) | 1.00 (0.73,1.38) | p=0.8 |
| **Triphosphates E451** | **Cases/Participants** | 219 / 14121 | 67 / 2910 | 36 / 2699 |  |
|  | **HR (95%CI)** | 1.00 | 1.19 (0.89,1.58) | 1.06 (0.74,1.51) | p=0.5 |
| **Polyphosphates E452** | **Cases/Participants** | 243 / 15452 | 46 / 2139 | 33 / 2139 |  |
|  | **HR (95%CI)** | 1.00 | 0.94 (0.68,1.30) | 0.92 (0.64,1.33) | p=0.6 |
| **Total celluloses** | **Cases/Participants** | 260 / 16571 | 35 / 1581 | 27 / 1578 |  |
|  | **HR (95%CI)** | 1.00 | 1.18 (0.83,1.69) | 1.05 (0.71,1.57) | p=0.6 |
| **Cellulose E460** | **Cases/Participants** | 279 / 18238 | 26 / 750 | 17 / 742 |  |
|  | **HR (95%CI)** | 1.00 | 1.58 (1.05,2.38) | 1.18 (0.72,1.93) | p=0.1 |
| **Hydroxypropylmethyl cellulose E464** | **Cases/Participants** | 316 / 19002 | 4 / 365 | 2 / 363 |  |
|  | **HR (95%CI)** | 1.00 | 0.79 (0.30,2.14) | 0.30 (0.08,1.22) | p=0.08 |
| **Carboxymethyl cellulose E466** | **Cases/Participants** | 293 / 18240 | 15 / 746 | 14 / 744 |  |
|  | **HR (95%CI)** | 1.00 | 1.02 (0.61,1.72) | 1.10 (0.64,1.88) | p=0.7 |
| **Total mono- and diglycerides of FAs** | **Cases/Participants** | 100 / 6585 | 126 / 6568 | 96 / 6577 |  |
|  | **HR (95%CI)** | 1.00 | 1.14 (0.87,1.49) | 1.23 (0.92,1.65) | p=0.06 |
| **Mono- and diglycerides of FAs E471** | **Cases/Participants** | 86 / 6577 | 133 / 6576 | 103 / 6577 |  |
|  | **HR (95%CI)** | 1.00 | 1.32 (1.00,1.74) | 1.46 (1.09,1.97) | p=0.02 |
| **Lactic acid esters of mono- and diglycerides of FAs E472b** | **Cases/Participants** | 278 / 17332 | 32 / 1227 | 12 / 1171 |  |
|  | **HR (95%CI)** | 1.00 | 1.46 (1.00,2.11) | 0.70 (0.39,1.26) | p=0.9 |
| **Citric acid esters of mono- and diglycerides of FAs E472c** | **Cases/Participants** | 288 / 18315 | 27 / 716 | 7 / 699 |  |
|  | **HR (95%CI)** | 1.00 | 1.34 (0.90,1.99) | 0.49 (0.23,1.04) | p=0.4 |
| **Acetyl tartaric acid esters of mono- and diglycerides of FAs E472e** | **Cases/Participants** | 287 / 17425 | 26 / 1157 | 9 / 1148 |  |
|  | **HR (95%CI)** | 1.00 | 1.06 (0.71,1.60) | 0.80 (0.41,1.55) | p=0.7 |
| **Total polyglycerol esters of FAs** | **Cases/Participants** | 247 / 15828 | 45 / 1967 | 30 / 1935 |  |
|  | **HR (95%CI)** | 1.00 | 0.99 (0.72,1.37) | 1.08 (0.73,1.58) | p=0.8 |
| **Polyglycerol esters of FAs E475** | **Cases/Participants** | 304 / 18539 | 12 / 611 | 6 / 580 |  |
|  | **HR (95%CI)** | 1.00 | 1.14 (0.64,2.03) | 1.14 (0.51,2.57) | p=0.6 |
| **Polyglycerol esters of interesterified ricinoleic acid E476** | **Cases/Participants** | 262 / 16783 | 36 / 1476 | 24 / 1471 |  |
|  | **HR (95%CI)** | 1.00 | 1.06 (0.74,1.51) | 0.89 (0.59,1.36) | p=0.8 |
| **Total lactylates** | **Cases/Participants** | 301 / 18275 | 15 / 746 | 6 / 709 |  |
|  | **HR (95%CI)** | 1.00 | 0.96 (0.57,1.61) | 0.80 (0.36,1.81) | p=0.6 |
| **Sodium stearoyl-2-lactylate E481** | **Cases/Participants** | 301 / 18295 | 15 / 736 | 6 / 699 |  |
|  | **HR (95%CI)** | 1.00 | 0.97 (0.57,1.63) | 0.81 (0.36,1.83) | p=0.6 |
| **Total modified starches** | **Cases/Participants** | 105 / 6578 | 107 / 6575 | 110 / 6577 |  |
|  | **HR (95%CI)** | 1.00 | 0.94 (0.71,1.23) | 0.91 (0.69,1.20) | p=0.5 |
| **Lecithins E322** | **Cases/Participants** | 134 / 6577 | 129 / 6576 | 59 / 6577 |  |
|  | **HR (95%CI)** | 1.00 | 1.10 (0.86,1.42) | 1.24 (0.89,1.74) | p=0.2 |
| **Sodium citrate E331** | **Cases/Participants** | 156 / 10589 | 117 / 4574 | 49 / 4567 |  |
|  | **HR (95%CI)** | 1.00 | 1.28 (1.00,1.65) | 1.12 (0.81,1.56) | p=0.2 |
| **Carob bean gum E410** | **Cases/Participants** | 165 / 11070 | 88 / 4330 | 69 / 4330 |  |
|  | **HR (95%CI)** | 1.00 | 0.94 (0.72,1.22) | 1.12 (0.84,1.49) | p=0.6 |
| **Guar gum E412** | **Cases/Participants** | 81 / 6577 | 127 / 6576 | 114 / 6577 |  |
|  | **HR (95%CI)** | 1.00 | 1.18 (0.89,1.58) | 1.39 (1.04,1.87) | p=0.02 |
| **Gum arabic E414** | **Cases/Participants** | 296 / 18209 | 9 / 761 | 17 / 760 |  |
|  | **HR (95%CI)** | 1.00 | 0.71 (0.36,1.38) | 2.53 (1.54,4.15) | p=0.009 |
| **Gum Xanthan Gum E415** | **Cases/Participants** | 83 / 6577 | 126 / 6576 | 113 / 6577 |  |
|  | **HR (95%CI)** | 1.00 | 1.37 (1.03,1.81) | 1.30 (0.97,1.73) | p=0.1 |
| **Pectins E440** | **Cases/Participants** | 89 / 6581 | 129 / 6574 | 104 / 6575 |  |
|  | **HR (95%CI)** | 1.00 | 0.94 (0.71,1.24) | 0.78 (0.57,1.07) | p=0.07 |
| **Ammonium salts of phosphatidic acid E442** | **Cases/Participants** | 287 / 17658 | 19 / 1036 | 16 / 1036 |  |
|  | **HR (95%CI)** | 1.00 | 0.89 (0.56,1.41) | 1.02 (0.61,1.70) | p=0.8 |
| **Sodium bicarbonate E500** | **Cases/Participants** | 91 / 6587 | 148 / 6567 | 83 / 6576 |  |
|  | **HR (95%CI)** | 1.00 | 1.33 (1.01,1.74) | 1.04 (0.76,1.42) | p=0.8 |
| **Beeswax E901** | **Cases/Participants** | 307 / 18906 | 8 / 419 | 7 / 405 |  |
|  | **HR (95%CI)** | 1.00 | 1.36 (0.67,2.76) | 2.43 (1.14,5.18) | p=0.03 |
| **Colorectal cancer** |  |  |  |  |  |
| **Total emulsifiers** | **Cases/Participants** | 69 / 30667 | 69 / 30666 | 69 / 30667 |  |
|  | **HR (95%CI)** | 1.00 | 1.05 (0.74,1.47) | 1.39 (0.98,1.99) | p=0.07 |
| **Total alginates** | **Cases/Participants** | 174 / 77519 | 20 / 7246 | 13 / 7235 |  |
|  | **HR (95%CI)** | 1.00 | 1.00 (0.62,1.60) | 0.90 (0.51,1.59) | p=0.8 |
| **Sodium alginate E401** | **Cases/Participants** | 176 / 78211 | 20 / 7073 | 11 / 6716 |  |
|  | **HR (95%CI)** | 1.00 | 1.07 (0.67,1.71) | 0.83 (0.45,1.54) | p=0.7 |
| **Total carrageenans** | **Cases/Participants** | 63 / 30684 | 87 / 30651 | 57 / 30665 |  |
|  | **HR (95%CI)** | 1.00 | 1.16 (0.83,1.62) | 1.01 (0.69,1.47) | p=0.7 |
| **Carrageenan E407** | **Cases/Participants** | 64 / 30667 | 85 / 30692 | 58 / 30641 |  |
|  | **HR (95%CI)** | 1.00 | 1.10 (0.78,1.54) | 1.00 (0.69,1.45) | p=0.9 |
| **Processed Euchema Seaweed E407a** | **Cases/Participants** | 180 / 83607 | 14 / 4442 | 13 / 3951 |  |
|  | **HR (95%CI)** | 1.00 | 1.20 (0.69,2.07) | 1.55 (0.88,2.74) | p=0.1 |
| **Total phosphates** | **Cases/Participants** | 67 / 30667 | 86 / 30666 | 54 / 30667 |  |
|  | **HR (95%CI)** | 1.00 | 1.22 (0.88,1.69) | 1.15 (0.79,1.67) | p=0.4 |
| **Trisodium phosphate E339** | **Cases/Participants** | 189 / 86423 | 12 / 2847 | 6 / 2730 |  |
|  | **HR (95%CI)** | 1.00 | 1.73 (0.96,3.13) | 1.28 (0.57,2.90) | p=0.2 |
| **Tripotassium phosphate E340** | **Cases/Participants** | 195 / 86824 | 6 / 2605 | 6 / 2571 |  |
|  | **HR (95%CI)** | 1.00 | 0.97 (0.43,2.19) | 1.03 (0.46,2.34) | p=0.9 |
| **Tricalcium phosphate E341** | **Cases/Participants** | 159 / 75438 | 36 / 8314 | 12 / 8248 |  |
|  | **HR (95%CI)** | 1.00 | 1.51 (1.04,2.20) | 0.84 (0.47,1.52) | p=0.6 |
| **Diphosphates E450** | **Cases/Participants** | 66 / 30683 | 89 / 30658 | 52 / 30659 |  |
|  | **HR (95%CI)** | 1.00 | 1.16 (0.83,1.62) | 1.04 (0.70,1.53) | p=0.4 |
| **Triphosphates E451** | **Cases/Participants** | 147 / 68381 | 37 / 11915 | 23 / 11704 |  |
|  | **HR (95%CI)** | 1.00 | 1.10 (0.75,1.60) | 1.01 (0.64,1.57) | p=0.9 |
| **Polyphosphates E452** | **Cases/Participants** | 151 / 71283 | 42 / 10361 | 14 / 10356 |  |
|  | **HR (95%CI)** | 1.00 | 1.32 (0.93,1.88) | 0.60 (0.35,1.05) | p=0.3 |
| **Total celluloses** | **Cases/Participants** | 169 / 72861 | 21 / 9591 | 17 / 9548 |  |
|  | **HR (95%CI)** | 1.00 | 0.82 (0.52,1.31) | 0.76 (0.46,1.25) | p=0.2 |
| **Cellulose E460** | **Cases/Participants** | 187 / 82526 | 11 / 4770 | 9 / 4704 |  |
|  | **HR (95%CI)** | 1.00 | 0.72 (0.39,1.33) | 0.71 (0.36,1.39) | p=0.2 |
| **Hydroxypropylmethyl cellulose E464** | **Cases/Participants** | 198 / 87945 | 1 / 2042 | 8 / 2013 |  |
|  | **HR (95%CI)** | 1.00 | 0.30 (0.04,2.12) | 1.84 (0.90,3.74) | p=0.3 |
| **Carboxymethyl cellulose E466** | **Cases/Participants** | 183 / 82120 | 15 / 5001 | 9 / 4879 |  |
|  | **HR (95%CI)** | 1.00 | 1.20 (0.71,2.05) | 0.80 (0.41,1.57) | p=0.8 |
| **Total mono- and diglycerides of FAs** | **Cases/Participants** | 76 / 30675 | 78 / 30658 | 53 / 30667 |  |
|  | **HR (95%CI)** | 1.00 | 0.97 (0.70,1.33) | 0.85 (0.59,1.23) | p=0.4 |
| **Mono- and diglycerides of FAs E471** | **Cases/Participants** | 73 / 30667 | 75 / 30729 | 59 / 30604 |  |
|  | **HR (95%CI)** | 1.00 | 0.92 (0.66,1.28) | 0.98 (0.69,1.41) | p=0.9 |
| **Lactic acid esters of mono- and diglycerides of FAs E472b** | **Cases/Participants** | 177 / 79718 | 20 / 6193 | 10 / 6089 |  |
|  | **HR (95%CI)** | 1.00 | 1.39 (0.87,2.23) | 0.81 (0.43,1.55) | p=0.95 |
| **Citric acid esters of mono- and diglycerides of FAs E472c** | **Cases/Participants** | 173 / 85264 | 23 / 3390 | 11 / 3346 |  |
|  | **HR (95%CI)** | 1.00 | 1.95 (1.25,3.03) | 1.31 (0.71,2.42) | p=0.03 |
| **Acetyl tartaric acid esters of mono- and diglycerides of FAs E472e** | **Cases/Participants** | 180 / 78827 | 19 / 6597 | 8 / 6576 |  |
|  | **HR (95%CI)** | 1.00 | 1.08 (0.67,1.75) | 0.84 (0.41,1.71) | p=0.7 |
| **Total polyglycerol esters of FAs** | **Cases/Participants** | 161 / 72053 | 29 / 10006 | 17 / 9941 |  |
|  | **HR (95%CI)** | 1.00 | 0.94 (0.63,1.41) | 0.84 (0.51,1.38) | p=0.4 |
| **Polyglycerol esters of FAs E475** | **Cases/Participants** | 195 / 85557 | 9 / 3237 | 3 / 3206 |  |
|  | **HR (95%CI)** | 1.00 | 1.28 (0.65,2.51) | 0.68 (0.22,2.14) | p=0.9 |
| **Polyglycerol esters of interesterified ricinoleic acid E476** | **Cases/Participants** | 170 / 77272 | 21 / 7409 | 16 / 7319 |  |
|  | **HR (95%CI)** | 1.00 | 0.91 (0.58,1.45) | 0.85 (0.51,1.42) | p=0.4 |
| **Total lactylates** | **Cases/Participants** | 196 / 84054 | 2 / 3992 | 9 / 3954 |  |
|  | **HR (95%CI)** | 1.00 | 0.16 (0.04,0.67) | 1.44 (0.74,2.82) | p=0.5 |
| **Sodium stearoyl-2-lactylate E481** | **Cases/Participants** | 196 / 84175 | 2 / 3934 | 9 / 3891 |  |
|  | **HR (95%CI)** | 1.00 | 0.17 (0.04,0.67) | 1.46 (0.75,2.87) | p=0.6 |
| **Total modified starches** | **Cases/Participants** | 54 / 30668 | 69 / 30711 | 84 / 30621 |  |
|  | **HR (95%CI)** | 1.00 | 1.22 (0.85,1.75) | 1.40 (0.99,2.00) | p=0.08 |
| **Lecithins E322** | **Cases/Participants** | 95 / 30667 | 72 / 30667 | 40 / 30666 |  |
|  | **HR (95%CI)** | 1.00 | 0.95 (0.69,1.31) | 1.17 (0.77,1.77) | p=0.5 |
| **Sodium citrate E331** | **Cases/Participants** | 105 / 46381 | 70 / 22867 | 32 / 22752 |  |
|  | **HR (95%CI)** | 1.00 | 1.09 (0.79,1.49) | 0.86 (0.57,1.29) | p=0.7 |
| **Carob bean gum E410** | **Cases/Participants** | 100 / 49966 | 72 / 21402 | 35 / 20632 |  |
|  | **HR (95%CI)** | 1.00 | 1.31 (0.95,1.80) | 0.85 (0.57,1.26) | p=0.8 |
| **Guar gum E412** | **Cases/Participants** | 57 / 30693 | 85 / 30640 | 65 / 30667 |  |
|  | **HR (95%CI)** | 1.00 | 1.24 (0.87,1.75) | 1.04 (0.73,1.50) | p=0.7 |
| **Gum arabic E414** | **Cases/Participants** | 188 / 82111 | 15 / 5015 | 4 / 4874 |  |
|  | **HR (95%CI)** | 1.00 | 1.49 (0.87,2.54) | 0.56 (0.21,1.51) | p=0.8 |
| **Gum Xanthan Gum E415** | **Cases/Participants** | 59 / 30670 | 74 / 30663 | 74 / 30667 |  |
|  | **HR (95%CI)** | 1.00 | 1.12 (0.79,1.59) | 1.11 (0.78,1.58) | p=0.4 |
| **Pectins E440** | **Cases/Participants** | 49 / 30676 | 79 / 30963 | 79 / 30361 |  |
|  | **HR (95%CI)** | 1.00 | 1.22 (0.84,1.77) | 1.28 (0.87,1.87) | p=0.2 |
| **Ammonium salts of phosphatidic acid E442** | **Cases/Participants** | 179 / 82241 | 13 / 4880 | 15 / 4879 |  |
|  | **HR (95%CI)** | 1.00 | 1.03 (0.59,1.82) | 1.49 (0.88,2.54) | p=0.2 |
| **Sodium bicarbonate E500** | **Cases/Participants** | 66 / 30705 | 76 / 30629 | 65 / 30666 |  |
|  | **HR (95%CI)** | 1.00 | 1.08 (0.76,1.52) | 1.26 (0.87,1.81) | p=0.3 |
| **Beeswax E901** | **Cases/Participants** | 198 / 86421 | 6 / 2802 | 3 / 2777 |  |
|  | **HR (95%CI)** | 1.00 | 1.03 (0.45,2.33) | 0.85 (0.27,2.66) | p=0.9 |

^a^ Multivariable Cox proportional hazard models were adjusted for age (time-scale), sex, BMI (continuous, kg/m²), height (continuous, cm), physical activity (categorical IPAQ variable: high, moderate, low), smoking status (never smoked, former smoker, current smokers), number of smoked cigarettes in pack-years (continuous), educational level (less than high school degree, <2 y after high school degree, ≥2 y after high school degree), number of dietary records (continuous), family history of cancer (yes/no), energy intake without alcohol (continuous, kcal/d), daily intakes of alcohol (continuous, g/d), total lipid intake (continuous, g/d), dietary sodium (continuous, mg/d), total fiber (continuous, g/d), total sugars (continuous, g/d), fruits and vegetables (g/d), total dairy foods (continuous, g/d) and red and processed meats (continuous, g/d). Finally, breast cancer models were additionally adjusted for oral contraception (yes/no, in total and premenopausal models only), age at menarche (never, <12 y, ≥12 y), number of biological children (continuous), age at first biological child (no child, <30 y, ≥30 y), menopausal status at baseline (premenopausal, postmenopausal, in total models only), hormonal treatment for menopause (yes/no, in total and postmenopausal models only).

^b^ Groups of emulsifiers were calculated as the sum of individual emulsifiers and defined as follows: total phosphates (E339, E 340, E341, E343, E450, E452), total lactylates (E481, E482), total polyglycerol esters of FAs (E475, E476), total mono and diglycerides of FAs (E471, E472, E472a, E472b, E472c, E472e), total celluloses (E460, E461, E464, E466, E468), total carrageenans (E407, E407a), total alginates (E400, E401, E402, E404, E405), total modified starches (E14xx).

^c^ The following emulsifiers were coded as sex-specific tertiles: total emulsifiers, total phosphates, total mono- and diglycerides of FAs, total carrageenans, total modified starches, E322, E407, E412, E415, E440, E450, E471, and E500. Due to a higher proportion of non- consumers among the included participants, the following emulsifiers were coded as non-consumers (1^st^ category), low consumers (2^nd^ category), and high consumers (3^rd^ category), with low- and high-consumptions defined according to sex-specific median intakes among consumers: total lactylates, total polyglycerol esters of FAs, total celluloses, total alginates, E331, E339, E340, E341, E401, E407a, E410, E414, E442, E451, E452, E460, E464, E466, E472c, E472b, E472e, E475, E476, E481, and E901. Cut-offs for intakes of: total emulsifier were 2701.3 and 5162.5 mg/d in men and 2618.5 and 4790.6 mg/d in women; total phosphates were 94 and 407.1 mg/d in men and 107.1 and 379.6 mg/d in women; total lactylates were 0.2 and 34.3 mg/d in men and 0.2 and 28.6 mg/d in women; polyglycerol esters of FAs were 0.0 and 29 mg/d in men and 0.0 and 25.7 mg/d in women; total mono- and diglycerides of FAs were 42.4 and 221.4 mg/d in men and 55 and 214.2 mg/d in women; total celluloses were 0.0 and 34.8 mg/d in men and 0.0 and 25.2 mg/d in women; total carrageenans were 12.5 and 75.3 mg/d in men and 13.5 and 66.6 mg/d in women; total alginates were 0.0 and 37.2 mg/d in men and 0.0 and 35.8 mg/d in women; total starches were 709 and 1607.7 mg/d in men and 671.7 and 1484 mg/d in women; E322 were 16.4 and 62.4 mg/d in men and 20.2 and 64.6 mg/d in women; E331 were 0.3 and 128.6 mg/d in men and 0.3 and 128.6 mg/d in women; E339 were 0.8 and 92.3 mg/d in men and 0.4 and 95.2 mg/d in women; E340 were 0.6 and 52.9 mg/d in men and 0.2 and 57.1 mg/d in women; E341 were 0.6 and 65.5 mg/d in men and 0.2 and 57.1 mg/d in women; E401 were 0.1 and 38.3 mg/d in men and 0.1 and 35.9 mg/d in women; E407 were 10.3 and 72 mg/d in men and 11.9 and 63.4 mg/d in women; E407a were 0.0 and 15.2 mg/d in men and 0.0 and 20.3 mg/d in women; E410 were 0.4 and 47.5 mg/d in men and 0.1 and 42.2 mg/d in women; E412 were 29.1 and 213.7 mg/d in men and 21.3 and 174.8 mg/d in women; E414 were 0.2 and 200.2 mg/d in men and 0.1 and 160.1 mg/d in women; E415 were 26.2 and 147.4 mg/d in men and 19 and 109.1 mg/d in women; E440 were 69.1 and 235.6 mg/d in men and 57.6 and 216.1 mg/d in women; E442 were 0.0 and 10.1 in men and 0.0 and 7.3 mg/d in women; E450 were 28 and 264.3 mg/d in men and 47.3 and 259.5 mg/d in women; E451 were 0.7 and 142.9 mg/d in men and 0.4 and 116.6 mg/d in women; E452 were 0.4 and 70.5 mg/d in men and 0.2 and 53.1 mg/d in women; E460 were 0.0 and 21.4 mg/d in men and 0.1 and 14.3 mg/d in women; E464 were 0.0 and 28.9 mg/d in men and 0.0 and 24.1 mg/d in women; E466 were 0.0 and 19.3 mg/d in men and 0.1 and 12.6 mg/d in women; E471 were 34 and 187.3 mg/d in men and 40.4 and 177.8 mg/d in women; E472b were 2.4 and 71.4 mg/d in men and 0.7 and 57.1 mg/d in women; E472c were 0.0 and 64.7 mg/d in men and 0.0 and 48.5 mg/d in women; E472e were 0.9 and 21 mg/d in men and 0.3 and 19.7 mg/d in women; E475 were 5.9 and 89.3 mg/d in men and 1.8 and 90.5 mg/d in women; E476 were 0.1 and 16.1 mg/d in men and 0.1 and 12.9 mg/d in women; E481 were 0.2 and 34.3 mg/d in men and 0.2 and 28.6 mg/d in women.; E500 were 100 and 1625 mg/d in men and 204 and 1550 mg/d in women; E901 were 0.0 and 0.5 mg/d in men and 0.0 and 0.5 mg/d in women.

**Sensitivity Analyses**

In sensitivity analyses, main results remained overall similar after further adjustments for the intakes of other emulsifiers than the one studied in the model (model 1), for the proportion of ultra-processed food (% weight) in the diet (model 2), for artificial sweetener intakes (model 3) and when using the average of all available 24h dietary records throughout the follow-up of each participant (up to 62 records per participant) instead of averaged values on their first two years of follow-up (model 4), except when stated otherwise in the main text (Results section). False discovery rate adjusted p-trend values were calculated for the associations between overall, total breast, premenopausal breast, postmenopausal breast, and prostate cancer risks and intakes of: total carrageenans (p-trend = 0.2, 0.08, 0.2, 0.2 and 0.6 respectively), E407 (p-trend = 0.5, 0.08, 0.2, 0.2 and 0.8 respectively), E340 (p-trend = 0.5, 0.7, 0.1, 0.4 and 0.8 respectively), E450 (p-trend = 0.9, 0.2, 0.1, 0.7 and 0.8 respectively), E471 (p-trend = 0.1, 0.1, 0.2, 0.4 and 0.1 respectively), E475 (p-trend = 0.5, 0.1, 0.2, 0.4 and 0.8 respectively), E410 (p-trend = 0.5, 0.1, 0.6, 0.2 and 0.8 respectively), E412 (p-trend = 0.4, 0.7, 0.8, 0.7 and 0.1 respectively), E414 (p-trend = 0.5, 0.5, 0.8, 0.7 and 0.1 respectively), E415 (p-trend = 0.2, 0.3, 0.6, 0.5 and 0.3 respectively), E440 (p-trend = 0.5, 0.3, 0.09, 0.7 and 0.2 respectively), E500 (p-trend = 0.8, 0.4, 0.09, 0.7 and 0.8 respectively), and E901 (p-trend = 0.5, 0.4, 0.3, 0.8 and 0.1 respectively). All statistically significant associations observed in this study in main and sensitivity analyses went in the same direction (direct associations, Figure 4, eTable E, eTable F), which limits the likelihood of chance finding “at random”. “Any” versus “none” comparisons were also computed (eTable F). In principal component analyses (eTable G), the first component, characterized particularly by high intakes of carrageenans, E410 and E471, was positively associated with increased breast cancer risk (p-trend=0.03), consistently with our main models when these emulsifiers were assessed individually. Components 2 and 3 were not associated with cancer risk, but it was expected since they were characterized by both high consumption of some emulsifiers and low consumption of some others, blurring the associations, compared to the approach where emulsifiers were investigated separately.

**eTable E. Sensitivity analyses for the associations between emulsifier intakes and cancer risks among study participants from the NutriNet-Santé cohort, 2009-2021 (n=92,000).^a^**

|  |  |  | | | **Categories of emulsifier intakes ^b^** | | | | | | | | |  | |
| --- | --- | --- | --- | --- | --- | --- | --- | --- | --- | --- | --- | --- | --- | --- | --- |
|  | **Sensitivity model** |  | | | **1 (low /none intake)** | | | **2 (medium / low)** | | | **3 (high intake)** | | | **P-trend** | |
| **Overall cancer** |  |  | | |  | | |  | | |  | | |  | |
| **Total carrageenans** | **model 1** | **Cases/Participants** | | | 753 / 30684 | | | 1029 / 30651 | | | 822 / 30665 | | |  | |
|  |  | **HR (95%CI)** | | | 1.00 | | | 1.14 (1.04,1.26) | | | 1.13 (1.02,1.26) | | | p=0.04 | |
|  | **model 2** | **Cases/Participants** | | | 753 / 30684 | | | 1029 / 30651 | | | 822 / 30665 | | |  | |
|  |  | **HR (95%CI)** | | | 1.00 | | | 1.14 (1.03,1.26) | | | 1.13 (1.02,1.25) | | | p=0.047 | |
|  | **model 3** | **Cases/Participants** | | | 753 / 30684 | | | 1029 / 30651 | | | 822 / 30665 | | |  | |
|  |  | **HR (95%CI)** | | | 1.00 | | | 1.14 (1.03,1.25) | | | 1.12 (1.01,1.25) | | | p=0.1 | |
|  | **model 4** | **Cases/Participants** | | | 731 / 30664 | | | 1049 / 30666 | | | 824 / 30666 | | |  | |
|  |  | **HR (95%CI)** | | | 1.00 | | | 1.1 (0.99,1.21) | | | 1.2 (1.08,1.34) | | | p<0.001 | |
| **Carrageenan E407** | **model 1** | **Cases/Participants** | | | 755 / 30667 | | | 1043 / 30692 | | | 806 / 30641 | | |  | |
|  |  | **HR (95%CI)** | | | 1.00 | | | 1.14 (1.04,1.26) | | | 1.10 (0.99,1.22) | | | p=0.2 | |
|  | **model 2** | **Cases/Participants** | | | 755 / 30667 | | | 1043 / 30692 | | | 806 / 30641 | | |  | |
|  |  | **HR (95%CI)** | | | 1.00 | | | 1.14 (1.03,1.26) | | | 1.09 (0.98,1.21) | | | p=0.2 | |
|  | **model 3** | **Cases/Participants** | | | 755 / 30667 | | | 1043 / 30692 | | | 806 / 30641 | | |  | |
|  |  | **HR (95%CI)** | | | 1.00 | | | 1.14 (1.03,1.25) | | | 1.08 (0.98,1.21) | | | p=0.2 | |
|  | **model 4** | **Cases/Participants** | | | 719 / 30664 | | | 1053 / 30666 | | | 822 / 30666 | | |  | |
|  |  | **HR (95%CI)** | | | 1.00 | | | 1.12 (1.02,1.24) | | | 1.21 (1.09,1.35) | | | p<0.001 | |
| **Tripotassium phosphate E340** | **model 1** | **Cases/Participants** | | | 2445 / 86824 | | | 77 / 2605 | | | 82 / 2571 | | |  | |
|  |  | **HR (95%CI)** | | | 1.00 | | | 0.99 (0.79,1.24) | | | 1.12 (0.89,1.39) | | | p=0.3 | |
|  | **model 2** | **Cases/Participants** | | | 2445 / 86824 | | | 77 / 2605 | | | 82 / 2571 | | |  | |
|  |  | **HR (95%CI)** | | | 1.00 | | | 0.99 (0.79,1.24) | | | 1.11 (0.89,1.39) | | | p=0.4 | |
|  | **model 3** | **Cases/Participants** | | | 2445 / 86824 | | | 77 / 2605 | | | 82 / 2571 | | |  | |
|  |  | **HR (95%CI)** | | | 1.00 | | | 0.98 (0.78,1.24) | | | 1.10 (0.88,1.37) | | | p=0.4 | |
|  | **model 4** | **Cases/Participants** | | | 2331 / 83113 | | | 128 / 4440 | | | 145 / 4443 | | |  | |
|  |  | **HR (95%CI)** | | | 1.00 | | | 0.66 (0.55,0.79) | | | 0.95 (0.80,1.12) | | | p=0.01 | |
| **Diphosphates E450** | **model 1** | **Cases/Participants** | | | 838 / 30683 | | | 1036 / 30658 | | | 730 / 30659 | | |  | |
|  |  | **HR (95%CI)** | | | 1.00 | | | 1.03 (0.94,1.14) | | | 0.99 (0.88,1.11) | | | p=0.7 | |
|  | **model 2** | **Cases/Participants** | | | 838 / 30683 | | | 1036 / 30658 | | | 730 / 30659 | | |  | |
|  |  | **HR (95%CI)** | | | 1.00 | | | 1.03 (0.94,1.13) | | | 0.98 (0.88,1.09) | | | p=0.97 | |
|  | **model 3** | **Cases/Participants** | | | 838 / 30683 | | | 1036 / 30658 | | | 730 / 30659 | | |  | |
|  |  | **HR (95%CI)** | | | 1.00 | | | 1.03 (0.94,1.13) | | | 0.98 (0.88,1.09) | | | p=0.98 | |
|  | **model 4** | **Cases/Participants** | | | 804 / 30664 | | | 1083 / 30667 | | | 717 / 30665 | | |  | |
|  |  | **HR (95%CI)** | | | 1.00 | | | 1.02 (0.92,1.12) | | | 1.05 (0.94,1.17) | | | p=0.4 | |
| **Mono- and diglycerides of FAs E471** | **model 1** | **Cases/Participants** | | | 790 / 30667 | | | 1018 / 30729 | | | 796 / 30604 | | |  | |
|  |  | **HR (95%CI)** | | | 1.00 | | | 1.16 (1.06,1.28) | | | 1.16 (1.05,1.29) | | | p=0.007 | |
|  | **model 2** | **Cases/Participants** | | | 790 / 30667 | | | 1018 / 30729 | | | 796 / 30604 | | |  | |
|  |  | **HR (95%CI)** | | | 1.00 | | | 1.16 (1.05,1.27) | | | 1.15 (1.04,1.28) | | | p=0.009 | |
|  | **model 3** | **Cases/Participants** | | | 790 / 30667 | | | 1018 / 30729 | | | 796 / 30604 | | |  | |
|  |  | **HR (95%CI)** | | | 1.00 | | | 1.16 (1.05,1.27) | | | 1.15 (1.04,1.27) | | | p=0.01 | |
|  | **model 4** | **Cases/Participants** | | | 773 / 30664 | | | 1005 / 30666 | | | 826 / 30666 | | |  | |
|  |  | **HR (95%CI)** | | | 1.00 | | | 1.09 (0.99,1.20) | | | 1.26 (1.14,1.40) | | | p<0.001 | |
| **Polyglycerol esters of FAs E475** | **model 1** | **Cases/Participants** | | | 2431 / 85557 | | | 103 / 3237 | | | 70 / 3206 | | |  | |
|  |  | **HR (95%CI)** | | | 1.00 | | | 1.09 (0.90,1.33) | | | 1.10 (0.86,1.40) | | | p=0.2 | |
|  | **model 2** | **Cases/Participants** | | | 2431 / 85557 | | | 103 / 3237 | | | 70 / 3206 | | |  | |
|  |  | **HR (95%CI)** | | | 1.00 | | | 1.09 (0.89,1.33) | | | 1.09 (0.86,1.39) | | | p=0.3 | |
|  | **model 3** | **Cases/Participants** | | | 2431 / 85557 | | | 103 / 3237 | | | 70 / 3206 | | |  | |
|  |  | **HR (95%CI)** | | | 1.00 | | | 1.09 (0.89,1.33) | | | 1.09 (0.86,1.39) | | | p=0.3 | |
|  | **model 4** | **Cases/Participants** | | | 2372 / 83451 | | | 110 / 4271 | | | 122 / 4274 | | |  | |
|  |  | **HR (95%CI)** | | | 1.00 | | | 0.53 (0.44,0.64) | | | 1.18 (0.99,1.42) | | | p=0.1 | |
| **Carob bean gum E410** | **model 1** | **Cases/Participants** | | | 1277 / 49966 | | | 747 / 21402 | | | 580 / 20632 | | |  | |
|  |  | **HR (95%CI)** | | | 1.00 | | | 1.06 (0.96,1.16) | | | 1.05 (0.95,1.16) | | | p=0.3 | |
|  | **model 2** | **Cases/Participants** | | | 1277 / 49966 | | | 747 / 21402 | | | 580 / 20632 | | |  | |
|  |  | **HR (95%CI)** | | | 1.00 | | | 1.06 (0.96,1.16) | | | 1.05 (0.95,1.16) | | | p=0.3 | |
|  | **model 3** | **Cases/Participants** | | | 1277 / 49966 | | | 747 / 21402 | | | 580 / 20632 | | |  | |
|  |  | **HR (95%CI)** | | | 1.00 | | | 1.05 (0.96,1.16) | | | 1.05 (0.95,1.16) | | | p=0.3 | |
|  | **model 4** | **Cases/Participants** | | | 815 / 37513 | | | 1017 / 27241 | | | 772 / 27242 | | |  | |
|  |  | **HR (95%CI)** | | | 1.00 | | | 0.86 (0.78,0.95) | | | 0.90 (0.81,1.00) | | | p=0.1 | |
| **Guar gum E412** | **model 1** | **Cases/Participants** | | | 733 / 30693 | | | 996 / 30640 | | | 875 / 30667 | | |  | |
|  |  | **HR (95%CI)** | | | 1.00 | | | 1.12 (1.01,1.23) | | | 1.09 (0.98,1.21) | | | p=0.1 | |
|  | **model 2** | **Cases/Participants** | | | 733 / 30693 | | | 996 / 30640 | | | 875 / 30667 | | |  | |
|  |  | **HR (95%CI)** | | | 1.00 | | | 1.12 (1.01,1.23) | | | 1.08 (0.98,1.20) | | | p=0.1 | |
|  | **model 3** | **Cases/Participants** | | | 733 / 30693 | | | 996 / 30640 | | | 875 / 30667 | | |  | |
|  |  | **HR (95%CI)** | | | 1.00 | | | 1.11 (1.01,1.23) | | | 1.08 (0.97,1.19) | | | p=0.1 | |
|  | **model 4** | **Cases/Participants** | | | 694 / 30671 | | | 1034 / 30692 | | | 876 / 30633 | | |  | |
|  |  | **HR (95%CI)** | | | 1.00 | | | 0.95 (0.86,1.05) | | | 1.02 (0.92,1.13) | | | p=0.6 | |
| **Gum arabic E414** | **model 1** | **Cases/Participants** | | | 2342 / 82111 | | | 151 / 5015 | | | 111 / 4874 | | |  | |
|  |  | **HR (95%CI)** | | | 1.00 | | | 1.10 (0.93,1.30) | | | 1.08 (0.89,1.31) | | | p=0.2 | |
|  | **model 2** | **Cases/Participants** | | | 2342 / 82111 | | | 151 / 5015 | | | 111 / 4874 | | |  | |
|  |  | **HR (95%CI)** | | | 1.00 | | | 1.10 (0.93,1.30) | | | 1.08 (0.89,1.30) | | | p=0.2 | |
|  | **model 3** | **Cases/Participants** | | | 2342 / 82111 | | | 151 / 5015 | | | 111 / 4874 | | |  | |
|  |  | **HR (95%CI)** | | | 1.00 | | | 1.10 (0.93,1.30) | | | 1.07 (0.88,1.30) | | | p=0.3 | |
|  | **model 4** | **Cases/Participants** | | | 2108 / 74103 | | | 273 / 8947 | | | 223 / 8946 | | |  | |
|  |  | **HR (95%CI)** | | | 1.00 | | | 0.67 (0.59,0.76) | | | 0.94 (0.81,1.08) | | | p<0.001 | |
| **Gum Xanthan Gum E415** | **model 1** | **Cases/Participants** | | | 735 / 30670 | | | 928 / 30663 | | | 941 / 30667 | | |  | |
|  |  | **HR (95%CI)** | | | 1.00 | | | 1.13 (1.02,1.25) | | | 1.14 (1.03,1.26) | | | p=0.02 | |
|  | **model 2** | **Cases/Participants** | | | 735 / 30670 | | | 928 / 30663 | | | 941 / 30667 | | |  | |
|  |  | **HR (95%CI)** | | | 1.00 | | | 1.13 (1.02,1.24) | | | 1.13 (1.02,1.25) | | | p=0.03 | |
|  | **model 3** | **Cases/Participants** | | | 735 / 30670 | | | 928 / 30663 | | | 941 / 30667 | | |  | |
|  |  | **HR (95%CI)** | | | 1.00 | | | 1.12 (1.02,1.24) | | | 1.13 (1.02,1.25) | | | p=0.04 | |
|  | **model 4** | **Cases/Participants** | | | 682 / 30664 | | | 1015 / 30666 | | | 907 / 30666 | | |  | |
|  |  | **HR (95%CI)** | | | 1.00 | | | 0.97 (0.88,1.07) | | | 0.96 (0.86,1.06) | | | p=0.4 | |
| **Pectins E440** | **model 1** | **Cases/Participants** | | | 657 / 30676 | | | 985 / 30963 | | | 962 / 30361 | | |  | |
|  |  | **HR (95%CI)** | | | 1.00 | | | 1.09 (0.99,1.21) | | | 1.09 (0.98,1.21) | | | p=0.3 | |
|  | **model 2** | **Cases/Participants** | | | 657 / 30676 | | | 985 / 30963 | | | 962 / 30361 | | |  | |
|  |  | **HR (95%CI)** | | | 1.00 | | | 1.09 (0.99,1.21) | | | 1.08 (0.97,1.20) | | | p=0.4 | |
|  | **model 3** | **Cases/Participants** | | | 657 / 30676 | | | 985 / 30963 | | | 962 / 30361 | | |  | |
|  |  | **HR (95%CI)** | | | 1.00 | | | 1.09 (0.99,1.21) | | | 1.07 (0.97,1.19) | | | p=0.4 | |
|  | **model 4** | **Cases/Participants** | | | 634 / 30664 | | | 992 / 30666 | | | 978 / 30666 | | |  | |
|  |  | **HR (95%CI)** | | | 1.00 | | | 1.08 (0.98,1.20) | | | 1.13 (1.02,1.26) | | | p=0.02 | |
| **Sodium bicarbonate E500** | **model 1** | **Cases/Participants** | | | 841 / 30705 | | | 978 / 30629 | | | 785 / 30666 | | |  | |
|  |  | **HR (95%CI)** | | | 1.00 | | | 1.02 (0.93,1.12) | | | 1.00 (0.90,1.11) | | | p=0.8 | |
|  | **model 2** | **Cases/Participants** | | | 841 / 30705 | | | 978 / 30629 | | | 785 / 30666 | | |  | |
|  |  | **HR (95%CI)** | | | 1.00 | | | 1.02 (0.93,1.12) | | | 1.00 (0.90,1.11) | | | p=0.7 | |
|  | **model 3** | **Cases/Participants** | | | 841 / 30705 | | | 978 / 30629 | | | 785 / 30666 | | |  | |
|  |  | **HR (95%CI)** | | | 1.00 | | | 1.02 (0.93,1.12) | | | 1.00 (0.90,1.11) | | | p=0.8 | |
|  | **model 4** | **Cases/Participants** | | | 780 / 30664 | | | 1074 / 30666 | | | 750 / 30666 | | |  | |
|  |  | **HR (95%CI)** | | | 1.00 | | | 0.97 (0.89,1.09) | | | 0.99 (0.89,1.10) | | | p=0.9 | |
| **Beeswax E901** | **model 1** | **Cases/Participants** | | | 2465 / 86421 | | | 76 / 2802 | | | 63 / 2777 | | |  | |
|  |  | **HR (95%CI)** | | | 1.00 | | | 0.93 (0.74,1.18) | | | 1.18 (0.91,1.52) | | | p=0.5 | |
|  | **model 2** | **Cases/Participants** | | | 2465 / 86421 | | | 76 / 2802 | | | 63 / 2777 | | |  | |
|  |  | **HR (95%CI)** | | | 1.00 | | | 0.93 (0.74,1.17) | | | 1.18 (0.91,1.52) | | | p=0.5 | |
|  | **model 3** | **Cases/Participants** | | | 2465 / 86421 | | | 76 / 2802 | | | 63 / 2777 | | |  | |
|  |  | **HR (95%CI)** | | | 1.00 | | | 0.93 (0.74,1.18) | | | 1.18 (0.91,1.51) | | | p=0.5 | |
|  | **model 4** | **Cases/Participants** | | | 2387 / 83703 | | | 138 / 4140 | | | 79 /4153 | | |  | |
|  |  | **HR (95%CI)** | | | 1.00 | | | 0.88 (0.74,1.05) | | | 0.93 (0.74,1.17) | | | p=0.2 | |
| **Overall breast cancer** |  |  | | |  | | |  | | |  | | |  | |
| **Total carrageenans** | **model 1** | **Cases/Participants** | | | 192 / 24107 | | | 283 / 24075 | | | 273 / 24088 | | |  | |
|  |  | **HR (95%CI)** | | | 1.00 | | | 1.18 (0.98,1.43) | | | 1.34 (1.10,1.63) | | | p=0.006 | |
|  | **model 2** | **Cases/Participants** | | | 192 / 24107 | | | 283 / 24075 | | | 273 / 24088 | | |  | |
|  |  | **HR (95%CI)** | | | 1.00 | | | 1.18 (0.97,1.42) | | | 1.33 (1.09,1.62) | | | p=0.007 | |
|  | **model 3** | **Cases/Participants** | | | 192 / 24107 | | | 283 / 24075 | | | 273 / 24088 | | |  | |
|  |  | **HR (95%CI)** | | | 1.00 | | | 1.18 (0.97,1.42) | | | 1.33 (1.09,1.61) | | | p=0.008 | |
|  | **model 4** | **Cases/Participants** | | | 189 / 24088 | | | 286 / 24089 | | | 273 / 24089 | | |  | |
|  |  | **HR (95%CI)** | | | 1.00 | | | 1.08 (0.89,1.30) | | | 1.37 (1.12,1.66) | | | p<0.001 | |
| **Carrageenan E407** | **model 1** | **Cases/Participants** | | | 194 / 24090 | | | 283 / 24116 | | | 271 / 24064 | | |  | |
|  |  | **HR (95%CI)** | | | 1.00 | | | 1.15 (0.96,1.39) | | | 1.30 (1.07,1.59) | | | p=0.009 | |
|  | **model 2** | **Cases/Participants** | | | 194 / 24090 | | | 283 / 24116 | | | 271 / 24064 | | |  | |
|  |  | **HR (95%CI)** | | | 1.00 | | | 1.15 (0.95,1.39) | | | 1.30 (1.07,1.58) | | | p=0.01 | |
|  | **model 3** | **Cases/Participants** | | | 194 / 24090 | | | 283 / 24116 | | | 271 / 24064 | | |  | |
|  |  | **HR (95%CI)** | | | 1.00 | | | 1.15 (0.95,1.39) | | | 1.29 (1.06,1.57) | | | p=0.01 | |
|  | **model 4** | **Cases/Participants** | | | 187 / 24088 | | | 293 / 24089 | | | 268 / 24089 | | |  | |
|  |  | **HR (95%CI)** | | | 1.00 | | | 1.10 (0.91,1.33) | | | 1.35 (1.11,1.16) | | | p=0.003 | |
| **Tripotassium phosphate E340** | **model 1** | **Cases/Participants** | | | 700 / 67935 | | | 20 / 2184 | | | 28 / 2151 | | |  | |
|  |  | **HR (95%CI)** | | | 1.00 | | | 0.77 (0.49,1.20) | | | 1.16 (0.79,1.70) | | | p=0.7 | |
|  | **model 2** | **Cases/Participants** | | | 700 / 67935 | | | 20 / 2184 | | | 28 / 2151 | | |  | |
|  |  | **HR (95%CI)** | | | 1.00 | | | 0.77 (0.49,1.20) | | | 1.16 (0.79,1.70) | | | p=0.7 | |
|  | **model 3** | **Cases/Participants** | | | 700 / 67935 | | | 20 / 2184 | | | 28 / 2151 | | |  | |
|  |  | **HR (95%CI)** | | | 1.00 | | | 0.77 (0.49,1.20) | | | 1.16 (0.80,1.71) | | | p=0.7 | |
|  | **model 4** | **Cases/Participants** | | | 666 / 65023 | | | 36 / 3620 | | | 46 / 3623 | | |  | |
|  |  | **HR (95%CI)** | | | 1.00 | | | 0.59 (0.42,0.82) | | | 0.96 (0.71,1.30) | | | p=0.1 | |
| **Diphosphates E450** | **model 1** | **Cases/Participants** | | | 207 / 24101 | | | 312 / 24084 | | | 229 / 24085 | | |  | |
|  |  | **HR (95%CI)** | | | 1.00 | | | 1.25 (1.04,1.51) | | | 1.18 (0.94,1.48) | | | p=0.04 | |
|  | **model 2** | **Cases/Participants** | | | 207 / 24101 | | | 312 / 24084 | | | 229 / 24085 | | |  | |
|  |  | **HR (95%CI)** | | | 1.00 | | | 1.24 (1.03,1.49) | | | 1.15 (0.94,1.40) | | | p=0.1 | |
|  | **model 3** | **Cases/Participants** | | | 207 / 24101 | | | 312 / 24084 | | | 229 / 24085 | | |  | |
|  |  | **HR (95%CI)** | | | 1.00 | | | 1.24 (1.03,1.49) | | | 1.15 (0.94,1.40) | | | p=0.1 | |
|  | **model 4** | **Cases/Participants** | | | 196 / 24088 | | | 331 / 24089 | | | 221 / 24089 | | |  | |
|  |  | **HR (95%CI)** | | | 1.00 | | | 1.25 (1.04,1.50) | | | 1.21 (0.99,1.49) | | | p=0.1 | |
| **Mono- and diglycerides of FAs E471** | **model 1** | **Cases/Participants** | | | 211 / 24090 | | | 292 / 24153 | | | 245 / 24027 | | |  | |
|  |  | **HR (95%CI)** | | | 1.00 | | | 1.21 (1.01,1.45) | | | 1.26 (1.04,1.53) | | | p=0.03 | |
|  | **model 2** | **Cases/Participants** | | | 211 / 24090 | | | 292 / 24153 | | | 245 / 24027 | | |  | |
|  |  | **HR (95%CI)** | | | 1.00 | | | 1.20 (1.00,1.44) | | | 1.25 (1.03,1.52) | | | p=0.03 | |
|  | **model 3** | **Cases/Participants** | | | 211 / 24090 | | | 292 / 24153 | | | 245 / 24027 | | |  | |
|  |  | **HR (95%CI)** | | | 1.00 | | | 1.20 (1.00,1.44) | | | 1.25 (1.03,1.51) | | | p=0.03 | |
|  | **model 4** | **Cases/Participants** | | | 190 / 24088 | | | 307 / 24089 | | | 251 / 24089 | | |  | |
|  |  | **HR (95%CI)** | | | 1.00 | | | 1.32 (1.10,1.59) | | | 1.50 (1.23,1.83) | | | p<0.001 | |
| **Polyglycerol esters of FAs E475** | **model 1** | **Cases/Participants** | | | 683 / 67018 | | | 33 / 2626 | | | 32 / 2626 | | |  | |
|  |  | **HR (95%CI)** | | | 1.00 | | | 1.10 (0.77,1.57) | | | 1.51 (1.06,2.17) | | | p=0.02 | |
|  | **model 2** | **Cases/Participants** | | | 683 / 67018 | | | 33 / 2626 | | | 32 / 2626 | | |  | |
|  |  | **HR (95%CI)** | | | 1.00 | | | 1.10 (0.77,1.57) | | | 1.50 (1.05,2.15) | | | p=0.02 | |
|  | **model 3** | **Cases/Participants** | | | 683 / 67018 | | | 33 / 2626 | | | 32 / 2626 | | |  | |
|  |  | **HR (95%CI)** | | | 1.00 | | | 1.10 (0.77,1.56) | | | 1.50 (1.05,2.15) | | | p=0.02 | |
|  | **model 4** | **Cases/Participants** | | | 673 / 65364 | | | 33 / 3450 | | | 42 / 3452 | | |  | |
|  |  | **HR (95%CI)** | | | 1.00 | | | 0.50 (0.35,0.72) | | | 1.24 (0.91,1.71) | | | p=0.4 | |
| **Carob bean gum E410** | **model 1** | **Cases/Participants** | | | 330 / 38896 | | | 233 / 17072 | | | 185 / 16302 | | |  | |
|  |  | **HR (95%CI)** | | | 1.00 | | | 1.21 (1.01,1.44) | | | 1.19 (0.99,1.43) | | | p=0.04 | |
|  | **model 2** | **Cases/Participants** | | | 330 / 38896 | | | 233 / 17072 | | | 185 / 16302 | | |  | |
|  |  | **HR (95%CI)** | | | 1.00 | | | 1.21 (1.01,1.44) | | | 1.19 (0.99,1.43) | | | p=0.04 | |
|  | **model 3** | **Cases/Participants** | | | 330 / 38896 | | | 233 / 17072 | | | 185 / 16302 | | |  | |
|  |  | **HR (95%CI)** | | | 1.00 | | | 1.21 (1.01,1.44) | | | 1.19 (0.99,1.43) | | | p=0.04 | |
|  | **model 4** | **Cases/Participants** | | | 211 / 29368 | | | 282 / 21449 | | | 255 / 21449 | | |  | |
|  |  | **HR (95%CI)** | | | 1.00 | | | 0.91 (0.75,1.11) | | | 1.09 (0.90,1.33) | | | p=0.3 | |
| **Guar gum E412** | **model 1** | **Cases/Participants** | | | 208 / 24116 | | | 290 / 24064 | | | 250 / 24090 | | |  | |
|  |  | **HR (95%CI)** | | | 1.00 | | | 1.11 (0.92,1.34) | | | 1.03 (0.85,1.24) | | | p=0.6 | |
|  | **model 2** | **Cases/Participants** | | | 208 / 24116 | | | 290 / 24064 | | | 250 / 24090 | | |  | |
|  |  | **HR (95%CI)** | | | 1.00 | | | 1.11 (0.92,1.34) | | | 1.02 (0.85,1.24) | | | p=0.6 | |
|  | **model 3** | **Cases/Participants** | | | 208 / 24116 | | | 290 / 24064 | | | 250 / 24090 | | |  | |
|  |  | **HR (95%CI)** | | | 1.00 | | | 1.11 (0.92,1.34) | | | 1.02 (0.85,1.23) | | | p=0.6 | |
|  | **model 4** | **Cases/Participants** | | | 202 / 24095 | | | 288 / 24115 | | | 258 / 24056 | | |  | |
|  |  | **HR (95%CI)** | | | 1.00 | | | 1.07 (0.92,1.24) | | | 1.09 (0.73,1.64) | | | p=0.3 | |
| **Gum arabic E414** | **model 1** | **Cases/Participants** | | | 653 / 63902 | | | 57 / 4254 | | | 38 / 4114 | | |  | |
|  |  | **HR (95%CI)** | | | 1.00 | | | 1.22 (0.93,1.61) | | | 1.03 (0.74,1.44) | | | p=0.4 | |
|  | **model 2** | **Cases/Participants** | | | 653 / 63902 | | | 57 / 4254 | | | 38 / 4114 | | |  | |
|  |  | **HR (95%CI)** | | | 1.00 | | | 1.22 (0.93,1.61) | | | 1.03 (0.74,1.44) | | | p=0.4 | |
|  | **model 3** | **Cases/Participants** | | | 653 / 63902 | | | 57 / 4254 | | | 38 / 4114 | | |  | |
|  |  | **HR (95%CI)** | | | 1.00 | | | 1.23 (0.93,1.62) | | | 1.04 (0.75,1.45) | | | p=0.4 | |
|  | **model 4** | **Cases/Participants** | | | 591 / 57666 | | | 94 / 7300 | | | 63 / 7300 | | |  | |
|  |  | **HR (95%CI)** | | | 1.00 | | | 0.73 (0.58,0.91) | | | 0.79 (0.61,1.04) | | | p=0.009 | |
| **Gum Xanthan Gum E415** | **model 1** | **Cases/Participants** | | | 196 / 24093 | | | 276 / 24087 | | | 276 / 24090 | | |  | |
|  |  | **HR (95%CI)** | | | 1.00 | | | 1.21 (1.01,1.47) | | | 1.17 (0.96,1.42) | | | p=0.2 | |
|  | **model 2** | **Cases/Participants** | | | 196 / 24093 | | | 276 / 24087 | | | 276 / 24090 | | |  | |
|  |  | **HR (95%CI)** | | | 1.00 | | | 1.21 (1.00,1.46) | | | 1.16 (0.96,1.41) | | | p=0.2 | |
|  | **model 3** | **Cases/Participants** | | | 196 / 24093 | | | 276 / 24087 | | | 276 / 24090 | | |  | |
|  |  | **HR (95%CI)** | | | 1.00 | | | 1.21 (1.00,1.46) | | | 1.16 (0.96,1.40) | | | p=0.2 | |
|  | **model 4** | **Cases/Participants** | | | 189 / 24088 | | | 299 / 24089 | | | 260 / 24089 | | |  | |
|  |  | **HR (95%CI)** | | | 1.00 | | | 1.02 (0.84,1.23) | | | 0.92 (0.76,1.13) | | | p=0.4 | |
| **Pectins E440** | **model 1** | **Cases/Participants** | | | 172 / 24095 | | | 281 / 24389 | | | 295 / 23786 | | |  | |
|  |  | **HR (95%CI)** | | | 1.00 | | | 1.12 (0.92,1.37) | | | 1.17 (0.96,1.44) | | | p=0.1 | |
|  | **model 2** | **Cases/Participants** | | | 172 / 24095 | | | 281 / 24389 | | | 295 / 23786 | | |  | |
|  |  | **HR (95%CI)** | | | 1.00 | | | 1.12 (0.92,1.36) | | | 1.16 (0.95,1.42) | | | p=0.2 | |
|  | **model 3** | **Cases/Participants** | | | 172 / 24095 | | | 281 / 24389 | | | 295 / 23786 | | |  | |
|  |  | **HR (95%CI)** | | | 1.00 | | | 1.12 (0.92,1.36) | | | 1.16 (0.95,1.41) | | | p=0.2 | |
|  | **model 4** | **Cases/Participants** | | | 178 / 24088 | | | 265 / 24089 | | | 305 / 24089 | | |  | |
|  |  | **HR (95%CI)** | | | 1.00 | | | 0.95 (0.78,1.15) | | | 1.13 (0.93,1.38) | | | p=0.1 | |
| **Sodium bicarbonate E500** | **model 1** | **Cases/Participants** | | | 214 / 24118 | | | 293 / 24062 | | | 241 / 24090 | | |  | |
|  |  | **HR (95%CI)** | | | 1.00 | | | 1.16 (0.96,1.39) | | | 1.09 (0.89,1.33) | | | p=0.3 | |
|  | **model 2** | **Cases/Participants** | | | 214 / 24118 | | | 293 / 24062 | | | 241 / 24090 | | |  | |
|  |  | **HR (95%CI)** | | | 1.00 | | | 1.16 (0.97,1.40) | | | 1.09 (0.90,1.33) | | | p=0.3 | |
|  | **model 3** | **Cases/Participants** | | | 214 / 24118 | | | 293 / 24062 | | | 241 / 24090 | | |  | |
|  |  | **HR (95%CI)** | | | 1.00 | | | 1.16 (0.97,1.40) | | | 1.09 (0.90,1.33) | | | p=0.3 | |
|  | **model 4** | **Cases/Participants** | | | 203 / 24088 | | | 314 / 24089 | | | 231 / 24089 | | |  | |
|  |  | **HR (95%CI)** | | | 1.00 | | | 1.09 (0.90,1.31) | | | 1.06 (0.87,1.30) | | | p=0.6 | |
| **Beeswax E901** | **model 1** | **Cases/Participants** | | | 692 / 67515 | | | 33 / 2383 | | | 23 / 2372 | | |  | |
|  |  | **HR (95%CI)** | | | 1.00 | | | 1.13 (0.79,1.61) | | | 1.18 (0.78,1.80) | | | p=0.3 | |
|  | **model 2** | **Cases/Participants** | | | 692 / 67515 | | | 33 / 2383 | | | 23 / 2372 | | |  | |
|  |  | **HR (95%CI)** | | | 1.00 | | | 1.13 (0.79,1.61) | | | 1.18 (0.77,1.80) | | | p=0.3 | |
|  | **model 3** | **Cases/Participants** | | | 692 / 67515 | | | 33 / 2383 | | | 23 / 2372 | | |  | |
|  |  | **HR (95%CI)** | | | 1.00 | | | 1.13 (0.80,1.61) | | | 1.18 (0.78,1.80) | | | p=0.3 | |
|  | **model 4** | **Cases/Participants** | | | 672 / 65277 | | | 52 / 3488 | | | 24 / 3501 | | |  | |
|  |  | **HR (95%CI)** | | | 1.00 | | | 0.95 (0.71,1.26) | | | 0.80 (0.53,1.21) | | | p=0.3 | |
| **Premenopausal breast cancer** | | |  |  | |  | | |  | | |  | |  | |
| **Total carrageenans** | **model 1** | **Cases/Participants** | | | 64 / 17077 | | | 117 / 17189 | | | 113 / 18697 | | |  | |
|  |  | **HR (95%CI)** | | | 1.00 | | | 1.43 (1.04,1.96) | | | 1.38 (1.00,1.91) | | | p=0.1 | |
|  | **model 2** | **Cases/Participants** | | | 64 / 17077 | | | 117 / 17189 | | | 113 / 18697 | | |  | |
|  |  | **HR (95%CI)** | | | 1.00 | | | 1.43 (1.04,1.96) | | | 1.38 (1.00,1.91) | | | p=0.1 | |
|  | **model 3** | **Cases/Participants** | | | 64 / 17077 | | | 117 / 17189 | | | 113 / 18697 | | |  | |
|  |  | **HR (95%CI)** | | | 1.00 | | | 1.43 (1.04,1.96) | | | 1.38 (1.00,1.91) | | | p=0.1 | |
|  | **model 4** | **Cases/Participants** | | | 59 / 17136 | | | 113 / 16921 | | | 123 / 18901 | | |  | |
|  |  | **HR (95%CI)** | | | 1.00 | | | 1.43 (1.03,1.97) | | | 1.67 (1.21,2.31) | | | p=0.002 | |
| **Carrageenan E407** | **model 1** | **Cases/Participants** | | | 65 / 17093 | | | 117 / 17230 | | | 112 / 18640 | | |  | |
|  |  | **HR (95%CI)** | | | 1.00 | | | 1.40 (1.02,1.92) | | | 1.36 (0.98,1.88) | | | p=0.1 | |
|  | **model 2** | **Cases/Participants** | | | 65 / 17093 | | | 117 / 17230 | | | 112 / 18640 | | |  | |
|  |  | **HR (95%CI)** | | | 1.00 | | | 1.40 (1.02,1.91) | | | 1.36 (0.98,1.87) | | | p=0.1 | |
|  | **model 3** | **Cases/Participants** | | | 65 / 17093 | | | 117 / 17230 | | | 112 / 18640 | | |  | |
|  |  | **HR (95%CI)** | | | 1.00 | | | 1.40 (1.02,1.92) | | | 1.36 (0.98,1.87) | | | p=0.1 | |
|  | **model 4** | **Cases/Participants** | | | 58 / 17176 | | | 115 / 16921 | | | 122 / 18861 | | |  | |
|  |  | **HR (95%CI)** | | | 1.00 | | | 1.47 (1.06,2.04) | | | 1.70 (1.23,2.35) | | | p=0.002 | |
| **Tripotassium phosphate E340** | **model 1** | **Cases/Participants** | | | 267 / 49714 | | | 10 / 1657 | | | 17 / 1592 | | |  | |
|  |  | **HR (95%CI)** | | | 1.00 | | | 0.99 (0.52,1.86) | | | 1.80 (1.09,2.96) | | | p=0.03 | |
|  | **model 2** | **Cases/Participants** | | | 267 / 49714 | | | 10 / 1657 | | | 17 / 1592 | | |  | |
|  |  | **HR (95%CI)** | | | 1.00 | | | 0.99 (0.52,1.86) | | | 1.80 (1.09,2.95) | | | p=0.03 | |
|  | **model 3** | **Cases/Participants** | | | 267 / 49714 | | | 10 / 1657 | | | 17 / 1592 | | |  | |
|  |  | **HR (95%CI)** | | | 1.00 | | | 0.99 (0.52,1.86) | | | 1.82 (1.11,2.99) | | | p=0.03 | |
|  | **model 4** | **Cases/Participants** | | | 261 / 47843 | | | 15 / 2482 | | | 19 / 2633 | | |  | |
|  |  | **HR (95%CI)** | | | 1.00 | | | 0.72 (0.42,1.22) | | | 1.06 (0.66,1.70) | | | p=0.8 | |
| **Diphosphates E450** | **model 1** | **Cases/Participants** | | | 61 / 16560 | | | 120 / 17021 | | | 113 / 19382 | | |  | |
|  |  | **HR (95%CI)** | | | 1.00 | | | 1.58 (1.14,2.18) | | | 1.50 (1.04,2.16) | | | p=0.02 | |
|  | **model 2** | **Cases/Participants** | | | 61 / 16560 | | | 120 / 17021 | | | 113 / 19382 | | |  | |
|  |  | **HR (95%CI)** | | | 1.00 | | | 1.56 (1.13,2.15) | | | 1.45 (1.04,2.02) | | | p=0.03 | |
|  | **model 3** | **Cases/Participants** | | | 61 / 16560 | | | 120 / 17021 | | | 113 / 19382 | | |  | |
|  |  | **HR (95%CI)** | | | 1.00 | | | 1.56 (1.13,2.15) | | | 1.46 (1.05,2.02) | | | p=0.03 | |
|  | **model 4** | **Cases/Participants** | | | 54 / 16494 | | | 119 / 16726 | | | 122 / 19738 | | |  | |
|  |  | **HR (95%CI)** | | | 1.00 | | | 1.65 (1.18,2.30) | | | 1.78 (1.27,2.50) | | | p=0.002 | |
| **Mono- and diglycerides of FAs E471** | **model 1** | **Cases/Participants** | | | 73 / 16654 | | | 110 / 17284 | | | 111 / 19025 | | |  | |
|  |  | **HR (95%CI)** | | | 1.00 | | | 1.24 (0.91,1.68) | | | 1.30 (0.95,1.78) | | | p=0.1 | |
|  | **model 2** | **Cases/Participants** | | | 73 / 16654 | | | 110 / 17284 | | | 111 / 19025 | | |  | |
|  |  | **HR (95%CI)** | | | 1.00 | | | 1.24 (0.91,1.68) | | | 1.30 (0.96,1.78) | | | p=0.1 | |
|  | **model 3** | **Cases/Participants** | | | 73 / 16654 | | | 110 / 17284 | | | 111 / 19025 | | |  | |
|  |  | **HR (95%CI)** | | | 1.00 | | | 1.23 (0.91,1.67) | | | 1.30 (0.95,1.77) | | | p=0.1 | |
|  | **model 4** | **Cases/Participants** | | | 60 / 16526 | | | 113 / 17039 | | | 122 / 19393 | | |  | |
|  |  | **HR (95%CI)** | | | 1.00 | | | 1.51 (1.10,2.09) | | | 1.78 (1.28,2.45) | | | p<0.001 | |
| **Polyglycerol esters of FAs E475** | **model 1** | **Cases/Participants** | | | 262 / 48656 | | | 15 / 2065 | | | 17 / 2242 | | |  | |
|  |  | **HR (95%CI)** | | | 1.00 | | | 1.11 (0.66,1.88) | | | 1.50 (0.91,2.47) | | | p=0.1 | |
|  | **model 2** | **Cases/Participants** | | | 262 / 48656 | | | 15 / 2065 | | | 17 / 2242 | | |  | |
|  |  | **HR (95%CI)** | | | 1.00 | | | 1.11 (0.66,1.88) | | | 1.50 (0.92,2.47) | | | p=0.1 | |
|  | **model 3** | **Cases/Participants** | | | 262 / 48656 | | | 15 / 2065 | | | 17 / 2242 | | |  | |
|  |  | **HR (95%CI)** | | | 1.00 | | | 1.11 (0.66,1.88) | | | 1.51 (0.92,2.47) | | | p=0.1 | |
|  | **model 4** | **Cases/Participants** | | | 254 / 47731 | | | 18 / 2329 | | | 23 / 2898 | | |  | |
|  |  | **HR (95%CI)** | | | 1.00 | | | 0.82 (0.50,1.33) | | | 1.36 (0.89,2.10) | | | p=0.4 | |
| **Carob bean gum E410** | **model 1** | **Cases/Participants** | | | 125 / 28265 | | | 100 / 12254 | | | 69 / 12444 | | |  | |
|  |  | **HR (95%CI)** | | | 1.00 | | | 1.41 (1.07,1.86) | | | 1.07 (0.79,1.45) | | | p=0.5 | |
|  | **model 2** | **Cases/Participants** | | | 125 / 28265 | | | 100 / 12254 | | | 69 / 12444 | | |  | |
|  |  | **HR (95%CI)** | | | 1.00 | | | 1.41 (1.07,1.86) | | | 1.07 (0.79,1.45) | | | p=0.5 | |
|  | **model 3** | **Cases/Participants** | | | 125 / 28265 | | | 100 / 12254 | | | 69 / 12444 | | |  | |
|  |  | **HR (95%CI)** | | | 1.00 | | | 1.41 (1.07,1.86) | | | 1.07 (0.79,1.45) | | | p=0.4 | |
|  | **model 4** | **Cases/Participants** | | | 79 / 22194 | | | 107 / 14495 | | | 109 / 16269 | | |  | |
|  |  | **HR (95%CI)** | | | 1.00 | | | 1.26 (0.92,1.73) | | | 1.38 (1.01,1.88) | | | p=0.046 | |
| **Guar gum E412** | **model 1** | **Cases/Participants** | | | 79 / 17423 | | | 122 / 17568 | | | 93 / 17972 | | |  | |
|  |  | **HR (95%CI)** | | | 1.00 | | | 1.21 (0.90,1.63) | | | 0.94 (0.69,1.28) | | | p=0.8 | |
|  | **model 2** | **Cases/Participants** | | | 79 / 17423 | | | 122 / 17568 | | | 93 / 17972 | | |  | |
|  |  | **HR (95%CI)** | | | 1.00 | | | 1.22 (0.90,1.63) | | | 0.95 (0.70,1.29) | | | p=0.9 | |
|  | **model 3** | **Cases/Participants** | | | 79 / 17423 | | | 122 / 17568 | | | 93 / 17972 | | |  | |
|  |  | **HR (95%CI)** | | | 1.00 | | | 1.22 (0.91,1.64) | | | 0.95 (0.70,1.29) | | | p=0.9 | |
|  | **model 4** | **Cases/Participants** | | | 77 / 18010 | | | 111 / 16892 | | | 107 / 18056 | | |  | |
|  |  | **HR (95%CI)** | | | 1.00 | | | 0.99 (0.90,1.10) | | | 1.00 (0.78,1.27) | | | p=0.8 | |
| **Gum arabic E414** | **model 1** | **Cases/Participants** | | | 250 / 46093 | | | 24 / 3416 | | | 20 / 3454 | | |  | |
|  |  | **HR (95%CI)** | | | 1.00 | | | 1.04 (0.68,1.60) | | | 1.07 (0.67,1.70) | | | p=0.7 | |
|  | **model 2** | **Cases/Participants** | | | 250 / 46093 | | | 24 / 3416 | | | 20 / 3454 | | |  | |
|  |  | **HR (95%CI)** | | | 1.00 | | | 1.04 (0.68,1.60) | | | 1.07 (0.67,1.70) | | | p=0.7 | |
|  | **model 3** | **Cases/Participants** | | | 250 / 46093 | | | 24 / 3416 | | | 20 / 3454 | | |  | |
|  |  | **HR (95%CI)** | | | 1.00 | | | 1.05 (0.68,1.61) | | | 1.08 (0.68,1.72) | | | p=0.7 | |
|  | **model 4** | **Cases/Participants** | | | 225 / 41878 | | | 36 / 5069 | | | 34 / 6011 | | |  | |
|  |  | **HR (95%CI)** | | | 1.00 | | | 0.79 (0.55,1.14) | | | 0.90 (0.62,1.30) | | | p=0.4 | |
| **Gum Xanthan Gum E415** | **model 1** | **Cases/Participants** | | | 71 / 17423 | | | 119 / 17631 | | | 104 / 17909 | | |  | |
|  |  | **HR (95%CI)** | | | 1.00 | | | 1.46 (1.08,1.97) | | | 1.15 (0.84,1.58) | | | p=0.5 | |
|  | **model 2** | **Cases/Participants** | | | 71 / 17423 | | | 119 / 17631 | | | 104 / 17909 | | |  | |
|  |  | **HR (95%CI)** | | | 1.00 | | | 1.46 (1.08,1.97) | | | 1.15 (0.84,1.58) | | | p=0.5 | |
|  | **model 3** | **Cases/Participants** | | | 71 / 17423 | | | 119 / 17631 | | | 104 / 17909 | | |  | |
|  |  | **HR (95%CI)** | | | 1.00 | | | 1.46 (1.08,1.98) | | | 1.16 (0.84,1.58) | | | p=0.5 | |
|  | **model 4** | **Cases/Participants** | | | 71 / 18036 | | | 118 / 16979 | | | 106 / 17943 | | |  | |
|  |  | **HR (95%CI)** | | | 1.00 | | | 1.28 (0.95,1.75) | | | 1.07 (0.78,1.46) | | | p=0.9 | |
| **Pectins E440** | **model 1** | **Cases/Participants** | | | 60 / 18635 | | | 114 / 17402 | | | 120 / 16926 | | |  | |
|  |  | **HR (95%CI)** | | | 1.00 | | | 1.44 (1.05,1.99) | | | 1.57 (1.13,2.19) | | | p=0.006 | |
|  | **model 2** | **Cases/Participants** | | | 60 / 18635 | | | 114 / 17402 | | | 120 / 16926 | | |  | |
|  |  | **HR (95%CI)** | | | 1.00 | | | 1.44 (1.04,1.99) | | | 1.56 (1.12,2.15) | | | p=0.007 | |
|  | **model 3** | **Cases/Participants** | | | 60 / 18635 | | | 114 / 17402 | | | 120 / 16926 | | |  | |
|  |  | **HR (95%CI)** | | | 1.00 | | | 1.44 (1.04,1.99) | | | 1.56 (1.13,2.16) | | | p=0.007 | |
|  | **model 4** | **Cases/Participants** | | | 69 / 18725 | | | 100 / 17083 | | | 126 / 17150 | | |  | |
|  |  | **HR (95%CI)** | | | 1.00 | | | 1.08 (0.78,1.48) | | | 1.42 (1.04,1.93) | | | p=0.02 | |
| **Sodium bicarbonate E500** | **model 1** | **Cases/Participants** | | | 63 / 16630 | | | 113 / 17272 | | | 118 / 19061 | | |  | |
|  |  | **HR (95%CI)** | | | 1.00 | | | 1.47 (1.06,2.02) | | | 1.50 (1.08,2.09) | | | p=0.01 | |
|  | **model 2** | **Cases/Participants** | | | 63 / 16630 | | | 113 / 17272 | | | 118 / 19061 | | |  | |
|  |  | **HR (95%CI)** | | | 1.00 | | | 1.46 (1.06,2.01) | | | 1.48 (1.07,2.05) | | | p=0.01 | |
|  | **model 3** | **Cases/Participants** | | | 63 / 16630 | | | 113 / 17272 | | | 118 / 19061 | | |  | |
|  |  | **HR (95%CI)** | | | 1.00 | | | 1.46 (1.06,2.02) | | | 1.49 (1.07,2.06) | | | p=0.01 | |
|  | **model 4** | **Cases/Participants** | | | 62 / 16846 | | | 115 / 16727 | | | 118 / 19385 | | |  | |
|  |  | **HR (95%CI)** | | | 1.00 | | | 1.41 (1.02,1.95) | | | 1.45 (1.04,2.01) | | | p=0.043 | |
| **Beeswax E901** | **model 1** | **Cases/Participants** | | | 266 / 49020 | | | 11 / 1890 | | | 17 / 2053 | | |  | |
|  |  | **HR (95%CI)** | | | 1.00 | | | 0.78 (0.42,1.43) | | | 1.56 (0.95,2.58) | | | p=0.2 | |
|  | **model 2** | **Cases/Participants** | | | 266 / 49020 | | | 11 / 1890 | | | 17 / 2053 | | |  | |
|  |  | **HR (95%CI)** | | | 1.00 | | | 0.78 (0.42,1.43) | | | 1.56 (0.95,2.58) | | | p=0.2 | |
|  | **model 3** | **Cases/Participants** | | | 266 / 49020 | | | 11 / 1890 | | | 17 / 2053 | | |  | |
|  |  | **HR (95%CI)** | | | 1.00 | | | 0.78 (0.43,1.44) | | | 1.57 (0.95,2.59) | | | p=0.2 | |
|  | **model 4** | **Cases/Participants** | | | 259 / 47343 | | | 21 / 2577 | | | 15 / 3037 | | |  | |
|  |  | **HR (95%CI)** | | | 1.00 | | | 0.92 (0.58,1.44) | | | 0.86 (0.51,1.45) | | | p=0.5 | |
| **Postmenopausal breast cancer** |  |  | | |  | | |  | | |  | | |  | |
| **Total carrageenans** | **model 1** | **Cases/Participants** | | | 128 / 9458 | | | 166 / 9852 | | | 160 / 8120 | | |  | |
|  |  | **HR (95%CI)** | | | 1.00 | | | 1.04 (0.82,1.32) | | | 1.30 (1.02,1.68) | | | p=0.03 | |
|  | **model 2** | **Cases/Participants** | | | 128 / 9458 | | | 166 / 9852 | | | 160 / 8120 | | |  | |
|  |  | **HR (95%CI)** | | | 1.00 | | | 1.04 (0.82,1.32) | | | 1.29 (1.01,1.66) | | | p=0.04 | |
|  | **model 3** | **Cases/Participants** | | | 128 / 9458 | | | 166 / 9852 | | | 160 / 8120 | | |  | |
|  |  | **HR (95%CI)** | | | 1.00 | | | 1.04 (0.82,1.32) | | | 1.28 (1.00,1.64) | | | p=0.04 | |
|  | **model 4** | **Cases/Participants** | | | 130 / 9232 | | | 173 / 10438 | | | 150 / 7759 | | |  | |
|  |  | **HR (95%CI)** | | | 1.00 | | | 0.92 (0.73,1.16) | | | 1.12 (0.94,1.56) | | | p=0.1 | |
| **Carrageenan E407** | **model 1** | **Cases/Participants** | | | 129 / 9405 | | | 166 / 9866 | | | 159 / 8159 | | |  | |
|  |  | **HR (95%CI)** | | | 1.00 | | | 1.02 (0.81,1.30) | | | 1.27 (0.99,1.63) | | | p=0.043 | |
|  | **model 2** | **Cases/Participants** | | | 129 / 9405 | | | 166 / 9866 | | | 159 / 8159 | | |  | |
|  |  | **HR (95%CI)** | | | 1.00 | | | 1.02 (0.80,1.29) | | | 1.26 (0.98,1.61) | | | p=0.049 | |
|  | **model 3** | **Cases/Participants** | | | 129 / 9405 | | | 166 / 9866 | | | 159 / 8159 | | |  | |
|  |  | **HR (95%CI)** | | | 1.00 | | | 1.02 (0.80,1.29) | | | 1.25 (0.97,1.60) | | | p=0.1 | |
|  | **model 4** | **Cases/Participants** | | | 129 / 9178 | | | 178 / 10459 | | | 146 / 7792 | | |  | |
|  |  | **HR (95%CI)** | | | 1.00 | | | 0.94 (0.74,1.19) | | | 1.17 (0.91,1.50) | | | p=0.2 | |
| **Tripotassium phosphate E340** | **model 1** | **Cases/Participants** | | | 433 / 25795 | | | 10 / 801 | | | 11 / 834 | | |  | |
|  |  | **HR (95%CI)** | | | 1.00 | | | 0.62 (0.33,1.17) | | | 0.75 (0.41,1.37) | | | p=0.2 | |
|  | **model 2** | **Cases/Participants** | | | 433 / 25795 | | | 10 / 801 | | | 11 / 834 | | |  | |
|  |  | **HR (95%CI)** | | | 1.00 | | | 0.62 (0.33,1.17) | | | 0.75 (0.41,1.37) | | | p=0.2 | |
|  | **model 3** | **Cases/Participants** | | | 433 / 25795 | | | 10 / 801 | | | 11 / 834 | | |  | |
|  |  | **HR (95%CI)** | | | 1.00 | | | 0.62 (0.33,1.17) | | | 0.74 (0.41,1.36) | | | p=0.2 | |
|  | **model 4** | **Cases/Participants** | | | 405 / 24182 | | | 21 / 1748 | | | 27 / 1499 | | |  | |
|  |  | **HR (95%CI)** | | | 1.00 | | | 0.52 (0.33,0.81) | | | 0.90 (0.61,1.33) | | | p=0.08 | |
| **Diphosphates E450** | **model 1** | **Cases/Participants** | | | 146 / 10072 | | | 192 / 10149 | | | 116 / 7209 | | |  | |
|  |  | **HR (95%CI)** | | | 1.00 | | | 1.12 (0.89,1.40) | | | 1.02 (0.76,1.37) | | | p=0.5 | |
|  | **model 2** | **Cases/Participants** | | | 146 / 10072 | | | 192 / 10149 | | | 116 / 7209 | | |  | |
|  |  | **HR (95%CI)** | | | 1.00 | | | 1.11 (0.88,1.38) | | | 0.99 (0.77,1.29) | | | p=0.7 | |
|  | **model 3** | **Cases/Participants** | | | 146 / 10072 | | | 192 / 10149 | | | 116 / 7209 | | |  | |
|  |  | **HR (95%CI)** | | | 1.00 | | | 1.11 (0.88,1.38) | | | 0.99 (0.77,1.29) | | | p=0.7 | |
|  | **model 4** | **Cases/Participants** | | | 142 / 9966 | | | 212 / 10796 | | | 99 / 6667 | | |  | |
|  |  | **HR (95%CI)** | | | 1.00 | | | 1.08 (0.87,1.35) | | | 0.93 (0.70,1.22) | | | p=0.6 | |
| **Mono- and diglycerides of FAs E471** | **model 1** | **Cases/Participants** | | | 138 / 10053 | | | 182 / 9929 | | | 134 / 7448 | | |  | |
|  |  | **HR (95%CI)** | | | 1.00 | | | 1.18 (0.94,1.48) | | | 1.22 (0.95,1.57) | | | p=0.1 | |
|  | **model 2** | **Cases/Participants** | | | 138 / 10053 | | | 182 / 9929 | | | 134 / 7448 | | |  | |
|  |  | **HR (95%CI)** | | | 1.00 | | | 1.17 (0.93,1.47) | | | 1.21 (0.94,1.55) | | | p=0.2 | |
|  | **model 3** | **Cases/Participants** | | | 138 / 10053 | | | 182 / 9929 | | | 134 / 7448 | | |  | |
|  |  | **HR (95%CI)** | | | 1.00 | | | 1.17 (0.93,1.46) | | | 1.20 (0.94,1.54) | | | p=0.2 | |
|  | **model 4** | **Cases/Participants** | | | 130 / 10138 | | | 194 / 10286 | | | 129 / 7005 | | |  | |
|  |  | **HR (95%CI)** | | | 1.00 | | | 1.23 (0.98,1.54) | | | 1.35 (1.05,1.74) | | | p=0.02 | |
| **Polyglycerol esters of FAs E475** | **model 1** | **Cases/Participants** | | | 421 / 25917 | | | 18 / 891 | | | 15 / 622 | | |  | |
|  |  | **HR (95%CI)** | | | 1.00 | | | 1.08 (0.67,1.74) | | | 1.51 (0.90,2.55) | | | p=0.1 | |
|  | **model 2** | **Cases/Participants** | | | 421 / 25917 | | | 18 / 891 | | | 15 / 622 | | |  | |
|  |  | **HR (95%CI)** | | | 1.00 | | | 1.08 (0.67,1.73) | | | 1.49 (0.89,2.50) | | | p=0.1 | |
|  | **model 3** | **Cases/Participants** | | | 421 / 25917 | | | 18 / 891 | | | 15 / 622 | | |  | |
|  |  | **HR (95%CI)** | | | 1.00 | | | 1.08 (0.67,1.73) | | | 1.49 (0.89,2.51) | | | p=0.1 | |
|  | **model 4** | **Cases/Participants** | | | 419 / 24730 | | | 15 / 1782 | | | 19 / 917 | | |  | |
|  |  | **HR (95%CI)** | | | 1.00 | | | 0.34 (0.21,0.58) | | | 1.13 (0.71,1.80) | | | p=0.048 | |
| **Carob bean gum E410** | **model 1** | **Cases/Participants** | | | 205 / 14641 | | | 133 / 7062 | | | 116 / 5727 | | |  | |
|  |  | **HR (95%CI)** | | | 1.00 | | | 1.09 (0.87,1.37) | | | 1.27 (1.01,1.61) | | | p=0.047 | |
|  | **model 2** | **Cases/Participants** | | | 205 / 14641 | | | 133 / 7062 | | | 116 / 5727 | | |  | |
|  |  | **HR (95%CI)** | | | 1.00 | | | 1.09 (0.87,1.37) | | | 1.27 (1.01,1.61) | | | p=0.048 | |
|  | **model 3** | **Cases/Participants** | | | 205 / 14641 | | | 133 / 7062 | | | 116 / 5727 | | |  | |
|  |  | **HR (95%CI)** | | | 1.00 | | | 1.09 (0.87,1.37) | | | 1.27 (1.00,1.61) | | | p=0.1 | |
|  | **model 4** | **Cases/Participants** | | | 132 / 9567 | | | 175 / 10172 | | | 146 / 7690 | | |  | |
|  |  | **HR (95%CI)** | | | 1.00 | | | 0.75 (0.59,0.96) | | | 0.94 (0.73,1.20) | | | p=0.8 | |
| **Guar gum E412** | **model 1** | **Cases/Participants** | | | 129 / 9105 | | | 168 / 9363 | | | 157 / 8962 | | |  | |
|  |  | **HR (95%CI)** | | | 1.00 | | | 1.04 (0.82,1.32) | | | 1.08 (0.84,1.37) | | | p=0.5 | |
|  | **model 2** | **Cases/Participants** | | | 129 / 9105 | | | 168 / 9363 | | | 157 / 8962 | | |  | |
|  |  | **HR (95%CI)** | | | 1.00 | | | 1.04 (0.82,1.32) | | | 1.06 (0.84,1.35) | | | p=0.5 | |
|  | **model 3** | **Cases/Participants** | | | 129 / 9105 | | | 168 / 9363 | | | 157 / 8962 | | |  | |
|  |  | **HR (95%CI)** | | | 1.00 | | | 1.04 (0.82,1.31) | | | 1.06 (0.83,1.34) | | | p=0.5 | |
|  | **model 4** | **Cases/Participants** | | | 125 / 8226 | | | 177 / 10399 | | | 151 / 8804 | | |  | |
|  |  | **HR (95%CI)** | | | 1.00 | | | 1.05 (0.90,1.22) | | | 1.08 (0.72,1.62) | | | p=0.1 | |
| **Gum arabic E414** | **model 1** | **Cases/Participants** | | | 403 / 24979 | | | 33 / 1375 | | | 18 / 1076 | | |  | |
|  |  | **HR (95%CI)** | | | 1.00 | | | 1.36 (0.95,1.96) | | | 1.00 (0.62,1.60) | | | p=0.5 | |
|  | **model 2** | **Cases/Participants** | | | 403 / 24979 | | | 33 / 1375 | | | 18 / 1076 | | |  | |
|  |  | **HR (95%CI)** | | | 1.00 | | | 1.36 (0.95,1.95) | | | 0.99 (0.62,1.60) | | | p=0.5 | |
|  | **model 3** | **Cases/Participants** | | | 403 / 24979 | | | 33 / 1375 | | | 18 / 1076 | | |  | |
|  |  | **HR (95%CI)** | | | 1.00 | | | 1.36 (0.95,1.96) | | | 0.99 (0.62,1.60) | | | p=0.5 | |
|  | **model 4** | **Cases/Participants** | | | 366 / 21750 | | | 58 / 3577 | | | 29 / 2102 | | |  | |
|  |  | **HR (95%CI)** | | | 1.00 | | | 0.69 (0.52,0.92) | | | 0.70 (0.48,1.03) | | | p=0.007 | |
| **Gum Xanthan Gum E415** | **model 1** | **Cases/Participants** | | | 125 / 9111 | | | 157 / 9125 | | | 172 / 9194 | | |  | |
|  |  | **HR (95%CI)** | | | 1.00 | | | 1.06 (0.84,1.36) | | | 1.17 (0.92,1.50) | | | p=0.2 | |
|  | **model 2** | **Cases/Participants** | | | 125 / 9111 | | | 157 / 9125 | | | 172 / 9194 | | |  | |
|  |  | **HR (95%CI)** | | | 1.00 | | | 1.06 (0.83,1.35) | | | 1.16 (0.91,1.47) | | | p=0.3 | |
|  | **model 3** | **Cases/Participants** | | | 125 / 9111 | | | 157 / 9125 | | | 172 / 9194 | | |  | |
|  |  | **HR (95%CI)** | | | 1.00 | | | 1.06 (0.83,1.34) | | | 1.16 (0.91,1.47) | | | p=0.3 | |
|  | **model 4** | **Cases/Participants** | | | 118 / 8065 | | | 181 / 10094 | | | 154 / 9270 | | |  | |
|  |  | **HR (95%CI)** | | | 1.00 | | | 0.88 (0.70,1.12) | | | 0.84 (0.67,1.08) | | | p=0.2 | |
| **Pectins E440** | **model 1** | **Cases/Participants** | | | 112 / 7566 | | | 167 / 9897 | | | 175 / 9967 | | |  | |
|  |  | **HR (95%CI)** | | | 1.00 | | | 0.96 (0.75,1.23) | | | 0.97 (0.75,1.27) | | | p=0.7 | |
|  | **model 2** | **Cases/Participants** | | | 112 / 7566 | | | 167 / 9897 | | | 175 / 9967 | | |  | |
|  |  | **HR (95%CI)** | | | 1.00 | | | 0.95 (0.74,1.22) | | | 0.96 (0.74,1.24) | | | p=0.7 | |
|  | **model 3** | **Cases/Participants** | | | 112 / 7566 | | | 167 / 9897 | | | 175 / 9967 | | |  | |
|  |  | **HR (95%CI)** | | | 1.00 | | | 0.95 (0.74,1.22) | | | 0.96 (0.74,1.24) | | | p=0.7 | |
|  | **model 4** | **Cases/Participants** | | | 109 / 7382 | | | 165 / 10128 | | | 179 / 9919 | | |  | |
|  |  | **HR (95%CI)** | | | 1.00 | | | 0.86 (0.67,1.10) | | | 0.96 (0.74,1.25) | | | p=0.95 | |
| **Sodium bicarbonate E500** | **model 1** | **Cases/Participants** | | | 151 / 9994 | | | 180 / 9777 | | | 123 / 7659 | | |  | |
|  |  | **HR (95%CI)** | | | 1.00 | | | 1.03 (0.82,1.29) | | | 0.89 (0.69,1.15) | | | p=0.5 | |
|  | **model 2** | **Cases/Participants** | | | 151 / 9994 | | | 180 / 9777 | | | 123 / 7659 | | |  | |
|  |  | **HR (95%CI)** | | | 1.00 | | | 1.03 (0.82,1.30) | | | 0.90 (0.70,1.16) | | | p=0.5 | |
|  | **model 3** | **Cases/Participants** | | | 151 / 9994 | | | 180 / 9777 | | | 123 / 7659 | | |  | |
|  |  | **HR (95%CI)** | | | 1.00 | | | 1.03 (0.82,1.30) | | | 0.90 (0.70,1.16) | | | p=0.5 | |
|  | **model 4** | **Cases/Participants** | | | 141 / 9532 | | | 199 / 10689 | | | 113 / 7208 | | |  | |
|  |  | **HR (95%CI)** | | | 1.00 | | | 0.94 (0.75,1.18) | | | 0.86 (0.66,1.12) | | | p=0.3 | |
| **Beeswax E901** | **model 1** | **Cases/Participants** | | | 426 / 26090 | | | 22 / 805 | | | 6 / 535 | | |  | |
|  |  | **HR (95%CI)** | | | 1.00 | | | 1.42 (0.92,2.20) | | | 0.71 (0.31,1.59) | | | p=0.8 | |
|  | **model 2** | **Cases/Participants** | | | 426 / 26090 | | | 22 / 805 | | | 6 / 535 | | |  | |
|  |  | **HR (95%CI)** | | | 1.00 | | | 1.42 (0.92,2.20) | | | 0.71 (0.32,1.59) | | | p=0.8 | |
|  | **model 3** | **Cases/Participants** | | | 426 / 26090 | | | 22 / 805 | | | 6 / 535 | | |  | |
|  |  | **HR (95%CI)** | | | 1.00 | | | 1.42 (0.92,2.20) | | | 0.71 (0.31,1.59) | | | p=0.8 | |
|  | **model 4** | **Cases/Participants** | | | 413 / 25123 | | | 31 / 1510 | | | 9 /795 | | |  | |
|  |  | **HR (95%CI)** | | | 1.00 | | | 0.96 (0.66,1.39) | | | 0.73 (0.38,1.42) | | | p=0.4 | |
| **Prostate cancer** | | |  |  | | |  | | |  | | |  | |  |
| **Total carrageenans** | **model 1** | **Cases/Participants** | | | 88 / 6577 | | | 144 / 6576 | | | 90 / 6577 | | |  | |
|  |  | **HR (95%CI)** | | | 1.00 | | | 1.33 (1.01, 1.75) | | | 1.22 (0.89, 1.67) | | | p=0.3 | |
|  | **model 2** | **Cases/Participants** | | | 88 / 6577 | | | 144 / 6576 | | | 90 / 6577 | | |  | |
|  |  | **HR (95%CI)** | | | 1.00 | | | 1.32 (1.01,1.74) | | | 1.20 (0.87,1.64) | | | p=0.3 | |
|  | **model 3** | **Cases/Participants** | | | 88 / 6577 | | | 144 / 6576 | | | 90 / 6577 | | |  | |
|  |  | **HR (95%CI)** | | | 1.00 | | | 1.32 (1.01,1.74) | | | 1.20 (0.88,1.65) | | | p=0.3 | |
|  | **model 4** | **Cases/Participants** | | | 86 / 6576 | | | 151 / 6577 | | | 85 / 6577 | | |  | |
|  |  | **HR (95%CI)** | | | 1.00 | | | 1.33 (1.02,1.75) | | | 1.29 (0.94,1.77) | | | p=0.1 | |
| **Carrageenan E407** | **model 1** | **Cases/Participants** | | | 88 / 6577 | | | 148 / 6576 | | | 86 / 6577 | | |  | |
|  |  | **HR (95%CI)** | | | 1.00 | | | 1.35 (1.03, 1.78) | | | 1.15 (0.84, 1.58) | | | p=0.5 | |
|  | **model 2** | **Cases/Participants** | | | 88 / 6577 | | | 148 / 6576 | | | 86 / 6577 | | |  | |
|  |  | **HR (95%CI)** | | | 1.00 | | | 1.34 (1.02,1.76) | | | 1.13 (0.82,1.56) | | | p=0.6 | |
|  | **model 3** | **Cases/Participants** | | | 88 / 6577 | | | 148 / 6576 | | | 86 / 6577 | | |  | |
|  |  | **HR (95%CI)** | | | 1.00 | | | 1.34 (1.02,1.76) | | | 1.14 (0.83,1.56) | | | p=0.6 | |
|  | **model 4** | **Cases/Participants** | | | 87 / 6576 | | | 149 / 6577 | | | 86 / 6577 | | |  | |
|  |  | **HR (95%CI)** | | | 1.00 | | | 1.28 (0.98,1.69) | | | 1.25 (0.91,1.72) | | | p=0.2 | |
| **Tripotassium phosphate E340** | **model 1** | **Cases/Participants** | | | 308 / 18889 | | | 9 / 421 | | | 5 / 420 | | |  | |
|  |  | **HR (95%CI)** | | | 1.00 | | | 1.19 (0.61, 2.32) | | | 0.76 (0.31, 1.85) | | | p=0.8 | |
|  | **model 2** | **Cases/Participants** | | | 308 / 18889 | | | 9 / 421 | | | 5 / 420 | | |  | |
|  |  | **HR (95%CI)** | | | 1.00 | | | 1.19 (0.61,2.31) | | | 0.75 (0.31,1.83) | | | p=0.8 | |
|  | **model 3** | **Cases/Participants** | | | 308 / 18889 | | | 9 / 421 | | | 5 / 420 | | |  | |
|  |  | **HR (95%CI)** | | | 1.00 | | | 1.19 (0.61,2.32) | | | 0.76 (0.31,1.84) | | | p=0.8 | |
|  | **model 4** | **Cases/Participants** | | | 290 / 18090 | | | 21 / 820 | | | 11 / 820 | | |  | |
|  |  | **HR (95%CI)** | | | 1.00 | | | 0.87 (0.56,1.36) | | | 0.66 (0.36,1.20) | | | p=0.1 | |
| **Diphosphates E450** | **model 1** | **Cases/Participants** | | | 94 / 6582 | | | 149 / 6574 | | | 79 / 6574 | | |  | |
|  |  | **HR (95%CI)** | | | 1.00 | | | 1.21 (0.92, 1.59) | | | 1.08 (0.75, 1.55) | | | p=0.5 | |
|  | **model 2** | **Cases/Participants** | | | 94 / 6582 | | | 149 / 6574 | | | 79 / 6574 | | |  | |
|  |  | **HR (95%CI)** | | | 1.00 | | | 1.18 (0.90,1.55) | | | 1.00 (0.73,1.38) | | | p=0.9 | |
|  | **model 3** | **Cases/Participants** | | | 94 / 6582 | | | 149 / 6574 | | | 79 / 6574 | | |  | |
|  |  | **HR (95%CI)** | | | 1.00 | | | 1.19 (0.91,1.55) | | | 1.00 (0.73,1.38) | | | p=0.8 | |
|  | **model 4** | **Cases/Participants** | | | 95 / 6576 | | | 148 / 6578 | | | 79 / 6576 | | |  | |
|  |  | **HR (95%CI)** | | | 1.00 | | | 1.02 (0.78,1.33) | | | 1.05 (0.76,1.44) | | | p=0.8 | |
| **Mono- and diglycerides of FAs E471** | **model 1** | **Cases/Participants** | | | 86 / 6577 | | | 133 / 6576 | | | 103 / 6577 | | |  | |
|  |  | **HR (95%CI)** | | | 1.00 | | | 1.33 (1.01, 1.76) | | | 1.49 (1.10, 2.00) | | | p=0.01 | |
|  | **model 2** | **Cases/Participants** | | | 86 / 6577 | | | 133 / 6576 | | | 103 / 6577 | | |  | |
|  |  | **HR (95%CI)** | | | 1.00 | | | 1.32 (1.00,1.73) | | | 1.46 (1.08,1.97) | | | p=0.02 | |
|  | **model 3** | **Cases/Participants** | | | 86 / 6577 | | | 133 / 6576 | | | 103 / 6577 | | |  | |
|  |  | **HR (95%CI)** | | | 1.00 | | | 1.32 (1.00,1.74) | | | 1.46 (1.09,1.97) | | | p=0.02 | |
|  | **model 4** | **Cases/Participants** | | | 98 / 6576 | | | 116 / 6577 | | | 108 / 6577 | | |  | |
|  |  | **HR (95%CI)** | | | 1.00 | | | 0.93 (0.71,1.23) | | | 1.35 (1.02,1.80) | | | p=0.04 | |
| **Polyglycerol esters of FAs E475** | **model 1** | **Cases/Participants** | | | 304 / 18539 | | | 12 / 611 | | | 6 / 580 | | |  | |
|  |  | **HR (95%CI)** | | | 1.00 | | | 1.14 (0.64, 2.05) | | | 1.17 (0.52, 2.65) | | | p=0.6 | |
|  | **model 2** | **Cases/Participants** | | | 304 / 18539 | | | 12 / 611 | | | 6 / 580 | | |  | |
|  |  | **HR (95%CI)** | | | 1.00 | | | 1.13 (0.63,2.03) | | | 1.14 (0.51,2.57) | | | p=0.6 | |
|  | **model 3** | **Cases/Participants** | | | 304 / 18539 | | | 12 / 611 | | | 6 / 580 | | |  | |
|  |  | **HR (95%CI)** | | | 1.00 | | | 1.14 (0.64,2.03) | | | 1.14 (0.51,2.56) | | | p=0.6 | |
|  | **model 4** | **Cases/Participants** | | | 286 / 18087 | | | 23 / 821 | | | 13 / 822 | | |  | |
|  |  | **HR (95%CI)** | | | 1.00 | | | 0.93 (0.61,1.43) | | | 1.28 (0.73,2.24) | | | p=0.6 | |
| **Carob bean gum E410** | **model 1** | **Cases/Participants** | | | 165 / 11070 | | | 88 / 4330 | | | 69 / 4330 | | |  | |
|  |  | **HR (95%CI)** | | | 1.00 | | | 0.94 (0.72, 1.23) | | | 1.12 (0.84, 1.50) | | | p=0.6 | |
|  | **model 2** | **Cases/Participants** | | | 165 / 11070 | | | 88 / 4330 | | | 69 / 4330 | | |  | |
|  |  | **HR (95%CI)** | | | 1.00 | | | 0.93 (0.71,1.22) | | | 1.12 (0.84,1.49) | | | p=0.6 | |
|  | **model 3** | **Cases/Participants** | | | 165 / 11070 | | | 88 / 4330 | | | 69 / 4330 | | |  | |
|  |  | **HR (95%CI)** | | | 1.00 | | | 0.94 (0.72,1.22) | | | 1.12 (0.84,1.49) | | | p=0.6 | |
|  | **model 4** | **Cases/Participants** | | | 99 / 8145 | | | 134 / 5793 | | | 89 / 5793 | | |  | |
|  |  | **HR (95%CI)** | | | 1.00 | | | 0.73 (0.55,0.96) | | | 0.81 (0.60,1.10) | | | p=0.2 | |
| **Guar gum E412** | **model 1** | **Cases/Participants** | | | 81 / 6577 | | | 127 / 6576 | | | 114 / 6577 | | |  | |
|  |  | **HR (95%CI)** | | | 1.00 | | | 1.19 (0.90,1.59) | | | 1.43 (1.07,1.93) | | | p=0.02 | |
|  | **model 2** | **Cases/Participants** | | | 81 / 6577 | | | 127 / 6576 | | | 114 / 6577 | | |  | |
|  |  | **HR (95%CI)** | | | 1.00 | | | 1.18 (0.89,1.58) | | | 1.39 (1.04,1.87) | | | p=0.03 | |
|  | **model 3** | **Cases/Participants** | | | 81 / 6577 | | | 127 / 6576 | | | 114 / 6577 | | |  | |
|  |  | **HR (95%CI)** | | | 1.00 | | | 1.18 (0.89,1.58) | | | 1.40 (1.04,1.87) | | | p=0.02 | |
|  | **model 4** | **Cases/Participants** | | | 74 / 6576 | | | 149 / 6577 | | | 99 / 6577 | | |  | |
|  |  | **HR (95%CI)** | | | 1.00 | | | 0.99 (0.83,1.18) | | | 0.70 (0.44,1.12) | | | p=0.6 | |
| **Gum arabic E414** | **model 1** | **Cases/Participants** | | | 296 / 18209 | | | 9 / 761 | | | 17 / 760 | | |  | |
|  |  | **HR (95%CI)** | | | 1.00 | | | 0.71 (0.36, 1.38) | | | 2.56 (1.55, 4.20) | | | p=0.009 | |
|  | **model 2** | **Cases/Participants** | | | 296 / 18209 | | | 9 / 761 | | | 17 / 760 | | |  | |
|  |  | **HR (95%CI)** | | | 1.00 | | | 0.71 (0.36,1.38) | | | 2.52 (1.53,4.15) | | | p=0.01 | |
|  | **model 3** | **Cases/Participants** | | | 296 / 18209 | | | 9 / 761 | | | 17 / 760 | | |  | |
|  |  | **HR (95%CI)** | | | 1.00 | | | 0.71 (0.36,1.38) | | | 2.53 (1.54,4.15) | | | p=0.009 | |
|  | **model 4** | **Cases/Participants** | | | 268 / 16437 | | | 25 / 1647 | | | 29 / 1646 | | |  | |
|  |  | **HR (95%CI)** | | | 1.00 | | | 0.48 (0.31,0.72) | | | 1.26 (0.85,1.86) | | | p=0.4 | |
| **Gum Xanthan Gum E415** | **model 1** | **Cases/Participants** | | | 83 / 6577 | | | 126 / 6576 | | | 113 / 6577 | | |  | |
|  |  | **HR (95%CI)** | | | 1.00 | | | 1.39 (1.05, 1.85) | | | 1.33 (0.99, 1.79) | | | p=0.1 | |
|  | **model 2** | **Cases/Participants** | | | 83 / 6577 | | | 126 / 6576 | | | 113 / 6577 | | |  | |
|  |  | **HR (95%CI)** | | | 1.00 | | | 1.36 (1.03,1.81) | | | 1.29 (0.96,1.73) | | | p=0.1 | |
|  | **model 3** | **Cases/Participants** | | | 83 / 6577 | | | 126 / 6576 | | | 113 / 6577 | | |  | |
|  |  | **HR (95%CI)** | | | 1.00 | | | 1.37 (1.03,1.81) | | | 1.30 (0.97,1.73) | | | p=0.1 | |
|  | **model 4** | **Cases/Participants** | | | 79 / 6576 | | | 134 / 6577 | | | 109 / 6577 | | |  | |
|  |  | **HR (95%CI)** | | | 1.00 | | | 0.94 (0.71,1.26) | | | 1.02 (0.76,1.38) | | | p=0.8 | |
| **Pectins E440** | **model 1** | **Cases/Participants** | | | 89 / 6581 | | | 129 / 6574 | | | 104 / 6575 | | |  | |
|  |  | **HR (95%CI)** | | | 1.00 | | | 0.94 (0.71, 1.24) | | | 0.79 (0.57, 1.08) | | | p=0.1 | |
|  | **model 2** | **Cases/Participants** | | | 89 / 6581 | | | 129 / 6574 | | | 104 / 6575 | | |  | |
|  |  | **HR (95%CI)** | | | 1.00 | | | 0.94 (0.71,1.24) | | | 0.78 (0.57,1.06) | | | p=0.1 | |
|  | **model 3** | **Cases/Participants** | | | 89 / 6581 | | | 129 / 6574 | | | 104 / 6575 | | |  | |
|  |  | **HR (95%CI)** | | | 1.00 | | | 0.94 (0.71,1.24) | | | 0.78 (0.57,1.07) | | | p=0.1 | |
|  | **model 4** | **Cases/Participants** | | | 86 / 6576 | | | 137 / 6577 | | | 99 / 6577 | | |  | |
|  |  | **HR (95%CI)** | | | 1.00 | | | 0.99 (0.75,1.31) | | | 0.78 (0.57,1.08) | | | p=0.1 | |
| **Sodium bicarbonate E500** | **model 1** | **Cases/Participants** | | | 91 / 6587 | | | 148 / 6567 | | | 83 / 6576 | | |  | |
|  |  | **HR (95%CI)** | | | 1.00 | | | 1.33 (1.01,1.74) | | | 1.04 (0.75,1.42) | | | p=0.8 | |
|  | **model 2** | **Cases/Participants** | | | 91 / 6587 | | | 148 / 6567 | | | 83 / 6576 | | |  | |
|  |  | **HR (95%CI)** | | | 1.00 | | | 1.33 (1.01,1.74) | | | 1.04 (0.76,1.42) | | | p=0.8 | |
|  | **model 3** | **Cases/Participants** | | | 91 / 6587 | | | 148 / 6567 | | | 83 / 6576 | | |  | |
|  |  | **HR (95%CI)** | | | 1.00 | | | 1.33 (1.01,1.74) | | | 1.04 (0.76,1.42) | | | p=0.8 | |
|  | **model 4** | **Cases/Participants** | | | 82 / 6576 | | | 164 / 6577 | | | 75 / 6577 | | |  | |
|  |  | **HR (95%CI)** | | | 1.00 | | | 1.18 (0.90,1.56) | | | 0.96 (0.70,1.34) | | | p=0.8 | |
| **Beeswax E901** | **model 1** | **Cases/Participants** | | | 307 / 18906 | | | 8 / 419 | | | 7 / 405 | | |  | |
|  |  | **HR (95%CI)** | | | 1.00 | | | 1.35 (0.67, 2.75) | | | 2.43 (1.14, 5.18) | | | p=0.03 | |
|  | **model 2** | **Cases/Participants** | | | 307 / 18906 | | | 8 / 419 | | | 7 / 405 | | |  | |
|  |  | **HR (95%CI)** | | | 1.00 | | | 1.36 (0.67,2.75) | | | 2.43 (1.14,5.18) | | | p=0.03 | |
|  | **model 3** | **Cases/Participants** | | | 307 / 18906 | | | 8 / 419 | | | 7 / 405 | | |  | |
|  |  | **HR (95%CI)** | | | 1.00 | | | 1.36 (0.67,2.76) | | | 2.42 (1.14,5.16) | | | p=0.03 | |
|  | **model 4** | **Cases/Participants** | | | 304 / 18426 | | | 10 / 652 | | | 8 / 652 | | |  | |
|  |  | **HR (95%CI)** | | | 1.00 | | | 0.70 (0.37,1.31) | | | 1.33 (0.65,2.70) | | | p=0.98 | |

^a^ Multivariable Cox proportional hazard models were adjusted for age (time-scale), sex, BMI (continuous, kg/m²), height (continuous, cm), physical activity (categorical IPAQ variable: high, moderate, low), smoking status (never smoked, former smoker, current smokers), number of smoked cigarettes in pack-years (continuous), educational level (less than high school degree, <2 y after high school degree, ≥2 y after high school degree), number of dietary records (continuous), family history of cancer (yes/no), energy intake without alcohol (continuous, kcal/d), daily intakes of alcohol (continuous, g/d), total lipid intake (continuous, g/d), dietary sodium (continuous, mg/d), total fiber (continuous, g/d), total sugars (continuous, g/d), fruits and vegetables (g/d), total dairy foods (continuous, g/d) and red and processed meats (continuous, g/d). Finally, breast cancer models were additionally adjusted for oral contraception (yes/no, in total and premenopausal models only), age at menarche (never, <12 y, ≥12 y), number of biological children (continuous), age at first biological child (no child, <30 y, ≥30 y), menopausal status at baseline (premenopausal, postmenopausal, in total models only), hormonal treatment for menopause (yes/no, in total and postmenopausal models only). **Model 1:** Main model + adjustment for the intakes of other emulsifiers than the one studied in the model (continuous, mg/d). **Model 2:** Main model + adjustment for proportion (%weight) of UPF (continuous, %). **Model 3**: Main model + adjustment for artificial sweeteners. **Model 4** was based on the main model, using the average of all available 24h dietary records throughout the follow-up of each participant (up to 62 records per participant) instead of averaged values on their first two years of follow-up.

^b^ Groups of emulsifiers were calculated as the sum of individual emulsifiers and defined as follows: total carrageenans (E407, E407a).

^c^ The following emulsifiers were coded as sex-specific tertiles: total carrageenans, E407, E412, E415, E440, E450, E471, and E500. Due to a higher proportion of non- consumers among the included participants, the following emulsifiers were coded as non-consumers (1^st^ category), low consumers (2^nd^ category), and high consumers (3^rd^ category), with low- and high-consumptions defined according to sex-specific median intakes among consumers: E340, E410, E414, E475 and E901. Cut-offs are presented in footnote to eTable D.

**eTable F. ‘Any’ versus ‘none’ models for the associations between emulsifier intakes and cancer risks among study participants from the NutriNet-Santé cohort, 2009-2021 (n=92,000)^a^.**

|  |  |  | **Categories of emulsifier intakes** |  |
| --- | --- | --- | --- | --- |
|  |  | **1 (non or low exposure)** | **2+3 (Exposure or higher exposure)** | **P-trend** |
| **Overall cancer** |  |  |  |  |
| **Total carrageenans**^b^ | **Cases/Participants** | 753 / 30684 | 1851 / 61316 |  |
|  | **HR (95%CI)** | 1.00 | 1.13 (1.03,1.24) | p=0.006 |
| **Carrageenan E407** | **Cases/Participants** | 755 / 30667 | 1849 / 61333 |  |
|  | **HR (95%CI)** | 1.00 | 1.12 (1.02,1.22) | p=0.02 |
| **Tripotassium phosphate E340** | **Cases/Participants** | 2445 / 86824 | 159 / 5176 |  |
|  | **HR (95%CI)** | 1.00 | 1.04 (0.89,1.23) | p=0.6 |
| **Diphosphates E450** | **Cases/Participants** | 838 / 30683 | 1766 / 61317 |  |
|  | **HR (95%CI)** | 1.00 | 1.01 (0.93,1.10) | p=0.8 |
| **Mono- and diglycerides of FAs E471** | **Cases/Participants** | 790 / 30667 | 1814 / 61333 |  |
|  | **HR (95%CI)** | 1.00 | 1.15 (1.06,1.26) | p=0.001 |
| **Polyglycerol esters of FAs E475** | **Cases/Participants** | 2431 / 85557 | 173 / 6443 |  |
|  | **HR (95%CI)** | 1.00 | 1.09 (0.93,1.28) | p=0.3 |
| **Carob bean gum E410** | **Cases/Participants** | 1277 / 49966 | 1327 / 42034 |  |
|  | **HR (95%CI)** | 1.00 | 1.05 (0.97,1.14) | p=0.2 |
| **Guar gum E412** | **Cases/Participants** | 733 / 30693 | 1871 / 61307 |  |
|  | **HR (95%CI)** | 1.00 | 1.10 (1.00,1.20) | p=0.04 |
| **Gum arabic E414** | **Cases/Participants** | 2342 / 82111 | 262 / 9889 |  |
|  | **HR (95%CI)** | 1.00 | 1.09 (0.95,1.24) | p=0.2 |
| **Gum Xanthan Gum E415** | **Cases/Participants** | 735 / 30670 | 1869 / 61330 |  |
|  | **HR (95%CI)** | 1.00 | 1.13 (1.03,1.23) | p=0.008 |
| **Pectins E440** | **Cases/Participants** | 657 / 30676 | 1947 / 61324 |  |
|  | **HR (95%CI)** | 1.00 | 1.08 (0.99,1.19) | p=0.1 |
| **Sodium bicarbonate E500** | **Cases/Participants** | 841 / 30705 | 1763 / 61295 |  |
|  | **HR (95%CI)** | 1.00 | 1.01 (0.93,1.11) | p=0.8 |
| **Beeswax E901** | **Cases/Participants** | 2465 / 86421 | 139 / 5579 |  |
|  | **HR (95%CI)** | 1.00 | 1.03 (0.87,1.22) | p=0.7 |
| **Overall breast cancer** |  |  |  |  |
| **Total carrageenans** | **Cases/Participants** | 192 / 24107 | 556 / 48163 |  |
|  | **HR (95%CI)** | 1.00 | 1.24 (1.04,1.47) | p=0.02 |
| **Carrageenan E407** | **Cases/Participants** | 194 / 24090 | 554 / 48180 |  |
|  | **HR (95%CI)** | 1.00 | 1.21 (1.02,1.43) | p=0.03 |
| **Tripotassium phosphate E340** | **Cases/Participants** | 700 / 67935 | 48 / 4335 |  |
|  | **HR (95%CI)** | 1.00 | 0.95 (0.71,1.28) | p=0.7 |
| **Diphosphates E450** | **Cases/Participants** | 207 / 24101 | 541 / 48169 |  |
|  | **HR (95%CI)** | 1.00 | 1.20 (1.01,1.42) | p=0.03 |
| **Mono- and diglycerides of FAs E471** | **Cases/Participants** | 211 / 24090 | 537 / 48180 |  |
|  | **HR (95%CI)** | 1.00 | 1.22 (1.03,1.44) | p=0.02 |
| **Polyglycerol esters of FAs E475** | **Cases/Participants** | 683 / 67018 | 65 / 5252 |  |
|  | **HR (95%CI)** | 1.00 | 1.27 (0.98,1.64) | p=0.1 |
| **Carob bean gum E410** | **Cases/Participants** | 330 / 38896 | 418 / 33374 |  |
|  | **HR (95%CI)** | 1.00 | 1.20 (1.03,1.39) | p=0.02 |
| **Guar gum E412** | **Cases/Participants** | 208 / 24116 | 540 / 48154 |  |
|  | **HR (95%CI)** | 1.00 | 1.06 (0.90,1.25) | p=0.5 |
| **Gum arabic E414** | **Cases/Participants** | 653 / 63902 | 95 / 8368 |  |
|  | **HR (95%CI)** | 1.00 | 1.13 (0.91,1.41) | p=0.3 |
| **Gum Xanthan Gum E415** | **Cases/Participants** | 196 / 24093 | 552 / 48177 |  |
|  | **HR (95%CI)** | 1.00 | 1.18 (1.00,1.40) | p=0.05 |
| **Pectins E440** | **Cases/Participants** | 172 / 24095 | 576 / 48175 |  |
|  | **HR (95%CI)** | 1.00 | 1.14 (0.95,1.36) | p=0.2 |
| **Sodium bicarbonate E500** | **Cases/Participants** | 214 / 24118 | 534 / 48152 |  |
|  | **HR (95%CI)** | 1.00 | 1.13 (0.95,1.34) | p=0.2 |
| **Beeswax E901** | **Cases/Participants** | 692 / 67515 | 56 / 4755 |  |
|  | **HR (95%CI)** | 1.00 | 1.15 (0.87,1.52) | p=0.3 |
| **Premenopausal breast cancer** |  |  |  |  |
| **Total carrageenans** | **Cases/Participants** | 64 / 17077 | 230 / 35886 |  |
|  | **HR (95%CI)** | 1.00 | 1.40 (1.05,1.87) | p=0.02 |
| **Carrageenan E407** | **Cases/Participants** | 65 / 17093 | 229 / 35870 |  |
|  | **HR (95%CI)** | 1.00 | 1.37 (1.03,1.83) | p=0.03 |
| **Tripotassium phosphate E340** | **Cases/Participants** | 267 / 49714 | 27 / 3249 |  |
|  | **HR (95%CI)** | 1.00 | 1.37 (0.91,2.04) | p=0.1 |
| **Diphosphates E450** | **Cases/Participants** | 61 / 16560 | 233 / 36403 |  |
|  | **HR (95%CI)** | 1.00 | 1.51 (1.12,2.03) | P=0.006 |
| **Mono- and diglycerides of FAs E471** | **Cases/Participants** | 73 / 16654 | 221 / 36309 |  |
|  | **HR (95%CI)** | 1.00 | 1.26 (0.96,1.66) | p=0.1 |
| **Polyglycerol esters of FAs E475** | **Cases/Participants** | 262 / 48656 | 32 / 4307 |  |
|  | **HR (95%CI)** | 1.00 | 1.29 (0.89,1.87) | p=0.2 |
| **Carob bean gum E410** | **Cases/Participants** | 125 / 28265 | 169 / 24698 |  |
|  | **HR (95%CI)** | 1.00 | 1.24 (0.97,1.58) | p=0.1 |
| **Guar gum E412** | **Cases/Participants** | 79 / 17423 | 215 / 35540 |  |
|  | **HR (95%CI)** | 1.00 | 1.07 (0.82,1.40) | p=0.6 |
| **Gum arabic E414** | **Cases/Participants** | 250 / 46093 | 44 / 6870 |  |
|  | **HR (95%CI)** | 1.00 | 1.05 (0.75,1.46) | p=0.8 |
| **Gum Xanthan Gum E415** | **Cases/Participants** | 71 / 17423 | 223 / 35540 |  |
|  | **HR (95%CI)** | 1.00 | 1.30 (0.99,1.72) | p=0.06 |
| **Pectins E440** | **Cases/Participants** | 60 / 18635 | 234 / 34328 |  |
|  | **HR (95%CI)** | 1.00 | 1.49 (1.11,2.01) | p=0.008 |
| **Sodium bicarbonate E500** | **Cases/Participants** | 63 / 16630 | 231 / 36333 |  |
|  | **HR (95%CI)** | 1.00 | 1.47 (1.09,1.98) | p=0.01 |
| **Beeswax E901** | **Cases/Participants** | 266 / 49020 | 28 / 3943 |  |
|  | **HR (95%CI)** | 1.00 | 1.12 (0.75,1.67) | p=0.6 |
| **Postmenopausal breast cancer** |  |  |  |  |
| **Total carrageenans** | **Cases/Participants** | 128 / 9458 | 326 / 17972 |  |
|  | **HR (95%CI)** | 1.00 | 1.14 (0.92,1.41) | p=0.2 |
| **Carrageenan E407** | **Cases/Participants** | 129 / 9405 | 325 / 18025 |  |
|  | **HR (95%CI)** | 1.00 | 1.11 (0.90,1.38) | p=0.3 |
| **Tripotassium phosphate E340** | **Cases/Participants** | 433 / 25795 | 21 / 1635 |  |
|  | **HR (95%CI)** | 1.00 | 0.68 (0.44,1.06) | p=0.08 |
| **Diphosphates E450** | **Cases/Participants** | 146 / 10072 | 308 / 17358 |  |
|  | **HR (95%CI)** | 1.00 | 1.06 (0.86,1.31) | p=0.6 |
| **Mono- and diglycerides of FAs E471** | **Cases/Participants** | 138 / 10053 | 316 / 17377 |  |
|  | **HR (95%CI)** | 1.00 | 1.18 (0.96,1.45) | p=0.1 |
| **Polyglycerol esters of FAs E475** | **Cases/Participants** | 421 / 25917 | 33 / 1513 |  |
|  | **HR (95%CI)** | 1.00 | 1.23 (0.86,1.76) | p=0.3 |
| **Carob bean gum E410** | **Cases/Participants** | 205 / 14641 | 249 / 12789 |  |
|  | **HR (95%CI)** | 1.00 | 1.17 (0.96,1.42) | p=0.1 |
| **Guar gum E412** | **Cases/Participants** | 129 / 9105 | 325 / 18325 |  |
|  | **HR (95%CI)** | 1.00 | 1.05 (0.85,1.29) | p=0.7 |
| **Gum arabic E414** | **Cases/Participants** | 403 / 24979 | 51 / 2451 |  |
|  | **HR (95%CI)** | 1.00 | 1.20 (0.89,1.61) | p=0.2 |
| **Gum Xanthan Gum E415** | **Cases/Participants** | 125 / 9111 | 329 / 18319 |  |
|  | **HR (95%CI)** | 1.00 | 1.10 (0.89,1.37) | p=0.4 |
| **Pectins E440** | **Cases/Participants** | 112 / 7566 | 342 / 19864 |  |
|  | **HR (95%CI)** | 1.00 | 0.96 (0.76,1.20) | p=0.7 |
| **Sodium bicarbonate E500** | **Cases/Participants** | 151 / 9994 | 303 / 17436 |  |
|  | **HR (95%CI)** | 1.00 | 0.98 (0.80,1.21) | p=0.8 |
| **Beeswax E901** | **Cases/Participants** | 426 / 26090 | 28 / 1340 |  |
|  | **HR (95%CI)** | 1.00 | 1.17 (0.79,1.73) | p=0.4 |
| **Prostate cancer** |  |  |  |  |
| **Total carrageenans** | **Cases/Participants** | 88 / 6577 | 234 / 13153 |  |
|  | **HR (95%CI)** | 1.00 | 1.28 (0.99,1.65) | p=0.06 |
| **Carrageenan E407** | **Cases/Participants** | 88 / 6577 | 234 / 13153 |  |
|  | **HR (95%CI)** | 1.00 | 1.27 (0.98,1.64) | p=0.07 |
| **Tripotassium phosphate E340** | **Cases/Participants** | 308 / 18889 | 14 / 841 |  |
|  | **HR (95%CI)** | 1.00 | 0.99 (0.58,1.69) | p=0.97 |
| **Diphosphates E450** | **Cases/Participants** | 94 / 6582 | 228 / 13148 |  |
|  | **HR (95%CI)** | 1.00 | 1.12 (0.87,1.44) | p=0.4 |
| **Mono- and diglycerides of FAs E471** | **Cases/Participants** | 86 / 6577 | 236 / 13153 |  |
|  | **HR (95%CI)** | 1.00 | 1.37 (1.07,1.77) | p=0.01 |
| **Polyglycerol esters of FAs E475** | **Cases/Participants** | 304 / 18539 | 18 / 1191 |  |
|  | **HR (95%CI)** | 1.00 | 1.14 (0.71,1.84) | p=0.6 |
| **Carob bean gum E410** | **Cases/Participants** | 165 / 11070 | 157 / 8660 |  |
|  | **HR (95%CI)** | 1.00 | 1.01 (0.80,1.27) | p=0.9 |
| **Guar gum E412** | **Cases/Participants** | 81 / 6577 | 241 / 13153 |  |
|  | **HR (95%CI)** | 1.00 | 1.28 (0.99,1.65) | p=0.06 |
| **Gum arabic E414** | **Cases/Participants** | 296 / 18209 | 26 / 1521 |  |
|  | **HR (95%CI)** | 1.00 | 1.34 (0.89,2.01) | p=0.2 |
| **Gum Xanthan Gum E415** | **Cases/Participants** | 83 / 6577 | 239 / 13153 |  |
|  | **HR (95%CI)** | 1.00 | 1.33 (1.03,1.72) | p=0.03 |
| **Pectins E440** | **Cases/Participants** | 89 / 6581 | 233 / 13149 |  |
|  | **HR (95%CI)** | 1.00 | 0.87 (0.67,1.13) | p=0.3 |
| **Sodium bicarbonate E500** | **Cases/Participants** | 91 / 6587 | 231 / 13143 |  |
|  | **HR (95%CI)** | 1.00 | 1.21 (0.94,1.57) | p=0.1 |
| **Beeswax E901** | **Cases/Participants** | 307 / 18906 | 15 / 824 |  |
|  | **HR (95%CI)** | 1.00 | 1.71 (1.01,2.89) | p=0.046 |

^a^ Multivariable Cox proportional hazard models were adjusted for age (time-scale), sex, BMI (continuous, kg/m²), height (continuous, cm), physical activity (categorical IPAQ variable: high, moderate, low), smoking status (never smoked, former smoker, current smokers), number of smoked cigarettes in pack-years (continuous), educational level (less than high school degree, <2 y after high school degree, ≥2 y after high school degree), number of dietary records (continuous), family history of cancer (yes/no), energy intake without alcohol (continuous, kcal/d), daily intakes of alcohol (continuous, g/d), total lipid intake (continuous, g/d), dietary sodium (continuous, mg/d), total fiber (continuous, g/d), total sugars (continuous, g/d), fruits and vegetables (g/d), total dairy foods (continuous, g/d) and red and processed meats (continuous, g/d). Finally, breast cancer models were additionally adjusted for oral contraception (yes/no, in total and premenopausal models only), age at menarche (never, <12 y, ≥12 y), number of biological children (continuous), age at first biological child (no child, <30 y, ≥30 y), menopausal status at baseline (premenopausal, postmenopausal, in total models only), hormonal treatment for menopause (yes/no, in total and postmenopausal models only).

^b^ Groups of emulsifiers were calculated as the sum of individual emulsifiers and defined as follows: total carrageenans (E407, E407a).

**eTable G. Associations between patterns of emulsifier intakes (create with principal component analysis) and cancer risks among study participants from the NutriNet-Santé cohort, 2009-2021 (n=92,000).^a^**

|  |  | **Categories of emulsifier patterns ^b^** | | |  |
| --- | --- | --- | --- | --- | --- |
|  |  | **1** | **2** | **3** | **P-trend** |
| **Overall cancer** |  |  |  |  |  |
| **Main component 1** | **Cases/Participants** | 905 / 30666 | 916 / 30667 | 783 / 30667 |  |
|  | **HR (95%CI)** | 1.00 | 1.05 (0.96,1.16) | 1.07 (0.96,1.19) | p=0.2 |
| **Main component 2** | **Cases/Participants** | 956 / 30666 | 899 / 30667 | 749 / 30667 |  |
|  | **HR (95%CI)** | 1.00 | 0.89 (0.81,0.99) | 0.93 (0.84,1.03) | p=0.1 |
| **Main component 3** | **Cases/Participants** | 778 / 30666 | 911 / 30667 | 915 / 30667 |  |
|  | **HR (95%CI)** | 1.00 | 0.98 (0.88,1.08) | 0.97 (0.88,1.07) | p=0.6 |
| **Overall breast cancer** |  |  |  |  |  |
| **Main component 1** | **Cases/Participants** | 221 / 24090 | 274 / 24090 | 253 / 24090 |  |
|  | **HR (95%CI)** | 1.00 | 1.21 (1.01,1.45) | 1.25 (1.02,1.52) | p=0.03 |
| **Main component 2** | **Cases/Participants** | 285 / 24090 | 235 / 24090 | 228 / 24090 |  |
|  | **HR (95%CI)** | 1.00 | 0.84 (0.70,1.01) | 0.94 (0.78,1.12) | p=0.4 |
| **Main component 3** | **Cases/Participants** | 245 / 24090 | 243 / 24090 | 260 / 24090 |  |
|  | **HR (95%CI)** | 1.00 | 0.90 (0.74,1.08) | 0.89 (0.74,1.06) | p=0.2 |
| **Premenopausal breast cancer** |  |  |  |  |  |
| **Main component 1** | **Cases/Participants** | 68 / 16103 | 111 / 17708 | 115 / 19152 |  |
|  | **HR (95%CI)** | 1.00 | 1.36 (1.00,1.86) | 1.35 (0.98,1.87) | p=0.09 |
| **Main component 2** | **Cases/Participants** | 93 / 17156 | 92 / 16652 | 109 / 19155 |  |
|  | **HR (95%CI)** | 1.00 | 1.07 (0.79,1.45) | 1.14 (0.86,1.51) | p=0.4 |
| **Main component 3** | **Cases/Participants** | 108 / 18712 | 97 / 16917 | 89 / 17334 |  |
|  | **HR (95%CI)** | 1.00 | 0.95 (0.71,1.27) | 0.78 (0.59,1.04) | p=0.09 |
| **Postmenopausal breast cancer** |  |  |  |  |  |
| **Main component 1** | **Cases/Participants** | 153 / 10640 | 163 / 9204 | 138 / 7586 |  |
|  | **HR (95%CI)** | 1.00 | 1.11 (0.88,1.40) | 1.17 (0.90,1.51) | p=0.2 |
| **Main component 2** | **Cases/Participants** | 192 / 9811 | 143 / 10220 | 119 / 7399 |  |
|  | **HR (95%CI)** | 1.00 | 0.75 (0.59,0.94) | 0.83 (0.66,1.05) | p=0.1 |
| **Main component 3** | **Cases/Participants** | 137 / 7782 | 146 / 9895 | 171 / 9753 |  |
|  | **HR (95%CI)** | 1.00 | 0.87 (0.68,1.11) | 0.96 (0.76,1.20) | p=0.8 |
| **Prostate cancer** |  |  |  |  |  |
| **Main component 1** | **Cases/Participants** | 124 / 6576 | 115 / 6577 | 83 / 6577 |  |
|  | **HR (95%CI)** | 1.00 | 1.04 (0.80,1.35) | 1.05 (0.77,1.43) | p=0.8 |
| **Main component 2** | **Cases/Participants** | 117 / 6576 | 113 / 6577 | 92 / 6577 |  |
|  | **HR (95%CI)** | 1.00 | 0.85 (0.64,1.12) | 0.94 (0.71,1.24) | p=0.6 |
| **Main component 3** | **Cases/Participants** | 95 / 6576 | 121 / 6577 | 106 / 6577 |  |
|  | **HR (95%CI)** | 1.00 | 1.02 (0.76,1.36) | 0.91 (0.69,1.21) | p=0.5 |

^a^ Multivariable Cox proportional hazard models were adjusted for age (time-scale), sex, BMI (continuous, kg/m²), height (continuous, cm), physical activity (categorical IPAQ variable: high, moderate, low), smoking status (never smoked, former smoker, current smokers), number of smoked cigarettes in pack-years (continuous), educational level (less than high school degree, <2 y after high school degree, ≥2 y after high school degree), number of dietary records (continuous), family history of cancer (yes/no), energy intake without alcohol (continuous, kcal/d), daily intakes of alcohol (continuous, g/d), total lipid intake (continuous, g/d), dietary sodium (continuous, mg/d), total fiber (continuous, g/d), total sugars (continuous, g/d), fruits and vegetables (g/d), total dairy foods (continuous, g/d) and red and processed meats (continuous, g/d). Finally, breast cancer models were additionally adjusted for oral contraception (yes/no, in total and premenopausal models only), age at menarche (never, <12 y, ≥12 y), number of biological children (continuous), age at first biological child (no child, <30 y, ≥30 y), menopausal status at baseline (premenopausal, postmenopausal, in total models only), hormonal treatment for menopause (yes/no, in total and postmenopausal models only).
^b^ Categories computed according to principal component analysis explained in eMethod4. Sex-specific tertiles for component 1 were -0.77 and 0.57 in men; and
-0.86 and 0.28 in women respectively; those of component 2 were -0.52 and 0.14 in men; and -0.44 and 0.19 in women respectively, and those of component 3 were -0.42 and 0.31 in men; and -0.45 and 0.14 in women respectively.

## **References**

1. Black AE. Critical evaluation of energy intake using the Goldberg cut-off for energy intake:basal metabolic rate. A practical guide to its calculation, use and limitations. *IntJObesRelat Metab Disord*. 2000;24(0307-0565 (Print)):1119-1130.

2. Black AE. The sensitivity and specificity of the Goldberg cut-off for EI:BMR for identifying diet reports of poor validity. *European Journal of Clinical Nutrition*. 2000;54(5):395-404. doi:10.1038/sj.ejcn.1600971

3. Goldberg GR, Black AE, Jebb SA, et al. Critical evaluation of energy intake data using fundamental principles of energy physiology: 1. Derivation of cut-off limits to identify under-recording. *Eur J Clin Nutr*. 1991;45(12):569-581.

4. Schofield WN. Predicting basal metabolic rate, new standards and review of previous work. *HumNutr Clin Nutr*. 1985;39 Suppl 1(0263-8290 (Print)):5-41.

5. Anses. *Etude Individuelle Nationale Des Consommations Alimentaires 3 (INCA 3)*.; 2017.

6. Harrell F, Dupont C. *Harrell Miscellaneous*. https://cran.r-project.org/web/packages/Hmisc/Hmisc.pdf

7. Lê S, Josse J, Husson F. {FactoMineR}: A Package for Multivariate Analysis. *Journal of Statistical Software*. 2008;25(1):1-18. doi:10.18637/jss.v025.i01

8. Benjamini Y, Hochberg Y. Controlling the False Discovery Rate: A Practical and Powerful Approach to Multiple Testing. *Journal of the Royal Statistical Society Series B (Methodological)*. 1995;57(1):289-300.

9. Therneau TM. *A Package for Survival Analysis in R*.; 2020. https://CRAN.R-project.org/package=survival

10. Schoenfeld D. Partial residuals for the proportional hazards regression model. *Biometrika*. 1982;69(1):239-241. doi:10.1093/biomet/69.1.239

11. Desquilbet L, Mariotti F. Dose-response analyses using restricted cubic spline functions in public health research. *Statistics in Medicine*. 2010;29(9):1037-1057. doi:10.1002/sim.3841
