# Supplementary material for: Food additive emulsifiers and cancer risk: Results from the French prospective NutriNet-Santé cohort
Source: PLoS Med. 2024 Feb 13;21(2):e1004338. doi: 10.1371/journal.pmed.1004338 (PMC10863884; doi:10.1371/journal.pmed.1004338)
Supplement: Attachment — Submitted filename: sellem.pdf [file pmed.1004338.s003.pdf]

This paper examines the association between consumptions of certain food additives and incident cancer in a French cohort.

I was asked for a statistical report and I interpret that to include all aspects of the design and conduct of the study.

Points of detail

Page 2 To make the abstract self–contained I would say what 'these associations' are.

Page 2 Later the authors use a more conventional notation for means and standard deviation and I suggest that would be better here too.

Page 4 Saying the first is a bit of a hostage to fortune. The more cautious tone of page 6 would be better.

Page 8 Providing some summary of the method for eliminating under–reporting would be beneficial or at least call out where it is expanded on in the supplement. Eliminating 16.5% of the cohort seems a rather large loss which needs more discussion especially if it is non–random. What covariates predict it? Have the authors considered multiple imputation for these mssing values?

Page 10 I know this is very pedantic but there are only two tertiles, what the authors have here is three tertile categories. Why not just call them low, medium and high in the same way as the other variables are split none, low and high?

Page 10 Good to see the package properly cited (and other packages in the supplementary material). What about the competing risk analysis package?

Page 12 Is the test for trend a test for linear trend? Or is this what is explained in eMethod6? An extra sentence here would help or in the methods section.

Page 23 Is the statement about participant and public involvement here based on the contribution of a formal advisory group? That would make it more convincing.

Figure 1 I think pie charts are deprecated these days. See the references in the English Wikipedia page `https://en.wikipedia.org/wiki/Pie_chart`

Figure 3 I am afraid this is unreadable, at least in the version I received.

References Perhaps give translations of the French titles so people know what they are missing?

eFigure1 I think this is too important to be put in the supplement.

eMethod5 With an N of 100000 almost any correlation is going to achieve a statistically significant level so I think it is the overall shape here which needs examining. I did not see anything very concerning in the plots.

eFigures The labels for the axes are unreadable in most of these, perhaps as a result of giving very detailed information in them.

Points of more substance

Mutual adjustment of additives

The authors have adjusted each additive for the effect of all the others (see page 10). This corresponds, as I see it, to the scientific question: What is the effect of this additive if all the others were held constant? The alternative, of course, is to present each additive unadjusted for the others. The choice here seems to depend on what the precise scientific question is.

- If most people only consume some of the additives then the evidence relevant to their choice would come from the analysis unadjusted. The adjusted analysis adjusts for things which are not relevant to them.
- If most people consume across the whole range of additives then the full model is most relevant to the choices they make.

The eFigure2 heatmap displays mostly weak relationships between additives suggesting that most people do not consume all or most of them (although there might be other factors at work here).

UPF

The authors use the concept of UPF several times. I am not an expert in nutritional epidemiology but my understanding is that at least one of the ways of measuring it, NOVA, has some problems (Braesco et al., 2022). I see that the authors' reference 61 used it.

Summary

Fully reported account of a major piece of work. Apart from various points of detail I have raised a concern about whether the analysis is optimal for the scientific or clinical question.

Michael Dewey

References

V Braesco, I Souchon, P Sauvant, T Haurogné, M Maillot, C Féart, and N Darmon. Ultra–processed foods: how functional is the NOVA system? *European Journal of Clinical Nutrition*, 76:1245–1253, 2022.
