# Supplementary material for: Food additive emulsifiers and cancer risk: Results from the French prospective NutriNet-Santé cohort
Source: PLoS Med. 2024 Feb 13;21(2):e1004338. doi: 10.1371/journal.pmed.1004338 (PMC10863884; doi:10.1371/journal.pmed.1004338)
Supplement: Attachment — Submitted filename: rebuttal_3108.docx [file pmed.1004338.s004.docx]

-----------------------------------------------------------

Requests from the editors:

Your manuscript has been assessed by three reviewers whose reports can be found below. As you will see from the comments, the reviewers have raised a number of concerns that need addressing. The academic editor also left comments. Please carefully revise the manuscript to address all comments raised.

+++ We thank the Editorial board and the Reviewers for their input and comments. We have addressed every comment they have raised in the rebuttal below.

We like to add for your consideration to examine ‘any’ vs ‘none’ comparisons, and provide cumulative counts and principal component analysis (dimension reduction).

+++ As requested, we have now performed “any” vs “none” comparisons by combining the second and the third categories of exposure, and assessing the hazard of cancer in this category in comparison to the one in none/low exposed participants. This has been added in the eMethods 5 and eTable 5.

Cumulative counts have been calculated with the Fine-Gray subdistribution model to account for competing risks, and plots are now presented in the eFigures 4.

To comply with this comment, we have now performed a principal component analysis that we presented in the eMethods 4 and eTable 6. The first component, characterized particularly by high intakes of carrageenans, E410 and E471, was positively associated with increased breast cancer risk, consistently with our main models when these emulsifiers were assessed individually. Components 2 and 3 were not associated with cancer risk, but it was expected since they were characterized by both high consumption of some emulsifiers and low consumption of some others, blurring the associations, compared to the approach where emulsifiers were investigated separately.

COHORT STUDIES:

Please ensure that the study is reported according to the STROBE guideline, and include the completed STROBE checklist as Supporting Information. Please add the following statement, or similar, to the Methods: ""This study is reported as per the Strengthening the Reporting of Observational Studies in Epidemiology (STROBE) guideline (S1 Checklist).

The STROBE guideline can be found here: <http://www.equator-network.org/reporting-guidelines/strobe/>

When completing the checklist, please use section and paragraph numbers, rather than page numbers.

+++ This study is reported as per the Strengthening the Reporting of Observational Studies in Epidemiology (STROBE) guideline (S1 Checklist). We have added the STROBE checklist to the supplementary material.

Did your study have a prospective protocol or analysis plan? Please state this (either way) early in the Methods section.

a) If an analysis plan (from your funding proposal, IRB or other ethics committee submission, study protocol, or other planning document written before analyzing the data) was used in designing the study, please include the relevant document with your revised manuscript as a Supporting Information file to be published alongside your study, and cite it in the Methods section. A legend for this file should be included at the end of your manuscript.

b) If no such document exists, please make sure that the Methods section transparently describes when analyses were planned, and when/why any data-driven changes to analyses took place.

c) In either case, changes in the analysis-- including those made in response to peer review comments-- should be identified as such in the Methods section of the paper, with rationale.

For all observational studies, in the manuscript text, please indicate: (1) the specific hypotheses you intended to test, (2) the analytical methods by which you planned to test them, (3) the analyses you actually performed, and (4) when reported analyses differ from those that were planned, transparent explanations for differences that affect the reliability of the study's results. If a reported analysis was performed based on an interesting but unanticipated pattern in the data, please be clear that the analysis was data-driven.

+++ These elements have been added to the Methods section as requested.

*“The NutriNet-Santé study is conducted according to the Declaration of Helsinki guidelines and was approved by the Institutional Review Board of the French Institute for Health and Medical Research (IRB Inserm n°0000388FWA00005831) and the “Commission Nationale de l’Informatique et des Libertés” (CNIL n°908450/n°909216). It is registered at clinicaltrials.gov as NCT03335644. Electronic informed consent is obtained from each participant. The NutriNet-Santé study was developed to investigate the relationships between multiple dietary exposures and the incidence of chronic diseases, such as cancer. The general protocol of the cohort, written in 2008 before the beginning of the study, is available online [36]. Regarding food additives specifically, the present work is part of a series of prespecified analyses that are included in a project funded by the European Research Council (*[*https://erc.europa.eu/news-events/magazine/erc-2019-consolidator-grants-examples#ADDITIVES)*](https://erc.europa.eu/news-events/magazine/erc-2019-consolidator-grants-examples#ADDITIVES))*.”*

And:

*“All methods have been described in line with the Strengthening the Reporting of Observational Studies in nutritional Epidemiology guidelines (S1 STROBE Checklist). Multi-adjusted Cox models for several confounders were prespecified. The analyses added following the peer-review process were as follows: restricting the sample to participants with at least 3 dietary records, starting follow-up 2 years after enrolment, further adjustments (for food groups and nutrients instead of dietary patterns, for artificial sweeteners in sensitivity analyses), analysis using all dietary records available during follow-up, any versus non comparisons, principal component analysis to compute emulsifier patterns, and cumulative incidence functions.”*

DATA AVAILABILITY STATEMENT:

PLOS Medicine requires that the de-identified data underlying the specific results in a published article be made available, without restrictions on access, in a public repository or as Supporting Information at the time of article publication, provided it is legal and ethical to do so. Please see the policy at <http://journals.plos.org/plosmedicine/s/data-availability> and FAQs at <http://journals.plos.org/plosmedicine/s/data-availability#loc-faqs-for-data-policy>

PLOS defines the “minimal data set” to consist of the data set used to reach the conclusions drawn in the manuscript with related metadata and methods, and any additional data required to replicate the reported study findings in their entirety.

+++ A data availability statement has been added, as follows:

*Data availability statement*

*Data described in the manuscript, code book, and analytic code will be made available upon request pending application and approval. Researchers from public institutions can submit a collaboration request including information on the institution and a brief description of the project to collaboration@etude-nutrinet-sante.fr. All requests will be reviewed by the steering committee of the NutriNet-Santé study. If the collaboration request is accepted, a data access agreement will be necessary and appropriate authorizations from the competent administrative authorities may be needed. In accordance with existing regulations, no personal identification data will be accessible.*

ABSTRACT:

Please structure your abstract using the PLOS Medicine headings: Background, Methods and Findings, Conclusions.

Please remove all other subheaders.

Abstract Background:

Provide the context of why the study is important. The final sentence should clearly state the study question.

Abstract Methods and Findings:

Please give further details of the studied cohort.

Please define all abbreviations for the reader at first use including those used for statistical reporting , HR, 95% CI, for example (lines 49-50).

Line 45 - “…cohort (42.1y [14.5], 78.8% female, 2009-2021)…” Please clearly define what the numerical values contained within square parentheses depict.

Suggest use of commas as opposed to hyphens (as these can be confused with negative values) to separate upper and lower bounds.

In the last sentence of the Abstract Methods and Findings section, please describe the main limitation(s) of the study's methodology.

+++ The abstract has been reformatted as requested.

Abstract Conclusions:

Please address the study implications without overreaching what can be concluded from the data.

Please interpret the study based on the results presented in the abstract, emphasizing what is new without overstating your conclusions.

Please avoid assertions of primacy ("These results provide the first epidemiological insights....")

+++ The abstract conclusions have been edited as requested.

AUTHOR SUMMARY:

Line 72 starting “Experimental…” suggest separate bullet point.

Line 86 - Assertions of primacy.

The E numbers are provided with a space between E and the number. The spaces should be removed throughout (e.g. E331 and not E 331).

+++ The author summary has been amended as requested.

INTRODUCTION:

Please address past research and explain the need for and potential importance of your study.

Please indicate whether your study is novel and how you determined that.

Please conclude the Introduction with a clear description of the study question or hypothesis.

+++ Some elements of the introduction have been restructured to emphasize the novelty of the present study. The introduction now ends with a clear description of the aims of this study.

*“However, the impact of food additive emulsifiers on cancer risk or carcinogenesis progression, is yet to be elucidated and current knowledge is based on scarce, contrasting evidence from experimental studies on animals [28–30]. To our knowledge, no epidemiological study has investigated the links between exposure to emulsifiers and cancer risk in humans.*

*In this context, there is a crucial need for large-scale epidemiological studies to understand the role played by food additive emulsifiers on human health, and particularly their potential long-term impact on non-communicable diseases such as cancers. In the prospective NutriNet-Santé cohort, which collected detailed information on specific commercial brands of industrial food consumed, we recently estimated the intakes of individual food additives (including emulsifiers), in more than 100,000 French adults [17]. Based on this previous work, the present study aims to assess the associations between exposure to food additive emulsifiers and cancer risk in the NutriNet-Santé prospective cohort.”*

For in-text reference callouts, citations placed within square parentheses should precede punctuation as follows, line 90 “…60% in the US and the UK [1], and about 30% in France [2] and throughout Europe [3].” Please check and amend throughout.

+++ These have been amended throughout the text.

Line 112: space missing after microbiota

+++ Done

METHODS and RESULTS:

Please add the following statement, or similar, to the Methods: "This study is reported as per the Strengthening the Reporting of Observational Studies in Epidemiology (STROBE) guideline (S1 Checklist).”

+++ Done

What is the meaning of the grey highlighted text? Please amend/clarify Line 155-156 “… and urinary and blood markers”. Please provide more detail.

+++ The grey highlighted text (previously used to refer to corresponding sections in the results or supplementary material) has been removed. The statement on markers has been clarified:

‘The web-based questionnaires used in the study (available here https://etude-nutrinet-sante.fr/build/qa/docs/guide.htm#environnement) have been tested and validated against both in-person interviews by trained dietitians [37], and urinary and blood markers [38,39] for the key food groups and nutrients (against plasma beta carotene, vitamin C, and n-3 polyunsaturated fatty acids and urinary protein, potassium and sodium), but not food additives.’

Line 171 – please define “OQALI and INRAE”

Line 173 - you may wish to include “... Agence nationale de sécurité sanitaire de l'alimentation, de l'environnement et du travail - ANSES)...”

+++ These have been defined.

Line 208 - “... ANOVA tests …” please define all abbreviations at first use Line 230 - “… consumed in g/d … ” please define all abbreviations at first use

Please define all abbreviations at first use, check and amend throughout.

+++ All abbreviations have been defined.

Equally, when reporting statistical information please define all abbreviations at first use – SD, HR, CI and so on - for the reader Suggest reporting statistical information as follows for clarity for the reader “…HR 1.11; 95%CI [1.02,1.21]; p trend=0.02…” (instead of HR=1.11, 95%CI=1.02-1.21, p-trend=0.02)

+++ Statistical reporting has been modified to match the suggested format.

We note that you have adjusted each additive for the effect of all the others. To help facilitate transparent data reporting, please also include unadjusted analyses for comparison. This also ties into a comment by reviewer 1, who notes that if most people only consume some of the additives, then the evidence relevant to their choice would come from the unadjusted analysis.

+++ The main model is now unadjusted for all other emulsifiers. The model with mutual adjustment has been moved to sensitivity analyses (model 1); the two approaches provided similar results.

DISCUSSION:

Please present and organize the Discussion as follows: a short, clear summary of the article's findings; what the study adds to existing research and where and why the results may differ from previous research; strengths and limitations of the study; implications and next steps for research, clinical practice, and/or public policy; one-paragraph conclusion.

Your discussion is largely structured in this way, we suggest you elaborate a bit more on possible next steps and/or public policy and remove assertions of primacy (e.g. see line 375).

+++ We have now removed all assertions of primacy from the discussion and elaborated more on next steps and policy changes, as follows:

‘These results provide novel epidemiological insights into the role of food additive emulsifiers on cancer risk. If confirmed by further epidemiological and experimental research, they could lead to a modification in the regulation of emulsifier use by the food industry, through food policies requiring a modification of the ADI of some emulsifiers, or even restricting the use of others, for better citizen protection. Given the recently established links between ultra-processed food, main source of food additive emulsifiers, and human health, the role of emulsifiers in the development of other long-term non-communicable diseases should also be explored, through epidemiological research, as well as experimental approaches on humans and animal models whenever feasible. In the meantime, several public health authorities recommend limiting the consumption of foods containing “cosmetic” additives, i.e. not essential for consumer safety [76,77].’

DISCLAIMER STATEMENTS:

Please remove all statements apart from acknowledgements, author contributions and abbreviations from the end of the main manuscript and include these only in the relevant parts of the manuscript submission form. They will be complied as metadata

+++ All unnecessary statements have been removed.

TABLES:

We note that the E numbers are provided with a space between E and the number e.g. E 339 instead of E339. The spaces should be removed throughout (applicable to all tables).

+++ Spaces have been removed throughout.

Please ensure that any and all abbreviations detailed in the tables are clearly defined in the caption/legend for the reader (e.g. see SD for table 1 and 2) Table 1 - Please report p values as p<0.001 and where higher as p=0.002, for example and not as <.001

+++ Done

FIGURES:

Figure titles and captions: We note that the E numbers are provided with a space between E and the number e.g. E 339 instead of E339. The spaces should be removed throughout.

+++ Done

Figure 1 - Please convert the pie chart in Figure 1 to a table, or another type of graph. Please consider avoiding the use of green and/or red to make your figures more accessible to those with colour blindness.

+++ A bar chart has now been used instead.

Figure 3: The text and numerical values are very small and rather inaccessible to the reader, please revise. Please clearly define the meaning of the dots and lines in the figure caption.

+++ Figure 3 has been revised accordingly.

SUPPORTING INFORMATION:

As above, please include the statistical analysis plan/study protocol and STROBE checklist

+++ The protocol of the cohort has been cited, and a STROBE checklist has been included.

Line 145 mentions a web based questionnaire. Please provide a copy of the questionnaire as an SI file.

+++ The questionnaire is available using this link <https://etude-nutrinet-sante.fr/build/qa/docs/guide.htm#environnement>. This has been added to the Methods section line 167.

We note that the clinical trial study was registered first on November 8, 2017, updated on July 26, 2021, and has a continuous enrollment. In accordance with ICMJE requirements, PLOS Medicine requires prospective, public registration of a data sharing plan (as part of clinical trials registration) for all trials that begin enrollment on or after January 1, 2019. Do you have a data sharing plan? If so, please add it as supporting information, if not please clearly state the reasons why not.

+++ Our study is a prospective cohort study and the enrolment started in 2009. No data sharing plan is available. As mentioned above, data described in the manuscript, code book, and analytic code will be made available upon request pending application and approval. Researchers from public institutions can submit a collaboration request including information on the institution and a brief description of the project to collaboration@etude-nutrinet-sante.fr. All requests will be reviewed by the steering committee of the NutriNet-Santé study. If the collaboration request is accepted, a data access agreement will be necessary and appropriate authorizations from the competent administrative authorities may be needed. In accordance with existing regulations, no personal identification data will be accessible.

eFigure 1:Thank you for including the participant flowchart

+++ We have moved it to the main text now.

eFigure 2: E numbers are provided with a space between E and the number. Please amend.

+++ This has been amended.

-----------------------------------------------------------

Comments from the AE:

Confounding matters, but the approach is not much justifiable.

1. Looking at the published paper on the associations of artificial sweeteners with cancer incidence in the same cohort at PLOS Medicine: <https://journals.plos.org/plosmedicine/article?id=10.1371/journal.pmed.1003950>.

This clearly indicates confounding by artificial sweeteners.

+++ We have now tested an adjustment for artificial sweeteners in sensitivity analyses, model 3, eTable4. Results remained similar. On a conceptual framework, there is no evidence to assume that high consumption of sweeteners would lead to higher consumption of emulsifiers; therefore, sweeteners themselves might not be confounders, and any possible confounding effect of this kind should be covered by adjusting for ultra-processed foods consumption.

2. The authors adjusted for dietary pattern scores, but those are not justifiable. A better approach was taken in the previously published manuscript .

+++ We have changed our adjustment approach and modified the manuscript throughout (Methods, Results, Tables and Figures). Please see below.

3. The authors aggregated all emulsifiers except the one assessed as the primary exposure, but makes not much sense.

+++ We have now taken out mutual adjustment for emulsifiers from the main model, and only tested it in sensitivity analyses.

In addition, in the report of artificial sweeteners, the authors failed to find consistent associations in their sensitivity analysis pertaining to dietary assessment methods. However, at this time the authors did not conduct such a careful analysis.

As such, the authors did the analyses parsimoniously, notable because of my familiarity to the previous paper. A small bias may give meaningful impact to the authors' interpretation because the observed associations were rather small.

+++ We have now changed our adjustment approach and performed additional sensitivity analyses. All models are now based on participants having at least 3 dietary records, and follow-up starts 2 years after the date of enrolment to comply with the Reviewers’ comments (please see below). This has been modified in the Methods starting line 222 and throughout the manuscript.

Main model was adjusted for age (time-scale), sex, BMI (continuous, kg/m²), height (continuous, cm), physical activity (categorical IPAQ variable: high, moderate, low), smoking status (never smoked, former smoker, current smokers), number of smoked cigarettes in pack-years (continuous), educational level (less than high school degree, <2 y after high school degree, ≥2 y after high school degree), number of dietary records (continuous), family history of cancer (yes/no), energy intake without alcohol (continuous, kcal/d), daily intakes of alcohol (continuous, g/d), lipids (continuous, g/d), sugars (continuous, g/d), sodium (continuous, g/d), fibre (continuous, g/d), consumption levels of fruits and vegetables (continuous, g/d), red and processed meats (continuous, g/d), and dairy products (continuous, g/d). Breast cancer models were additionally adjusted for oral contraception (yes/no, in total and premenopausal models only), age at menarche (never, <12 y, ≥12 y), number of biological children (continuous), age at first biological child (no child, <30 y, ≥30 y), menopausal status at baseline (premenopausal, postmenopausal, in total models only), hormonal treatment for menopause (yes/no, in total and postmenopausal models only).

In sensitivity analyses, based on the main model, model 1 included a mutual adjustment for the rest of emulsifiers (continuous, mg/d), model 2 included adjustment for the proportion of UPF in the diet (in % weight, continuous), model 3 included adjustment for total artificial sweeteners (continuous, mg/d), and model 4 included repeated emulsifier data using the average of all available 24h dietary records throughout the follow-up of each participant (up to 62 records per participant) (eTable4). We have also performed any versus none models (eTable5), and tested associations with patterns of emulsifiers (eTable6), as suggested by the Editorial board. Overall, main results were similar across these sensitivity analyses, supporting the robustness of the findings.

The presentation of the main findings needs to be improved, not indicating how the associations could be sensitive to different sets of covariates modelled.

+++ The sensitivity analyses described above now illustrate the stability of the results across different sets of covariates modelled and various methodological choices. We have also reported methods and results of sensitivity analyses in the Appendix (eMethods, eResults, eTables 4, 5 and 6).

Overall, major revisions are required including re-analyses.

+++ All additional analyses requested by the Editors and Reviewers have been performed.

-----------------------------------------------------------

Comments from the reviewers:

Reviewer #1: See attachment

This paper examines the association between consumptions of certain food additives and incident cancer in a French cohort.

I was asked for a statistical report and I interpret that to include all aspects of the design and conduct of the study.

+++ We sincerely thank Dr Dewey (Reviewer 1) for his insightful comments. All have been addressed below.

Points of detail

Page 2 To make the abstract self–contained I would say what ‘these associations’ are.

+++ We apologise. This has been amended now, line 36.

Page 2 Later the authors use a more conventional notation for means and standard deviation and I suggest that would be better here too.

+++ This has been corrected, page 2.

Page 4 Saying the first is a bit of a hostage to fortune. The more cautious tone of page 6 would be better.

+++ We have toned down this statement, in the Introduction pages 5 and 6 and throughout the manuscript.

Page 8 Providing some summary of the method for eliminating under– reporting would be beneficial or at least call out where it is expanded on in the supplement. Eliminating 16.5% of the cohort seems a rather large loss which needs more discussion especially if it is non–random. What covariates predict it? Have the authors considered multiple imputation for these missing values?

+++ We have now added details in the Methods section and called out where the full methodology is described in the supplement. We have also discussed how this phenomenon is frequent in dietary surveys and cohorts and the importance and relevance of excluding these participants in nutritional epidemiology. Multiple imputation was performed for all covariate data (physical activity level, level of education, smoking, BMI) but not for dietary data. Dietary data are complex and highly intercorrelated. Therefore, imputing data only for energy intake only without imputing for all food groups, macro and micro nutrients, vitamins, minerals, and food additives is not optimal. As our main exposure is a dietary exposure, we have chosen to exclude under-reported to avoid imputing for all dietary variables used in our models, to limit classification bias in the main exposure variables. This is the most frequently used strategy in other cohorts when analysing dietary data, such as the UK Biobank (1), EPIC (2) and the Nurses’ Health Study (3).

The discussion now reads:

*‘**Lastly, as classically observed in nutritional epidemiology studies, a significant sub-sample of the cohort (17%) was flagged as energy under-reporters and were excluded from the final sample. Different reasons might explain this phenomenon, mainly social desirability bias, but also entry errors while declaring their dietary intakes. These participants declare abnormally low and implausible energy intakes in absence of any specific restrictive diets. Indeed, participants were asked whether they were following any caloric restriction; in that case, they were included back in the analyses. This ensured that flagged under-reporters have true incoherent reporting, and must be excluded. The prevalence of energy under-reports in our study (17%) is in the range of those reported in other similar studies around the world: for instance, the prevalence of under-reporters ranged between 3 and 20% in the multicentric European EPIC cohort [61], 25.1% in the American NHANES study [62], and 18% in the Norwegian Breast Cancer Screening Program [63]. In the nationally representative INCA 3 study conducted in 2016 by the French Food Safety Agency [64] 18% of adult participants were identified as under-reporters using the Black method. Under-reporters in our cohort were older and were more inclined to be male and current smokers, to have a higher BMI and alcohol intake, and a lower educational level and monthly income [65]. Although their exclusion may limit the generalizability of the findings, it was necessary, in order to avoid important exposure classification bias.’*

Page 10 I know this is very pedantic but there are only two tertiles, what the authors have here is three tertile categories. Why not just call them low, medium and high in the same way as the other variables are split none, low and high?

+++ We have now renamed the categories as “low, medium and high” through the text, tables and appendix.

Page 10 Good to see the package properly cited (and other packages in the supplementary material). What about the competing risk analysis package?

+++ Thank you. No specific package was used for the competing risk analysis. This has been handled during the data management phase by having a variable related to the label of the first incident cancer, that was used for the analyses to create the censoring variable used in cause-specific Cox models.

Page 12 Is the test for trend a test for linear trend? Or is this what is explained in eMethod6? An extra sentence here would help or in the methods section.

+++ We apologise for this omission. This is indeed different and we have added an information about it in the Methods. *‘P-trends were calculated by handling the categorial variable as an ordinal score.’*

Page 23 Is the statement about participant and public involvement here based on the contribution of a formal advisory group? That would make it more convincing.

+++ No formal advisory group exists for the moment. Its creation, within the NutriNet-Santé cohort, is currently under consideration. Interactions with the public / patients on this topic emerged during the discussions and question-and-answer sessions following the conferences and debates led by our research team. This has been clarified in the statement.

Figure 1 I think pie charts are deprecated these days. See the references in the English Wikipedia page https://en.wikipedia.org/wiki/Pie_ chart

+++ We have replaced the pie chart by a bar chart.

Figure 3 I am afraid this is unreadable, at least in the version I received.

+++ This was linked to the PDF conversion of the file. We have now provided a higher quality image.

References

Perhaps give translations of the French titles so people know what they are missing?

+++ Done

eFigure1 I think this is too important to be put in the supplement.

+++eFigure1 has been moved to the main manuscript.

eMethod5 With an N of 100000 almost any correlation is going to achieve a statistically significant level so I think it is the overall shape here which needs examining. I did not see anything very concerning in the plots.

+++ p-values have been removed.

eFigures The labels for the axes are unreadable in most of these, perhaps as a result of giving very detailed information in them.

+++ Better versions are now provided.

Points of more substance

Mutual adjustment of additives

The authors have adjusted each additive for the effect of all the others (see page 10). This corresponds, as I see it, to the scientific question: What is the effect of this additive if all the others were held constant? The alternative, of course, is to present each additive unadjusted for the others. The choice here seems to depend on what the precise scientific question is.

•	If most people only consume some of the additives then the evidence relevant to their choice would come from the analysis unadjusted. The adjusted analysis adjusts for things which are not relevant to them.

•	If most people consume across the whole range of additives then the full model is most relevant to the choices they make.

The eFigure2 heatmap displays mostly weak relationships between additives suggesting that most people do not consume all or most of them (although there might be other factors at work here).

+++ Given the moderate correlations between emulsifiers as shown in the heatmap, and to comply with this comment, we have now removed mutual adjustment from the main model (and added a sensitivity analysis with a mutual adjustment for emulsifiers (model 1), and for artificial sweeteners (model 3) as requested by Reviewer #2 -> eTable4). Results were similar between these models.

UPF

The authors use the concept of UPF several times. I am not an expert in nutritional epidemiology but my understanding is that at least one of the ways of measuring it, NOVA, has some problems (Braesco et al., 2022). I see that the authors’ reference 61 used it.

+++ Although no classification is perfect, NOVA is the most extensively used classification for foods according to their levels of processing, and has been used in the framework of more than 70 prospective studies worldwide, some of which having led to updates in dietary guidelines (4). It has also been endorsed by a recent report of the FAO (5). We have recently co-authored a paper in Nature Food explaining the best practices to apply it in epidemiological studies (6). The level of detail of dietary data in NutriNet-Santé (>3500 generic food items + brands) guarantees a high level of reliability in the application of NOVA to our food nomenclature. We have now moved the adjustment for UPF to sensitivity analyses (model 2).

Summary

Fully reported account of a major piece of work. Apart from various points of detail I have raised a concern about whether the analysis is optimal for the scientific or clinical question.

Michael Dewey

+++ We thank the Reviewer once again for his positive and constructive feedback.

References

V Braesco, I Souchon, P Sauvant, T Haurogn´e, M Maillot, C F´eart, and N Darmon. Ultra–processed foods: how functional is the NOVA system? European Journal of Clinical Nutrition, 76:1245–1253, 2022.

Michael Dewey

Reviewer #2: Peer review of food additive emulsifiers and cancer risk

The authors have examined the associations of emulsifiers added to food and risk of cancer in a large cohort of French men and women. The authors have linked dietary intake taken from repeat 24 hour recalls to three food composition databases to determine the amount of a number of different food additives and related intakes with risk of cancer after an average follow-up time of 8.5 years. Overall, the study was well conducted, the exposure was measured well and follow-up for cancer outcomes were thorough. I have a few comments that I would like the authors to address:

+++ We thank Reviewer #2 for this positive comment and for his/her interesting advice. All points have been addressed below.

1. I am interested in the exposure - food additives and would like to know more. The authors have used a web-based 24 h dietary recall to assess food consumption. They have reported that this method has been validated. Has the relative validity of the intake of emulsifiers added to foods been compared against that from the diet diaries/records? What is the day-to-day variation in the intake of these food additives. How do the intakes obtained from this study compare to that from any other studies?

+++ Prior to the launching of the NutriNet-Santé cohort in 2008-2009, two studies have been performed to validate the web-based dietary records against biomarkers: one against plasma biomarkers (7) suggested that this tool provides reasonable estimates of true intake of fruits, vegetables, fish, beta carotene, vitamin C, and n-3 fatty acids. The other one against urinary biomarkers (8) showed that these web-based dietary records appear to be valid for estimating usual intakes of proteins and potassium. Besides, the self-administered web-based dietary record tool has also been validated against an interview by a trained dietician showing high correlations for all food groups, energy, macro and micro-nutrients (9); however, these validation studies did not specifically focus on food additives. This has been clarified in the Methods page 8.

Nevertheless, the accuracy of the qualitative and quantitative food additive exposure assessment of the NutriNet-Santé cohort represents an important strength of the study, thanks to detailed and repeated 24h-dietary records, links to multiple food composition databases (OQALI (15), Open Food Facts (16), GNPD (17), EFSA (18), and GSFA (19)), ad-hoc laboratory assays, and dynamic matching to account for reformulations of industrial food items over time (20). This pipeline is a pioneer procedure as the available data from surveillance and monitoring studies do not have details regarding commercial brands. Validation of additive exposure versus plasmatic or urinary biomarkers would be ideal, however, it can be challenging, and even impossible for the majority of food additives, including most emulsifiers, due to the lack of measurable biomarkers, since most additives are metabolized into substances that are non-specific and/or ubiquitous in human biofluids. We are currently working with the International Agency for Research on Cancer to develop a pipeline for measurement of urinary biomarkers of exposure to Tartrazin (E102), Norbixin (E160b), Sodium erythrobate (E316), Acesulfam-K (E950) and Steviol (E960) (i.e. additives for which metabolites could be specific enough to serve as intake biomarkers). This will contribute to further increase the confidence in the capacity of our dietary assessment tool in evaluating the exposure to food additives.

Intakes of emulsifiers in our study were lower than those reported in the EFSA opinions using simulation scenarios based on maximum permitted levels, and no brand-specific data (10–13), and were of the same order of magnitude as those reported in the American Cancer Prevention Study-3 (CPS-3) Diet Assessment Sub-Study, using brand-specific qualitative data coupled with simulations for quantitative data (4.2 g/day of total emulsifiers in our study, versus 1.9 g/day of total emulsifiers in CPS-3) (14). We have added these elements to the discussion page 16.

The average day-to-day variation coefficient of total emulsifiers in our study was 69%. Intra-individual variability might be partly linked to 1) changes in additive composition: this has been accounted for by the dynamic matching that we performed to integrate product reformulations and/or 2) changes in food consumption: our main analysis now includes only participants with at least 3 dietary records, with a mean number of record days = 6.0 (SD 3.1). In addition, we have now added a sensitivity analysis using the average of all available 24h dietary records throughout the follow-up of each participant (up to 62 records per participant, eTable4, model 4), which did not substantially modify main results.

2.	Was there any other further measurement of diet throughout the follow-up period? If not why and what implications might this have for the results of the study?

+++ Participants are invited to provide 3 24hr dietary records every 6 months. We chose to consider averaged data from the first two years of follow-up as a proxy for dietary habit, also in order to ensure having a prospective design and a sufficient time lapse between the dietary record and the date of cancer diagnosis. However, to account for possible variation throughout the follow-up period, we have now added a sensitivity analysis averaging all available dietary records during the participant’s entire follow-up period (up to 62 dietary records per participant) (eMethod5, eTable4 model 4).

3.	Dietary intake was collected over a period of two years? Did follow-up of the cohort begin before or after this time. It would seem sensible to begin follow-up after the data collection period ended.

+++ We started follow-up with dietary data collection as we hypothesised that baseline dietary data reflected usual dietary habits happening at the enrolment period but also years before, as usually hypothesized in nutritional epidemiology. In order to comply with this comment, we now start the follow-up at the end of the dietary data collection period, i.e., two years after the date of enrolment in the cohort. This has been modified in the Methods page 11.

4.	The method of ascertaining outcomes in this study appear to be robust and reliable. Did the study authors obtain any further information about the cancers e.g. the stage and grade?

+++ Data about stage and grade is progressively being collected and coded (depending on our funding capacity for the physician in charge). So far, data was available for a subsample of the cases (1414 cases/2604). The most frequent breast cancer subtypes were oestrogen-positive (ER+) and progesterone-positive (PR+) (85 and 75%, respectively), while triple negative breast cancers represented 10% of all breast cancer cases. At diagnosis, breast cancer was localized, advanced and metastatic in 69.6%, 28.9% and 0.2% of cases, respectively. As regards prostate cancer, 42% of cases were low-risk tumours (Gleason score 6 and below), 45% intermediate risk (Gleason score 7), and 13% high-risk tumour (Gleason score 8 and above). We have added this information to the Results section page 14.

5.	I am wondering how meaningful the outcome of total cancer is in this population when there are a high proportion of breast cancer and then almost half of the cancers are classified as "other". Going back to the introduction and trying to form some sort of hypothesis with risk of cancer, it would seem that those that affect the digestive tract would be most plausibly linked with intake of additives, but only colorectal cancer is mentioned but was not related to intake of additives (as the authors have stated there are small numbers of cases of colorectal cancer. I am just trying to understand how the hypothesis links to the methods and then to the findings.

Recent in vitro / in vivo experimental studies suggested detrimental effects of food additive emulsifiers such as alterations to the gut microbiota (15–17) and increased low-grade inflammation (16–19). Microbiota dysbiosis and chronic inflammation may potentially lead to higher risks of gut diseases (including inflammatory bowel disease), but are also involved in the aetiology of many other chronic diseases, including extra intestinal cancers (20,21). We have added these elements to the Introduction page 6.

In addition, we have now reported in detail the number of cancer cases, besides breast, colon and prostate cancers, page 14.

6.	While the authors have shown a p for trend for the tertiles of additives and hazard rate of cancers, most of the associations don't appear follow a dose-response relation with the hazards of cancer very similar between the second and the third tertile. Some comment on this would be helpful.

+++ In restricted cubic spline curves (eFigure3), p-values for non-linearity tests show mostly linear trends. However, for some associations, non-linear relationships are suggested by the plots (e450, e471, e440 and e500 with premenopausal breast cancer, e412 with prostate cancer, e415 with overall and breast cancer). This might be linked, for instance, to saturation of receptors, such as the Toll-like receptor 4, mediator of intestinal inflammation that could be induced by carrageenan exposure (22). As the exposure to emulsifiers increases, the receptors may become saturated, limiting the emulsifier’s ability to exert further effects, leading to a plateau in the response. This has been added to the results page 15, and the discussion page 18.

Reviewer #3: Thank you for this opportunity to review this important paper from the NutriNet Sante cohort. Many studies have investigated dietary associations between UPF and health/disease, therefore I am delighted to see an analysis that is able to investigate specific food components rather than only UPFs. NutriNet Sante is one of the only cohort studies that collects dietary data at the food item and brand level, and given emulsifier prevalence is subject to wide brand-related variations, this is crucial to understanding emulsifier/health interactions. Therefore, although the study is (yet another) re-analysis of cohort data, it is in fact highly novel (there are almost no cohort analyses of emulsifiers and health) and rigorous (almost no other cohort could analyse such data).

However, there are some major limitations that must be addressed, both in how data are analysed and how methods and results are reported.

+++ We thank Reviewer #3 for his/her positive comment, and insightful advice. We addressed all points below.

(1)	The methods section of this paper is similar to all other NutriNet Sante papers. Which is of course inevitable and to be expected. However, care should be taken to report methods (or the absence of methods) of relevance to the specific research question in the specific paper, especially in relation to dietary assessment methods.

a.	For example, Line 154-156 - "The web-based questionnaires used in the study have been tested and validated against both in-person interviews by trained dietitians, and urinary and blood markers.[34,35]"

My apologies I am not familiar with the two validation papers, so the following comment may be incorrect, however, I would guess that the validation papers relate to validity in measuring energy, macronutrients, and possibly micronutrients?

But I imagine the 3x 24 h recall's have not been validated to measure emulsifier exposure (ie the exposure of interest in this paper)? Just because a 24 h recall is accurate at measuring intakes of E, prot, fiber etc, does not mean it is accurate at measuring emulsifier exposure. This generic statement should be heavily nuanced for example - have you validated the method for measuring emulsifier intake? (eg against dietitian assessment, 7-day food diary or a stool biomarker e.g. CG or CMC?). Im not expecting this to be done for the purposes of the revision, but it is essential that nutritional epidemiologists are critical of their dietary assessment methods. So if it is not done, please say explicitly that it has not been validated for measuring emulsifier exposure, and please tone down the assertion that your dietary methods are validated, when they possibly may not be for the exposure of interest in this paper.

+++ Indeed, prior to the launching of the NutriNet-Santé cohort in 2008-2009, two studies have been performed to validate the web-based dietary records against biomarkers: one against plasma biomarkers (7) suggested that this tool provides reasonable estimates of true intake of fruits, vegetables, fish, beta carotene, vitamin C, and n-3 fatty acids. The other one against urinary biomarkers (8) showed that these web-based dietary records appear to be valid for estimating usual intakes of proteins and potassium. Besides, the self-administered web-based dietary record tool has also been validated against an interview by a trained dietician showing high correlations for all food groups, energy, macro and micro-nutrients (9). However, these validation studies against biomarkers and dietitian interviews did not specifically focus on intakes of food additives. This has been clarified in the Methods page 8.

Nevertheless, the accuracy of the qualitative and quantitative food additive exposure assessment of the NutriNet-Santé cohort represents an important strength of the study, thanks to detailed and repeated 24h-dietary records, links to multiple food composition databases (OQALI (23), Open Food Facts (24), GNPD (25), EFSA (26), and GSFA (27)), ad-hoc laboratory assays, and dynamic matching to account for reformulations of industrial food items over time (28). We fully agree with Reviewer #3 that validation of additive exposure versus plasmatic or urinary biomarkers would be ideal, however, it can be challenging, and even impossible for the majority of food additives, including most emulsifiers, due to the lack of measurable biomarkers, since most additives are metabolized into substances that are non-specific and/or ubiquitous in human biofluids. We are currently working with the International Agency for Research on Cancer to develop a pipeline for measurement of urinary biomarkers of exposure to Tartrazin (E102), Norbixin (E160b), Sodium erythrobate (E316), Acesulfam-K (E950) and Steviol (E960) (i.e. additives for which metabolites could be specific enough to serve as intake biomarkers). This will contribute to further increase the confidence in the capacity of our dietary assessment tool in evaluating the exposure to food additives.

We have now discussed this point in the limitations section page 18.

b.	Line 204 - This study included participants from the NutriNet-Santé cohort who completed at least two 24h-dietary records during their first two years of follow-up/…" Please clarify. In the dietary methods you say that every 6 months 3x 24 h recalls were requested. So in line 204 do you mean they were eligible if they:

i.	Conducted 3 x 24 recalls in Jan 2018 and then 3 x 24 h recalls in Jan 2019? (ie that the are eligible if they completed TWO PERIODS of 3x 24-h recalls) OR

ii.	Conducted 1 x 24 recall in Jan 2018 and then 1 x 24 h recall in Jan 2019? (ie they are eligible if they completed ONLY TWO random 24 h recalls ever).

If it is (i) then great, but please clarify this as it does not read like this. If it is (ii) then this is a very, very weak methodology. The CV of emulsifier intake has, to my knowledge, not been previously measured but I would imagine it was very, very high due to wide variations in food types and emulsifier types in different foods. Thus emulsifier intake, with a high CV, would require many many days of measurement. Certainly 2x 24h recalls would be wholly inadequate.

You need only to look at Table 2 to see very low intakes of individual emulsifiers (often less than 20 mg/d!!) and yet with very, very high SD. Therefore, how could 2 x 24 h recalls ever sufficiently accurately measure intake.

If it is (ii) then I would strongly request the group consider more stringent criteria for eligibility, 2 x 24 h recalls really will have very little accuracy. And whilst I understand this is an epidemiological study and data were used to compute tertiles of intake, the method to measure these should be optimised. If it is (ii) then please be more stringent with your cut off for the eligible number of 24-h recalls.

+++ We apologise if this statement was unclear. The initial version of the manuscript included participants with at least 2 days of 24h dietary records. Of note, this only represented 7.6% of the cohort, all others having 3 or more days of 24h-dietary records. This methodological choice for the main model represents a compromise between data precision, limitation of selection bias and maximisation of statistical power. It is what has been applied in previous NutriNet-Santé analyses on ultra-processed foods or artificial sweeteners and various health outcomes (29–34). However, in order to comply with Reviewer#3’s comment, we have now included only individuals with at least 3 24hr dietary records during their first two years of participation, to increase the precision of intake data (mean number of dietary records per participant = 6.0 (SD 3.0)). Modifications have been made throughout the manuscript and supplementary material. We also conducted a sensitivity analysis by averaging all available dietary records during the participant’s entire follow-up period (up to 62 dietary records per participant), which did not substantially modify main results (added in eMethods5, eTable4 model 4).

c.	You report that on average 5>5 24-h recalls were completed. For the reason stated above, the mean is important but does not give the full picture. Therefore please ALSO report the numbers with only two (which you will hopefully now exclude), three, four, five…. And then more than five. This will show the seriousness of the problem of the raw dietary data collection described above.

+++ In addition to our answer to the previous point and as done in some of our previous papers (e.g. on artificial sweeteners and CVD risk (31)), we have now provided the distribution of the number of dietary records per study participants in eMethod1.

d.	Case ascertainment - "physician expert committee validated every major health event after reviewing the participants' medical records and collecting additional information from the participants' treating physicians or hospitals". Systems may well be different in different countries, but are you saying that for every patient who reported cancer in the questionnaires, that a physician checked the "hospital medical records" for these? this is how this reads. If so then wonderful!, but I just wanted to check, please clarfy as "medical records" cold mean hospital records, or medical questionnaires, and itisn't clear. Im sure you review hospital records which is great, so lets make sure everyone knows how great this approach is.

+++ Indeed, a physician of our team is dedicated full time to the collection and checking of medical records of participants each time a new cancer diagnosis is declared by a participant. They refer to other physicians of the medical pole for discussion in case of doubt. The documents are indeed hospital records. We have amended the text accordingly. Thank you.

(2)	Adjustments and data presentation - the series of adjustments performed is, again, somewhat generic and not specific to the requirements of this paper (cancer). Examples include:

a.	Why were total lipid intake adjusted, for all cancers? What was your scientific and nutritional rationale? Are their known associations of total lipid intake for all cancers, and each of the cancers analysed?

+++ We adjusted for lipids since emulsifiers have lipophilic properties, they might bind to dietary lipids which could therefore interfere with their bioavailability or mechanism of action. The role of lipid exposure in carcinogenesis is still unclear but suggested (35). Lipid intake (as well as sugar intake) is also involved in obesity aetiology, itself a risk factor for many cancer locations (36). Besides, lipids are also involved in tumour progression and play a key role in tumour microenvironment (37). We have added this precision to the methods section page 11.

b.	For CRC why was total fiber intake not adjusted for? Fiber has a well described association with lower risk of CRC. Intakes are likely to be lower in those with higher emulsifier intake (although I don't know this), although your data in Table 1 suggest higher fibre in lower emulsifier groups). Adjusting for healthy eating pattern is insufficient to adjust for fiber intake. So fiber must be adjusted for in the CRC model.

I can see a sensitivity analysis has been performed, which includes total fibre, but why is the strongest nutritional associated cancer cause not included in the primary model in the primary paper, rather than in a sensitivity analysis?

How different is the data? (sorry I cant see fig 3 on the pdf it is very low quality, and I currently cannot download the actual PNG file. I would prefer explicit summary of the data for model 1 in the MAIN paper for CRC at least, as it adjusts for fibre. It would only be one extra paragraph.

+++ In the previous round of analyses, adjustment for fibre did not significantly change the hazard ratio estimates.

We have now changed our adjustment strategy and replaced adjustment for dietary patterns by adjustment for more specific food groups, nutrients and components such as dietary fibre. The main model is adjusted for the available factors known or suspected to be associated with emulsifier intake or diet and with cancer risk: anthropometric and sociodemographic factors (age (as timescale), sex, educational level), lifestyle factors (smoking status, number of pack-years, physical activity level, alcohol consumption), anthropometrics (BMI, height), family history of cancer, number of dietary records, energy intake without alcohol, dietary factors with suspected or known impact on the risk of one or several cancer types (intakes of sodium, fibre, sugar, total lipids (rationale in the earlier question), as well as consumption levels of fruits and vegetables, dairy products, and red and processed meats (36)). Breast cancer models were additionally adjusted for oral contraception, age at menarche, number of biological children, age at first biological child, menopausal status at baseline, and hormonal treatment for menopause. Several sensitivity analyses were then conducted to test different adjustments, illustrating the high stability of the results (eTable4).

c.	I think the major tertiles displayed (eg in table 1) were based upon total emulsifier intake. However it would appear that all risks were calculated against intakes of the emulsifier groups or individual emulsifiers only - is that right? (that makes sense as individual emulsifiers have different impacts on health).

But does it therefore mean that the reader cannot see the actual intakes of each emulsifier group or each individual emulsifier that make up Tertile1, T2 and T3? Maybe this is in Supplementary data (my apologies I cannot currently view this). But if it is not then if the reader is to every interpret the risk of each emsulfier group or each emulsifier, then we need to know the intakes in T1, T2, T3 for each emulsifier group and each emulsifier… sometimes you must be making comparisons of tertiles with very very low intakes, which concerns me around accuracy, but also application. We need to see what these values are for each tertial for each emsulfiier group and each emulsifier.

+++ Cut-offs for tertiles (or categories) of each specific emulsifier (as used in Cox models) are displayed in the footnotes to eTable 2 and 3 in the Supplementary material, for readability purposes. We have now referred to this section in the Results page 13. We have also added a table providing mean daily intake of each emulsifier by category of intake: none/low, medium, high (eTable2).

(3)	Emulsifier quantification 177-180 - more detail on the emulsifier quantification are required. This is the central strength of this paper, yet is reported very briefly. If there is insufficient text available then it must be summarised in supplementary methods.

The three approaches used lead to increasing levels of inaccuracy. It is impossible, as described, to estimate the level of inaccuracy, as it is unclear for how many foods and how many emulsifiers was used for each method. Eg were most estimates foods measured using the very accurate method 1? Or were most using the very inaccurate method 3? Examples of required details include, but are not restricted to:

a.	"ad-hoc laboratory assays quantifying additives in specific food items (N=2,677 food additive pairs analysed" - please provide detail

i.	does this mean the presence of ALL emulsifiers in 2677 foods?

ii.	What are food-additive pairs? This is crucial information.

iii.	For example, assuming conservative estimate of 5000 UPF/emulsifier-containing foods and >60 emulsifiers, that is 300,000 lab analyses if done completely via method 1 (which is therefore clearly not happened)… it is crucial to report how many were laboratory analysed and for what. Therefore For the above please report:

iv.	How many food items were analysed

v.	and for their content of how many different emsulifiers?

b.	"doses in generic food categories provided by the European Food Safety Authority (EFSA)" - are you saying that EFSA have provided you with the actual quantities of emulsifier in broad food groups? How did you get this information? This is not available for general release.

Therefore For the above please report:

i.	How many food groups were provided

ii.	and for their content of how many different emulsifiers?

c.	For the above, where a food item (eg a cheese sauce) does not have any laboratory data, and so you have to use method 2, can you confirm you would only use "food group" assumptions from EFSA (eg for cheese sauces) ONLY if the specific food item you were calculating (ie the specific brand of cheese sauce) did actually contain emulsifiers in the ingredients list. Otherwise blanket assumptions will result in some food items with no emuslfiiers being coded as containing them… and some food items with emulsifiers being coded as not containing them.

d.	"generic doses from the Codex General Standard for Food Additives (GSFA).[41]"

i.	Please expand - a general reader will nt understand this.

ii.	Again, for the above please report:

iii.	How many food items was this used

iv.	and for their content of how many different emulsifiers?

These should be addressed in the rebuttal and briefly in the manuscript or supplementary information.

+++ Thank you for these precise questions that allowed us to improve the detail of information provided.

The strength of our methodology relies in the precise qualitative assessment of additive exposure, i.e., presence/absence of a specific emulsifier in the food consumed. This unique level of detail is permitted by the fact that commercial names/brands of industrial product consumed were collected and matched with Open Food Facts, Oqali and GNPD databases providing the ingredient list and thus, presence of the specific emulsifier), at the time when the product was consumed. Therefore, we only attribute a non-null dose of a specific additive to a given product declared by a participant if this specific product contains this specific additive.

Then, the quantitative assessment of the doses of additives in the products which contain a specific additive is challenging since (unfortunately for public health research!) manufacturers are not compelled to declare this information on the packaging. Hence the 3-step method used to assess doses in our cohort. In all, in the framework of the ADDITIVES project, we performed 2677 quantified analyses, corresponding to a total of 61 food additives in 196 different (generic) food items. “Pairs” (i.e. a specific additive in a specific food vector) selected for laboratory assays corresponded to the most frequently consumed and most emblematic commercial food/beverage items for a given additive. Specifically, for emulsifiers, we had access to 501 laboratory quantified analyses corresponding to 9 emulsifiers (E322, E339, E340, E341, E343, E440, E450, E451, E452) in 37 (generic) food items (several commercial brands were tested per food item, e.g., in the case of milk chocolate, milk chocolate with nuts, creamy desserts, omega-3 enriched margarines, sausages, jams, chocolate mousse…). In addition to the assays carried out by certified laboratories, which were sent to us by the consumer association UFC Que Choisir, we contacted two companies (Mérieux & Eurofins) and the Direction Générale de la Consommation, de la Concurrence et de la Répression des Fraudes (DGCCRF) to carry out these assays. Only the additives listed in their catalogue could be measured. In case data was not available from this source, EFSA and GSFA doses were only applied if the specific food item did actually contain the specific emulsifiers in the ingredients list. We used 1497 emulsifier data from EFSA (data available online in each EFSA Opinion + transmission of specific information by EFSA following an official Public Access to Document request PAD 2020/077), related to 58 food additive emulsifiers present in 237 food categories. EFSA collects many information from manufacturers related to their specific commercial products but for confidentiality reasons, only transfers information for generic food items or food groups (no brand-specific data). As regards GSFA, we used 5352 emulsifier data concerning 45 food additive emulsifiers coming from 226 food categories. As for EFSA, data from GSFA are not brand-specific but relate to generic food items or food categories. We have added these clarifications to the methods section page 9, and eMethod2.

e.	Table 2 is excellent, thank you for detailed intake data.

+++ Thank you.

(4)	Line 165 - under and over-reporting:

a.	Is the Black method the Golderg method? If so this is the more commonly used term. If not please clarify.

+++ As detailed in the supplementary material, participants who under-reported their energy intake were excluded from the analyses and were identified using the method from Black (38,39) based on the original method developed by Goldberg et al.(40). This method relies on the hypothesis that the maintenance of a stable body weight requires a balance between energy intake and expenditure. The equations developed by Black account for the reported dietary energy intake, basal metabolic rate (calculated using Schofield’s equations)(41), sex, age, height, weight, number of dietary records, physical activity level (PAL), and intra/inter-individual variability. We have clarified in the main text page 8 that the method was based on the original approach proposed by Goldberg et al.

b.	Also, the numbers excluded due to under- and over- reporting should be reported in the main text of the paper.

+++ This has now been reported in the Methods section page 8.

c.	I see from supp figures that >21,000 were excluded due to under-reporting. Given this is based upon(if Goldberg, or summarised by Black) then this should be 2 SD beyond the EI(measured)/BMR(est). If normally distributed this would be 2.5% of the population predicted to be under-reporters, but in fact it is 21,423/129,571 - which is >16% of the population under-reporting.

I realise you cannot do anything about this, but it really is huge and I ask you to consider moving forward, why your dietary assessment tool may be quite so inaccurate. There is no evidence that 16% of the population intentionally under-report, so much of this is likely the inaccuracy of th 24h recalls.

+++ Energy under-reporting is a classical phenomenon in nutrition studies and cohorts. In fact, the prevalence of energy under-reports in our study (16.5%) is in the range of those reported in other studies around the world using similar methods: for instance, the proportion of under-reporters was 25.1% in the American NHANES study (42), and 18% in the Norwegian Breast Cancer Screening Program (43). In the nationally representative INCA 3 study conducted in 2016 by the French Food Safety Agency (44) 18% of adult participants were identified as under-reporters using the Black method. Different reasons might explain this phenomenon, mainly social desirability bias, but also input errors while using the online tool. These participants usually declare abnormally low energy intakes, implausible even in case of restrictive diets. Participants were also asked whether they were following any caloric restriction; in that case, they were included back in the analyses. This ensured that flagged under-reporters have true incoherent reporting, and must be excluded. Although their exclusion may limit the generalizability of the findings, it was necessary, in order to avoid important exposure classification bias. The detailed methodology for under-energy reporting detection is in the supplementary material.

We have now discussed these points in the manuscript, pages 19-20:

*‘Besides, ≈17% of the cohort was excluded due to energy-underreporting assessed via a standard method* (45)*, in order to eliminate true reporting errors in the absence of any restrictive diet. This proportion was consistent with the one observed in other studies, e.g. 25.1% in the American NHANES study* (42)*, and 18% in the Norwegian Breast Cancer Screening Program* (43)*. In the nationally representative INCA 3 study conducted in 2016 by the French Food Safety Agency* (44)*, 18% of adult participants were identified as under-reporters using the Black method applied in the present study.’*

d.	Supp fig 1 implies there were no over-reporter. Which is very odd. Is this actually correct. Again, please consider within your group the accuracy of your data collection tool.

+++ Several quality control operations were performed to account for over-reporting. Limitations in the online tool were set when participants reported the quantities of food consumed, aiming to alert them that the number they were about to enter was potentially an outlier, thereby encouraging double check and correction. Later on, during the data cleaning process, limitations were set per food category within one eating episode and per record for quantities; for instance, limitations for fruits were set for 3000 grams/day, 1500 grams/day for fish, 2000 grams/day for yoghurts, etc… if more than 10% of reported food items had outliers, then the full record was excluded. Otherwise, values were corrected to the maximum authorised values or standardised. We have added some clarifications in the Methods section page 8 and eMethod1.

MINOR

(5)	The abstract is poorly written - a shame for such a good study. It will be brilliant once ou make improvements, not limited to:

a.	The first sentence of the abstract is: "Objective: This study aimed to investigate THESE associations in a large population-based prospective cohort." Given this will be the first line on a paper "these" is meaningless. Please change.

+++ We apologise. The word “these” has been deleted and the first sentence has been rephrased, page 2.

b.	"Main outcome measures: Associations with incident cancer risk were assessed using multivariable Cox models" sentences are incomplete - association of cancer with what? How was incidence of cancer measured? (it is not mentioned in the abstract). This is the most detailed every analysis of emsulfiers and disease. Please show that early on.

+++ These elements have been added in the abstract.

c.	Pleas report average years of follow-up. Crucial in epidemiology but very low in some reports (but not here).

+++ This has been added in the “Participants” section of the abstract page 2.

d.	Conclusion "In this large prospective cohort, we observed direct associations between cancer risks (overall, breast, and colorectal) and…" no mention of CRC and risk are presented in the abstract.

+++ This has been corrected in the abstract, in line with updated results: "In this large prospective cohort, we observed direct associations between cancer risks (overall, breast, and prostate)…”.

(6)	E number nomenclature. In some places the E numbers are provided with a space between E and the number e.g. E 331 (see author summary - but also check throughout). The spaces should be removed throughout.

+++ Done

(7)	Line 100 "…dozens of food additive emulsifiers are found in thousands of daily-used processed food items (e.g. chocolate, pastries, but also ready-to-eat prepared fruits and vegetables). This is confusing to interpret. As written it sounds as though you are saying there are some foods with dozens of emulsifiers in them - which may grab incorrect media headlines.

Are you saying there are dozens of emulsifiers in the food chain? If so perhaps be more explicit e.g. The numbers of emulsifiers varies in the food chain depending upon national definitions used but can range from XXX in EU to XXX in US". Your own work and that of other national evaluations could be used.

+++ We have rephrased this statement.

*‘**As a consequence, they can be found in thousands of daily-used processed food items (e.g. chocolate, pastries, but also ready-to- eat fruit, vegetable or legume preparations) [16]. The number of authorised emulsifiers varies in the food chain globally, depending upon local definitions and regulations used, but can range from ≈60 in the European Union (EU) to ≈170 in the United States (US) [15].’*

(8)	Line 113 "…potentially leading to higher risks of gut diseases such as inflammatory bowel disease[23] and Crohn's disease.[24]" Crohn's is an IBD, therefore please rephrase (e.g. including…)

+++ Done

(9)	Human studies:

a.	Line 114-116, and Line 327-329. Its probably important to STRONGLY emphasise, in the interests of balance and scientific rigour, that the CMC doses used in this first in human study are super-physiological. For example you show intakes of CMC in your population are 3.9 mg/d, whereas the human study for which you emphasise changes in microbiome and inflammation used 15 g/d!! (almost 4000 times more)

+++ Thank you for stressing this point. It has been clarified in the manuscript pages 6 and 17.

b.	Discussion - Line 327 - states that a human study showed high doses of CMC impact microbiome - stating the dose was 15 mg/d. This is 3 log out!

+++ We apologise for this typo. We have corrected it in the manuscript page 17.

(10)	Discussion - the association of celluloses and breast cancer is interesting. Celluloses are also fiber and, although not all are included in the diet from plant foods, cellulose is widely consumed as part of the plant cell wall. Some comment should be added in the discussion about this apparently counter intuitive finding.

+++ We have added this statement to the discussion:

*‘**Some additive emulsifier substances can also be naturally occurring, such as pectins and celluloses, which are also fibre. Although this might seem counterintuitive given the protective role of fibre on cancer* (36)*, the additive form might exert deleterious health effects, due to the disruption of food matrix in industrial products containing added emulsifiers compared to plants and fruit, which might lead to different effects on human health.’*

Any attachments provided with reviews can be seen via the following link:

<https://www.editorialmanager.com/pmedicine/l.asp?i=841268&l=MOZV64NW>

__________________________________________________

In compliance with data protection regulations, you may request that we remove your personal registration details at any time. (Use the following URL: <https://www.editorialmanager.com/pmedicine/login.asp?a=r>). Please contact the publication office if you have any questions.

References

1.	Chang K, Gunter MJ, Rauber F, Levy RB, Huybrechts I, Kliemann N, et al. Ultra-processed food consumption, cancer risk and cancer mortality: a large-scale prospective analysis within the UK Biobank. eClinicalMedicine [Internet]. 2023 Feb 1 [cited 2023 Aug 30];56. Available from: https://www.thelancet.com/journals/eclinm/article/PIIS2589-5370(23)00017-2/fulltext

2.	Deschasaux M, Huybrechts I, Murphy N, Julia C, Hercberg S, Srour B, et al. Nutritional quality of food as represented by the FSAm-NPS nutrient profiling system underlying the Nutri-Score label and cancer risk in Europe: Results from the EPIC prospective cohort study. PLOS Medicine. 2018 Sep 18;15(9):e1002651.

3.	Wang L, Du M, Wang K, Khandpur N, Rossato SL, Drouin-Chartier JP, et al. Association of ultra-processed food consumption with colorectal cancer risk among men and women: results from three prospective US cohort studies. BMJ. 2022 Aug 31;378:e068921.

4.	Srour B, Kordahi MC, Bonazzi E, Deschasaux-Tanguy M, Touvier M, Chassaing B. Ultra-processed foods and human health: from epidemiological evidence to mechanistic insights. The Lancet Gastroenterology & Hepatology. 2022 Dec 1;7(12):1128–40.

5.	Monteiro CA, Cannon G, Lawrence M, Costa Louzada ML da, Pereira Machado P. Ultra-processed foods,diet quality, and health using the NOVA classification system. Rome, FAO [Internet]. 2019 [cited 2019 Sep 4]; Available from: http://www.fao.org/3/ca5644en/ca5644en.pdf

6.	Martinez-Steele E, Khandpur N, Batis C, Bes-Rastrollo M, Bonaccio M, Cediel G, et al. Best practices for applying the Nova food classification system. Nat Food. 2023 Jun 1;

7.	Lassale C, Castetbon K, Laporte F, Deschamps V, Vernay M, Camilleri GM, et al. Correlations between Fruit, Vegetables, Fish, Vitamins, and Fatty Acids Estimated by Web-Based Nonconsecutive Dietary Records and Respective Biomarkers of Nutritional Status. JAcadNutr Diet. 2016 Mar;116(2212-2672 (Print)):427–38.

8.	Lassale C, Castetbon K, Laporte F, Camilleri GM, Deschamps V, Vernay M, et al. Validation of a Web-based, self-administered, non-consecutive-day dietary record tool against urinary biomarkers. BrJNutr. 2015 Mar 28;113(1475-2662 (Electronic)):953–62.

9.	Touvier M, Kesse-Guyot E, Mejean C, Pollet C, Malon A, Castetbon K, et al. Comparison between an interactive web-based self-administered 24 h dietary record and an interview by a dietitian for large-scale epidemiological studies. BrJNutr. 2011 Apr;105(1475-2662 (Electronic)):1055–64.

10.	EFSA. Re-evaluation of xanthan gum (E 415) as a food additive [Internet]. 2017 Jul [cited 2022 Jan 30]. Available from: https://www.efsa.europa.eu/en/efsajournal/pub/4909

11.	EFSA, Younes M, Aggett P, Aguilar F, Crebelli R, Filipič M, et al. Re-evaluation of carrageenan (E 407) and processed Eucheuma seaweed (E 407a) as food additives [Internet]. 2018 Apr [cited 2022 Mar 30] p. e05238. Available from: https://onlinelibrary.wiley.com/doi/abs/10.2903/j.efsa.2018.5238

12.	Mortensen A, Aguilar F, Crebelli R, Domenico AD, Frutos MJ, Galtier P, et al. Re-evaluation of lecithins (E 322) as a food additive. EFSA Journal. 2017;15(4):e04742.

13.	EFSA. Re-evaluation of celluloses E 460(i), E 460(ii), E 461, E 462, E 463, E 464, E 465, E 466, E 468 and E 469 as food additives [Internet]. 2017 Sep [cited 2022 Jan 30]. Available from: https://www.efsa.europa.eu/en/efsajournal/pub/5047

14.	Um CY, Hodge RA, Tran HQ, Campbell PT, Gewirtz AT, McCullough ML. Association of Emulsifier and Highly Processed Food Intake with Circulating Markers of Intestinal Permeability and Inflammation in the Cancer Prevention Study-3 Diet Assessment Sub-Study. Nutrition and Cancer. 2021 Aug 6;0(0):1–11.

15.	Elmén L, Zlamal JE, Scott DA, Lee RB, Chen DJ, Colas AR, et al. Dietary Emulsifier Sodium Stearoyl Lactylate Alters Gut Microbiota in vitro and Inhibits Bacterial Butyrate Producers. Frontiers in Microbiology [Internet]. 2020 [cited 2022 Jan 29];11. Available from: https://www.frontiersin.org/article/10.3389/fmicb.2020.00892

16.	Naimi S, Viennois E, Gewirtz AT, Chassaing B. Direct impact of commonly used dietary emulsifiers on human gut microbiota. Microbiome. 2021 Mar 22;9(1):66.

17.	Viennois E, Bretin A, Dubé PE, Maue AC, Dauriat CJG, Barnich N, et al. Dietary Emulsifiers Directly Impact Adherent-Invasive E. coli Gene Expression to Drive Chronic Intestinal Inflammation. Cell Reports [Internet]. 2020 Oct 6 [cited 2022 Jan 4];33(1). Available from: https://www.cell.com/cell-reports/abstract/S2211-1247(20)31218-3

18.	Benard C, Cultrone A, Michel C, Rosales C, Segain JP, Lahaye M, et al. Degraded Carrageenan Causing Colitis in Rats Induces TNF Secretion and ICAM-1 Upregulation in Monocytes through NF-κB Activation. PLOS ONE. 2010 Jan 13;5(1):e8666.

19.	Um CY, Hodge RA, Tran HQ, Campbell PT, Gewirtz AT, McCullough ML. Association of Emulsifier and Highly Processed Food Intake with Circulating Markers of Intestinal Permeability and Inflammation in the Cancer Prevention Study-3 Diet Assessment Sub-Study. Nutrition and Cancer. 2021 Aug 6;0(0):1–11.

20.	Zitvogel L, Galluzzi L, Viaud S, Vétizou M, Daillère R, Merad M, et al. Cancer and the gut microbiota: An unexpected link. Sci Transl Med. 2015 Jan 21;7(271):271ps1.

21.	Huybrechts I, Zouiouich S, Loobuyck A, Vandenbulcke Z, Vogtmann E, Pisanu S, et al. The Human Microbiome in Relation to Cancer Risk: A Systematic Review of Epidemiologic Studies. Cancer Epidemiol Biomarkers Prev. 2020 Oct 1;29(10):1856–68.

22.	Bancil AS, Sandall AM, Rossi M, Chassaing B, Lindsay JO, Whelan K. Food Additive Emulsifiers and Their Impact on Gut Microbiome, Permeability, and Inflammation: Mechanistic Insights in Inflammatory Bowel Disease. J Crohns Colitis. 2021 Jun 22;15(6):1068–79.

23.	Observatoire de l’alimentation (Oqali). OQALI - Home page [Internet]. [cited 2020 Oct 22]. Available from: https://www.oqali.fr/oqali_eng/

24.	Open Food Facts [Internet]. [cited 2020 Oct 22]. Available from: https://world.openfoodfacts.org/discover

25.	Global New Products Database (GNPD). Banque de données mondiale de nouveaux produits, suivi des tendances nouveaux produits et innovations [Internet]. [cited 2020 Oct 22]. Available from: https://www.gnpd.com/sinatra/anonymous_frontpage/

26.	EFSA ANS Panel. (EFSA Panel on Food Additives and Nutrient Sources added to Food) - Scientific Opinion on the re-evaluation of aspartame (E 951) as a food additive. EFSA Journal 2013;11(12):3496, 263 pp. doi:10.2903/j.efsa.2013.3496. 2013;

27.	Food and Agriculture Organization/World Health Organization (FAO/WHO). Codex General Standard for Food Additives (GSFA, Codex STAN 192-1995) [Internet]. Codex Alimentarius Commission; 2019 [cited 2018 Sep 19]. Available from: http://www.fao.org/fao-who-codexalimentarius/sh-proxy/en/?lnk=1&url=https%253A%252F%252Fworkspace.fao.org%252Fsites%252Fcodex%252FStandards%252FCODEX%2BSTAN%2B192-1995%252FCXS_192e.pdf

28.	Chazelas E, Druesne-Pecollo N, Esseddik Y, Szabo de Edelenyi F, Agaësse C, De Sa A, et al. Exposure to food additive mixtures in 106,000 French adults from the NutriNet-Santé cohort. Sci rep. 2021 Oct 4;11(1):19680.

29.	Srour B, Fezeu LK, Kesse-Guyot E, Allès B, Debras C, Druesne-Pecollo N, et al. Ultraprocessed Food Consumption and Risk of Type 2 Diabetes Among Participants of the NutriNet-Santé Prospective Cohort. JAMA Intern Med. 2020 Feb 1;180(2):283.

30.	Srour B, Fezeu LK, Kesse-Guyot E, Allès B, Méjean C, Andrianasolo RM, et al. Ultra-processed food intake and risk of cardiovascular disease: prospective cohort study (NutriNet-Santé). BMJ. 2019 May 29;365:l1451.

31.	Debras C, Chazelas E, Sellem L, Porcher R, Druesne-Pecollo N, Esseddik Y, et al. Artificial sweeteners and risk of cardiovascular diseases: results from the prospective NutriNet-Santé cohort. BMJ. 2022 Sep 7;378:e071204.

32.	Srour B, Chazelas E, Druesne-Pecollo N, Esseddik Y, Edelenyi FS de, Agaësse C, et al. Dietary exposure to nitrites and nitrates in association with type 2 diabetes risk: Results from the NutriNet-Santé population-based cohort study. PLOS Medicine. 2023 Jan 17;20(1):e1004149.

33.	Debras C, Chazelas E, Srour B, Druesne-Pecollo N, Esseddik Y, Edelenyi FS de, et al. Artificial sweeteners and cancer risk: Results from the NutriNet-Santé population-based cohort study. PLOS Medicine. 2022 Mar 24;19(3):e1003950.

34.	Beslay M, Srour B, Méjean C, Allès B, Fiolet T, Debras C, et al. Ultra-processed food intake in association with BMI change and risk of overweight and obesity: A prospective analysis of the French NutriNet-Santé cohort. PLoS Med. 2020 Aug;17(8):e1003256.

35.	Snaebjornsson MT, Janaki-Raman S, Schulze A. Greasing the Wheels of the Cancer Machine: The Role of Lipid Metabolism in Cancer. Cell Metab. 2020 Jan 7;31(1):62–76.

36.	WCRF/AICR. Continuous Update Project Expert Report 2018. Recommendations and public health and policy implications. 2018;92.

37.	Vasseur S, Guillaumond F. Lipids in cancer: a global view of the contribution of lipid pathways to metastatic formation and treatment resistance. Oncogenesis. 2022 Aug 9;11(1):1–15.

38.	Black AE. Critical evaluation of energy intake using the Goldberg cut-off for energy intake:basal metabolic rate. A practical guide to its calculation, use and limitations. IntJObesRelat Metab Disord. 2000 Sep;24(0307-0565 (Print)):1119–30.

39.	Black AE. The sensitivity and specificity of the Goldberg cut-off for EI:BMR for identifying diet reports of poor validity. European Journal of Clinical Nutrition. 2000 May;54(5):395–404.

40.	Goldberg GR, Black AE, Jebb SA, Cole TJ, Murgatroyd PR, Coward WA, et al. Critical evaluation of energy intake data using fundamental principles of energy physiology: 1. Derivation of cut-off limits to identify under-recording. Eur J Clin Nutr. 1991 Dec;45(12):569–81.

41.	Schofield WN. Predicting basal metabolic rate, new standards and review of previous work. HumNutr Clin Nutr. 1985;39 Suppl 1(0263-8290 (Print)):5–41.

42.	Murakami K, Livingstone MBE. Prevalence and characteristics of misreporting of energy intake in US adults: NHANES 2003-2012. Br J Nutr. 2015 Oct 28;114(8):1294–303.

43.	Markussen MS, Veierød MB, Ursin G, Andersen LF. The effect of under-reporting of energy intake on dietary patterns and on the associations between dietary patterns and self-reported chronic disease in women aged 50–69 years. British Journal of Nutrition. 2016 Aug;116(3):547–58.

44.	Anses. Etude Individuelle Nationale des Consommations Alimentaires 3 (INCA 3). 2017.

45.	Black AE. Critical evaluation of energy intake using the Goldberg cut-off for energy intake:basal metabolic rate. A practical guide to its calculation, use and limitations. Int J Obes Relat Metab Disord. 2000 Sep;24(9):1119–30.
