# Supplementary material for: Food additive emulsifiers and cancer risk: Results from the French prospective NutriNet-Santé cohort
Source: PLoS Med. 2024 Feb 13;21(2):e1004338. doi: 10.1371/journal.pmed.1004338 (PMC10863884; doi:10.1371/journal.pmed.1004338)
Supplement: Attachment — Submitted filename: Response_R3.docx [file pmed.1004338.s005.docx]

Academic Editor Comments:

The abstract does not reflect the study findings well, and the current highlight seems partly misleading. The authors should revise text on their interpretations.

The authors conducted sensitivity analyses using repeated measures of their dietary data. First of all, the use of repeated measures in a longitudinal study would be not uncommon. Many cohorts with repeated measures accounted for them in their longitudinal analyses, such as Framingham Heart Study, Nurses’ Health Study, and Health Professionals Follow-up Study. Different numbers of repeats and different timing would not be problematic unless meaningful evidence for bias was present.

While detailed modelling approach was unclear and could be improved, the results in “model 4” in eTable 4 are valuable.

Then, the authors presented some results apparently inconsistent with the results highlighted in the abstract.

For example, the association of E415 with overall cancer showed inconsistent HRs (95% CI): 1.13 (1.02,1.25) in the abstract, and 0.96 (0.86, 1.06) in model 4 of eTable 4; Polyglycerol esters of FAs (E475) and breast cancer did it, too: 1.50 (1.05,2.15) in the abstract, and 1.24 (0.91, 1.71) in model 4 of eTable 4. Guar gum (E412) and prostate cancer did it, too: 1.39 ( (1.04, 1.87) in the abstract, and 0.70 (0.44, 1.12) in model 4 of eTable 4. Gum Arabic E414 and prostate cancer: 2.43 (1.14, 5.18) in the abstract, and 1.26 (0.85, 1.86) in model 4 of eTable 4. (etc.)

Those showed noticeable inconsistencies, and the inconsistency should be recognised by readers clearly. Despite the apparent failure to confirm the robustness of their findings, the authors said, “Overall main results were similar in sensitivity analyses” (Line 351-352). This interpretation is inappropriate or at least not compatible with the results.

The authors should apply the inevitable revisions as follows at least:

1) The authors should document the inconsistency in one of the secondary analyses in the main text (paragraph before Discussion).

+++ Thank you for your careful reading of our work and your advice. To follow these recommendations, we have now rephrased the way we report our findings, in the main text and the abstract by focusing on the associations that were statistically significant throughout all sensitivity analyses. The results section now reads (page 14-15):

“*In the main models, higher intakes of mono- and diglycerides of FAs (E471) were associated with higher risks of overall cancer (HR _high vs. low category_ = 1.15; 95%CI [1.04,1.27], p-trend=0.01), breast cancer (HR = 1.24; 95%CI [1.03,1.51], p-trend=0.04) and prostate cancer (HR = 1.46; 95%CI [1.09,1.97], p-trend=0.02). In addition, associations with breast cancer risk were observed for higher intakes of total carrageenans (E407 and E407a) (HR = 1.32; 95%CI [1.09,1.60], p-trend=0.009) and carrageenan (E407) (HR = 1.28; 95%CI [1.06,1.56], p-trend=0.01). In analyses by menopausal status, higher risks of premenopausal breast cancer were associated with higher intakes of diphosphates (E450) (HR = 1.45; 95%CI [1.04,2.02], p-trend=0.03), pectins (E440) (HR = 1.55; 95%CI [1.12,2.14], p-trend=0.008) and sodium bicarbonate (E500) (HR = 1.48; 95%CI [1.07,2.05], p-trend=0.01). No significant association was detected between studied emulsifiers and colorectal cancer risk (eTable4) in our study. Overall, these main results were similar in all sensitivity analyses (eMethod5, eTables5, 6 and 7 in S1 Appendix).*

*In addition, the following associations were observed in the main model but were not robust in all sensitivity analyses, especially when all dietary records available during follow-up (up to 62 records) were used to calculate emulsifier intakes (Figure 4 and eTable5 – model 4): 1) higher intakes of Xanthan gum (E415) were associated with a higher risk of overall cancer (HR in the main model = 1.13; 95%CI [1.02,1.25], p-trend=0.04); 2) higher risks of breast cancers were associated with higher intakes of polyglycerol esters of FAs (E475) (HR = 1.50; 95%CI [1.05,2.15], p-trend=0.02) and carob bean gum (E410) (HR = 1.19; 95%CI [0.99,1.43], p-trend=0.045); 3) higher intakes of total carrageenans (E407 and E407a) were associated with postmenopausal breast cancer (HR = 1.28; 95%CI [1.00,1.64], p-trend=0.04); 4) higher intakes of dipotassium phosphate (E340) were associated with a higher risk of premenopausal breast cancer (HR = 1.77; 95%CI [1.08,2.91], p-trend=0.03); 5) higher risks of prostate cancer were associated with higher intakes of Guar gum (E412) (HR = 1.39; 95%CI [1.04,1.87], p-trend=0.02), gum arabic (E414) (HR = 2.53; 95%CI [1.54,4.15], p-trend=0.009) and Beeswax (E901) (HR = 2.43; 95%CI [1.14,5.18], p-trend=0.03).”*

2) The authors should discuss the limitation of this study more elaborately by documenting the internal inconsistency according to the number of repeats to analyse.

+++ We have now discussed this inconsistency in the limitations section, as follows (page 20, lines 468-472):

“*Lastly, depending on the regularity of consumption of specific emulsifiers and variation across time, the number of dietary records completed by the participants may have an impact on the less robust associations, as suggested by our sensitivity analyses. Consequently, caution is needed in interpreting associations that are not consistent across all sensitivity analyses.*”

3) In the abstract, the authors should highlight the results which were consistent in every sensitivity analysis.

and

4) In the abstract, the authors should note some findings were not robust in sensitivity analyses.

+++ Consistently, we have now focused the presentation of the findings in the abstract on the associations that remained robust throughout all sensitivity analyses, and we have added a sentence to mention that some associations were suggested in the main models but not robust in all sensitivity analyses. The abstract now reads:

*“Overall,* *2,604 incident cancer cases were diagnosed during follow-up (including 750 breast, 322 prostate, and 207 colorectal cancers). Higher intakes of mono- and diglycerides of fatty acids (E471) were associated with higher risks of overall cancer (HR _high vs. low category_ = 1.15; 95%CI [1.04,1.27], p-trend=0.01), breast cancer (HR = 1.24; 95%CI [1.03,1.51], p-trend=0.04) and prostate cancer (HR = 1.46; 95%CI [1.09,1.97], p-trend=0.02). In addition, associations with breast cancer risk were observed for higher intakes of total carrageenans (E407 and E407a) (HR = 1.32; 95%CI [1.09,1.60], p-trend=0.009) and carrageenan (E407) (HR = 1.28; 95%CI [1.06,1.56], p-trend=0.01). No association was detected between any of the emulsifiers and colorectal cancer risk.* *Several associations with other emulsifiers were observed but were not robust throughout sensitivity analyses. Main limitations include possible exposure measurement errors in emulsifiers intake and potential residual confounding linked to the observational design.”*

-----------------------------------------------------------

Editorial Comments:

i) In line with the Academic Editor comments, the results for the sensitivity analysis must be accurately reflected in the manuscript (the results and tables can remain in the SI, but needs to be discussed in the abstract, results and discussion)

+++ As mentioned above, we now focus the abstract and the results section on the emulsifiers for which associations remained robust in all sensitivity analyses. We have now discussed in the abstract, results and discussion the fact that some associations were significant in the main models but not in some sensitivity analyses.

ii) Include absolute risk (not just focus on relative risk)

+++ Absolute incidence rates according to categories of emulsifier intakes, standardized by age and sex, are now presented in eTable3.

-----------------------------------------------------------

Comments from the reviewers:

Reviewer #1: The authors have addressed my points. WHile checking the reision I noticed that in the supplement, specifically eFigure 4, some of the text is in French. I think most people will recognise the French for cumulative incidence functions and probability but "ensembles de covariables" left me scratching my head.

While the authors are editing the file is it possible to include hyperlinks from the table of contents to the main sections? It is quite a chunky document to page through.

Michael Dewey

+++ Thank you and sorry for the typo. We have now replaced « ensemble de covariables » by « Categories of emulsifier intakes » and have included hyperlinks in table of contents.

Reviewer #2: Comments have been dealt with well. One more minor comment.

1. In Table 1, the missing value rows state "N (%)" but only the N is provided. It would be useful to have the % as well for each of the variables.

+++ Thank you. The % have been added to the table.

Reviewer #3: Thank you for your detailed and thoughtful response to my comments. An already very good manuscript is now much improved.

+++ Thank you very much!

Any attachments provided with reviews can be seen via the following link:

__________________________________________________

In compliance with data protection regulations, you may request that we remove your personal registration details at any time. (Use the following URL: <https://www.editorialmanager.com/pmedicine/login.asp?a=r>). Please contact the publication office if you have any questions.
